# Comparison of Chemical Transport Model and Observation Derived Ozone-NO_*x*_‑VOC Isopleths for the South Coast Air Basin of California

**DOI:** 10.1021/acsestair.6c00227

**Published:** 2026-09-08

**Authors:** Zongrun Li, Yu Qian, Yongtao Hu, Petros Vasilakos, M. Talat Odman, Armistead G. Russell

**Affiliations:** † School of Civil and Environmental Engineering, 1372 Georgia Institute of Technology, Atlanta, Georgia 30332, United States; ‡ PSI Center for Energy and Environmental Sciences, 28498 Paul Scherrer Institute PSI, Villigen 5232, Switzerland

**Keywords:** CMAQ, HDDM, ozone isopleth, least squares

## Abstract

We developed, applied, evaluated, and compared chemical transport model-based and observation-based methods for constructing ozone isopleths used to understand ozone-emission relationships, focusing on the South Coast Air Basin of California (SoCAB). The High-order Decoupled Direct Method (HDDM) embedded in the Community Multiscale Air Quality (CMAQ) model was applied under different emission levels to map out the sensitivities of ozone levels to anthropogenic emissions of nitrogen oxides (NO_*x*_) and volatile organic compounds (VOCs). Ozone concentrations and sensitivities to emissions simulated by CMAQ-HDDM were used to develop ozone isopleth diagrams for monitoring locations across the SoCAB, and those isopleths were compared with observation-derived isopleths. The CMAQ-HDDM- and observation-derived isopleths reproduced the observed ozone concentrations (*R* ^2^ = 0.70 and 0.96, respectively) and showed better agreement, especially at inland locations, which typically experience higher ozone levels than central or coastal sites. The responses of ozone to NO_*x*_ and VOC emissions were compared over time, based on historical VOC and NO_*x*_ emission levels. The sensitivities to NO_*x*_ emissions showed a stronger correlation (*R* ^2^ = 0.75) between the CMAQ-HDDM- and observation-derived methods than the sensitivities to VOC emissions (*R* ^2^ = 0.18). For most regions in SoCAB, both the CMAQ-HDDM- and observation-derived isopleths indicate that the ozone regime has transitioned from VOC-limited to NO_*x*_-limited around the late 2000s. As a result, for equal absolute emission changes, further reductions in NO_*x*_ emissions are more effective than comparable VOC controls in achieving future ozone decreases, with greater confidence for inland cities in the SoCAB.

## Graphical abstract

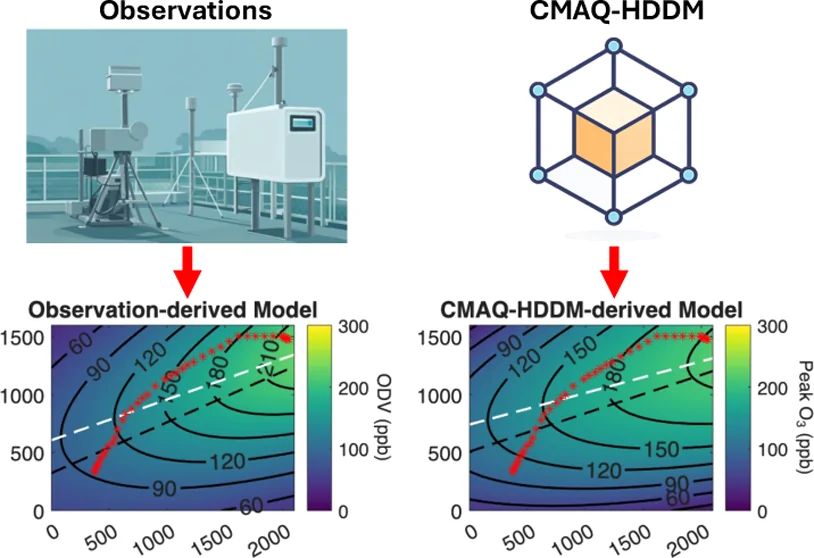

## 1 Introduction

The South Coast Air Basin (SoCAB) of California has the highest peak ozone levels in the U.S. despite stringent controls imposed over the last few decades. Although significant ozone reductions have been realized, recent trends have found a leveling off in the ozone design value (ODV, defined by the average of the fourth-highest daily maximum 8-hour ozone concentration in three consecutive years, and it is the metric used for attainment determination in the U.S., using the average of three fourth-highest values to reduce the impact of single episodic conditions^1^) and even an increase in the middle of the 2010–2020 decade. These observations have heightened interest in further understanding the response of ozone levels to changes in precursor emissions, mainly volatile organic compounds (VOCs) and nitrogen oxides (NO_*x*_). Ozone (O_3_) formation is governed by nonlinear photochemical processes involving NO_*x*_-VOC interactions, resulting in regime-dependent sensitivities.^2^ Consequently, emission reductions may increase or decrease ozone levels depending on whether the atmosphere is NO_*x*_- or VOC-limited. A better quantification of ozone sensitivity to changes in precursor emissions can help guide effective future emission control policies.

Multiple methods can be applied to understand ozone sensitivities to precursor emissions or pollutants, including using ground monitoring data, smog chambers, box models, airborne measurements, statistical or machine learning models, and chemical transport models (CTMs).^3–12^ Ambient observations and chamber experiments capture ozone formation processes under realistic atmospheric conditions, providing valuable and relatively direct constraints on ozone sensitivity regimes. However, these approaches are inherently limited in spatial coverage, constrained to locations where monitoring sites exist or field campaigns are conducted. Pusede and Cohen established an empirical framework using long-term ground monitoring data of NO_*x*_ and O_3_ (1995–2010) to diagnose ozone-NO_*x*_ sensitivities.^9^ To isolate confounding meteorological effects, they implemented a temperature-stratification approach and analyzed ozone exceedance probabilities within identical temperature bins. By leveraging the weekday-to-weekend shift in heavy-duty traffic emissions as a natural proxy for controlled NO_*x*_ reductions, they used the resulting response slopes (Δozone exceedance probability/ΔNO_2_) to distinguish between chemical regimes, where a negative slope denotes a VOC-limited regime and a positive slope denotes a NO_*x*_-limited regime. Subsequently, Nussbaumer and Cohen refined this methodology by substituting O_3_ with total odd oxygen (O_*x*_ = O_3_ + NO_2_), an informative metric for evaluating ozone budgets that remains conserved under intense localized NO titration.^10^ Perdigones et al. further extended this observational analysis by using hourly changes in O_*x*_ to estimate ozone production rates and their sensitivity to NO_*x*_, and by estimating HO_*x*_ production from VOC reactivity and an analytical steady-state radical model.^13^ Applying this enhanced approach to the Los Angeles Basin (1994–2019), they found that major urban receptors, such as North Main Street, Reseda, and San Bernardino, have successfully transitioned from VOC-limited to NO_*x*_-limited regimes on extreme ozone exceedance days (>100 ppb). Wu et al. deployed transportable smog chamber systems at Pasadena and Redlands during the 2021 RECAP-CA campaign to determine peak ozone sensitivity to precursor emissions.^11^ By capturing real-time ambient air and systematically perturbing it via VOC additions or NO_*x*_ manipulations (both enrichment and dilution), they directly measured empirical ozone responses to precursor variations. Furthermore, they integrated these chamber observations with an observationally constrained 0-D photochemical box model to construct ozone isopleths, enabling a precise evaluation of ozone regimes in the SoCAB. Pfannerstill et al. used airborne eddy-covariance VOC flux measurements over Los Angeles to quantify summertime emitted OH reactivity and ozone formation potential.^14^ They reported that biogenic terpenoid emissions contribute approximately 60% to these quantities, implying that the control of NO_*x*_ is key to reducing ozone formation in Los Angeles. Moore et al. trained random-forest models using meteorological simulations and hourly ground-monitoring NO_*x*_ and ozone observations to diagnose summertime peak-hour ozone production regimes across the basin.^12^ The study found that the LA urban core, immediate downwind areas, and coastal sites remained VOC-limited on most ozone-season days as of 2021, while downwind inland basins, such as south and east of San Bernardino, are NO_*x*_-limited.

CTMs, such as the Community Multiscale Air Quality (CMAQ) model, can predict ozone concentrations under different emission levels, which can be used to understand ozone sensitivities by running multiple simulations (a.k.a., brute force method).^8,15,16^ Additionally, CTMs provide broader spatial coverage and can be applied to assess the impacts of future emission control policies on ozone levels, such as the zero-emission vehicle policy.^17,18^ However, this approach can be computationally expensive. The High-order Decoupled Direct Method (HDDM)^19^ provides an effective alternative and has been successfully used to estimate ozone sensitivities in previous studies.^19,20^ For example, a high-order integral method, combined with a limited number of CMAQ-HDDM simulations, can be employed to efficiently generate ozone isopleths, offering a computationally efficient solution.^20^ However, CTM results have biases due to uncertainties in their meteorological and emissions inputs, and simplifications in transport and chemical processes. Mesoscale turbulence schemes commonly used in CMAQ, such as Reynolds-Averaged Navier–Stokes (RANS) models, cannot explicitly resolve large eddies that affect the vertical mixing and transport of ozone precursors.^21^ Moreover, current U.S. inventories underestimate total VOC emissions from volatile chemical products (VCPs) by a factor of 2–3 nationally and regionally while simultaneously overestimating mobile-source VOCs by ∼40%.^22^ Qin et al. found that the National Emission Inventory (NEI) 2011 underestimates per-capita VCP VOC emissions by a factor of ∼3–4 relative to bottom-up estimates from SHEDS^23^ and McDonald et al.,^22^ and scaling the California NEI VCP emissions by 3 times brought CMAQ predictions of organic PM_2.5_ and O_3_ into closer agreement with Pasadena observations. In addition, chemical species are often lumped to simplify chemical reactions and reduce computational complexity. For example, existing simplified mechanisms were primarily developed to represent VOC chemistry from mobile sources, and they inadequately capture VCP chemistry. Key VCP species, including oxygenated VOCs, siloxanes, and low-volatility compounds are typically mapped to hydrocarbon surrogates or treated as non-reactive, leading to substantial underestimation of both ozone and secondary organic aerosol (SOA) formation from VCPs.^24–27^ Recent mechanism development efforts have sought to address these gaps. The Community Regional Atmospheric Chemistry Multiphase Mechanism (CRACMM) introduced explicit treatment of oxygenated intermediate-volatility organic compounds (IVOCs), including propylene glycol and a lumped siloxane-containing species, and expanded coverage of low-to intermediate-volatility compounds.^28^ Similarly, the development of the RACM2B-VCP mechanism has improved simulations of VCP-related chemistry, though biases in VOC reactivity and SOA formation remain.^27^ These simplifications introduce certain limitations in representing fine-scale turbulence and detailed chemistry, leading to a potential bias in ozone concentration simulations.

Among the methods for understanding ozone precursor emission sensitivities, the use of isopleths is an attractive approach to visualize and investigate ozone-emission relationships and has been used widely in analyses of air quality control strategies.^11,29–31^ Isopleths visually illustrate how ozone levels respond to variations in precursor emissions. The “ridge line” of the ozone isopleth, defined as the locations where ozone sensitivities to NO_*x*_ and VOC emissions are equal, is often used to indicate regions where NO_*x*_ and VOC emission controls are equally effective. Additionally, the ozone isopleth is an effective diagram to identify areas where reducing NO_*x*_ or VOC emissions may increase ozone levels. Observation-derived ozone isopleths are based on anthropogenic VOC and NO_*x*_ emissions estimates and ozone observations, which have been explored in a previous study.^32^ In that work, ozone was assumed to follow quadratic and log-quadratic response functions to anthropogenic NO_*x*_ and VOC emissions, with parameters determined by fitting ozone observations and emission estimates using the least-squares method. This method is considerably less computationally intensive than CMAQ-derived (or CMAQ-HDDM) approaches for constructing ozone isopleths. However, historical emission estimates from 1980 to 2019 are subject to uncertainty, reducing the model’s accuracy.

In this study, we conducted CMAQ-HDDM modeling of the SoCAB under several historical emission levels and projected future emission scenarios to quantify ozone sensitivity to changes in anthropogenic NO_*x*_ and VOC emissions. Then, we developed a novel method to derive ozone isopleths based on modeled ozone, as well as its first-order and second-order derivatives with respect to anthropogenic NO_*x*_ and VOC emissions. Instead of using the high-order integration method presented in the previous study,^20^ we assumed the ozone isopleths follow quadratic and log-quadratic response functions and then used the least-squares method to determine parameters in the functions. The method uses more concise formulations and can potentially be applied to other regions beyond the SoCAB. Moreover, the method can extend isopleth construction under future emission scenarios, such as electrification^17,33^ or temperature-dependent emission changes due to global warming,^14^ that fall outside the historically observed emission range, and that observation-derived isopleths have limitations in resolving. While other model-based methods (e.g., using a large number of simulations, followed by fitting a response surface) can also be used to construct isopleths, the use of HDDM effectively uses fewer simulations. Since observation-derived isopleths also employ the least-squares method, an interesting point is that both approaches use the same functional form to construct ozone isopleths, yet they rely on different data sources: observations versus modeled ozone and its derivatives. This raises questions about how well the two methods compare overall, how their predicted responses to emission reductions differ, and what these comparisons imply about future ozone sensitivity to anthropogenic VOC and NO_*x*_ emission changes. For understanding these questions, we compared HDDM-derived with observation-derived isopleths to provide more insights into the ozone-emissions response. Also, we analyzed the evolution of ozone sensitivity from historical conditions to projected future emission scenarios, providing implications for future emission control strategies.

## 2 Materials and Methods

### 2.1 CMAQ Model, Modeling Domain, and Period

In this study, we applied CMAQ version 5.0.2 with the HDDM^34–36^ to simulate pollutant concentrations and estimate both first- and second-order ozone sensitivities to anthropogenic VOC and NO_*x*_ emissions. CMAQ-HDDM is derived by differentiating the full three-dimensional governing equation, including advection, turbulent diffusion, chemistry, emissions, and deposition, with respect to emissions. The resulting sensitivities at a given grid cell therefore represent the response of O_3_ at that location to emission perturbations across the air parcel’s evolution in the basin, considering the full three-dimensional transport, mixing, chemical aging, and deposition propagated through the model. CMAQ-HDDM simulation using the Carbon Bond 6 (CB6) mechanism^37^ was conducted from May 1, 2016, to July 31, 2016 (details of CMAQ configurations can be found in Table S1). Based on ozone observations, this three-month period covers most of the high-ozone days during a typical year in the SoCAB regions and includes two of the highest ozone periods in 2016, from June 2 to June 4 and from July 20 to July 29. The year 2016 was chosen as it is the base year for scientific and regulatory modeling using a specially constructed and evaluated 2016 NEI. A nested-grid modeling approach was employed, with the outer domain covering the contiguous U.S. at a 12 km horizontal resolution, and the inner domain covering the SoCAB region at a 4 km horizontal resolution (Figure S1). The Weather Research & Forecasting Model version 3.9 (WRF)^38^ was applied to provide meteorological conditions for CMAQ simulations. The initial and boundary conditions of WRF simulations were provided by the North American Mesoscale Forecast System (NAM) analysis dataset.^39^ Land use in the WRF simulations was based on the National Land Cover Database (NLCD) 2011.^40^ The grid nudging and observational nudging were conducted using National Centers for Environmental Prediction (NCEP) upper air and surface air observational datasets.^41,42^ Details on the WRF configuration options can be found in Table S2. WRF performance for the simulation period was well within the guidelines (Table S3).

We used the same meteorological conditions (2016 ozone season) with different anthropogenic emission levels representing 1985, 2001, 2011, 2016, and 2028 in CMAQ simulations to separate meteorological influences from emissions changes. Biogenic emissions in each simulation were the same. Anthropogenic emissions inputs for the years 2001, 2011, 2016, and 2028 were developed using the EPA’s emissions modeling platforms,^43^ and the versions and inventories are provided in Table S4. Since there was no spatially and temporally detailed inventory available for 1985, we constructed the 1985 emissions by hindcasting the 2016 inventory, accounting for changes in sources, including industrial, transportation, population-linked activities, and population distributions. The spatial redistribution of our emissions estimates was conducted using census data^44^ and source and county-specific emission spatial distribution from the California Air Resources Board (CARB) (Figure S2). We applied the same VOC speciation profile as in 2016 and varied only the total emission magnitude for 1985 VOC emissions. This may underestimate VOC reactivity, since the pre-catalyst mobile fleet of the 1980s emitted a more aromatic, more reactive VOC mix than present-day profiles.^45^ We compared our NEI-based emission estimates with the CARB inventory for the studied years and found that the total levels and trends between these inventories were consistent (Tables S5–S9, Figures S3 and S4). However, differences exist between the total annual emissions reported by CARB and NEI, particularly for coarse particulate matter (PMC), defined as PM_10_ minus PM_2.5_. These discrepancies can be partly attributed to differences in the emissions modeling systems underlying the two inventories. For example, CARB uses the EMission FACtor (EMFAC) model, whereas NEI relies on MOtor Vehicle Emission Simulator (MOVES) for on-road emissions.^46^ Then, Sparse Matrix Operator Kernel Emissions (SMOKE) programs were used to generate hourly, gridded emissions required by CMAQ as inputs. The biogenic emissions were provided by the Biogenic Emission Inventory System (BEIS),^47^ which online estimates the biogenic emissions using meteorological conditions and land cover. Therefore, because both meteorology and land use were held fixed, the biogenic emissions remained unchanged across the CMAQ-HDDM scenarios. Since Fresno, Inyo, Monterey, and San Benito counties were not entirely within the 4 km modeling domain, the total emissions used in CMAQ were only a fraction of the county total emissions reported for these counties in Tables S5–S9.

### 2.2 Developing Ozone Isopleths

In this study, we developed ozone isopleths (ozone response surfaces under different anthropogenic NO_*x*_ and VOC emissions) for 24 monitoring sites across SoCAB using either CMAQ-HDDM simulations or observational data, as these sites provided more continuous and comprehensive records over the study period (site names and locations are available on the project website^48^ and Figure S1). Our analysis focused on high-ozone days, using the maximum daily 8-hour average (MDA8) ozone concentrations (modeled or observed). We focused on these high-ozone periods because the regulatory standard is defined by the ODV. For observation-derived isopleths, we used ODV directly. For CMAQ-HDDM-derived isopleths, rather than simulating ODV, which would require three consecutive years of simulations and substantial computational resources, we used peak MDA8-O_3_ and the maximum of each first- and second-order sensitivity from the 13 days high-ozone modeling period as a surrogate. These 13 days were selected as the highest-ozone days of the 2016 ozone season based on observed MDA8-O_3_ (the June 2–4 and July 20–29 episodes), and the same 13 days were used for all emission scenarios so that differences among scenarios reflect emission changes rather than meteorology. Because ODV is governed by the highest MDA8-O_3_ days within a year, peak episodic MDA8-O_3_ captures the photochemical regime most relevant to the regulatory metric. Also, across all sites and years, these two are strongly correlated with a near-unity slope (Figure S5; slope = 0.94 and *R* ^2^ = 0.83), indicating that the episodic peak reproduces the ODV magnitude at historical emission levels. As such, our analyses represented ozone responses under conditions conducive to ozone formation, rather than typical days.

#### 2.2.1 Developing CMAQ-HDDM-derived Isopleths

We conducted 15 CMAQ-HDDM simulations under different emission scenarios (Figure 1 and Table S10). Five simulations used historical or projected emissions for the years 1985, 2001, 2011, 2016, and 2028 (black circles in Figure 1). Using 2016 as the baseline, we conducted an additional four simulations with small perturbations in NO_*x*_ and VOC emissions (green stars). To capture ozone sensitivity under relatively low NO_*x*_ and/or VOC conditions, we included six more simulations (red triangles). These six simulations extend beyond the range of historical and projected emission levels and provide ozone concentrations and sensitivity estimates under low-NO_*x*_ and/or low-VOC conditions, thereby reducing reliance on extrapolated estimates in those regions.

**Figure 1:**
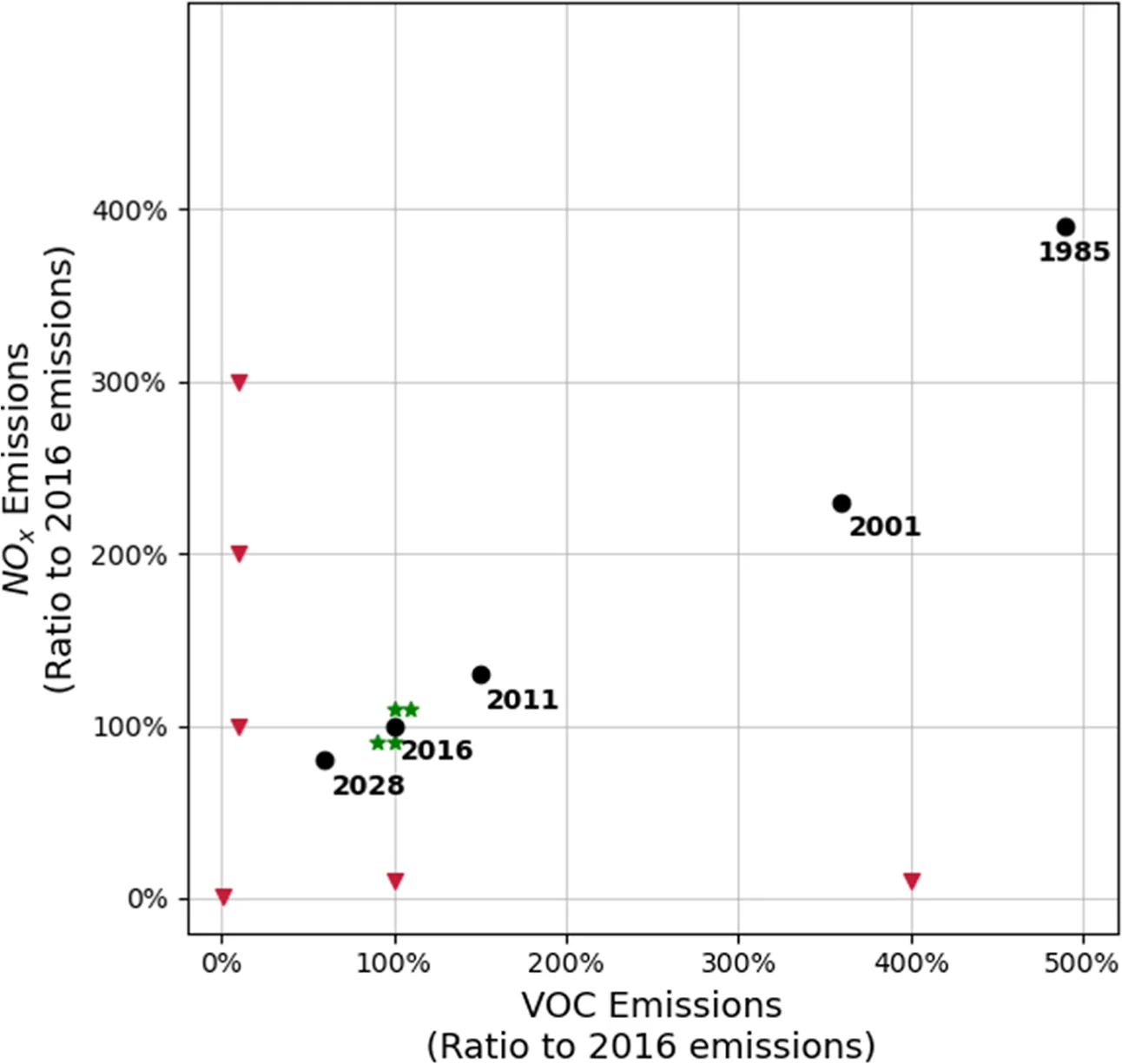
Different emission levels used in CMAQ-HDDM simulations (detailed emission levels are shown in Table S10).

In this study, an innovative approach was developed to construct ozone isopleths using both first- and second-order sensitivities. First, we assumed ozone response follows a quadratic (quadratic model) or a log-quadratic (log-quadratic model) response function to NO_*x*_ and VOC emissions

1 $$\begin{array}{l} O_{3}\left(E_{{NO}_{x}},E_{VOC}\right)\text{or}\log\left(O_{3}\left(E_{{NO}_{x}},E_{VOC}\right)\right)\\ =\beta +\alpha_{{NO}_{x}}E_{{NO}_{x}}+\alpha_{VOC}E_{VOC}+\alpha_{{NO}_{x}-VOC}E_{{NO}_{x}}E_{VOC}+\alpha_{{{NO}_{x}}^{2}}{E_{{NO}_{x}}}^{2}+\alpha_{{VOC}^{2}}{E_{VOC}}^{2} \end{array}$$

From eq 1, O_3_ (*E*_NO*x*_, *E*_VOC_) is the ozone response function under NO_*x*_ emission level *E*_NO*x*_ and VOC emission level *E*_VOC_. β, α_NO*x*_, α_VOC_, α_NO*x*‑VOC_, α_NO*x*_^2^, and α_VOC_^2^ are the parameters we need to estimate using different emission levels and corresponding CMAQ-HDDM simulations. With eq 1, we can derive the mathematical formulae for the first- and second-order sensitivities as

2 $$\{\begin{array}{l} \frac{\partial O_{3}}{\partial E_{{NO}_{x}}}\text{or}\frac{\partial {\log O}_{3}}{\partial E_{{NO}_{x}}}=\alpha_{{NO}_{x}}+\alpha_{{NO}_{x}-VOC}E_{VOC}+2\alpha_{{{NO}_{x}}^{2}}E_{{NO}_{x}}\\ \frac{\partial O_{3}}{\partial E_{VOC}}\text{or}\frac{\partial {\log O}_{3}}{\partial E_{VOC}}=\alpha_{VOC}+\alpha_{{NO}_{x}-VOC}E_{{NO}_{x}}+2\alpha_{{VOC}^{2}}E_{VOC}\\ \frac{\partial^{2}O_{3}}{\partial E_{{NO}_{x}}\partial E_{VOC}}\text{or}\frac{\partial^{2}\log O_{3}}{\partial E_{{NO}_{x}}\partial E_{VOC}}=\alpha_{{NO}_{x}-VOC}\\ \frac{\partial^{2}O_{3}}{\partial {E_{{NO}_{x}}}^{2}}\text{or}\frac{\partial^{2}\log O_{3}}{\partial {E_{{NO}_{x}}}^{2}}=2\alpha_{{{NO}_{x}}^{2}}\\ \frac{\partial^{2}O_{3}}{{\partial E_{VOC}}^{2}}\text{or}\frac{\partial^{2}{\log O}_{3}}{{\partial E_{VOC}}^{2}}=2\alpha_{{VOC}^{2}} \end{array}$$

3 $$E_{{NO}_{x}}=-\frac{\alpha_{{NO}_{x}}+\alpha_{{NO}_{x}-VOC}E_{VOC}}{2\alpha_{{{NO}_{x}}^{2}}}$$

4 $$E_{{NO}_{x}}=\frac{\left(2\alpha_{{VOC}^{2}}-\alpha_{{NO}_{x}-VOC}\right)E_{VOC}+\left(\alpha_{VOC}-\alpha_{{NO}_{x}}\right)}{2\alpha_{{{NO}_{x}}^{2}}-\alpha_{{NO}_{x}-VOC}}$$

Then, the least-squares method can be conducted using the simulated ozone concentrations and first- and second-order sensitivities under different emission levels to find the optimized parameters by minimizing the least-squares differences between the left- and right-hand sides of eqs 1 and 2 simultaneously. To place the concentration and sensitivity constraints on comparable magnitudes, emissions were normalized by the 2016 base-scenario emissions before fitting. The fitted response functions and their derived sensitivities were subsequently transformed back to physical emission units, from which the diagnostic lines were calculated. Specifically, the zero-NO_*x*_-sensitivity and equal-NO_*x*_-VOC-sensitivity lines are calculated from the ozone response function (eq 1) using eqs 3 and 4, respectively. For a given emission level, its position relative to the zero-sensitivity and ridge lines indicates the sign of $\frac{\partial O_{3}}{\partial E_{{NO}_{x}}}\,\;\text{or}$ identifies the NO_*x*_- or VOC-limited regime (detailed mathematical derivations are provided in Text S1).

### 2.3 Developing Observations-derived Isopleths

An alternative way to develop ozone isopleths at monitoring sites is to directly use the ODV observations and corresponding anthropogenic emission levels, and the method was described in the previous study.^32^ This method assumes that ozone responds to anthropogenic NO_*x*_ and VOC emissions according to a quadratic or a log-quadratic function, as shown in eq 1. In this study, we primarily focus on using the log-quadratic model to develop observation-derived isopleths, as the previous study^32^ indicated that this model had lower relative uncertainties and avoided negative ozone predictions. Unlike CMAQ-HDDM, observational data do not provide first- or second-order sensitivities. Therefore, the least-squares method is applied solely to minimize the differences between the left- and right-hand sides of eq 1 to determine the optimized parameters. Although this approach lacks explicit sensitivity information for parameter estimation, observation-derived isopleths benefit from a continuous long-term record (1980–2019) of co-varying emission estimates and ozone observations.

## 3 Results

In this section, we first evaluated our CMAQ simulations by comparing them with observations. Then, we developed ozone isopleths using the CMAQ-HDDM simulations with quadratic and log-quadratic models, compared their performance, and quantified their uncertainties. Additionally, we also developed ozone isopleths using observations and conducted comparison analyses to understand the discrepancies between ozone isopleths developed using CMAQ-HDDM and observations. For the analyses presented here, we mainly used Crestline, a downwind location experiencing some of the highest ozone levels in the SoCAB, as the primary example to illustrate the method and its uncertainty. For the other cities in SoCAB, similar analyses can be found in the Supporting Information (Figures S10–S15, Tables S13–S16) and on the project website.^48^

### 3.1 CMAQ Performance Evaluation

The 2016 CMAQ simulation results were evaluated by comparing simulated and corresponding observed pollutants, including MDA8-O_3_, daily-averaged CO, and daily-averaged NO_2_ (Figures S6 and S7). For MDA8-O_3,_ which is the primary focus of the study, the performance generally met the criteria (statistical levels achieved by approximately two-thirds of historical applications) and either met or came very close to meeting the goal (performance comparable to the top one-third of historical applications) suggested by a previous study,^49^ indicating that the CMAQ simulation shows reasonable performance (Table S11). Since there are no sensitivity observations, we cannot directly evaluate the performance of the first- and second-order sensitivities estimated by HDDM. However, by using the observation-derived ozone isopleth, we can evaluate the HDDM first-order sensitivities. We compared HDDM-simulated $\partial O_{3}/E_{NO_{x}}$ and ∂O_3_/*E*_VOC_ with the corresponding observation-derived sensitivities across 24 sites over four simulated years (1985, 2001, 2011, and 2016; Figure S8 and Table S12). The R^2^ and RMSE values were 0.50 and 0.04 ppb/(tons per day) for $\partial O_{3}/E_{NO_{x}}$ and 0.15 and 0.04 ppb/(tons per day) for ∂O_3_/*E*_VOC_, respectively. HDDM-simulated sensitivities in more recent years aligned well with the observation-derived estimates. Larger discrepancies appeared in 1985, where HDDM-simulated $\partial O_{3}/E_{NO_{x}}$ was higher and ∂O_3_/*E*_VOC_ was lower than the observation-derived sensitivities. This can be partly explained by the larger uncertainties in NO_*x*_ and VOC emission estimates for earlier years and can also impact the observation-derived ozone isopleths.

Given that the meteorology used was from 2016, not 1985, 2001, or 2011, a rank-ordered method was used to assess how well the model captured high ozone levels experienced in years other than 2016 (Figure S9). Specifically, we first identified peak ozone periods of each individual year with the same length of 13 days based on the regional mean MDA8-O_3_ across all monitoring sites. Then, daily MDA8-O_3_ concentrations at all monitoring sites were extracted for each day in the identified period, and these values were ranked from high to low. The same ranking procedure was applied to the corresponding simulated concentrations. Both 2011 and 2016 comparisons showed high consistency between the simulated and observed MDA8-O_3_ concentrations (Figure S9). These results indicate that the CMAQ simulations captured peak ozone values over our simulated periods, though peak ozone results for 1985 and 2001 were biased low by about 20% compared to the observations. Multiple potential reasons could have led to such bias, including a bias in the estimated emissions, a mismatch in the meteorology (using 2016 meteorological conditions that may differ from those in 1985 and 2001), and systematic biases in the CMAQ chemistry mechanism.^50,51^ Based on the 2011 and 2016 results, which are more recent years with detailed NEI emissions, we found that the model can capture peak ozone levels. So, uncertainties in emissions are probably the leading cause of the ozone bias between simulations and observations, especially in 1985 and 2001, when the discrepancies between observed and simulated ozone are relatively large (Figure S9). To understand the impacts of emissions over these two years, we adjusted the levels by increasing VOC emissions by 40% and decreasing NO_*x*_ emissions by 20%, then compared these with the original simulations to assess their effect on simulated ozone levels. For 1985, simulated MDA8-O_3_ was 22% lower before the adjustment. The ozone levels increased and showed better agreement in capturing the observed ozone intensity after the emission adjustments, though they were biased higher (Figure S9). For 2001, the simulated MDA8-O_3_ slightly increased due to the emission adjustment and showed improved agreement (Figure S9). This analysis implies that the primary cause of the underestimation of peak ozone levels relates to the emissions estimates for 1985 and 2001.

Although the simulated ozone levels for 2011 and 2016 were more consistent with the observed ozone levels, this does not necessarily mean that the emissions for those years were less biased compared to other years, as the ozone-emissions response differed for each year. The effect of emissions bias can vary depending on the emission level. For instance, we conducted the same emission adjustment for 2011 and 2016, and the results showed that simulated ozone levels would not change significantly (Figure S9), indicating that the ozone levels were much less sensitive to emissions in 2011 and 2016 than in 1985 and 2001. Thus, it is possible that bias in estimated emissions exists at all times, rather than only for certain years.

### 3.2 CMAQ-HDDM-derived Isopleths

Ozone isopleths for the 24 monitoring sites were developed by fitting quadratic and log-quadratic models to simulated ozone concentrations and their sensitivities to VOC and NO_*x*_ emissions across 15 emission levels (Figures 2 and S10). A least-squares approach was applied to minimize the squared differences between CMAQ-HDDM-simulated and model-predicted ozone concentrations and first- and second-order sensitivities.

**Figure 2:**
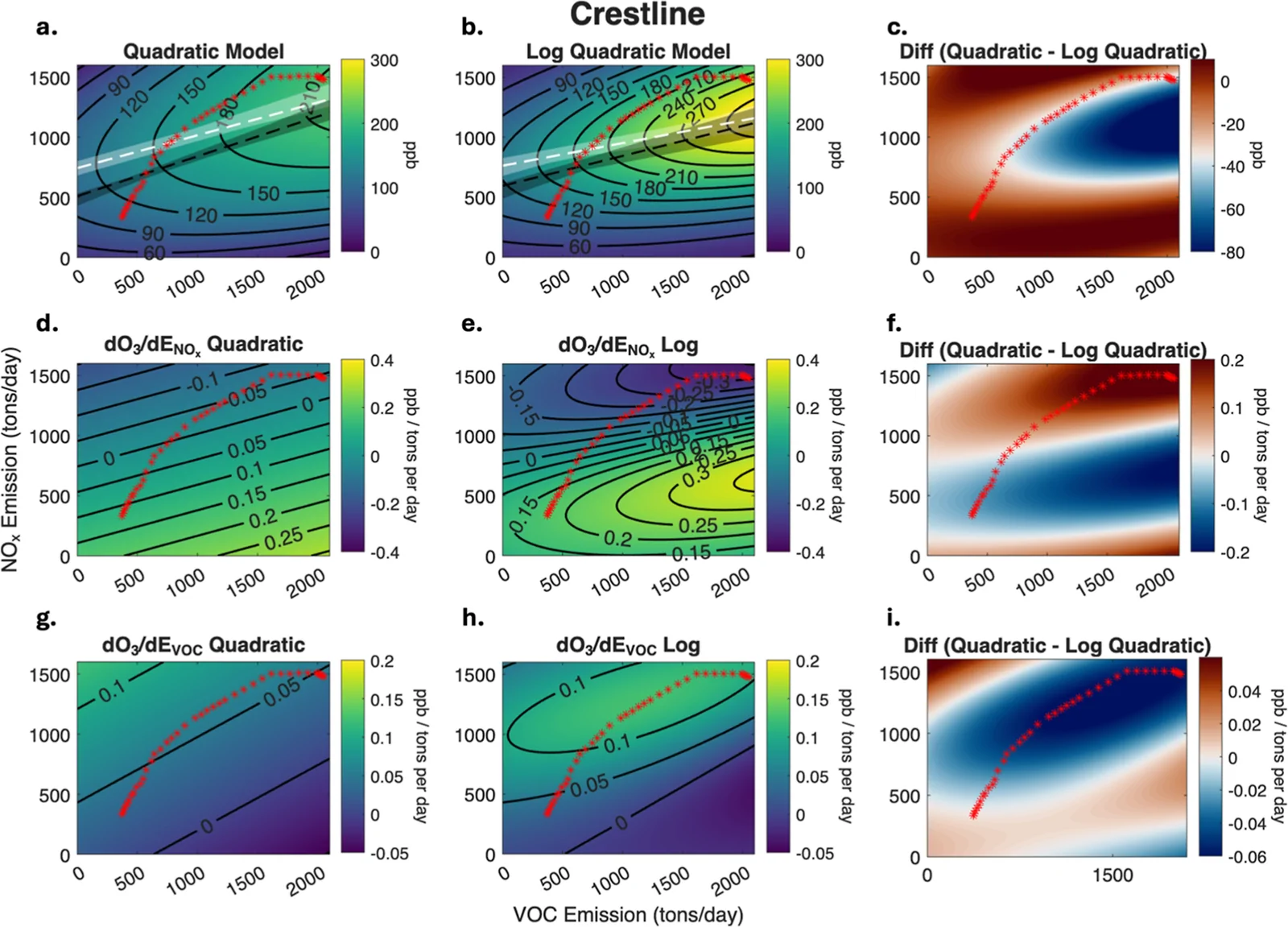
Comparisons between the CMAQ-HDDM-derived quadratic model and log-quadratic model ozone-emission concentrations and sensitivity isopleths for Crestline, California. The first column (panels a, d, and g) shows the quadratic model-based isopleths. The second column (panels b, e, and h) shows the log-quadratic model-based isopleths. The third column (panels c, f, and i) shows the difference between those two. The first row (panels a, b, and c) shows the ozone concentration isopleths; the second row (panels d, e, and f) shows the ozone-to-NO_*x*_ emissions sensitivity isopleths; and the third row (panels g, h, and i) shows the ozone-to-VOC emissions sensitivity isopleths. The black dashed line indicates the zero-NO_*x*_-sensitivity line, and the white dashed line indicates the equal-NO_*x*_-VOC sensitivity line. The shaded area around each line spans the full range of the 15 leave-one-out fits, representing the uncertainty in the construction of the CMAQ-HDDM-derived ridge line, and does not include potential impacts from emissions, meteorological, and other model input uncertainties. The red points show the historical emission trajectory from 1980 to 2019.

To assess the performance of the least-squares fitting, we compared CMAQ-HDDM-simulated ozone concentrations and first-order sensitivities with the values predicted by the least-squares models (Figures 3 and S11). For Crestline, the quadratic model reproduced ozone concentrations well, and the first-order sensitivity of ozone to NO_*x*_ showed higher correlation and lower RMSE compared to the log-quadratic model. In contrast, the two models exhibited similar performance in reproducing the first-order sensitivity of ozone to VOC. Similar results were also found for the other sites in SoCAB (Figure S11, Tables S13 and S14), indicating the quadratic model had more agreement with CMAQ-HDDM simulated concentrations and ozone-to-NO_*x*_ sensitivity. For the quadratic model, the agreement between CMAQ-HDDM-simulated ozone and the model-reproduced ozone was strongest, followed by ozone-to-NO_*x*_ sensitivities, and weakest for ozone-to-VOC sensitivities. The log-quadratic model captured the ozone concentration better than ozone-to-VOC sensitivities, followed by ozone-to-NO_*x*_ sensitivities. For ozone reproduction performance, both quadratic- and log-quadratic-form isopleths reproduced CMAQ-HDDM-simulated ozone, with R^2^ values exceeding 0.51 and 0.33, respectively, except for the LA North Main and coastal sites West LA, LAX, and Long Beach (Table S13). Ozone at coastal sites was more influenced by meteorological conditions, which may introduce additional variability and reduce reproductive performance. For ozone-to-NO_*x*_ sensitivity, both models tended to underestimate sensitivity when CMAQ-HDDM simulations indicated high values. The relatively poorer reproduction of sensitivities to VOC and NO_*x*_ emissions, compared to ozone concentration (Tables S13 and S14), can be attributed to the magnitude differences between ozone and its first-order derivatives. The least-squares method naturally prioritizes variables with larger magnitudes (e.g., ozone) to minimize the overall squared error.

**Figure 3:**
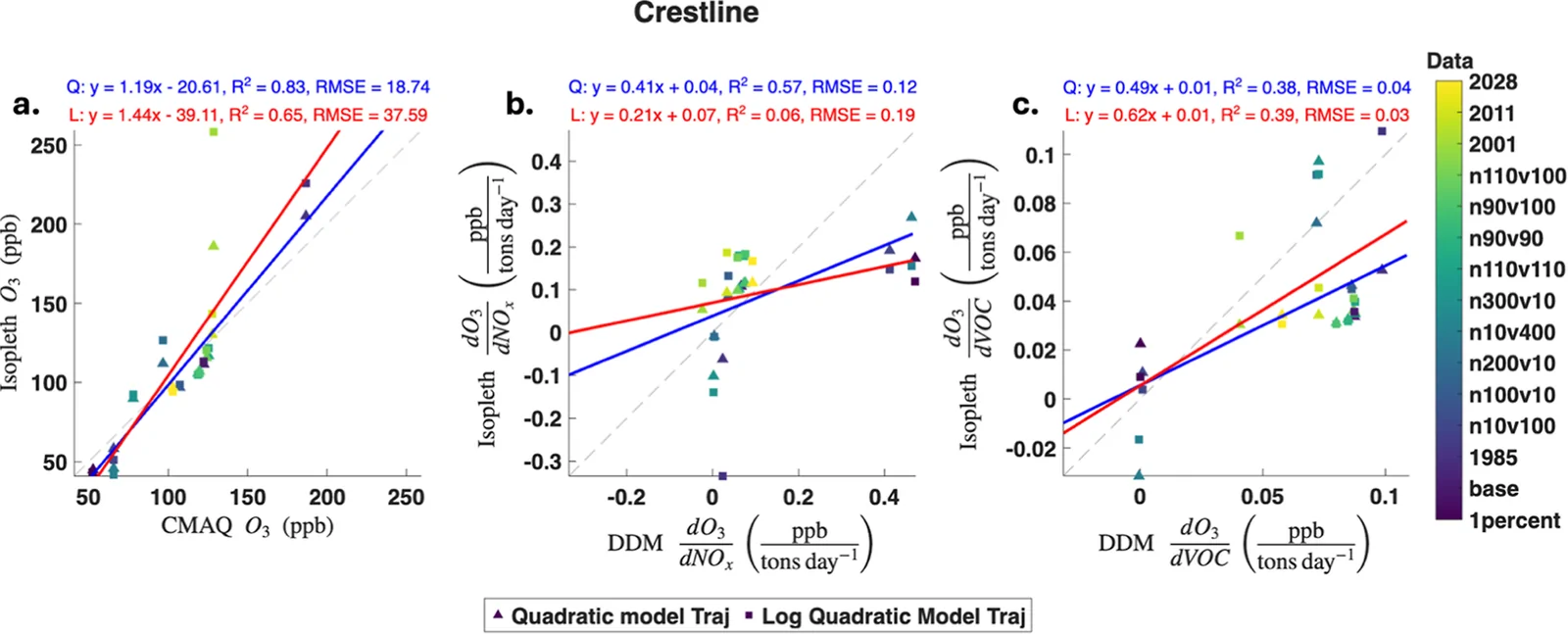
Comparisons between CMAQ-HDDM estimates and values reconstructed using the quadratic and log-quadratic models, including ozone (panel a), ozone to NO_*x*_ sensitivity (panel b), and ozone to VOC sensitivity (panel c) at Crestline, California. Comparisons between CMAQ-HDDM and the quadratic model are shown as triangle markers, while those between CMAQ-HDDM and the log-quadratic model are shown as square markers. The red and blue lines represent the regression relationships between CMAQ-HDDM and the quadratic and log-quadratic models, respectively. Point colors indicate different emission levels used in the CMAQ-HDDM simulations (e.g., n110v100 represents a scenario with NO_*x*_ emissions at 110% of the 2016 level and VOC emissions at 100% of the 2016 level. Detailed definitions of emission levels for each scenario are provided in Table S10).

On the isopleths, SN = SV and SN = 0 lines are identified (Figures 2 and S10), where SN is the first-order sensitivity of ozone concentration to NO_*x*_ emissions ( $\frac{\partial O_{3}}{\partial E_{{NO}_{x}}}$ ), and SV is the first-order sensitivity of ozone concentration to VOC emissions ( $\frac{\partial O_{3}}{\partial E_{VOC}}$ ). The SN = SV line (white dashed line) divides the emissions space into two major regimes: above this line is identified as radical-limited or the VOC-limited regime, where VOC emissions reduction is more effective for reducing ozone concentrations than NO_*x*_ emissions reduction for equal absolute emission changes. The region below this line represents the NO_*x*_-limited regime, where reducing NO_*x*_ emissions is more effective for ozone control. The SN = 0 line (black dashed line) marks the NO_*x*_ emission control transition: above this line, decreasing NO_*x*_ emissions increases ozone (SN < 0, often referred to as the radical-limited regime), whereas below it, decreasing NO_*x*_ emissions decreases ozone. For Crestline, both models indicated that the ozone regime transitioned from VOC-limited to NO_*x*_-limited from previous years’ emission levels to recent years, as illustrated by the red dots in Figure 2. The log-quadratic model tends to predict higher ozone concentrations than the quadratic model and shows large discrepancies when the emission levels are high. For sensitivities, large discrepancies can also be found in the regions with high emission levels. In recent years, when emission levels were low, and more CMAQ-HDDM simulations were available (lower-left region in Figure 2, near the 2016 baseline emissions, which correspond to approximately 396 and 401 tons/day for NO_*x*_ and VOC, respectively), the differences between the quadratic and log-quadratic models were smaller. As observed at Crestline, the results from these two methods at the other sites generally agree in capturing the transition from VOC-limited to NO_*x*_-limited ozone regimes. However, the quadratic model predicts lower ozone concentrations at high emission levels and higher concentrations at low emission levels compared to the log-quadratic model, and this pattern was also observed in other locations (Figure S10).

### 3.3 Model and Isopleth Uncertainty Analysis

We evaluated the uncertainty of the developed isopleths and sensitivity lines using data withholding. We used the model developed using all 15 data points as the reference and refit the isopleths 15 times, each time withholding one case. For the isopleth, the mean and standard deviation of the differences were calculated to evaluate the model’s uncertainty (Figures 4 and S12). For the sensitivity lines, we re-derived each line from the 15 withheld-point models and quantified its uncertainty as the full range (minimum to maximum) spanned by the 15 resulting lines, shown as a shaded band around the sensitivity line derived from the reference model (Figures 2 and S10).

**Figure 4:**
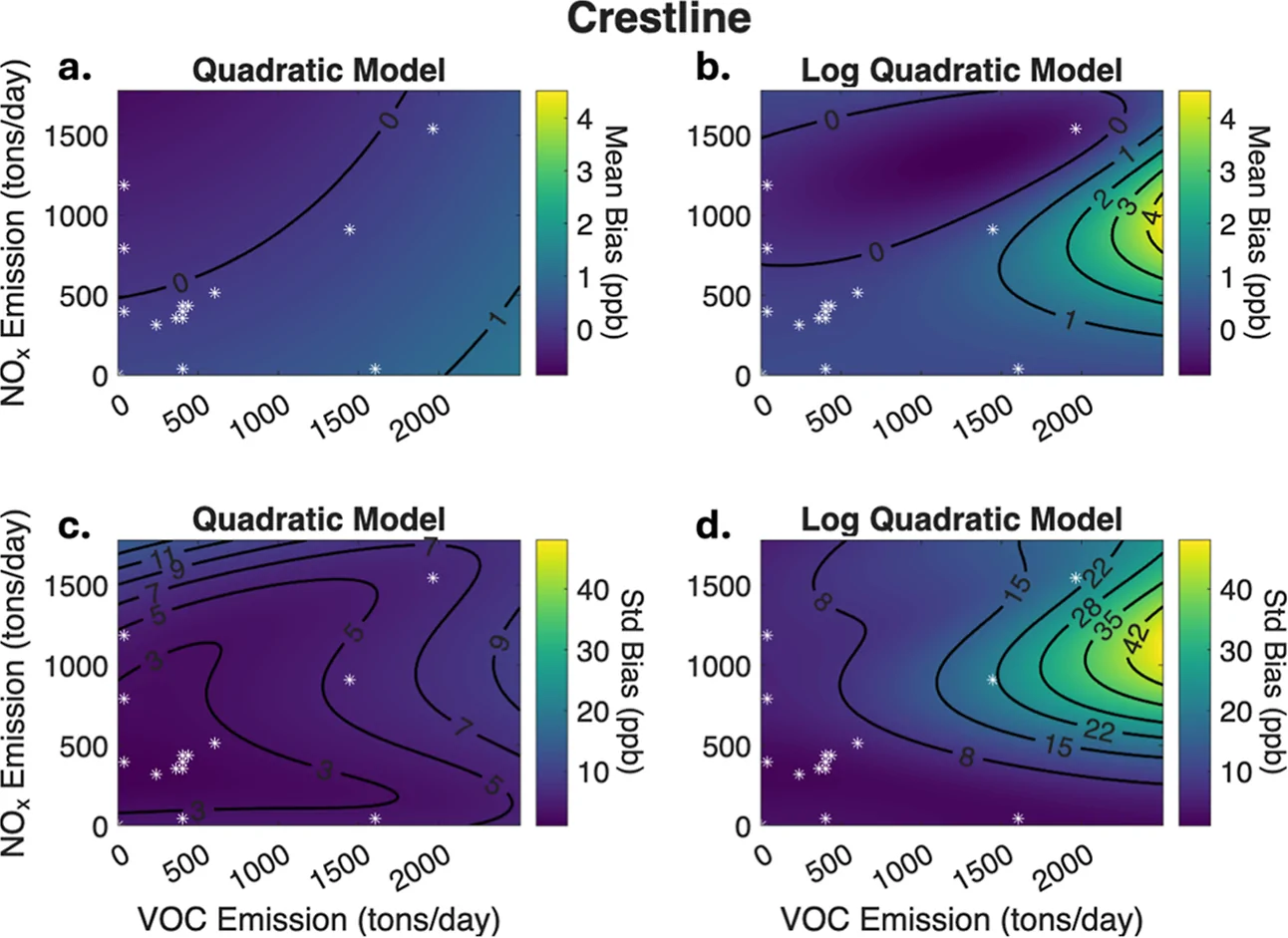
Mean bias (panels a and b, first row) and standard deviation of bias (panels c and d, second row) of CMAQ-HDDM-derived isopleths. The first column (panels a and c) and the second column (panels b and d) show the uncertainties of the isopleths developed using the quadratic model and the log-quadratic model, respectively. The white crosses indicate the 15 emission levels used in the isopleth development.

For ozone isopleths, mean bias and standard deviation of biases increased as emissions increased due to a lack of data in high ozone level areas. Biases were relatively low in the region where NO_*x*_ and VOC emissions were at low levels (Figures 4 and S12). Consistent with this, the uncertainty band of the sensitivity lines is comparable to or narrower for the quadratic model than for the log-quadratic model, especially at low emission levels (Figures 2 and S10). As multiple CMAQ-HDDM simulations are concentrated around the 2016 baseline emission scenario (*E*_NOx_ = 396 tons/day and *E*_VOC_ = 401 tons/day), withholding one data point near the baseline has relatively less impact on the ozone isopleth fitting. Neighboring data points provide similar ozone concentration or sensitivity information, thereby maintaining the stability of the fitted ozone isopleth or sensitivity lines. In contrast, the number of high-emission scenario cases is limited, which leads to large uncertainties when withholding such a data point. However, since the emission control policy is steady, the regions where the emissions are lower rather than higher are of greater interest. In this case, both the quadratic and log-quadratic models estimate ozone with a mean bias below 3 ppb and a standard deviation of bias below 13 ppb for SoCAB sites (Figure S12).

Comparing the quadratic and log-quadratic models, the quadratic model exhibits relatively lower mean and standard biases, particularly in regions where VOC emissions exceed 1500 tons per day (Figures 4 and S12). Consistent with this, the uncertainty band of the sensitivity lines is comparable to or narrower for the quadratic model than for the log-quadratic model, especially at low emission levels (Figures 2 and S10). Additionally, the log-quadratic model tends to produce steeper gradients because its sensitivity follows an exponential form, whereas the quadratic model’s sensitivity is linear at higher ozone concentrations. This difference also explains the larger uncertainties associated with the log-quadratic model. Overall, although the log-quadratic model prevents the negative ozone predictions, its steep gradient, especially at high-emission regions, may require more CMAQ-HDDM simulations to constrain the uncertainty. Therefore, the quadratic model performs better for developing CMAQ-HDDM-derived ozone isopleths using these 15 simulations.

### 3.4 Observational-derived and CMAQ-HDDM-derived Ozone Isopleths Comparisons

We compared the quadratic-form CMAQ-HDDM-derived isopleth with the log-quadratic observation-derived isopleth (Figures 5 and S13), as these two models showed better performance in the previous section’s analyses and the study by Qian et al.^32^ From the ozone concentration isopleths, both models indicate that the ozone formation regime in Crestline shifts from VOC-limited to NO_*x*_-limited due to the emission controls (the historical emission trajectories are shown as red points in Figures 5 and S13). When both emissions were high (*E*_NO*x*_ > 1000 tons/day and *E*_VOC_ > 1500 tons/day), which was the level during and before the 1990s, the HDDM-based method tended to estimate ozone concentrations slightly lower than the observation-based estimation. This can be explained by underestimations of ozone concentrations in the 1985 CMAQ-HDDM simulations, likely caused by biases in the early-year emissions inventory. In the middle range of both NO_*x*_ emissions (from 400 to 1000 tons/day) and VOC emissions (from 0 to 1500 tons/day), which have been the emission levels since 2000 and are expected for future years under continued emission control policies, the HDDM-based method tended to estimate ozone concentrations higher than the observation-based estimation for most of the sites. Since the observation-based model used the ODV, which is the average of three consecutive years’ fourth-highest MDA8-O_3_ concentrations, whereas the HDDM-derived isopleths used peak MDA8-O_3_ concentrations during high ozone periods, the peak ozone tends to be higher with HDDM-derived isopleths. Large discrepancies also appeared when NO_*x*_ emissions were lower and close to zero, where the ozone levels estimated by observation-derived isopleths tended to be higher than those from CMAQ-HDDM-derived isopleths. This is because the observation-derived isopleths were driven by historical emission levels that did not reach zero-NO_*x*_ emissions, while the CMAQ-HDDM-derived isopleths explicitly included cases with very low NO_*x*_ and VOC emissions (1% to 10% of the baseline 2016 emissions). As a result, the CMAQ-HDDM-derived isopleth likely better captures ozone responses under low emission levels, which is essential for accurately estimating ozone levels under future emission controls and informing the formulation of new air quality standards.

**Figure 5:**
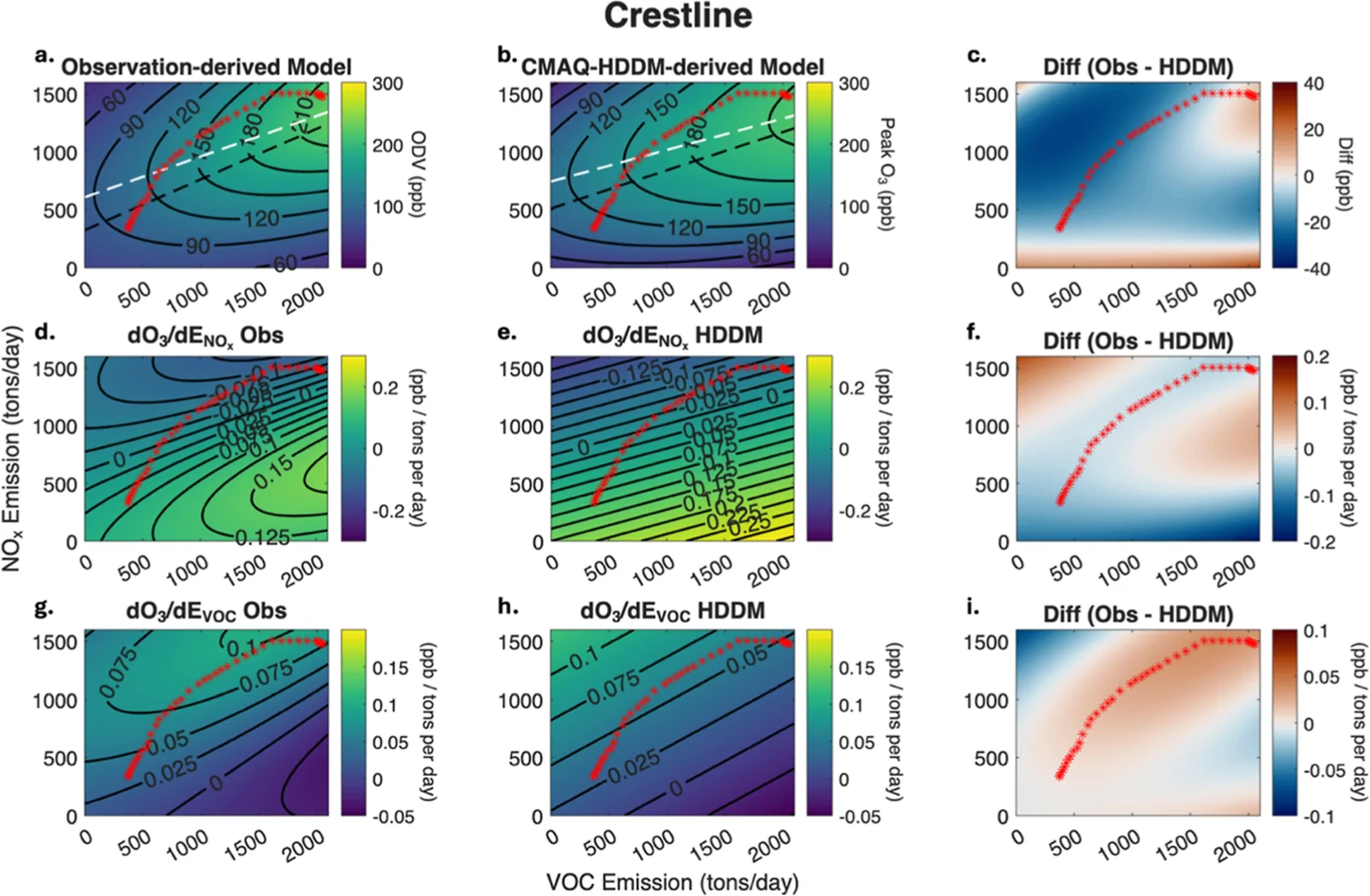
Comparisons between the CMAQ-HDDM-derived (quadratic model) and the observation-derived (log-quadratic model) ozone concentration and sensitivity isopleths for Crestline. The first column (panels a, d, and g) shows the observation-derived isopleths. The second column (panels b, e, and h) shows the CMAQ-HDDM-derived isopleths. The third column (panels c, f, and i) shows the difference between those two. The first row (panels a–c) shows the ozone concentration isopleths and their differences; the second row (panels d–f) shows the ozone-to-NO_*x*_ emissions sensitivity isopleths and their differences; and the third row (panels g–i) shows the ozone-to-VOC emissions sensitivity isopleths and their differences. The black dashed line indicates the zero-NO_*x*_-sensitivity line, and the white dashed line indicates the equal-NO_*x*_-VOC sensitivity line. The red points show the historical emission trajectory from 1980 to 2019.

The ozone to NO_*x*_ and VOC sensitivities also show similar patterns between the quadratic-form CMAQ-HDDM-derived isopleth and the log-quadratic observation-derived isopleth. The ozone-NO_*x*_ sensitivity increased from the low-VOC/high-NO_*x*_ region to the high-VOC/low-NO_*x*_ region, shifting from negative to positive (Figures 5 and S13). Conversely, the ozone-VOC sensitivity decreased from the low-VOC/high-NO_*x*_ region to the high-VOC/low-NO_*x*_ region, transitioning from positive to negative. The large discrepancies in ozone-to-NO_*x*_ sensitivity can be found in regions where NO_*x*_ emissions are low (<400 tons/day), with the CMAQ-HDDM-based model producing higher sensitivity estimates, or where VOC emissions are low (<500 tons/day) and NO_*x*_ emissions are high (>1300 tons/day), with the observation-based model reporting higher values. For ozone-to-VOC sensitivity, large differences can be found when VOC emissions are low (<500 tons/day) and NO_*x*_ emissions are high (>1200 tons/day), with the CMAQ-HDDM-based model reporting higher sensitivities.

A further evaluation of the differences between CMAQ-HDDM-derived isopleths and observation-derived isopleths can be made by comparing the estimated historical trends of ozone concentrations and ozone-to-emissions sensitivities (along emissions trajectories indicated by red points in Figures 5 and S13). For Crestline, CMAQ-HDDM-derived isopleths had high correlations with observed ozone and sensitivities estimated by observation-derived isopleths (*R* ^2^ > 0.83) (Figure 6). Note that the ozone concentrations predicted by the observation-derived isopleths were not the same as the observed values due to least-squares reconstruction errors, although the differences were small. Across all sites in SoCAB, the CMAQ-HDDM-derived isopleths agreed well with the observation-derived isopleths along the historical emission trajectory, with an overall R^2^ of 0.70 and an RMSE of 28.54 ppb (Figure S14, Tables S15 and S16). The observed ozone trends were well captured (*R* ^2^ > 0.52) using the CMAQ-HDDM-derived isopleth, except for West LA, Long Beach, and LAX (Table S15). These three stations are near the coast, where meteorological conditions can play an important role in affecting the ozone predictions, which were not fully considered in CMAQ-HDDM simulations, as we used the same 2016 meteorological conditions to drive the simulations. Also, the CMAQ-HDDM-based ozone levels tended to be a bit lower for earlier years (during and before the 1990s) and higher after the year 2000 at more than half of the sites (Figures 6 and S14).

**Figure 6:**
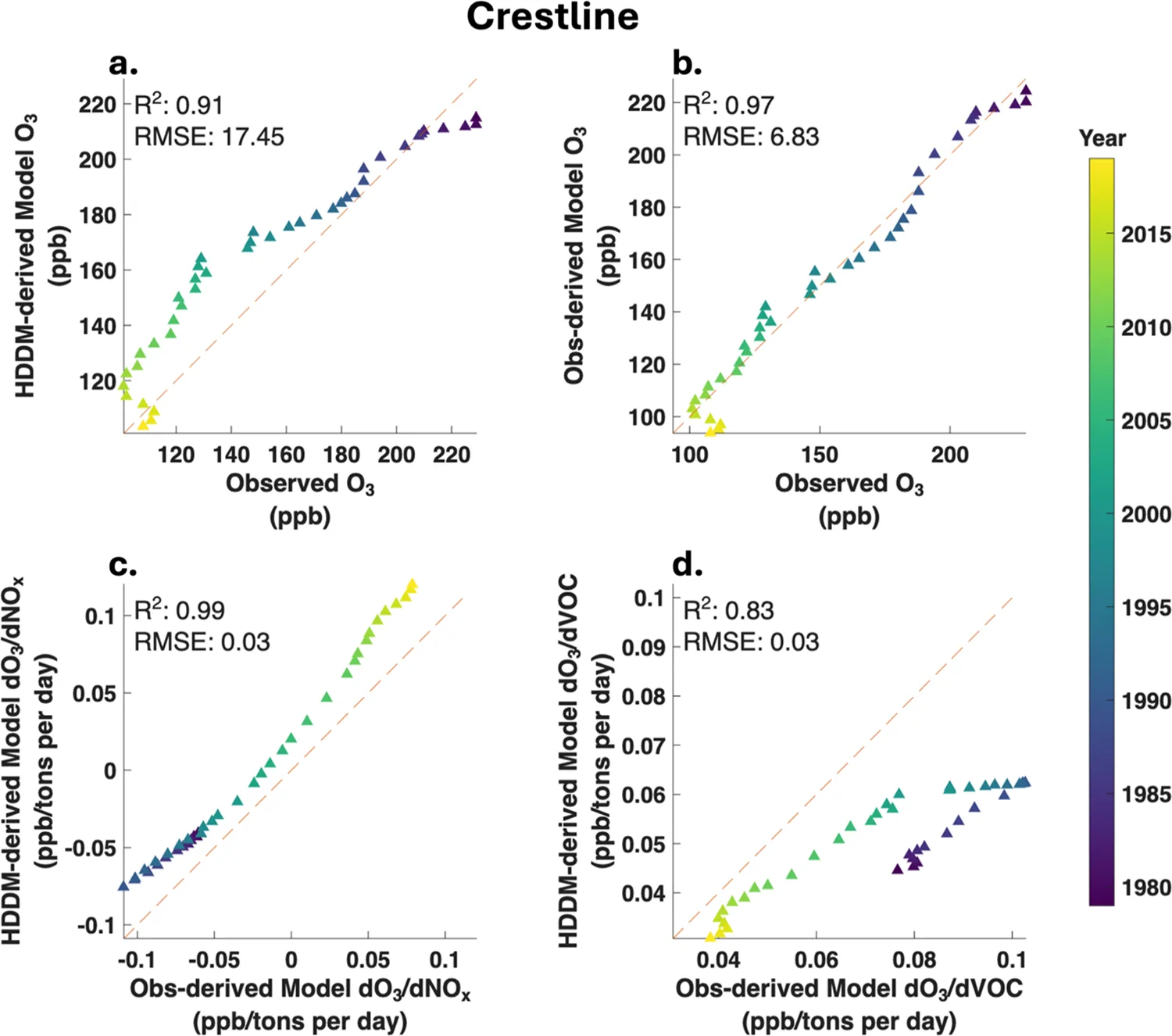
Comparison of observed ozone with CMAQ-HDDM- and observation-derived model estimates along emission levels from 1980 to 2019 (panels a and b, first row), and comparisons between HDDM-derived and observation-derived sensitivity trends along emission levels from 1980 to 2019 (panels c and d, second row). The color indicates the year of the points. The dashed line indicates the 1:1 reference line.

For ozone sensitivity to emissions, we observed that both CMAQ-HDDM-based and observation-based methods indicated that SN shifted from negative to positive from 1985 to recent years, while SV still remained positive in 2019. The SN from both models agreed well with each other, where *R* ^2^ was higher than 0.88, except for Pico Rivera and LAX. For SV, the correlations between these two models were relatively low for most of the sites, and the *R* ^2^ ranged from 0.00 to 0.89, indicating the spatial heterogeneity of *R* ^2^. Also, CMAQ-HDDM-derived SV tended to vary less compared to the SV predicted by the observation-based model, which generally showed a decreasing trend from the 1980s onwards. However, the decrease in these models’ estimated SV was not monotonic at all sites. For example, the SV increased from 1980 to 1990, but then generally decreased from 1990 to recent years in Crestline.

## 4 Discussion

We simulated and analyzed the ozone concentrations and their sensitivities to NO_*x*_ and VOC emissions using CMAQ-HDDM simulations over the SoCAB. Then, we developed CMAQ-HDDM-derived ozone isopleths for 24 monitoring locations in the SoCAB using quadratic and log-quadratic models. The quadratic model shows better agreement in ozone and sensitivities with CMAQ-HDDM simulations used in fitting the models and has lower uncertainties, especially in regions where emission levels were high, compared to the log-quadratic model. However, the quadratic form model has a potential risk of negative ozone predictions, though such negative ozone values were not found for the studied cities and emission levels.

We compared the quadratic-form CMAQ-HDDM-derived isopleth and the log-quadratic observation-derived isopleth for the studied SoCAB cities. Importantly, both the model-derived and observationally driven approaches provide similar isopleths, supporting the use of both approaches. While other model-based approaches should lead to similar isopleths, HDDM with the least-squares fitting effectively uses the sensitivities to construct the isopleths. The two methods show good agreement in NO_*x*_ sensitivity (*R* ^2^ = 0.75, RMSE = 0.04 ppb/(tons per day)), while the agreement in VOC sensitivity is more moderate (*R* ^2^ = 0.18) with a comparable RMSE (0.05 ppb/(tons per day)). Both methods showed that VOC reduction was generally more effective for ozone concentration mitigation prior to approximately 2000 to 2005, whereas NO_*x*_ emissions reduction became more effective thereafter. From the ozone isopleths derived from observations or CMAQ-HDDM, we found that at inland cities, where emissions are the dominant driver of peak ozone, recent years’ emission levels are located in the NO_*x*_-sensitive regime, well below the zero-SN line and the SN = SV line, which suggests that ozone levels are more sensitive to NO_*x*_ reductions than to VOC reductions for equal absolute emission changes. Perturbations on emissions in CMAQ further confirm this pattern, showing results consistent with the ozone isopleth analysis. For the same emission perturbations (20% NO_*x*_ decrease and 40% VOC increase), the peak ozone concentration changes were more significant for earlier years, while no obvious changes under recent years’ emission levels, indicating reduced ozone-to-VOC sensitivity in recent years. For future ozone control, strategies can still focus on NO_*x*_ emission reduction. For example, the recent electric vehicle adoption could further reduce NO_*x*_ emissions in California, and a recent study indicated that ozone could be reduced due to the policy.^17^ The results from both methods also revealed some similar spatial distribution patterns. Firstly, the ozone concentrations were higher over the inland area of the basin and lower over the coastal area. Though this pattern has been consistent over the past four decades, the declining rate of ozone concentration over the basin is not evenly distributed. The ozone-to-NO_*x*_ emissions sensitivity also reflected a similar pattern. In inland regions, SN started mostly negative and kept increasing to positive around 2000 to 2005 across different monitoring sites. This clearly showed how the response of ozone to NO_*x*_ emissions changed and indicated that the NO_*x*_ emissions controls did not decrease ozone in the past in the inland area, but have been increasingly beneficial. On the other hand, the ozone-to-VOC emissions sensitivities started positive but have decreased over time in inland regions, while remaining positive. Spatially, SV was higher in inland areas and lower along the coast, indicating that the inland area of the basin benefited more from VOC reduction than the coastal area.

The observation- and CMAQ-HDDM-based methods have discrepancies, as these methods use different data sources to construct ozone isopleths. The ozone concentrations from observations or CMAQ simulations are similar, but the emission sensitivities are different. The CMAQ-HDDM-derived sensitivity isopleths relied on simulation-provided sensitivities, while the observation-derived isopleths calculated sensitivities using long-term ozone observations and precursor emission estimates. Large discrepancies were found when emission levels are extremely low or high, since the observation-derived isopleths lack such extreme emission levels in their training data. The HDDM-derived isopleths can better capture ozone sensitivities under low NO_*x*_ emissions, which is critical for estimating projected ozone under future emissions, as NO_*x*_ emissions are expected to decrease. The discrepancies between ozone isopleths developed using CMAQ-HDDM and observations also display certain spatial patterns, as the isopleths tend to be quite different for the sites along the coast. For example, the observation-derived isopleths suggest that at recent emission levels in LAX, a coastal area, ozone response falls within the VOC-limited regime, whereas the CMAQ-HDDM-derived isopleths indicate that the site is NO_*x*_-limited. During summer daytime, which corresponds to the typical ODV observation period, sea breezes generally blow from the ocean inland. Inland, downwind sites, such as Crestline, receive aged air masses in which NO_*x*_-VOC-O_3_ photochemistry has largely proceeded, so peak ozone is primarily a function of emissions, which is well captured by CMAQ-HDDM-derived and observation-derived isopleths. Coastal sites, by contrast, are strongly influenced by sea-breeze recirculation, marine-layer dynamics, NO titration near fresh sources, and transport variability, leading to greater meteorological impacts on peak ozone. As a result, the CMAQ-HDDM-derived model reproduces observed ozone less well at these sites (Tables S15 and S16), leading to larger discrepancies with the observation-derived isopleths. This spatial pattern is consistent with prior empirical modeling in the SoCAB.^52^ The study using a generalized additive model that incorporated both meteorology and precursor emissions similarly reported poorer model performance at coastal sites than at inland sites.

Both observation- and CMAQ-HDDM-based methods have advantages and disadvantages in developing ozone isopleths. The observation-derived isopleths more accurately replicate ozone concentrations and responses at historical emission levels, as the method is trained on historical observations and corresponding estimated emissions. However, predictions of ozone concentrations or sensitivities for future or near-zero anthropogenic emission levels, which are expected to be lower, may have large uncertainties, given the limited historical data resembling these future conditions for reliable forecasting. This limitation is not specific to the observational isopleth approach used here;^32^ observation-based methods more broadly, including analyzing monitoring data,^9,10,13,14^ chamber experiments,^11^ and machine-learning models trained on measurements,^12^ are all anchored to observed conditions and similarly cannot or have limitations in resolving the future, counterfactual, or near-zero anthropogenic emission regimes that fall outside the historical record and that can guide policy developments. Nevertheless, some observation-based methods use weekday-weekend variability in NO_*x*_ emissions, which can be as large as a 50% decrease, to improve estimates of ozone sensitivities to precursor emissions.^9,10,12,13,53^ Since observation-derived isopleths do not require chemical transport model simulations, the method provides a fast and computationally efficient solution for guiding near-future emission control strategies for reducing ozone when the data is available. In contrast, CMAQ-HDDM-based methods benefit from counterfactual emission scenario simulations for better understanding ozone and its sensitivities under future emission levels, especially when these levels significantly differ from current levels, such as in the case of intensive adoption of electric vehicles. Additionally, not all regions have monitoring networks as dense as those in SoCAB. For regions with limited air quality monitoring, CMAQ-HDDM-derived isopleths can be constructed to assess ozone responses to emission changes at different locations, accounting for spatial and temporal variations in ozone sensitivity. This complementarity is supported by our comparison analyses. Although the CMAQ-HDDM-derived isopleths are constructed without using observed ozone, they independently reproduce the inland VOC-limited to NO_*x*_-limited regime transition identified by the observation-derived isopleths. The resulting present-day pattern, in which inland, downwind receptors are the most NO_*x*_-limited while the urban core and coastal areas remain comparatively VOC-limited, is consistent with recent observational and chamber-based studies of the Los Angeles basin.^10–12^ Building on this consistency, future work could use observations to further constrain and improve the CMAQ-HDDM simulations, both to better reproduce historical ozone sensitivities and to provide spatially resolved sensitivities in locations lacking monitoring.

Limitations of the CMAQ-HDDM-based approach for constructing ozone isopleths remain. First, we applied the same 2016 meteorological conditions across all CMAQ-HDDM simulations for different years. This is done intentionally to focus on the impact of emissions on peak ozone levels and is supported by past studies that have examined the impact of inter-annual temperature changes on peak ozone levels.^54,55^ However, temperature increases associated with global warming can introduce uncertainties into long-term ozone simulations since warming increases biogenic VOC emissions,^56^ temperature-dependent VCP emissions,^14^ and peroxyacetyl nitrate (PAN) decomposition rates.^57^ So, using fixed 2016 meteorology may overestimate biogenic emissions in earlier years, given the warming trend, though it may also underestimate them due to land-use changes. As a result, the CMAQ-HDDM-derived isopleths may indicate a somewhat earlier or later VOC-limited to NO_*x*_-limited transition than would be inferred if year-specific changes in biogenic emissions were accounted for. However, anthropogenic emission controls can still be one of the dominant factors driving peak ozone in historical years or the near future. The observation-derived isopleths implicitly incorporate year-specific historical meteorology, since they are constructed from actual observed ozone. The good agreement between CMAQ-HDDM-derived and observation-derived isopleths on the direction of the regime transition (Figures 5, S13 and Table S15 and S16) indicates that changes in precursor emissions play an important role in ozone controls, especially at inland sites. Additionally, previous studies that separated anthropogenic emissions and meteorological impacts on peak ozone also indicated that anthropogenic emission controls are the critical driver of observed historical ozone trend,^52,54,55^ although meteorological influences may not be negligible, particularly at coastal locations and during anomalous climate conditions such as El Niño years. Future studies could further refine CMAQ-HDDM-derived ozone isopleths by incorporating projected meteorological conditions and updated, evolving chemical mechanisms under development now, such as the CRACMM,^28^ which more explicitly represents VCPs, an important source of anthropogenic VOCs in urban areas. Additionally, the method assumed that the ozone-emission response follows a quadratic or log-quadratic form. Higher-order formulations (e.g., cubic or quartic) can introduce additional noise (showing up as increased curvature) in the isopleths, though with little additional accuracy. To verify this, we fit a cubic ozone response function at all 24 sites in SoCAB and evaluated its uncertainty using the same leave-one-out cross-validation procedure (Figure S15). The cubic model exhibited substantially higher uncertainty than the quadratic model, which can be attributed to its four additional coefficients (10 in total, versus 6 for the quadratic model) being poorly constrained by the 15 available CMAQ-HDDM scenarios. The results indicate that the quadratic form represents a practical balance between capturing nonlinear ozone chemistry and maintaining robust parameter identifiability under a feasible simulation number. Also, the squared errors between model-predicted and CMAQ-HDDM-simulated ozone concentrations, as well as first- and higher-order sensitivities, would be treated with equal weight in the least-squares method after being normalized by 2016 emission levels. A weighted least-squares approach could be explored; however, this would require a better understanding of the uncertainties associated with CMAQ-HDDM simulations of both concentrations and sensitivities. With such an understanding, the CMAQ-HDDM simulations with higher uncertainties can be assigned lower weights to improve isopleth construction. Additionally, this study only discussed ozone concentration responses to anthropogenic emissions. Although such ozone isopleths are critical for guiding ozone control strategies, the economic effectiveness of controlling VOC or NO_*x*_ emissions can differ, which is not accounted for in the derived ozone isopleths. Moreover, we developed the ozone isopleths site by site, and the models do not provide spatially continuous ozone predictions under different emission levels. Future studies can follow these methods for developing ozone isopleths as a function of emissions but consider including spatial variables such as latitude and longitude. With such ozone isopleths that consider spatial variations, one can estimate ozone levels for each county. By combining county-level ozone concentrations under different emission levels with population data, ozone exposures can be estimated, providing a health-impact perspective to inform ozone control policies.

## Supplementary Material



## Data Availability

Data and code are available at: https://github.com/zli867/HDDMOzoneIsopleth (accessed on August 27, 2026). Details of ozone isopleth analyses for each monitoring site in SoCAB are also available at the project website: https://zli867.github.io/projects/CMAQ-DDM/index.html (accessed on August 27, 2026).

## Notes

Supporting Information model configuration; meteorological and emissions information; mathematical derivations; emission scenarios; performance evaluations; site-specific ozone-isopleth analyses; Supporting Information text; 15 figures; and 16 tables (PDF)
