# Supplementary material for: Comparison of Chemical Transport Model and Observation Derived Ozone-NO x ‑VOC Isopleths for the South Coast Air Basin of California
Source: ACS EST Air. 2026 Sep 8;3(10):2767–80. doi: 10.1021/acsestair.6c00227 (PMC13660400; doi:10.1021/acsestair.6c00227)
Supplement: Supplementary file 1 [file ea6c00227_si_001.pdf]

Supplemental Material for

Comparison of Chemical Transport Model and Observation Derived Ozone-NO_x-VOC Isopleths for the South Coast Air Basin of California

Zongrun Li[1], Yu Qian[1], Yongtao Hu[1], Petros Vasilakos[2], M. Talat Odman[1], Armistead G. Russell[1, *]

[1] School of Civil and Environmental Engineering, Georgia Institute of Technology, Atlanta, GA 30332, United States

[2] PSI Center for Energy and Environmental Sciences, Paul Scherrer Institute, 5232 Villigen PSI, Switzerland

[*] Corresponding Author. Phone: 404-894-3079; Email: ted.russell@ce.gatech.edu

Summary of Content:

Number of pages	68
Number of texts	1
Number of figures	15
Number of tables	16

(a)

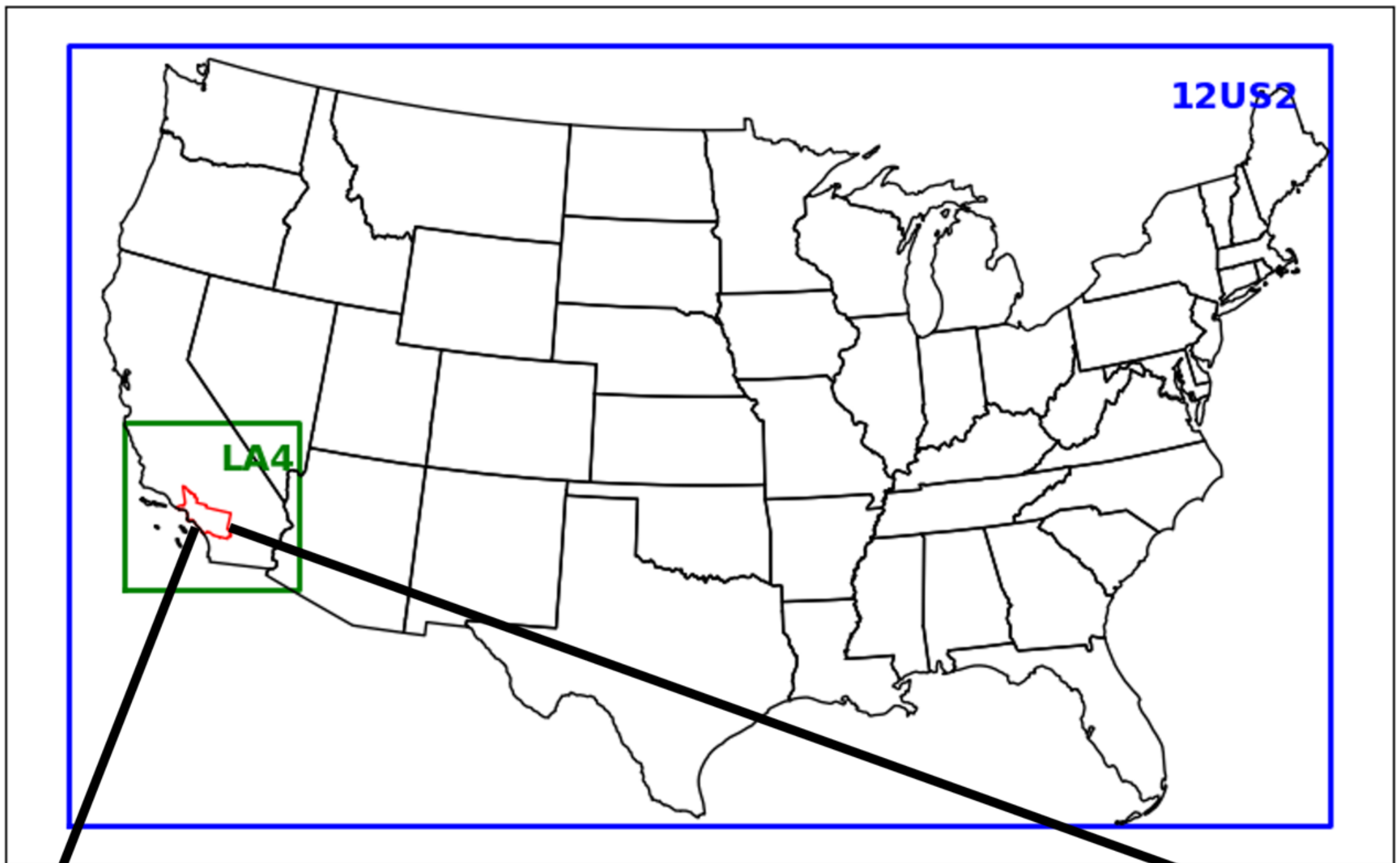

(b)

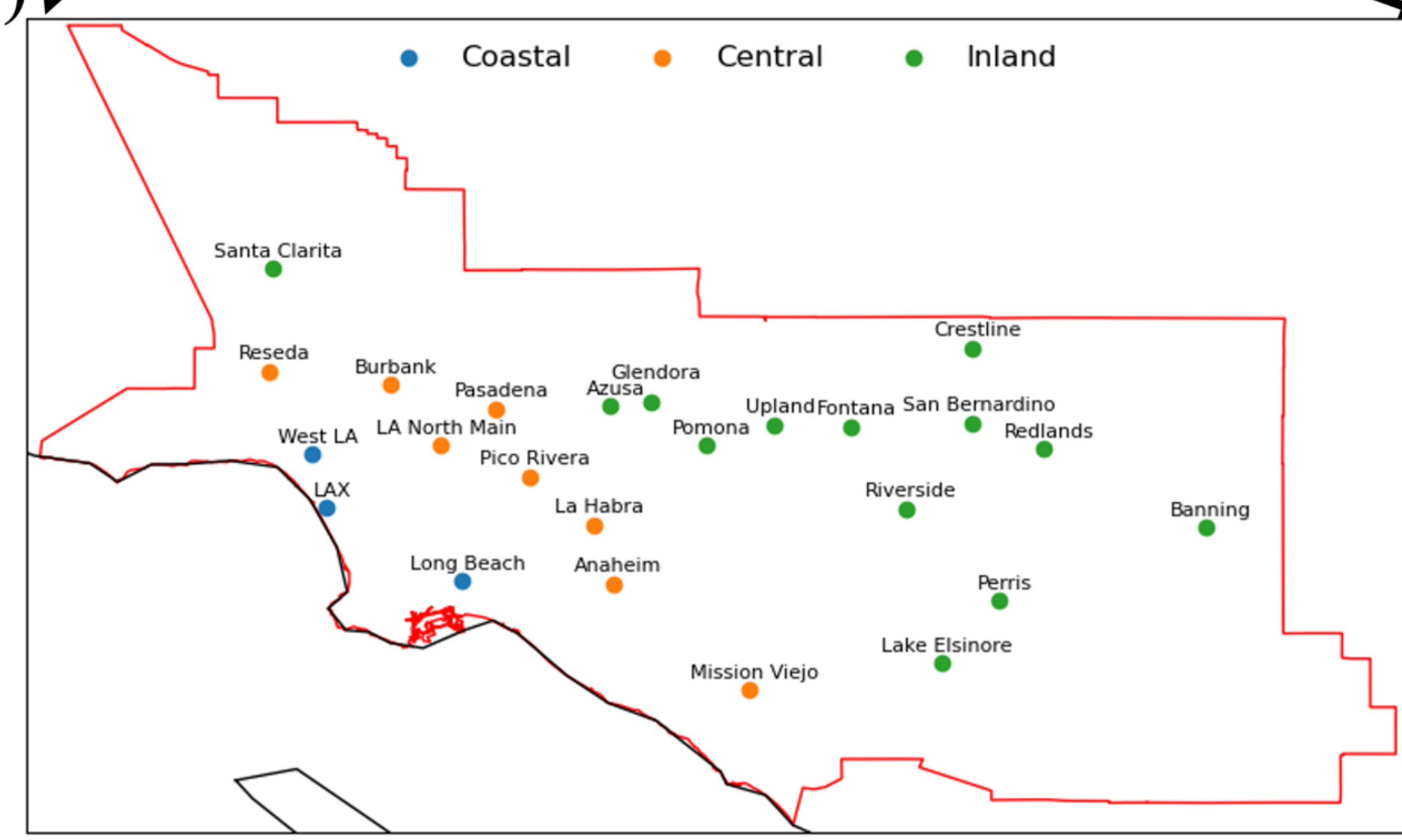

Figure S1. (a) Map of the modeling domain for the US 12-km resolution (12US2: 394 columns by 244 rows) and the southern California 4-km resolution domains (LA4: 165 columns by 156 rows). The red line shows the boundary of the SoCAB region. (b) Locations of the studied monitoring sites within the SoCAB region. Sites are classified into coastal, central, and inland groups based on their distance to the coastline. The red boundary indicates the SoCAB region.

Table S1. Model configurations for CMAQ modeling.

Parameter	Setting
Number of vertical layers	34
Model-top pressure	100 hPa
Gas-phase chemistry	Carbon Bond 6
Aerosol module	AERO6
Dry deposition	M3Dry
Biogenic Emission	Biogenic Emission Inventory System (BEIS)

Table S2. Model configurations for WRF modeling.

Physics	12 km	4 km
Longwave radiation	rrtmg scheme	rrtmg scheme
Shortwave radiation	rrtmg scheme	rrtmg scheme
Land surface	Pleim-Xiu LSM	Pleim-Xiu LSM
Cumulus	Kain-Fritsch scheme	Kain-Fritsch scheme
Microphysics	Morrison (2 moments)	Morrison (2 moments)
PBL	ACM2 (Pleim) PBL	ACM2 (Pleim) PBL
Observational nudging	on	on
Grid nudging	Above the 19th model level	off
Soil nudging	on	off

Table S3. Performance Evaluation Statistics of the WRF Simulated Meteorological Fields against TDL Hourly Observations for the ozone season in 2016.

	WS Bias	WS RMSE	WD Bias	WD GE	TMP Bias	TMP GE	HMD Bias	HMD GE
Benchmark	< ±0.5 m/s	< 2 m/s	< ±10 deg	< 30 deg	< ±0.5 K	< 2 K	< ±1 g/kg	< 2 g/kg
12US2	0.36	1.85	4.78	32.27	0.15	1.50	0.34	1.08
LA4	-0.2	1.43	1.93	39.10	0.05	1.68	0.25	1.01

WS: wind speed; WD: wind direction; TMP: surface temperature; HMD: specific humidity

Bias (MB) = $\frac{1}{N}\sum(M_j - O_j)$

Gross error (GE) = $\frac{1}{N}\sum|M_j - O_j|$

Root mean square error (RMSE) = $\sqrt{\frac{1}{N}\sum(M_j - O_j)^2}$

where N is the number of observation (O) and simulation (M) pairs.

Table S4. Emissions Modeling Platforms and Inventories for 2001, 2011, 2016, and 2028

Year	Modeling Platform	Inventory
2001	2011v6.1	2002ef_11g*
2011	2011v6.3	2011en_11g
2016	2016v7.2 (beta and Regional Haze)	2016ff_16j
2028	2016v1	2028fh_16j

* Used by EPA for Chesapeake Bay Study; combined with 2001 CEM emissions and 2001v2 fire emissions.

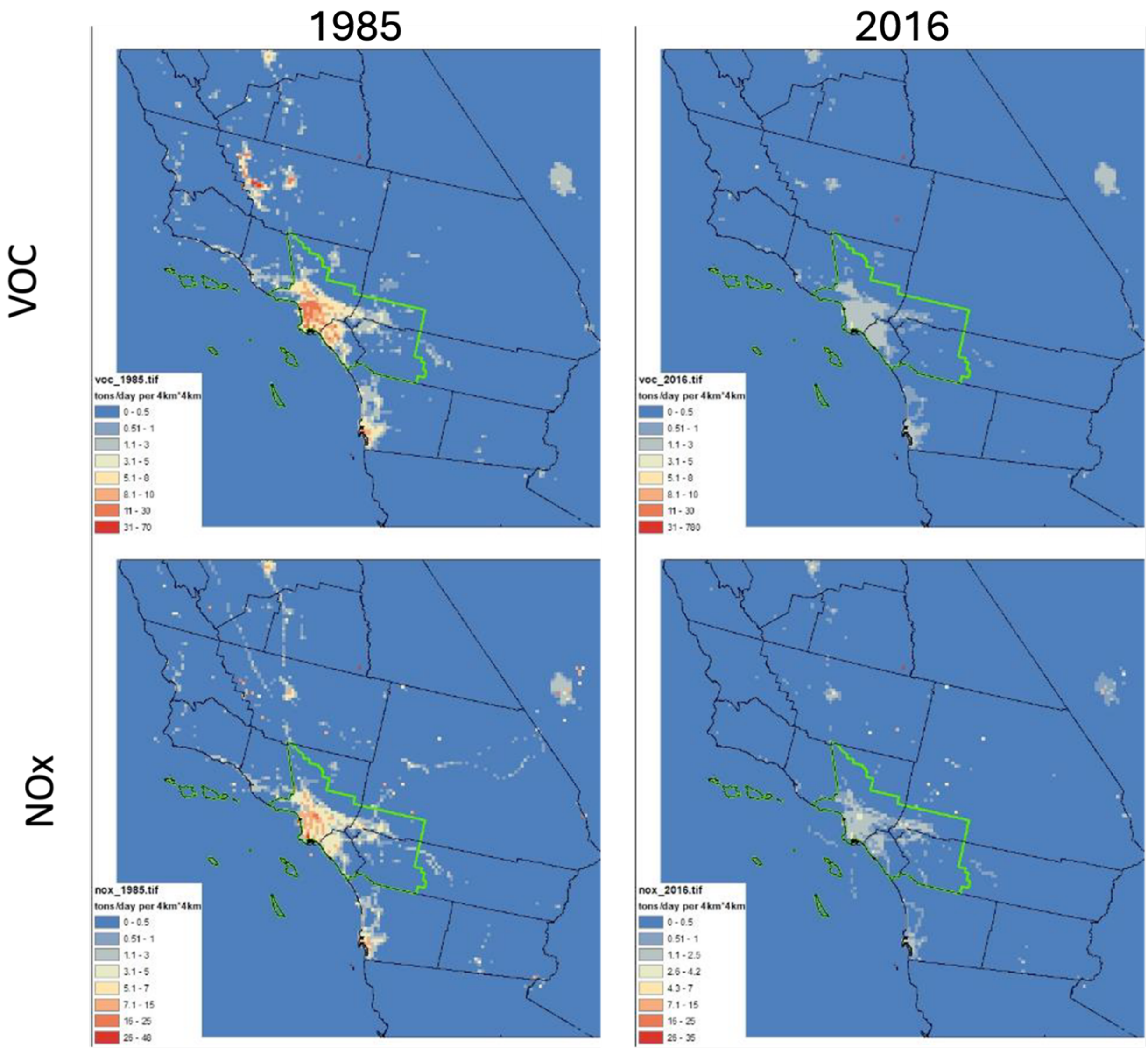

Figure S2. Spatial distribution of VOC and NO_x emissions for 1985 and 2016, respectively. The green line indicates the SoCAB boundary.

Table S5. 1985 county-level total emissions, derived from the 2016 NEI platform and CARB estimates.

1985 Emissions (tons/day)												
County	2016ff						CARB					
	VOC	CO	NO_x	SO_2	PMC	$PM_{2.5}$	VOC	CO	NO_x	SO_2	PMC	$PM_{2.5}$
Fresno	128.1	715.3	146.0	30.3	27.6	15.3	134.9	761.4	162.5	37.4	46.6	28.2
Imperial	25.0	186.2	35.4	3.2	62.7	11.1	32.8	183.2	34.2	3.6	213.9	42.0
Inyo	5.9	42.2	4.6	2.6	117.9	17.6	7.8	53.9	5.9	1.4	697.9	129.7
Kern	789.1	834.3	270.1	31.9	29.7	20.3	637.0	890.8	379.8	65.7	51.2	38.8
Kings	29.1	205.8	27.5	3.6	30.6	17.9	24.2	108.0	26.0	4.2	15.8	6.7
Los Angeles	1536.9	10398.5	1133.8	74.4	39.4	67.9	1518.5	9053.3	1111.5	90.0	112.9	79.2
Monterey	26.0	129.3	25.0	19.3	4.4	5.4	70.2	540.0	85.7	12.0	22.2	10.3
Orange	455.5	2720.5	263.2	11.6	7.7	15.4	431.4	2412.2	270.7	14.8	25.4	16.8
Riverside	164.8	1234.5	169.8	10.0	18.1	11.9	175.6	1096.6	153.3	8.7	32.0	15.9
San Benito	0.8	3.3	0.5	0.0	0.4	0.1	10.2	66.4	12.6	1.4	8.5	3.7
San Bernardino	238.6	1724.7	313.3	20.2	32.2	25.5	232.8	1367.7	289.9	21.7	93.7	52.6
San Diego	428.0	2831.9	293.2	22.1	32.3	19.4	399.7	2893.6	268.6	21.0	59.3	24.3
San Luis Obispo	43.4	308.4	41.4	12.1	5.8	3.3	49.6	305.4	51.7	18.8	17.4	6.8
Santa Barbara	62.3	352.9	47.9	6.1	6.3	3.4	76.0	485.1	77.5	16.1	11.6	6.5
Tulare	61.5	391.6	69.8	5.3	33.2	10.9	59.6	357.0	56.9	5.3	18.2	10.3
Ventura	106.9	652.9	92.9	3.1	5.3	4.3	105.2	631.4	97.3	7.0	11.6	7.5
Sum	4101.9	22732.3	2934.4	255.8	453.6	249.7	3965.5	21206.0	3084.1	329.2	1438.2	479.3

Table S6. 2001 county-level total emissions, from the NEI emission platform and CARB estimates.

2001 Emissions (tons/day)												
County	2002ef						CARB					
	VOC	CO	NO_x	SO_2	PMC	$PM_{2.5}$	VOC	CO	NO_x	SO_2	PMC	$PM_{2.5}$
**Fresno**	136.9	379.5	154.1	13.1	27.7	10.3	99.4	380.1	147.9	3.3	62.6	26.0
**Imperial**	78.6	126.5	52.9	6.0	38.8	8.0	23.4	100.4	38.2	1.2	274.4	44.8
**Inyo**	3.4	11.9	4.5	0.5	3.9	0.8	5.6	25.1	5.4	0.7	202.5	37.3
**Kern**	151.2	442.0	299.5	9.8	33.5	15.1	120.3	351.7	200.2	9.7	48.3	24.7
**Kings**	42.2	73.4	44.8	1.5	12.6	3.3	29	68.7	31.7	0.5	25.3	8
**Los Angeles**	607.1	3466.5	930.4	71.6	78.2	41.2	577.6	3152.5	716.2	60.9	52.9	55.7
**Monterey**	19.3	75.3	26.1	1.3	7.4	1.9	41.5	190.9	63.2	8.6	18.4	8.1
**Orange**	194.4	985.9	238.6	17.4	27.1	11.7	175.2	901.0	171.6	5.7	12.9	12.7
**Riverside**	148.7	718.6	262.7	9.7	42.8	10.7	102.4	544.6	189.6	2.6	32.9	14.0
**San Benito**	2.0	4.3	3.0	0.1	0.4	0.1	5.7	24.6	10.8	0.1	7.0	1.6
**San Bernardino**	197.8	1017.1	408.3	11.4	63.1	22.5	142.2	767.1	347.0	11.7	58.9	33.9
**San Diego**	236.4	1157.0	279.2	10.6	58.7	19.3	202.5	1023.8	227.7	6.4	55.2	26.9
**San Luis Obispo**	39.5	172.7	50.2	14.5	12.9	7.0	27.2	143.5	39.6	16.8	8.1	5.7
**Santa Barbara**	55.1	226.0	96.5	24.9	9.5	6.3	39.4	169.3	81.9	32.0	9.7	7.6
**Tulare**	124.0	203.6	78.1	3.2	16.7	7.4	69.2	182	61.8	0.9	33.1	10.8
**Ventura**	73.2	320.2	108.5	14.1	12.0	7.9	57.2	277.2	66.9	9.1	10.5	7.6
**Sum**	2109.8	9380.5	3037.4	209.7	445.3	173.5	1717.8	8302.5	2399.7	170.2	912.7	325.4

Table S7. 2011 county-level total emissions, from the NEI emission platform and CARB estimates.

County	2011 Emissions (tons/day)											
	2011ek						CARB					
	VOC	CO	NO_x	SO_2	PMC	$PM_{2.5}$	VOC	CO	NO_x	SO_2	PMC	$PM_{2.5}$
**Fresno**	53.4	168.4	85.8	1.5	20.2	9.0	66.7	176.2	91.1	1.6	48.9	16.8
**Imperial**	14.4	61.8	27.0	0.2	46.5	8.2	16.6	66.3	23.4	0.5	250.2	40.5
**Inyo**	2.0	6.5	1.5	0.2	3.3	0.9	3.6	12.3	3.2	0.5	28.5	5.0
**Kern**	85.1	185.7	118.9	6.4	25.9	14.2	86.2	189.4	120.8	6.2	35.0	18.8
**Kings**	10.3	31.2	19.0	0.2	10.0	2.5	26.7	37.6	20.1	0.3	19.4	5.4
**Los Angeles**	340.0	1602.7	369.1	12.7	45.2	41.1	309.2	1461.1	376.4	16.8	54.0	44.4
**Monterey**	7.3	23.0	9.4	0.2	4.7	1.5	31.2	101.4	46.4	2.6	19.4	6.7
**Orange**	108.3	476.3	84.1	1	10.5	10.3	97.3	446.7	87.1	1.8	12.4	10.8
**Riverside**	76.6	347.9	120.9	0.8	28.4	11.7	69.3	296.2	107.2	1	36.3	13.8
**San Benito**	0.5	1.7	1.1	0.0	0.3	0.1	4.2	13.4	7.2	0.0	6.0	1.4
**San Bernardino**	101.3	467.9	208.0	4.5	44.0	26.0	91.4	388.7	190.9	5.4	76.3	30.2
**San Diego**	131.9	505.7	118.8	1.3	42.1	15.9	134.8	507.0	135.2	1.9	49.3	19.7
**San Luis Obispo**	17.2	67.4	17.3	0.5	7.6	3.4	19.3	70.9	28.6	6.6	7.4	4.2
**Santa Barbara**	25.7	75.9	21.5	1.0	8.2	3.3	31.8	101.9	73.0	11.9	8.9	5.2
**Tulare**	28.4	103.9	42.2	0.4	14.1	5.3	60.2	98.7	39.8	0.5	27.5	8.3
**Ventura**	37.9	147.7	29.2	0.7	8.0	5.3	37.2	137.2	42.7	3.0	10.6	6.0
**Sum**	1040.3	4273.7	1273.8	31.6	319.0	158.7	1085.7	4105.0	1393.1	60.6	690.1	237.2

Table S8. 2016 county-level total emissions, from the NEI emission platform and CARB estimates.

2016 Emissions (tons/day)												
County	2016ff						CARB					
	VOC	CO	NO_x	SO_2	PMC	$PM_{2.5}$	VOC	CO	NO_x	SO_2	PMC	$PM_{2.5}$
**Fresno**	41.7	127.1	54.9	0.8	30.8	10.1	55.5	117.1	59.4	1.6	49.7	14.1
**Imperial**	12.6	48.6	16.4	0.1	57.0	10.3	15.3	54.8	17.1	0.3	245.2	39.0
**Inyo**	2.2	6.7	1.6	0.5	5.4	1.1	3.4	8.6	2.2	0.5	28.6	4.9
**Kern**	61.6	138.5	76.2	4.9	26.4	13.6	71.2	143.6	86.1	9.6	35.4	18.0
**Kings**	8.9	26.7	12	0.1	23.6	6.0	21.1	27.9	13.1	0.2	18.4	4.8
**Los Angeles**	267.5	1210.2	236.3	11.5	46.6	40.5	249.0	1035.3	269.2	14.5	57.7	41.5
**Monterey**	10.7	21.1	6.2	0.2	5.9	1.7	29.3	84.2	44.6	1.3	21.2	7.0
**Orange**	110.1	445.9	63.4	1.5	11.6	13.8	80.9	338.4	65.1	1.3	13.3	10.3
**Riverside**	60.8	303.5	85.5	1.2	32.7	13.9	60.9	223.2	74.3	0.9	42.1	13.1
**San Benito**	0.3	0.7	0.2	0.0	0.4	0.1	3.7	9.5	4.3	0.0	6.2	1.3
**San Bernardino**	72.5	369.9	145.5	2.9	42.9	23.3	74.4	282.5	153.0	4.4	81.4	30.8
**San Diego**	109.0	460.5	84.3	1.9	43.5	19.1	113.3	389.2	99.0	1.3	56.8	19.4
**San Luis Obispo**	12.6	53.4	12.2	0.6	6.2	2.6	17.9	56.7	28.4	1.1	7.7	3.5
**Santa Barbara**	19.8	58.4	14.3	1.0	7.2	2.6	28.5	81.3	69.0	2.2	9.8	3.7
**Tulare**	20.4	74.8	28.0	0.4	38.2	10.4	46.2	61.7	26.2	0.4	27.5	6.7
**Ventura**	28.5	115.4	20.9	0.7	8.3	4.0	32.6	107.0	36.5	1.5	11.5	5.9
**Sum**	839.2	3461.4	857.9	28.3	386.7	173.1	903.2	3021.0	1047.5	41.1	712.5	224.0

Table S9. 2028 county-level total emissions, from the NEI emission platform and CARB estimates.

2028 Emissions (tons/day)												
County	2028fh						CARB					
	VOC	CO	NO_x	SO_2	PMC	$PM_{2.5}$	VOC	CO	NO_x	SO_2	PMC	$PM_{2.5}$
**Fresno**	35.6	95.4	26.3	0.8	31.5	9.5	53.9	84.2	30.8	1.8	51.4	14.1
**Imperial**	9.3	29.4	9.0	0.1	57.2	10.1	14.5	53.0	12.1	0.2	245.9	39.6
**Inyo**	1.5	4.3	1.0	0.4	5.0	1.1	3.2	5.3	1.1	0.5	28.8	5.0
**Kern**	51.4	120.6	51.8	4.3	26.9	12.4	68.5	117.1	57.4	10.9	36.0	17.9
**Kings**	7.6	16.9	6.7	0.1	23.4	5.4	21.4	30.6	9.2	0.2	14.1	4.5
**Los Angeles**	218.5	935.8	147.2	13.4	47.4	39.8	214.0	724.9	166.7	14	64.3	42.5
**Monterey**	8.9	16.2	3.8	0.2	6.0	1.8	29.1	68.0	46.6	1.5	25.1	7.7
**Orange**	99.9	382.8	32.0	1.3	12.3	13.6	70.1	267.7	37.6	1.3	14.4	10.3
**Riverside**	51.0	256.5	45.8	1.1	34.8	13.9	60.4	172.1	33.1	0.9	54.2	14.9
**San Benito**	0.2	0.7	0.1	0.0	0.5	0.1	3.8	7.0	2.2	0.0	6.6	1.4
**San Bermardino**	50.9	277.1	91.5	2.7	42.0	21.8	66.1	209.6	112.4	5.4	95.2	35.6
**San Diego**	95.1	376.3	42.9	1.9	44.5	18.8	102.7	304.7	71.7	1.4	68.2	20.6
**San Luis Obispo**	10.5	42.0	6.2	0.6	6.3	2.5	17.1	45.8	30.5	1.3	8.1	3.6
**Santa Barbara**	16.4	43.7	8.3	0.9	7.4	2.4	29.0	68.2	86.9	2.9	11.6	4.1
**Tulare**	16.3	56.3	14.9	0.4	38.4	10.2	44.2	44.7	13.0	0.4	28.4	6.6
**Ventura**	22.6	83.0	11.3	0.4	8.6	3.9	29.8	82.4	34.2	1.9	12.7	6.3
**Sum**	695.7	2737.0	498.8	28.6	392.2	167.3	827.8	2285.3	745.5	44.6	765.0	234.7

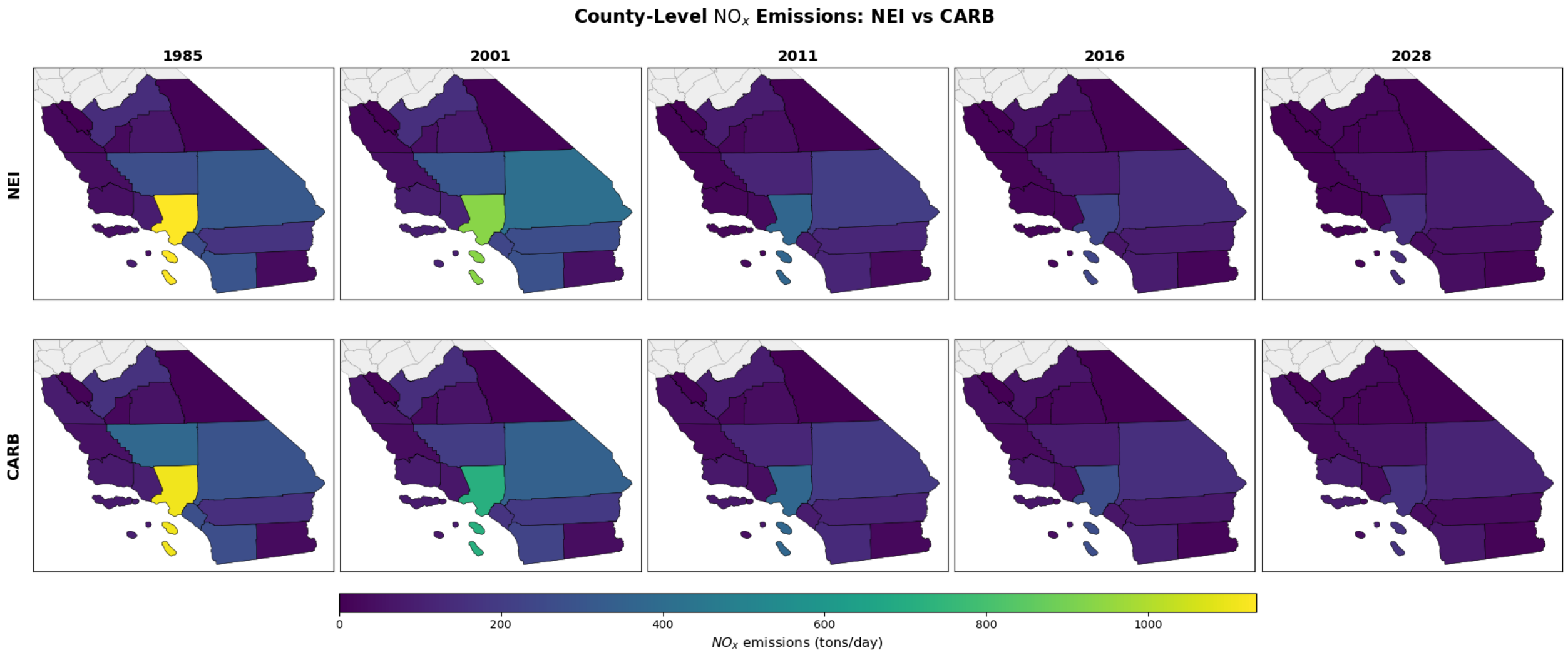

Figure S3. County-level anthropogenic NO_x emissions from NEI (first row) and CARB (second row) emission inventories for 1985, 2001, 2011, 2016, and 2028 (unit: tons/day).

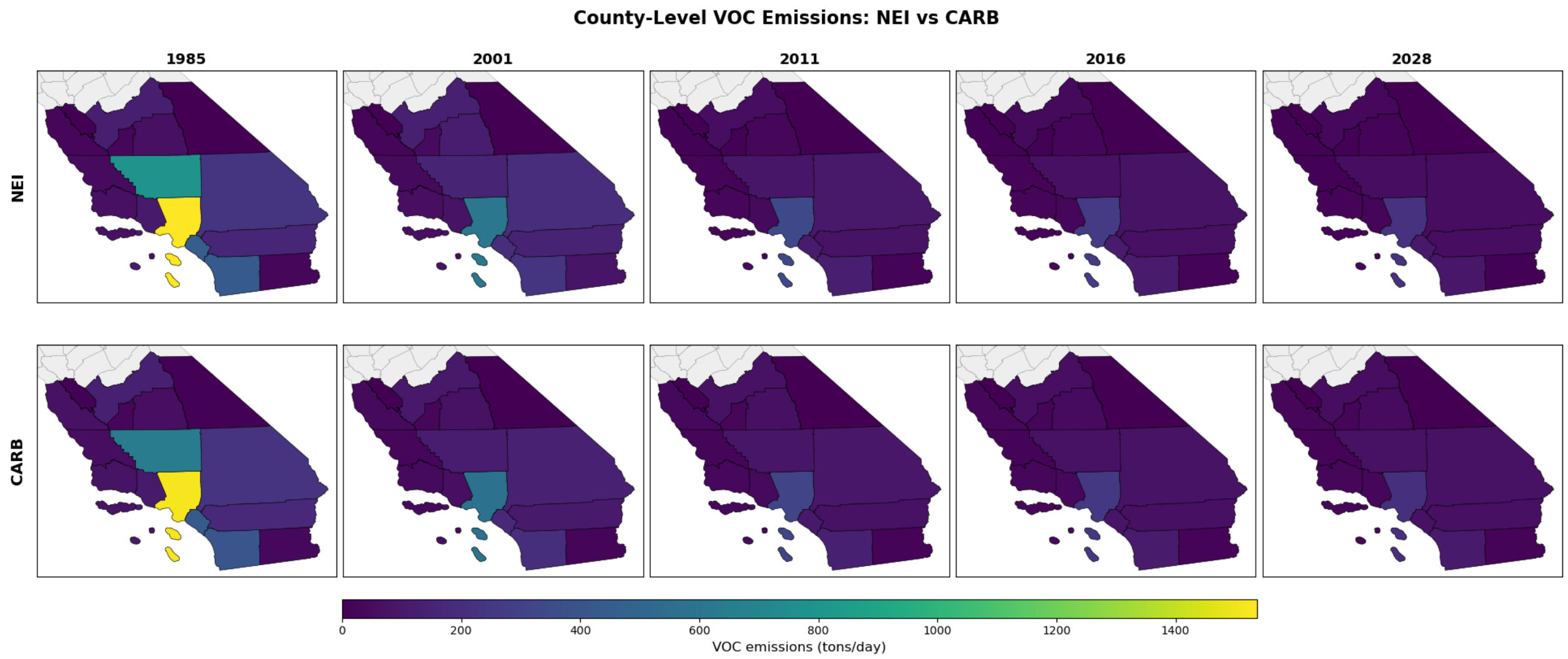

Figure S4. County-level anthropogenic VOC emissions from NEI (first row) and CARB (second row) emission inventories for 1985, 2001, 2011, 2016, and 2028 (unit: tons/day).

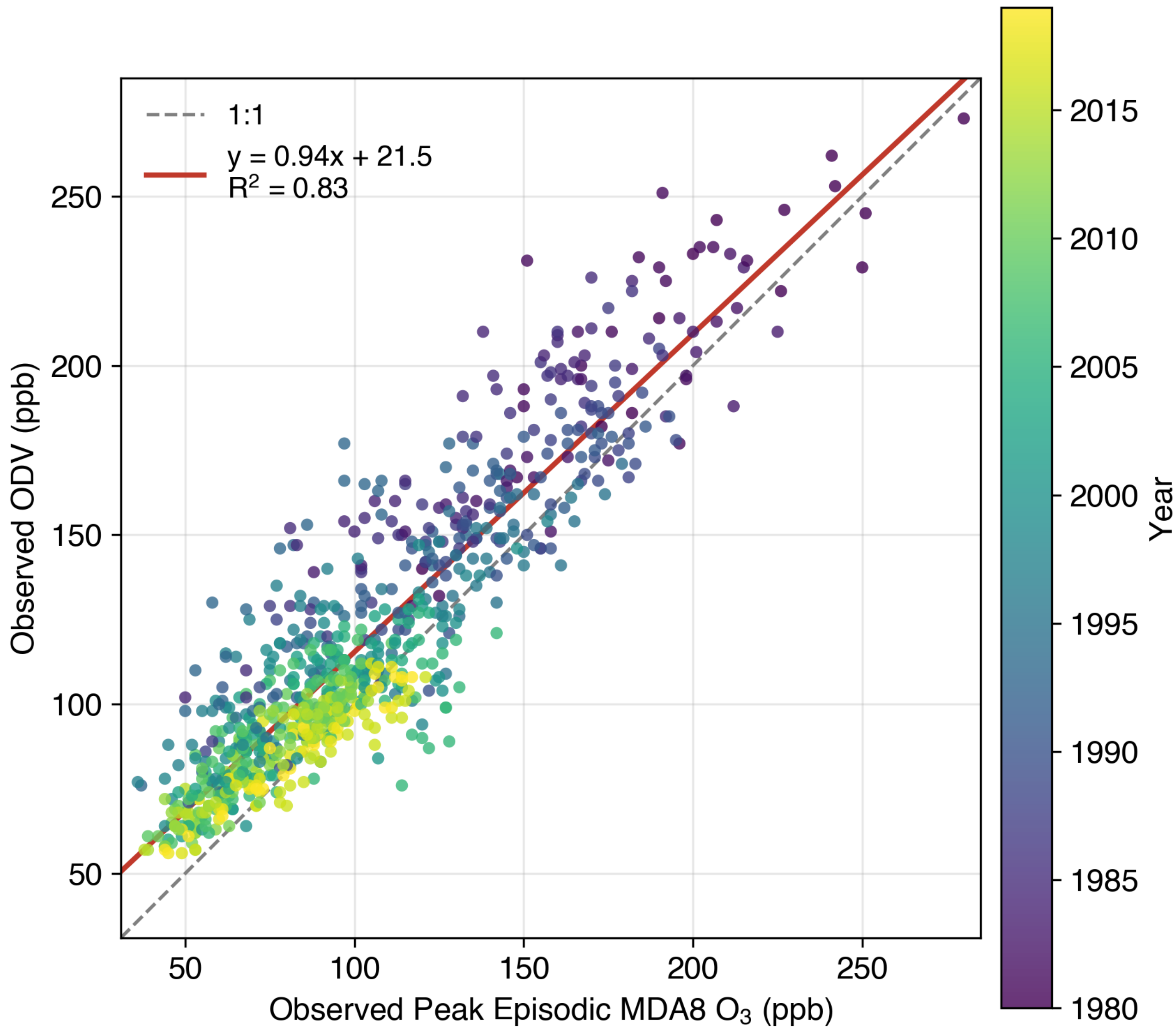

Figure S5. Observed ODV versus the observed peak MDA8 ozone from the 13-day high-ozone period (from June 2 to June 4 and from July 20 to July 29), across all SoCAB sites and years from 1980 to 2019. Points colored by year; red line is the linear regression line; black dashed line is 1:1.

Table S10. Definition of CMAQ-HDDM emission scenarios relative to 2016 baseline emissions. The NO_x and anthropogenic VOC emission levels for the 2016 baseline scenario are 396 and 401 tons/day. VOC emission changes refer only to anthropogenic VOCs, while biogenic VOCs remain unchanged across all scenarios.

Scenario	NO_x emissions (% of 2016)	VOC emissions (% of 2016)
1 percent	1%	1%
base	100%	100%
1985	390%	490%
n10v100	10%	100%
n100v10	100%	10%
n200v10	200%	10%
n10v400	10%	400%
n300v10	300%	10%
n110v110	110%	110%
n90v90	90%	90%
n110v100	110%	100%
n90v100	90%	100%
2001	230%	360%
2011	130%	150%
2028	80%	60%

Text S1. Detailed mathematical derivations of least-squares methods

Quadratic Model:

For the *i^th^* CMAQ-HDDM simulation, the ozone response function is expressed as:

$$O_3\left(E_{NO_{x_i}}, E_{VOC_i}\right)_i = \beta + \alpha_{NO_x}E_{NO_{x_i}} + \alpha_{VOC}E_{VOC_i} + \alpha_{NO_x-VOC}E_{NO_{x_i}}E_{VOC_i} + \alpha_{NO_x{}^2}E_{NO_{x_i}}{}^2 + \alpha_{VOC^2}E_{VOC_i}{}^2 \quad (S1)$$

Taking the first- and second-order partial derivatives at the emission levels of simulation *i* yields:

$$\begin{cases} \left(\dfrac{\partial O_3}{\partial E_{NO_x}}\right)_i = \alpha_{NO_x} + \alpha_{NO_x-VOC}E_{VOC_i} + 2\alpha_{NO_x{}^2}E_{NO_{x_i}} \\ \left(\dfrac{\partial O_3}{\partial E_{VOC}}\right)_i = \alpha_{VOC} + \alpha_{NO_x-VOC}E_{NO_{x_i}} + 2\alpha_{VOC^2}E_{VOC_i} \\ \left(\dfrac{\partial^2 O_3}{\partial E_{NO_x}\,\partial E_{VOC}}\right)_i = \alpha_{NO_x-VOC} \\ \left(\dfrac{\partial^2 O_3}{\partial E_{NO_x}{}^2}\right)_i = 2\alpha_{NO_x{}^2} \\ \left(\dfrac{\partial^2 O_3}{\partial E_{VOC}{}^2}\right)_i = 2\alpha_{VOC^2} \end{cases} \quad (S2)$$

The left-hand side of Equations S1 and S2 is obtained directly from CMAQ-HDDM simulation Sim_i $(i = 1, 2, \dots, 15)$, while the right-hand side contains the six coefficients $(\alpha(\cdot),\ \beta)$ to be derived. By combining Equation S1 and Equation S2, the six equations can be written jointly in matrix form as:

$$\underbrace{\begin{pmatrix} O_3 \\ \dfrac{\partial O_3}{\partial E_{NO_x}} \\ \dfrac{\partial O_3}{\partial E_{VOC}} \\ \dfrac{\partial^2 O_3}{\partial E_{NO_x}\,\partial E_{VOC}} \\ \dfrac{\partial^2 O_3}{\partial E_{NO_x}{}^2} \\ \dfrac{\partial^2 O_3}{\partial E_{VOC}{}^2} \end{pmatrix}_i}_{b_i} = \underbrace{\begin{pmatrix} 1 & E_{NO_x} & E_{VOC} & E_{NO_x}E_{VOC} & E_{NO_x}{}^2 & E_{VOC}{}^2 \\ 0 & 1 & 0 & E_{VOC} & 2E_{NO_x} & 0 \\ 0 & 0 & 1 & E_{NO_x} & 0 & E_{VOC} \\ 0 & 0 & 0 & 1 & 0 & 0 \\ 0 & 0 & 0 & 0 & 2 & 0 \\ 0 & 0 & 0 & 0 & 0 & 2 \end{pmatrix}_i}_{A_i} \underbrace{\begin{pmatrix} \beta \\ \alpha_{NO_x} \\ \alpha_{VOC} \\ \alpha_{NO_x-VOC} \\ \alpha_{NO_x{}^2} \\ \alpha_{VOC^2} \end{pmatrix}}_{\alpha} \quad (S3)$$

To jointly estimate the coefficients across all 15 simulations, we stack the individual systems from Equation S3 into a single overdetermined linear system:

$$\begin{pmatrix} \boldsymbol{b_1} \\ \boldsymbol{b_2} \\ \vdots \\ \boldsymbol{b_{15}} \end{pmatrix} = \begin{pmatrix} \boldsymbol{A_1} \\ \boldsymbol{A_2} \\ \vdots \\ \boldsymbol{A_{15}} \end{pmatrix} \boldsymbol{\alpha} \quad (S4)$$

where each block $\boldsymbol{A_i}$ uses the emission levels ($\mathrm{E_{VOC_i}}, \mathrm{E_{NO_{x_i}}}$) specific to simulation i. The resulting system has a 90 × 1 response vector on the left-hand side and a 90 × 6 design matrix multiplied by the 6 × 1 coefficient vector on the right-hand side. With 90 equations and 6 unknowns, the system is overdetermined and the coefficient vector α is estimated by ordinary least squares.

Log-quadratic Model:

The log-quadratic model follows the same framework as the quadratic model, with log(O_3) replacing O_3 as the response variable:

$$\log(O_3(\mathrm{E_{NO_{x_i}}}, \mathrm{E_{VOC_i}}))_i = \beta + \alpha_{\mathrm{NO_x}}\mathrm{E_{NO_{x_i}}} + \alpha_{\mathrm{VOC}}\mathrm{E_{VOC_i}} + \alpha_{\mathrm{NO_x-VOC}}\mathrm{E_{NO_{x_i}}}\mathrm{E_{VOC_i}} + \alpha_{\mathrm{NO_x{}^2}}\mathrm{E_{NO_{x_i}}}^2 + \alpha_{\mathrm{VOC^2}}\mathrm{E_{VOC_i}}^2 \quad (S5)$$

Since CMAQ-HDDM provides sensitivities of O_3 rather than $\log O_3$, the chain rule is applied to convert them.

$$\begin{cases} \left(\dfrac{\partial \log \mathrm{O_3}}{\partial \mathrm{E_{NO_x}}}\right)_i = \dfrac{1}{O_{3_i}}\left(\dfrac{\partial O_3}{\partial \mathrm{E_{NO_x}}}\right)_i \\ \left(\dfrac{\partial \log \mathrm{O_3}}{\partial \mathrm{E_{VOC}}}\right)_i = \dfrac{1}{O_{3_i}}\left(\dfrac{\partial O_3}{\partial \mathrm{E_{VOC}}}\right)_i \\ \left(\dfrac{\partial^2 \log \mathrm{O_3}}{\partial \mathrm{E_{NO_x}}\,\partial \mathrm{E_{VOC}}}\right)_i = \dfrac{1}{O_{3_i}}\left(\dfrac{\partial^2 O_3}{\partial \mathrm{E_{NO_x}}\,\partial \mathrm{E_{VOC}}}\right)_i - \dfrac{1}{O_{3_i}^2}\left(\dfrac{\partial O_3}{\partial \mathrm{E_{VOC}}}\right)_i\left(\dfrac{\partial O_3}{\partial \mathrm{E_{NO_x}}}\right)_i \\ \left(\dfrac{\partial^2 \log \mathrm{O_3}}{\partial \mathrm{E_{NO_x}}^2}\right)_i = \dfrac{1}{O_{3_i}}\left(\dfrac{\partial^2 O_3}{\partial \mathrm{E_{NO_x}}^2}\right)_i - \dfrac{1}{O_{3_i}^2}\left(\dfrac{\partial O_3}{\partial \mathrm{E_{NO_x}}}\right)_i^2 \\ \left(\dfrac{\partial^2 \log \mathrm{O_3}}{\partial \mathrm{E_{VOC}}^2}\right)_i = \dfrac{1}{O_{3_i}}\left(\dfrac{\partial^2 O_3}{\partial \mathrm{E_{VOC}}^2}\right)_i - \dfrac{1}{O_{3_i}^2}\left(\dfrac{\partial O_3}{\partial \mathrm{E_{VOC}}}\right)_i^2 \end{cases} \quad (S6)$$

The transformed sensitivities in Equation S6 then replace the corresponding entries in the response vector $\mathbf{b}_i$ of Equation S3, while the design matrix $\mathbf{A}_i$ and coefficient vector $\boldsymbol{\alpha}$ remain unchanged. The coefficients are estimated by ordinary least squares following the same procedure as Equation S4.

Diagnostic Sensitivity Lines:

For the quadratic model, the zero-NO_x-sensitivity line (white dashed) is defined by $\partial O_3/\partial E_{\mathrm{NO}_x} = 0$. Using Equation S1, we have:

$$\alpha_{\mathrm{NO}_x} + \alpha_{\mathrm{NO}_x\text{–VOC}}\, E_{\mathrm{VOC}} + 2\alpha_{\mathrm{NO}_x{}^2}\, E_{\mathrm{NO}_x} = 0 \quad (S7)$$

which gives

$$E_{\mathrm{NO}_x} = -\frac{\alpha_{\mathrm{NO}_x} + \alpha_{\mathrm{NO}_x\text{–VOC}}\, E_{\mathrm{VOC}}}{2\alpha_{\mathrm{NO}_x{}^2}} \quad (S8)$$

The equal-sensitivity (ridge) line is defined by $\partial O_3/\partial E_{\mathrm{NO}_x} = \partial O_3/\partial E_{\mathrm{VOC}}$. Using Equation S1, we have:

$$\alpha_{\mathrm{NO}_x} + \alpha_{\mathrm{NO}_x\text{–VOC}}\, E_{\mathrm{VOC}} + 2\alpha_{\mathrm{NO}_x{}^2}\, E_{\mathrm{NO}_x} = \alpha_{\mathrm{VOC}} + \alpha_{\mathrm{NO}_x\text{–VOC}}\, E_{\mathrm{NO}_x} + 2\alpha_{\mathrm{VOC}^2}\, E_{\mathrm{VOC}} \quad (S9)$$

which, solved for E_{NO_x}, gives

$$E_{\mathrm{NO}_x} = \frac{(2\alpha_{\mathrm{VOC}^2} - \alpha_{\mathrm{NO}_x\text{–}\mathrm{VOC}})\,E_{\mathrm{VOC}} + (\alpha_{\mathrm{VOC}} - \alpha_{\mathrm{NO}_x})}{2\alpha_{\mathrm{NO}_x{}^2} - \alpha_{\mathrm{NO}_x\text{–}\mathrm{VOC}}} \quad (S10)$$

Similarly, for the log-quadratic model, using Equation S5, we have:

$$\frac{\partial O_3}{\partial E_{\mathrm{NO}_x}} = O_3\left(\alpha_{\mathrm{NO}_x} + \alpha_{\mathrm{NO}_x\text{–}\mathrm{VOC}} E_{\mathrm{VOC}} + 2\alpha_{\mathrm{NO}_x{}^2} E_{\mathrm{NO}_x}\right) \quad (S11)$$

$$\frac{\partial O_3}{\partial E_{\mathrm{VOC}}} = O_3\left(\alpha_{\mathrm{VOC}} + \alpha_{\mathrm{NO}_x\text{–}\mathrm{VOC}} E_{\mathrm{NO}_x} + 2\alpha_{\mathrm{VOC}^2} E_{\mathrm{VOC}}\right) \quad (S12)$$

Because $O_3 > 0$, this strictly positive factor cancels in both $\partial O_3/\partial E_{\mathrm{NO}_x} = 0$ and $\partial O_3/\partial E_{\mathrm{NO}_x} = \partial O_3/\partial E_{\mathrm{VOC}}$. Consequently, the zero-NO_x-sensitivity and ridge lines take exactly the same algebraic form as in the quadratic model (Equations S8 and S10), with the quadratic coefficients replaced by the log-quadratic ones.

Both diagnostic lines can be written in the generic form $\mathrm{E}_{\mathrm{NO_x}} = \mathrm{a}\,\mathrm{E}_{\mathrm{VOC}} + \mathrm{b}$. For any emission point $(\mathrm{E}_{\mathrm{NO_x}}, \mathrm{E}_{\mathrm{VOC}})$ we form the signed quantity:

$$\Delta \equiv \mathrm{E}_{\mathrm{NO_x}} - (\mathrm{a}\,\mathrm{E}_{\mathrm{VOC}} + \mathrm{b}) \quad (S13)$$

so that $\Delta < 0$ places the point below the line (its $\mathrm{NO_x}$ emission lies beneath the line at the same VOC level), $\Delta > 0$ places it above (over) the line, and $\Delta = 0$ places it on the line.

Emission Normalization:

In this study, the linear systems in Equations S3 and S4 were assembled and solved with NO_x and VOC emissions normalized by corresponding emissions in the 2016 base scenario ($E_{NOx,base}$ = 395.7 and $E_{VOC,base}$ = 401.4 tons day^{-1}):

$$e_{NO_x} = \frac{E_{NO_x}}{E_{NO_x,base}} \quad (S14)$$

$$e_{VOC} = \frac{E_{VOC}}{E_{VOC,base}} \quad (S15)$$

In Equation S3, $\boldsymbol{b_i}$ and α_i are in units of ppb. A_i is dimensionless. Ozone concentration and the first- and second-order sensitivity equations enter the least-squares objective at comparable magnitudes with normalization; without normalization, the much smaller sensitivity terms would be effectively unconstrained, and the design matrix would be poorly conditioned. The normalization of emissions increases the weights of first- and second-order sensitivities and can be explained by the chain rule. Assuming $O_3\left(E_{NO_x}, E_{VOC}\right) = O_3\left(E_{NO_x}/E_{NO_x,base}, E_{VOC}/E_{VOC,base}\right) = O_3(e_{NO_x}, e_{VOC})$, the derivatives can be calculated using chain rule:

$$\frac{\partial O_3}{\partial E_{\mathrm{NO}_x}} = \frac{1}{E_{\mathrm{NO}_x,\mathrm{base}}} \frac{\partial O_3}{\partial e_{\mathrm{NO}_x}} \quad (S16)$$

$$\frac{\partial O_3}{\partial E_{\mathrm{VOC}}} = \frac{1}{E_{\mathrm{VOC,base}}} \frac{\partial O_3}{\partial e_{\mathrm{VOC}}} \quad (S17)$$

$$\frac{\partial^2 O_3}{\partial E_{\mathrm{NO}_x}^2} = \frac{1}{E_{\mathrm{NO}_x,\mathrm{base}}^2} \frac{\partial^2 O_3}{\partial e_{\mathrm{NO}_x}^2} \quad (S18)$$

$$\frac{\partial^2 O_3}{\partial E_{\mathrm{VOC}}^2} = \frac{1}{E_{\mathrm{VOC,base}}^2} \frac{\partial^2 O_3}{\partial e_{\mathrm{VOC}}^2} \quad (S19)$$

$$\frac{\partial^2 O_3}{\partial E_{\mathrm{NO}_x}\,\partial E_{\mathrm{VOC}}} = \frac{1}{E_{\mathrm{NO}_x,\mathrm{base}}\,E_{\mathrm{VOC,base}}}\frac{\partial^2 O_3}{\partial e_{\mathrm{NO}_x}\,\partial e_{\mathrm{VOC}}} \qquad (S20)$$

For example, for NO_x, using $\frac{\partial O_3}{\partial e_{\mathrm{NO}_x}}$ or $\frac{\partial^2 O_3}{\partial e_{\mathrm{NO}_x}^2}$ as the fitting target multiplies each first-order sensitivity by $E_{NOx,base}$ or each second-order sensitivity by $E_{NOx,base}^2$.

Solving Equation S4 in the normalized space yields normalized-space coefficients, which are projected back to the physical ozone response function by substituting Equations S14 and S15 in Equation S1 (quadratic model) or Equation S5 (log-quadratic model). The sensitivities are projected back to physical units (ppb/(ton·day^{-1}) and ppb/(ton·day^{-1})^2 for first- and second-order derivatives) using Equations S16 to S20.

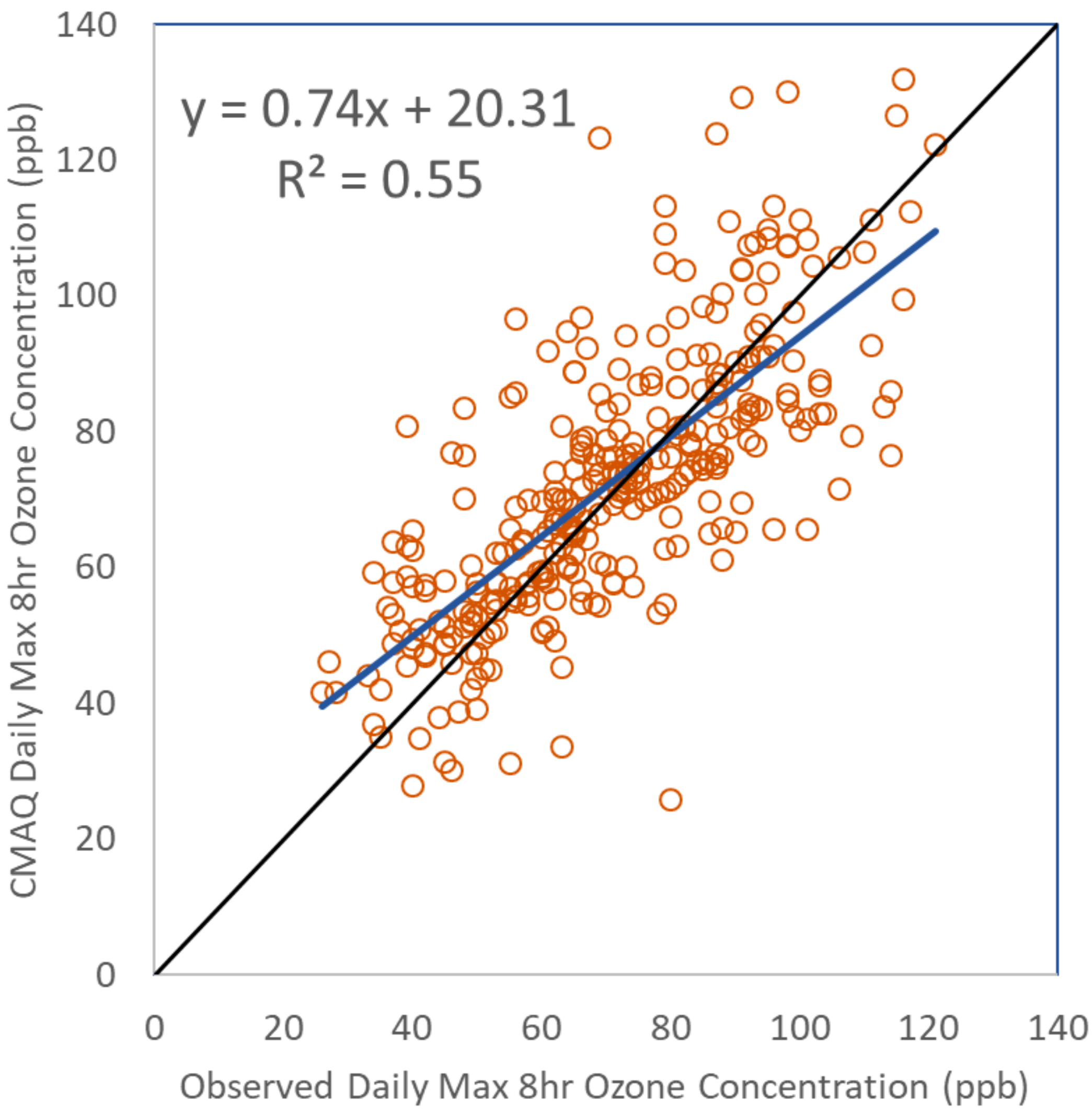

Figure S6. Comparison between CMAQ-simulated and observed MDA8 ozone concentrations for all 24 monitoring sites over two high-ozone periods, from June 2 to June 4 and from July 20 to July 29 in 2016, totaling 13 days.

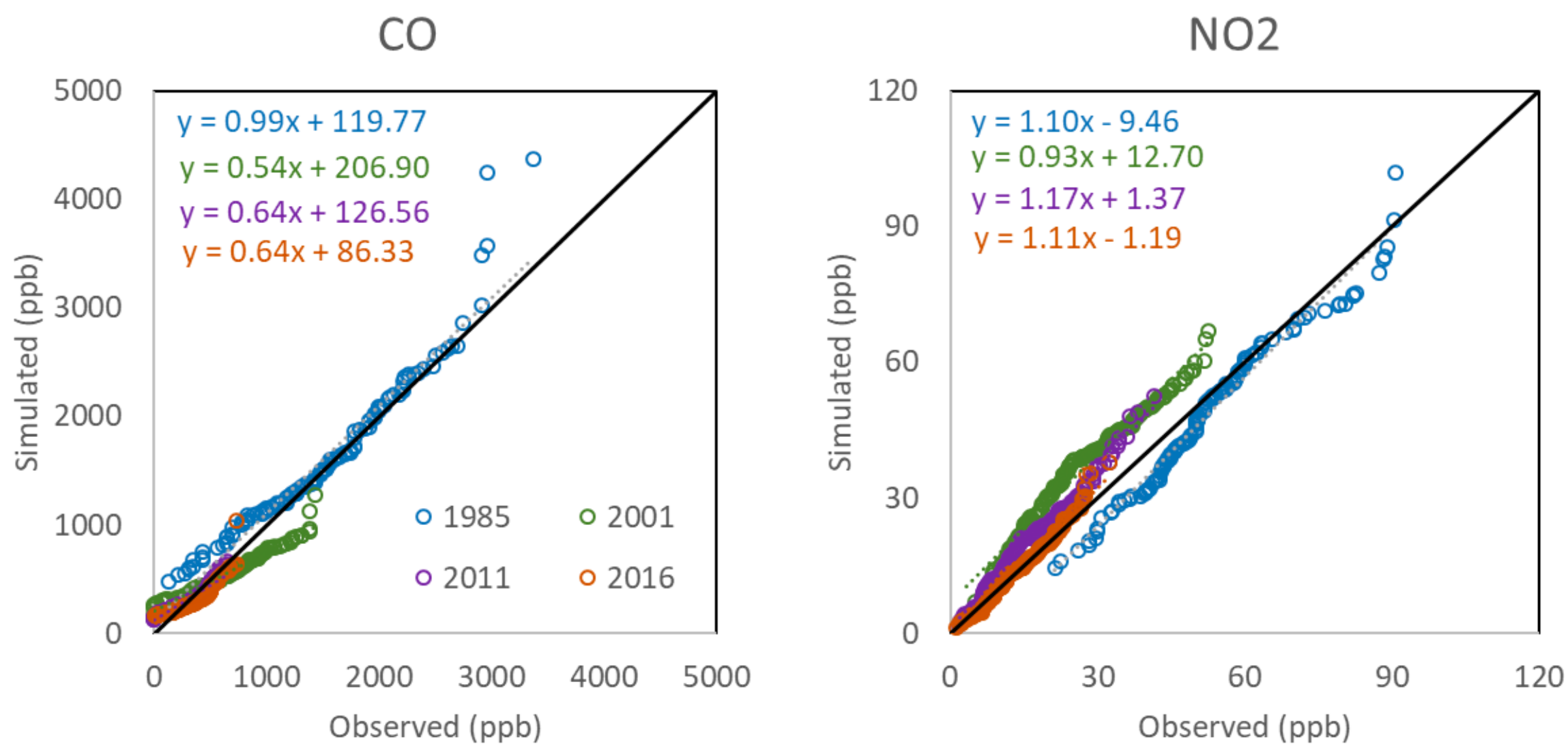

Figure S7. Comparison between simulated and observed daily average CO and NO_2 concentrations for all sites over two high-ozone periods, from June 2 to June 4 and from July 20 to July 29 in 2016. Different colors indicate different years, with regression equations shown in the corresponding colors.

Table S11. Comparison statistics between CMAQ-simulated and observed MDA8 ozone concentrations for all 24 monitoring sites over the 13-day period.

Metrics	Simulations in the study	Goal*	Criteria*
MB (ppb)	1.75	-	-
ME (ppb)	10.71	-	-
NMB (%)	2.5%	< ±5%	< ±15%
NME (%)	15.1%	< 15%	< 25%
r	0.74	> 0.75	> 0.50

* Suggested model performance from the previous study (Emery, Liu et al. 2017).
Criteria: performance achieved by two-thirds of historical applications.
Goal: performance comparable to the top one-third of historical applications.

Mean bias (MB) = $\frac{1}{N}\sum(M_j - O_j)$

Mean error (ME) = $\frac{1}{N}\sum|M_j - O_j|$

Normalized mean bias (NMB) = $\frac{\sum(M_j - O_j)}{\sum O_j} \times 100$

Normalized mean error (NME) = $\frac{\sum|M_j - O_j|}{\sum O_j} \times 100$

Correlation coefficient (r) = $\frac{\Sigma|M_j - \bar{M}_j| \times |O_j - \bar{O}_j|}{\sqrt{\Sigma(M_j - \bar{M}_j)^2 \times \Sigma(O_j - \bar{O}_j)^2}}$

N: number of observation (O) and simulation (M) pairs, as 312.
Mean modeled ozone: 72.6 ppb
Mean observed ozone: 70.8 ppb

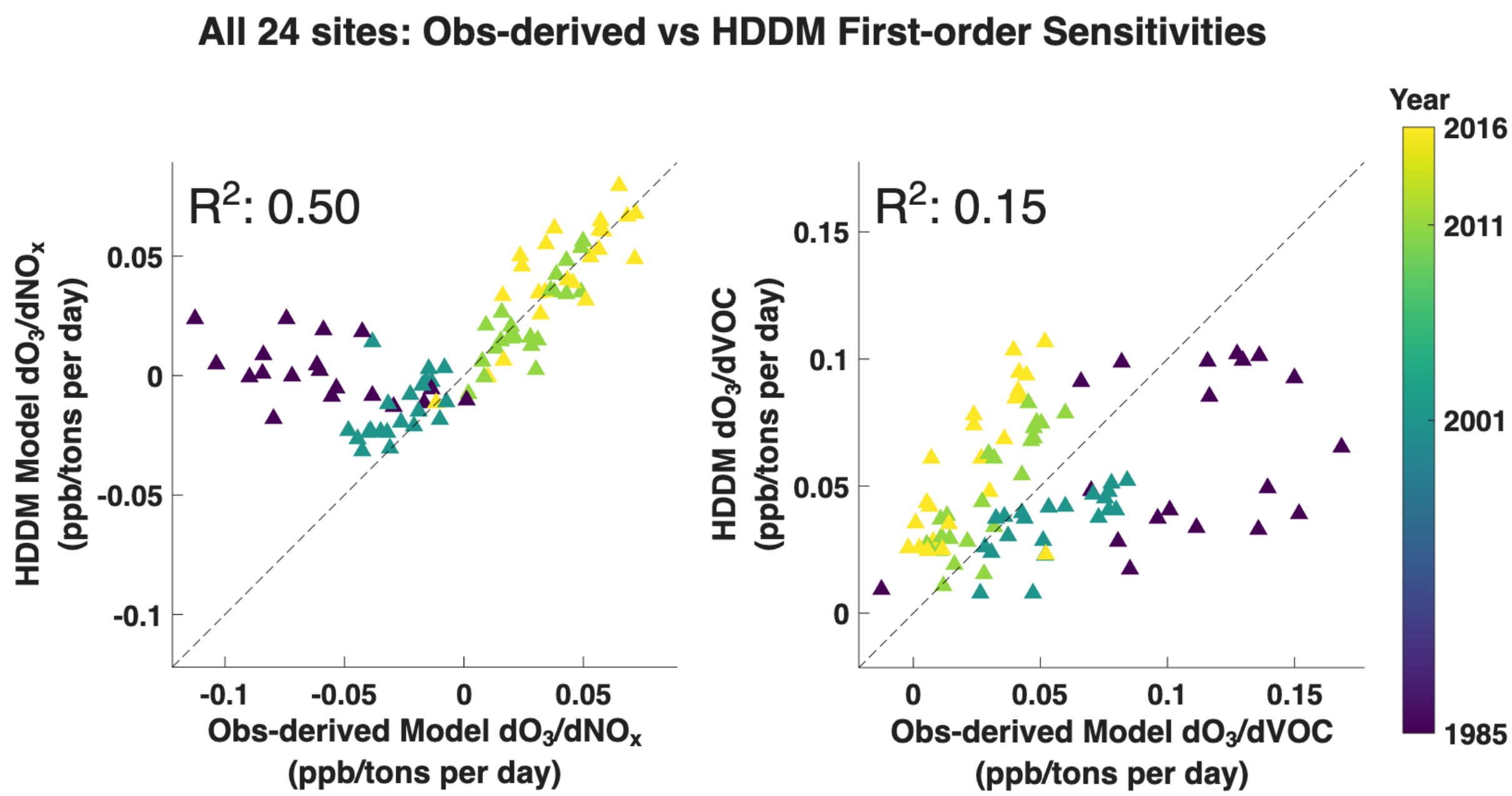

Figure S8. Comparison of first-order ozone sensitivities to precursor emissions derived from HDDM simulations (peak ozone over the 13-day high-ozone period) and from the observation-derived isopleth model (ODV) across 24 monitoring sites in the South Coast Air Basin for four simulated years (1985, 2001, 2011, and 2016). The left panel shows $\partial O_3/E_{NO_x}$ and the right panel shows $\partial O_3/E_{VOC}$, both in units of ppb/(tons per day). The color indicates the year of the points. The dashed line indicates the 1:1 reference line.

Table S12. Comparisons between CMAQ-HDDM and observation-derived first-order ozone sensitivities ($\partial O_3/\partial E_{NO_x}$, $\partial O_3/\partial E_{VOC}$), reported by year as R^2 and RMSE (ppb/(tons·day^{-1})) across SoCAB sites.

Year	R^2 ($\partial O_3/\partial E_{NO_x}$)	RMSE ($\partial O_3/\partial E_{NO_x}$)	R^2 ($\partial O_3/\partial E_{VOC}$)	RMSE ($\partial O_3/\partial E_{VOC}$)
1985	0.23	0.07	0.15	0.06
2001	0.27	0.01	0.47	0.02
2011	0.70	0.01	0.76	0.02
2016	0.67	0.01	0.53	0.04
**Overall**	**0.50**	**0.04**	**0.15**	**0.04**

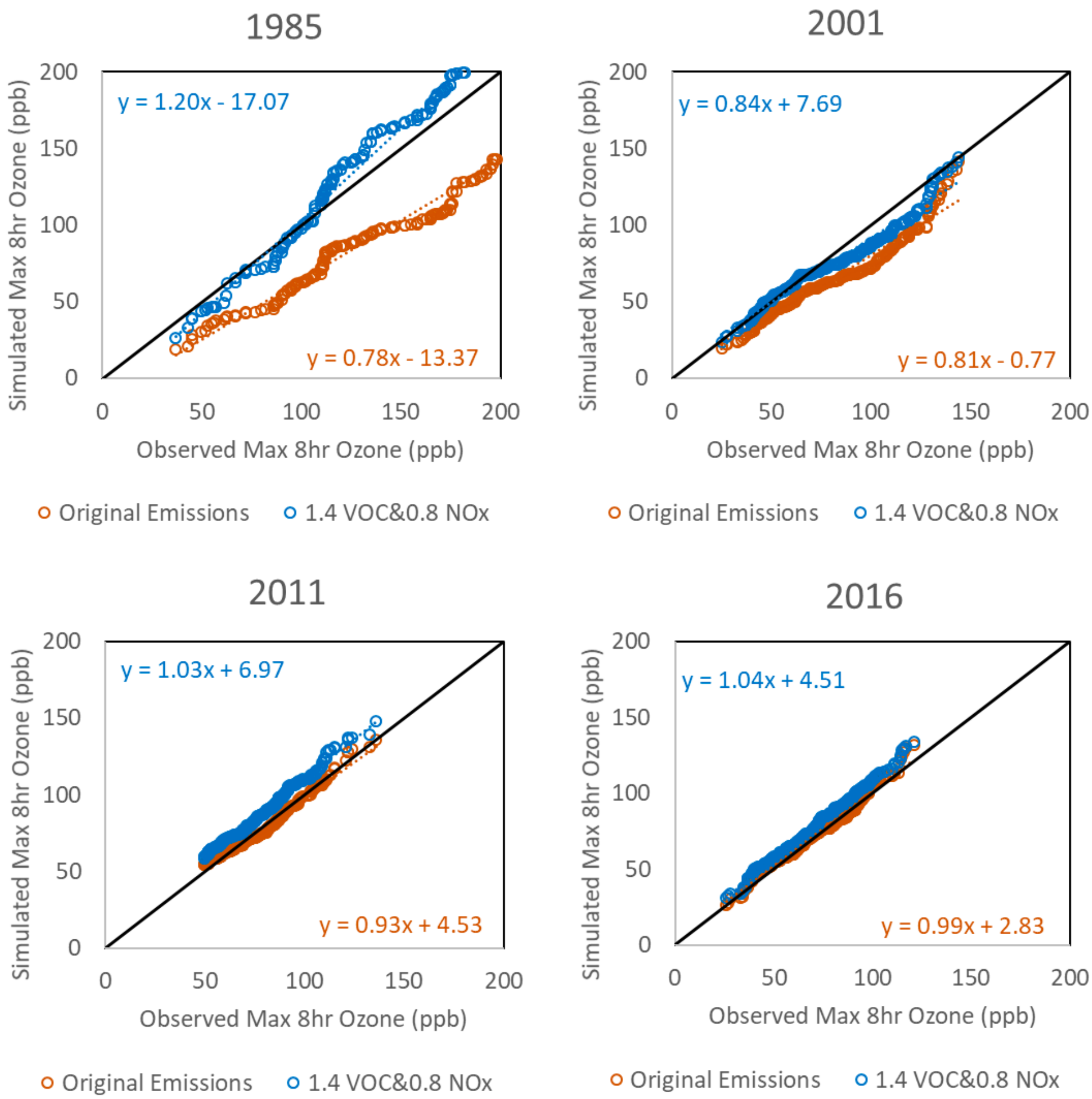

Figure S9. Comparison between simulated and observed ozone concentrations (ppb) in a rank-ordered basis for 1985, 2001, 2011, and 2016. The orange dots indicate the simulation based on original inventory emissions, and the blue dots indicate 40% increased VOC and 20% decreased NO_x emissions.

Table S13. Summary of the R^2 between CMAQ-HDDM estimates and values reconstructed using the quadratic and log-quadratic model of ozone concentration, ($\frac{\partial O_3}{\partial E_{NO_x}}$) (SN), and ($\frac{\partial O_3}{\partial E_{VOC}}$) (SV), respectively.

Site	O_3 (quadratic)	O_3 (log-quadratic)	SN (quadratic)	SN (log-quadratic)	SV (quadratic)	SV (log-quadratic)
Crestline	0.83	0.65	0.57	0.06	0.38	0.39
San Bernardino	0.70	0.42	0.49	0.03	0.22	0.20
Redlands	0.77	0.49	0.52	0.04	0.32	0.27
Banning	0.96	0.91	0.58	0.06	0.60	0.60
Fontana	0.61	0.39	0.48	0.03	0.21	0.24
Upland	0.63	0.45	0.51	0.05	0.30	0.38
Riverside	0.84	0.67	0.53	0.05	0.24	0.25
Perris	0.95	0.85	0.56	0.07	0.57	0.57
Glendora	0.62	0.42	0.48	0.01	0.17	0.19
Azusa	0.59	0.41	0.48	0.01	0.16	0.16
Pomona	0.69	0.55	0.52	0.06	0.32	0.39
Lake Elsinore	0.94	0.87	0.61	0.07	0.60	0.67
Santa Clarita	0.89	0.83	0.61	0.15	0.58	0.57
Burbank	0.74	0.64	0.44	0.06	0.09	0.04
Pasadena	0.57	0.39	0.46	0.01	0.13	0.10
Pico Rivera	0.59	0.42	0.49	0.03	0.17	0.15
La Habra	0.69	0.56	0.54	0.03	0.28	0.26
Reseda	0.86	0.76	0.54	0.07	0.57	0.59
LA North Main	0.21	0.08	0.44	0.01	0.16	0.10
Anaheim	0.51	0.42	0.54	0.10	0.36	0.33
Mission Viejo	0.59	0.46	0.56	0.10	0.61	0.58
West LA	0.51	0.33	0.45	0.04	0.13	0.09
LAX	0.12	0.10	0.38	0.17	0.02	0.03
Long Beach	0.03	0.01	0.43	0.07	0.01	0.00
**Overall**	**0.75**	**0.58**	**0.46**	**0.04**	**0.42**	**0.42**

Table S14. Summary of the RMSE between CMAQ-HDDM estimates and values reconstructed using the quadratic and log-quadratic model of ozone concentration, ($\frac{\partial O_3}{\partial E_{NO_x}}$) (SN), and ($\frac{\partial O_3}{\partial E_{VOC}}$) (SV), respectively. Ozone RMSE is in ppb; SN and SV RMSE are in ppb/(tons per day).

Site	O_3 (quadratic)	O_3 (log-quadratic)	SN (quadratic)	SN (log-quadratic)	SV (quadratic)	SV (log-quadratic)
Crestline	18.74	37.59	0.12	0.19	0.04	0.03
San Bernardino	24.71	51.84	0.17	0.27	0.05	0.04
Redlands	21.48	47.41	0.15	0.25	0.04	0.04
Banning	8.18	19.55	0.08	0.13	0.02	0.02
Fontana	24.72	45.01	0.18	0.25	0.04	0.04
Upland	24.44	40.41	0.18	0.22	0.04	0.03
Riverside	16.39	33.76	0.13	0.19	0.03	0.03
Perris	9.43	21.43	0.09	0.14	0.02	0.02
Glendora	28.17	53.78	0.23	0.30	0.05	0.05
Azusa	26.72	48.26	0.23	0.28	0.05	0.04
Pomona	22.34	36.26	0.17	0.21	0.04	0.03
Lake Elsinore	8.94	19.94	0.08	0.13	0.01	0.01
Santa Clarita	9.38	14.56	0.07	0.09	0.01	0.01
Burbank	13.34	16.97	0.14	0.16	0.01	0.01
Pasadena	25.13	43.09	0.24	0.29	0.04	0.04
Pico Rivera	20.52	33.13	0.19	0.23	0.03	0.03
La Habra	18.08	30.17	0.15	0.19	0.03	0.03
Reseda	11.74	18.92	0.09	0.12	0.01	0.01
LA North Main	26.08	35.99	0.24	0.28	0.03	0.03
Anaheim	16.35	21.54	0.11	0.13	0.02	0.02
Mission Viejo	14.04	19.36	0.08	0.11	0.01	0.01
West LA	18.24	25.36	0.14	0.18	0.02	0.02
LAX	15.82	14.03	0.13	0.14	0.02	0.01
Long Beach	18.61	19.34	0.15	0.18	0.02	0.02
**Overall**	**19.37**	**33.54**	**0.16**	**0.20**	**0.03**	**0.03**

Table S15. Comparison (R^2) of observed ozone with CMAQ-HDDM- and observation-derived model estimates along the historical emissions trajectory. Also included are R^2 between CMAQ-HDDM-derived and observation-derived model estimated ($\frac{\partial O_3}{\partial E_{NO_x}}$) (SN), and ($\frac{\partial O_3}{\partial E_{VOC}}$) (SV), respectively, along the historical emissions trajectory.

Site	O_3 (HDDM)	O_3 (Obs)	SN	SV
Crestline	0.91	0.97	0.99	0.83
San Bernardino	0.76	0.94	0.99	0.44
Redlands	0.84	0.95	0.97	0.89
Banning	0.69	0.84	0.97	0.09
Fontana	0.78	0.93	0.97	0.01
Upland	0.81	0.97	0.99	0.07
Riverside	0.78	0.95	0.99	0.22
Perris	0.76	0.9	0.99	0.53
Glendora	0.80	0.97	0.97	0.00
Azusa	0.75	0.95	0.89	0.09
Pomona	0.74	0.94	0.98	0.14
Lake Elsinore	0.79	0.93	0.99	0.18
Santa Clarita	0.69	0.90	0.97	0.2
Burbank	0.52	0.93	0.97	0.11
Pasadena	0.64	0.95	0.98	0.00
Pico Rivera	0.65	0.95	0.19	0.07
La Habra	0.64	0.96	0.94	0.00
Reseda	0.55	0.86	0.98	0.01
LA North Main	0.60	0.98	0.88	0.00
Anaheim	0.70	0.96	0.98	0.03
Mission Viejo	0.65	0.88	0.98	0.05
West LA	0.30	0.94	0.98	0.00
LAX	0.43	0.80	0.28	0.11
Long Beach	0.41	0.96	0.97	0.63
**Overall**	**0.70**	**0.96**	**0.75**	**0.18**

Table S16. Comparison (RMSE) of observed ozone with CMAQ-HDDM- and observation-derived model estimates along the historical emissions trajectory. Also included are RMSE between CMAQ-HDDM-derived and observation-derived model estimated ($\frac{\partial O_3}{\partial E_{NO_x}}$) (SN), and ($\frac{\partial O_3}{\partial E_{VOC}}$) (SV), respectively, along the historical emissions trajectory. Ozone RMSE is in ppb; SN and SV RMSE are in ppb/(tons per day).

Site	O_3 (HDDM)	O_3 (Obs)	SN	SV
Crestline	17.45	6.83	0.03	0.03
San Bernardino	33.07	9.89	0.04	0.04
Redlands	26.77	8.15	0.04	0.02
Banning	29.99	10.24	0.06	0.04
Fontana	32.10	14.60	0.05	0.06
Upland	30.93	8.69	0.05	0.07
Riverside	26.04	9.51	0.03	0.05
Perris	26.98	9.85	0.03	0.03
Glendora	30.53	8.85	0.07	0.06
Azusa	34.15	10.17	0.05	0.06
Pomona	42.11	9.48	0.04	0.05
Lake Elsinore	29.13	5.69	0.03	0.02
Santa Clarita	24.49	12.76	0.06	0.08
Burbank	42.03	9.54	0.03	0.08
Pasadena	31.71	9.74	0.06	0.08
Pico Rivera	28.71	9.00	0.05	0.05
La Habra	33.94	6.00	0.04	0.05
Reseda	20.52	10.47	0.03	0.05
LA North Main	25.87	4.14	0.04	0.04
Anaheim	27.04	4.87	0.03	0.05
Mission Viejo	15.48	8.19	0.03	0.04
West LA	24.22	5.52	0.02	0.05
LAX	17.51	4.27	0.03	0.03
Long Beach	13.63	2.90	0.03	0.03
**Overall**	**28.54**	**8.92**	**0.04**	**0.05**

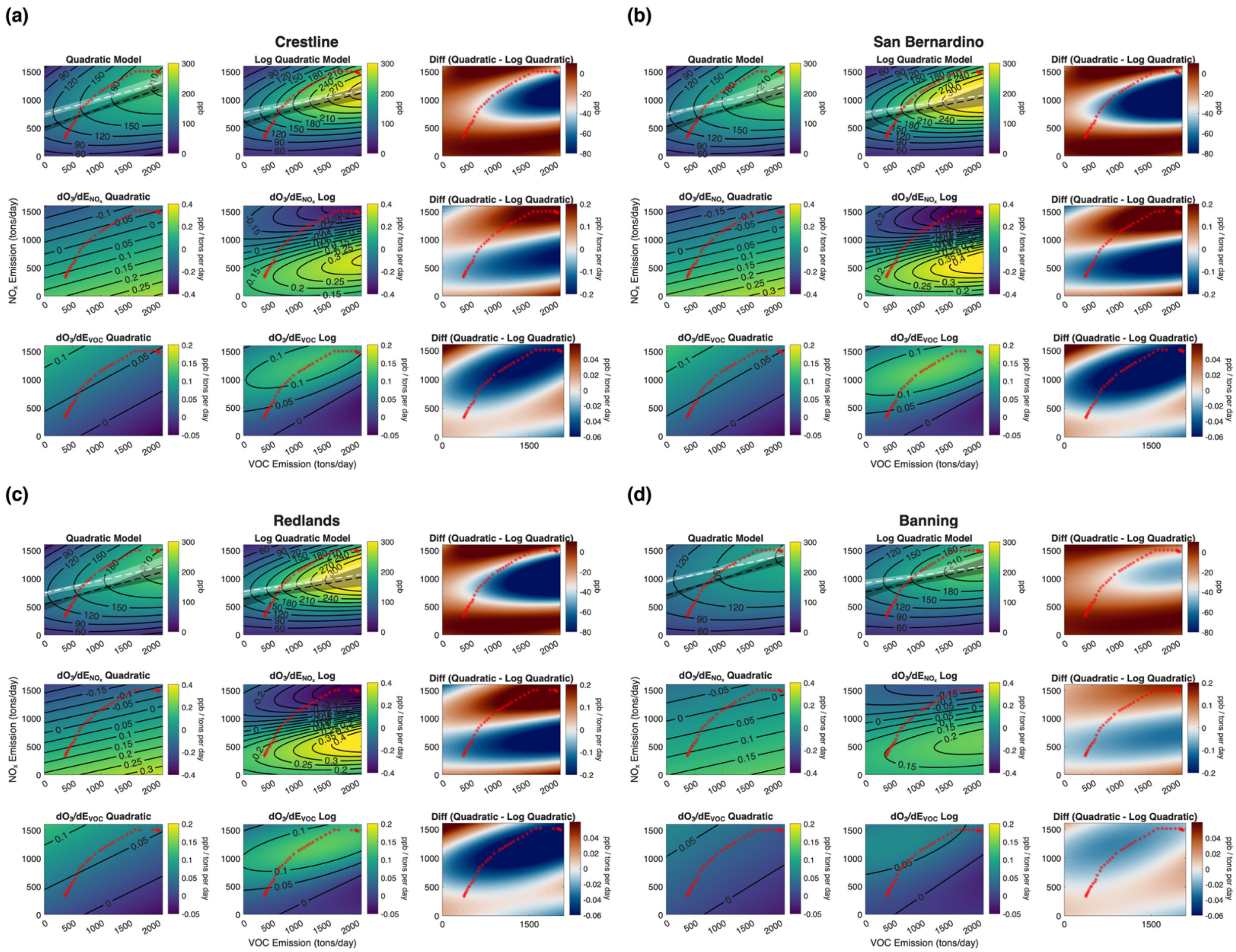
(a)
Crestline
(b)
San Bernardino
(c)
Redlands
(d)
Banning
Quadratic Model
Log Quadratic Model
Diff (Quadratic - Log Quadratic)
dO3/dENOx Quadratic
dO3/dENOx Log
dO3/dEVOC Quadratic
dO3/dEVOC Log
NOx Emission (tons/day)
VOC Emission (tons/day)
ppb
ppb / tons per day

(e)

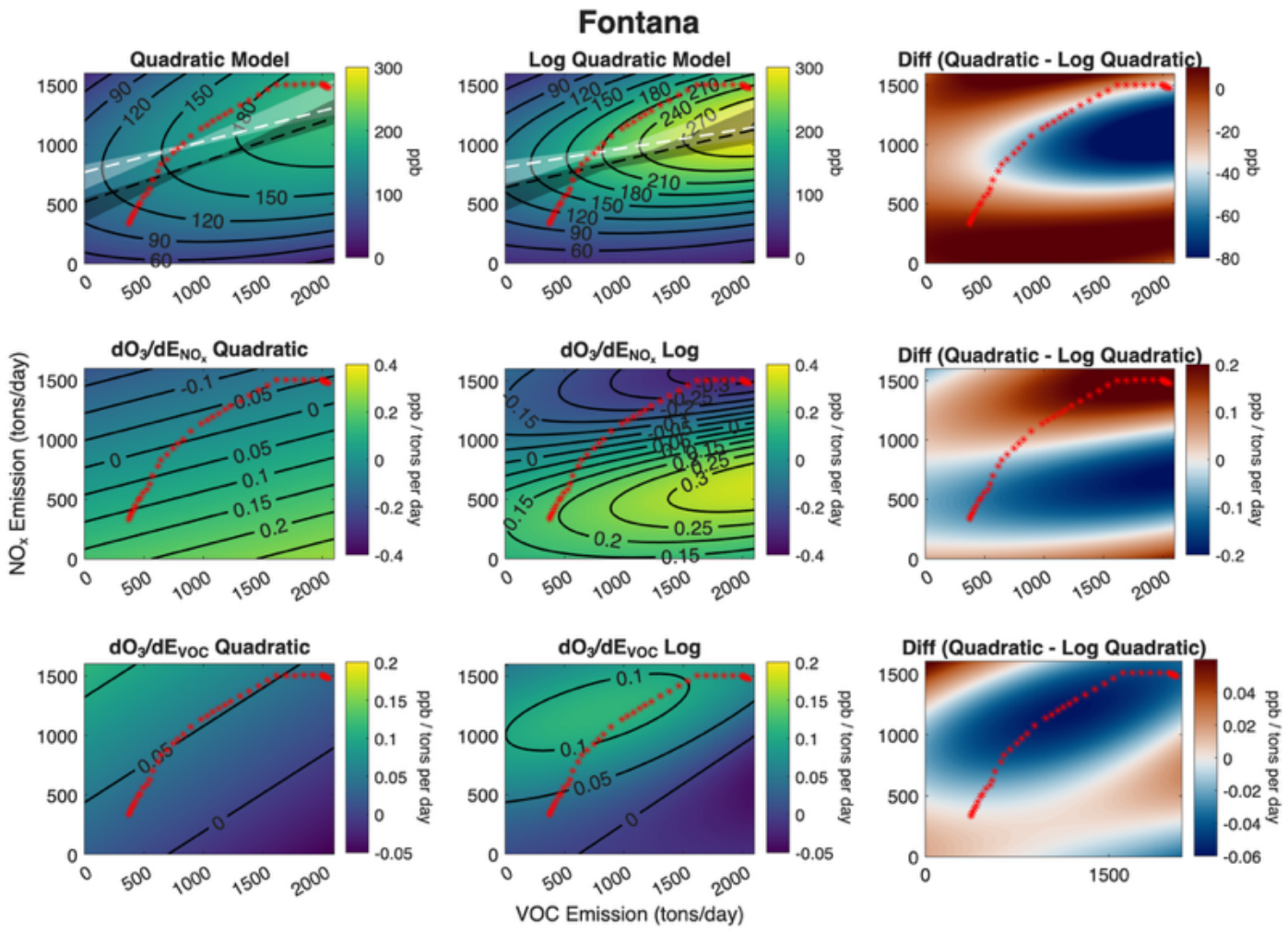
Fontana
Quadratic Model
Log Quadratic Model
Diff (Quadratic - Log Quadratic)
dO3/dENOx Quadratic
dO3/dENOx Log
dO3/dEVOC Quadratic
dO3/dEVOC Log
NOx Emission (tons/day)
VOC Emission (tons/day)
ppb
ppb / tons per day

(f)

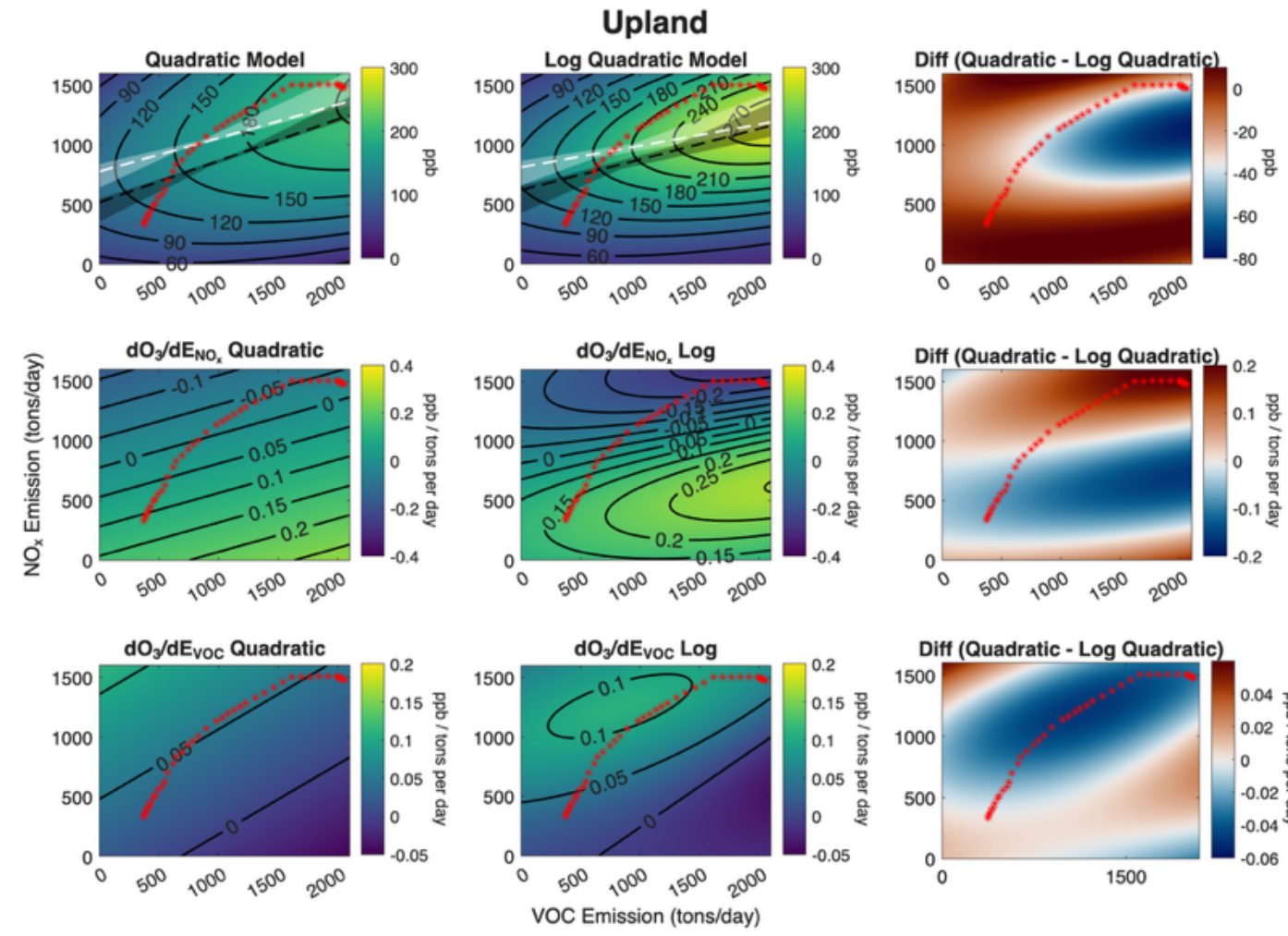
Upland
Quadratic Model
Log Quadratic Model
Diff (Quadratic - Log Quadratic)
dO3/dENOx Quadratic
dO3/dENOx Log
dO3/dEVOC Quadratic
dO3/dEVOC Log
NOx Emission (tons/day)
VOC Emission (tons/day)
ppb
ppb / tons per day

(g)

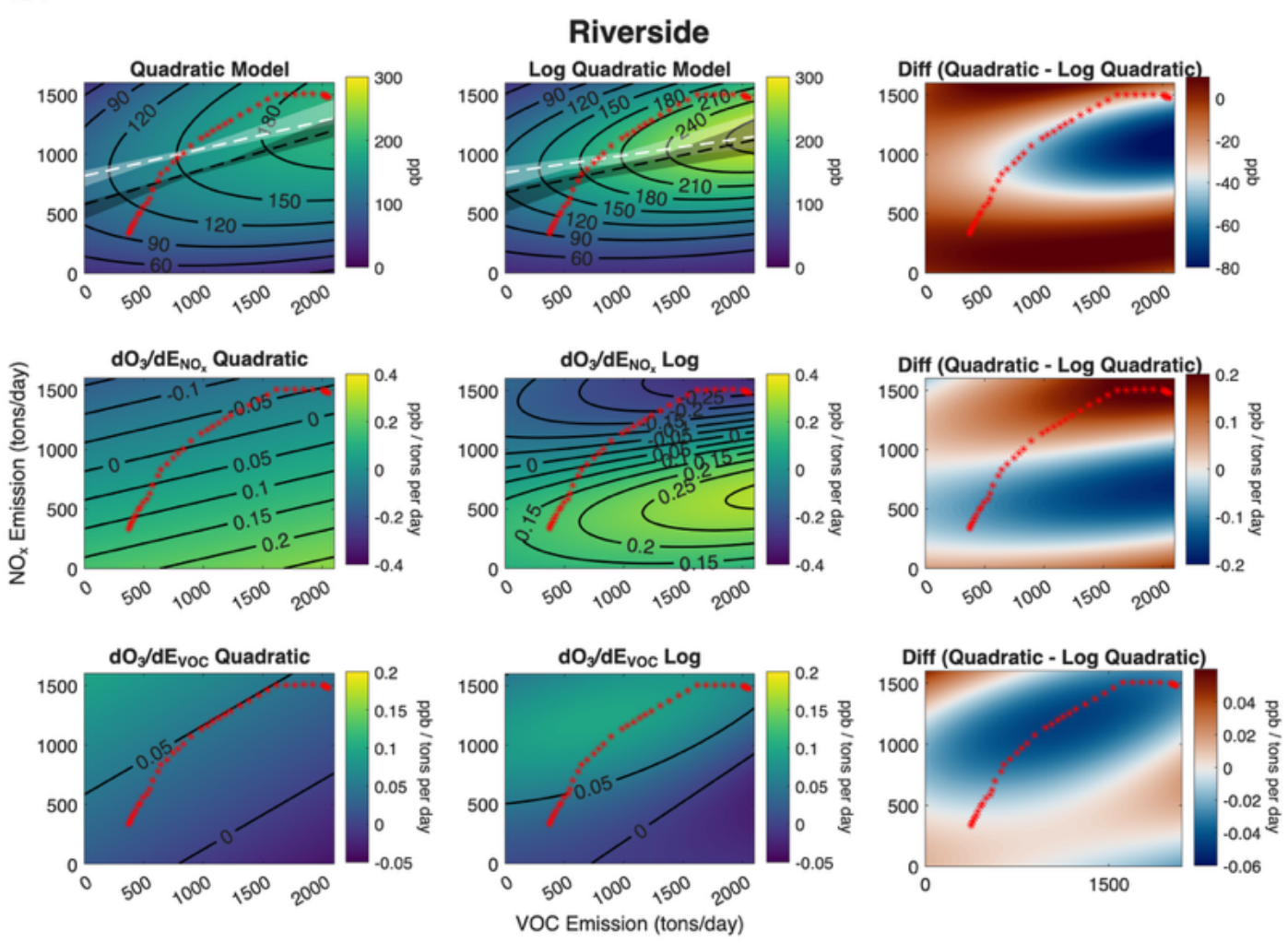
Riverside
Quadratic Model
Log Quadratic Model
Diff (Quadratic - Log Quadratic)
dO3/dENOx Quadratic
dO3/dENOx Log
dO3/dEVOC Quadratic
dO3/dEVOC Log
NOx Emission (tons/day)
VOC Emission (tons/day)
ppb
ppb / tons per day

(h)

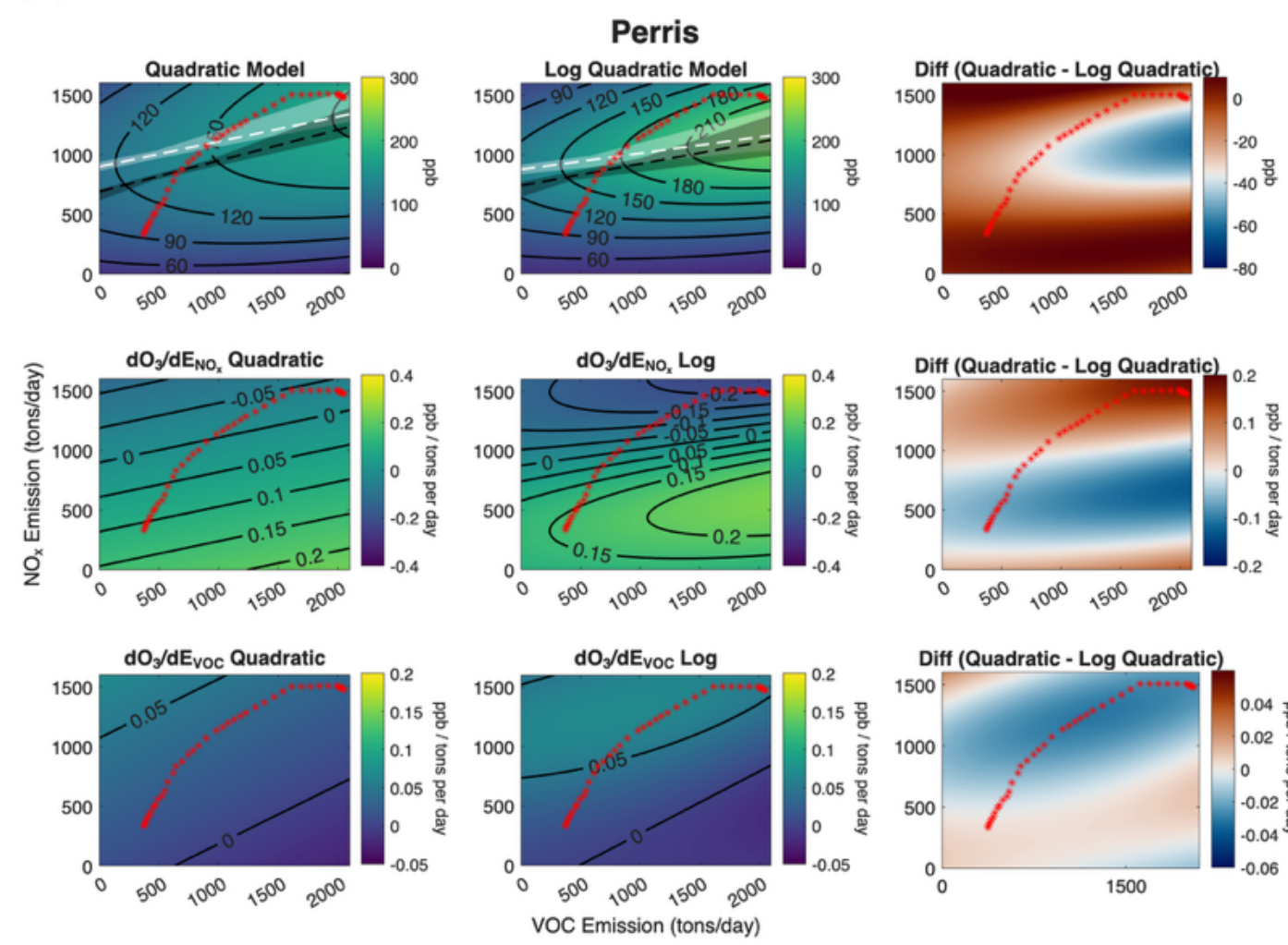
Perris
Quadratic Model
Log Quadratic Model
Diff (Quadratic - Log Quadratic)
dO3/dENOx Quadratic
dO3/dENOx Log
dO3/dEVOC Quadratic
dO3/dEVOC Log
NOx Emission (tons/day)
VOC Emission (tons/day)
ppb
ppb / tons per day

(i)

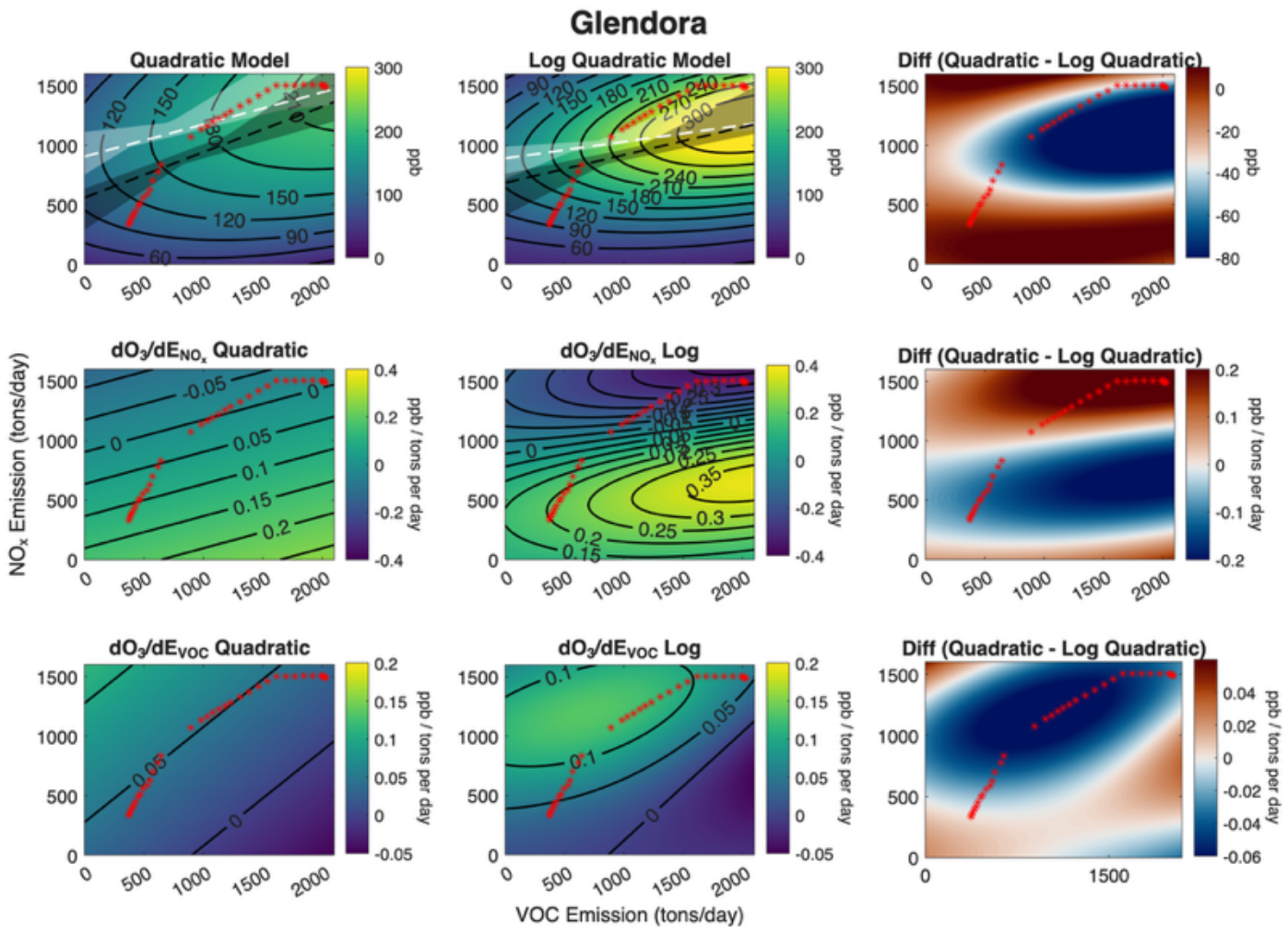
Glendora
Quadratic Model
Log Quadratic Model
Diff (Quadratic - Log Quadratic)
dO3/dENOx Quadratic
dO3/dENOx Log
dO3/dEVOC Quadratic
dO3/dEVOC Log
NOx Emission (tons/day)
VOC Emission (tons/day)
ppb
ppb / tons per day

(j)

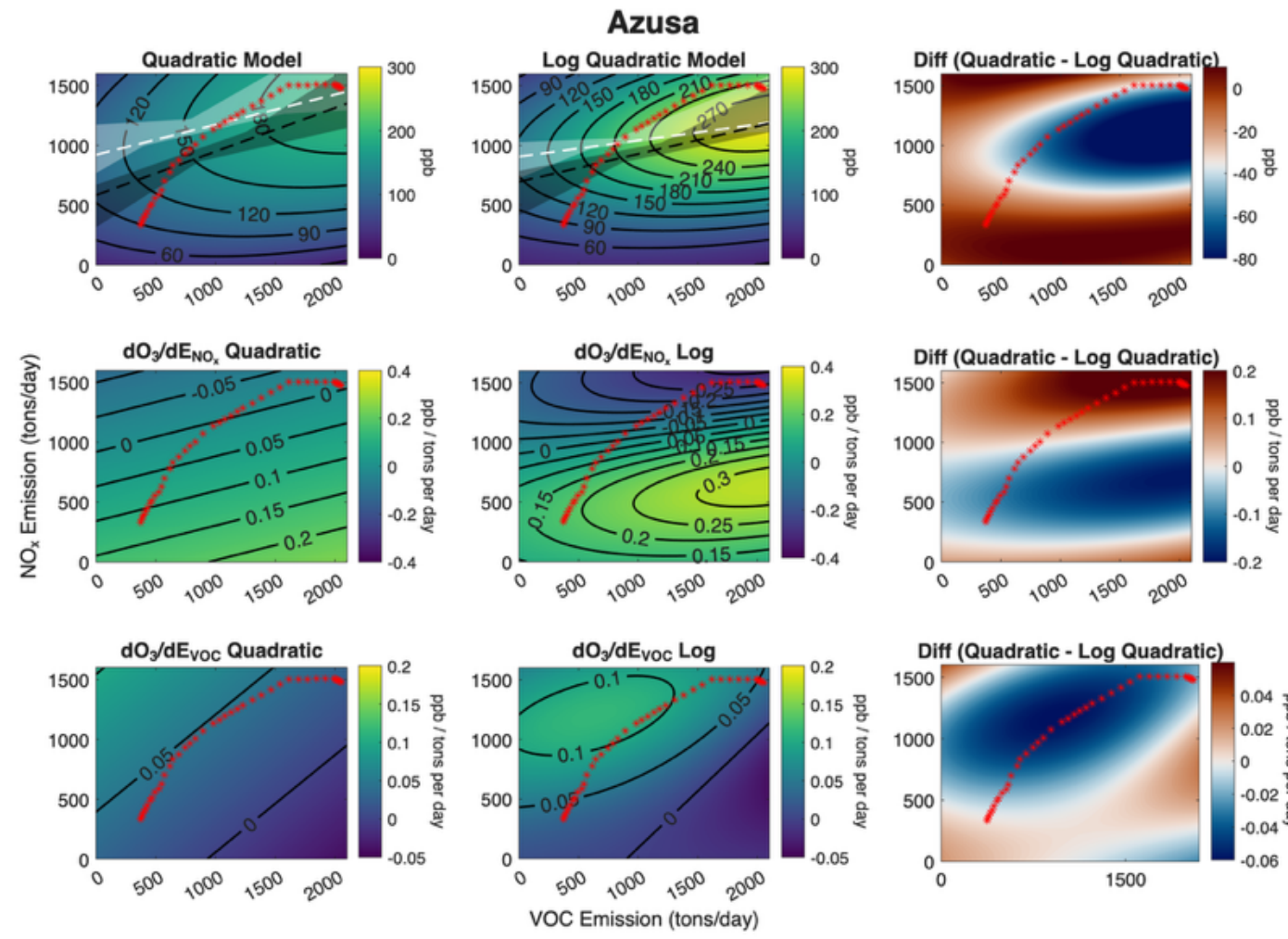
Azusa
Quadratic Model
Log Quadratic Model
Diff (Quadratic - Log Quadratic)
dO3/dENOx Quadratic
dO3/dENOx Log
dO3/dEVOC Quadratic
dO3/dEVOC Log
NOx Emission (tons/day)
VOC Emission (tons/day)
ppb
ppb / tons per day

(k)

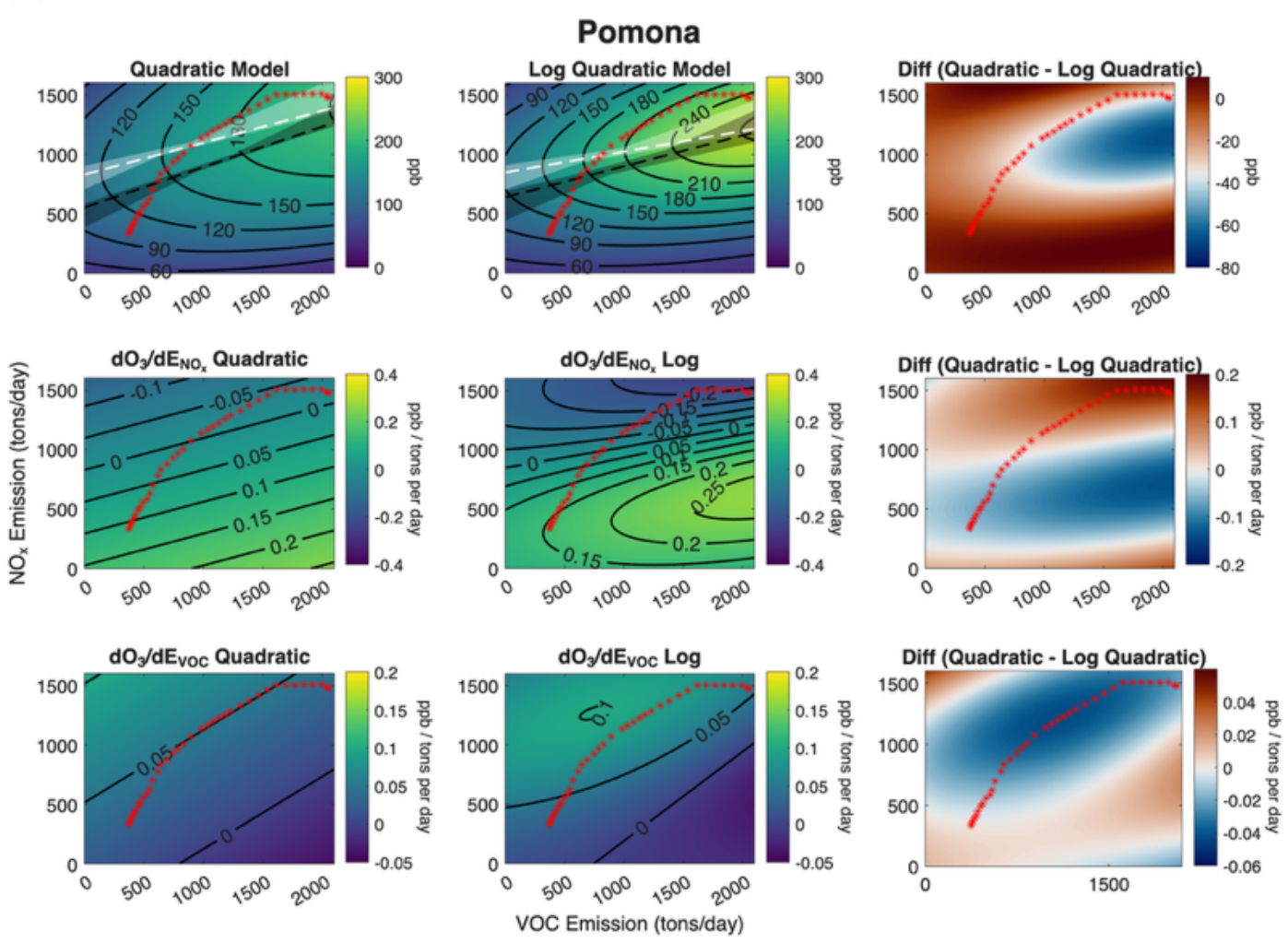
Pomona
Quadratic Model
Log Quadratic Model
Diff (Quadratic - Log Quadratic)
dO3/dENOx Quadratic
dO3/dENOx Log
dO3/dEVOC Quadratic
dO3/dEVOC Log
NOx Emission (tons/day)
VOC Emission (tons/day)
ppb
ppb / tons per day

(l)

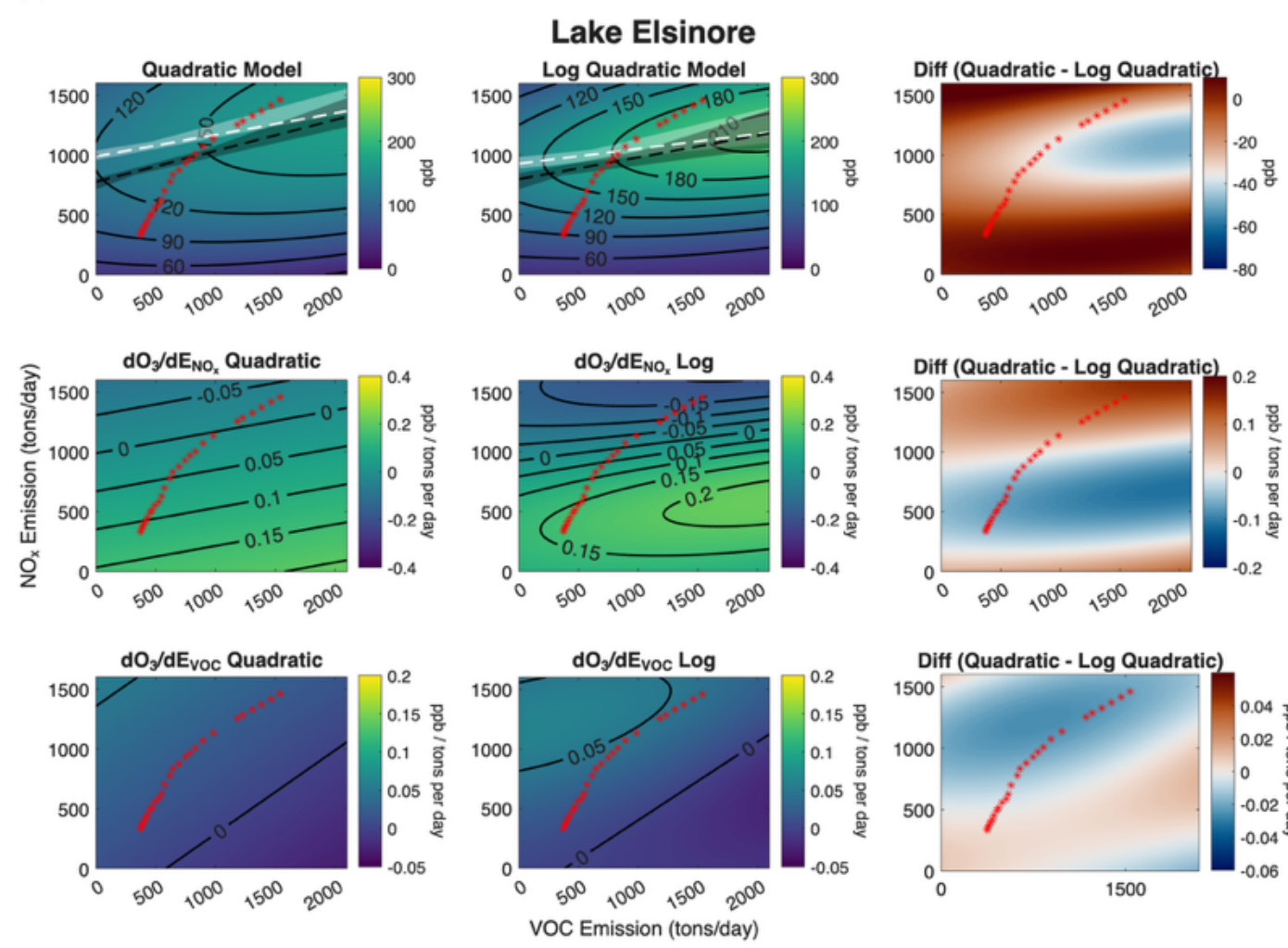
Lake Elsinore
Quadratic Model
Log Quadratic Model
Diff (Quadratic - Log Quadratic)
dO3/dENOx Quadratic
dO3/dENOx Log
dO3/dEVOC Quadratic
dO3/dEVOC Log
NOx Emission (tons/day)
VOC Emission (tons/day)
ppb
ppb / tons per day

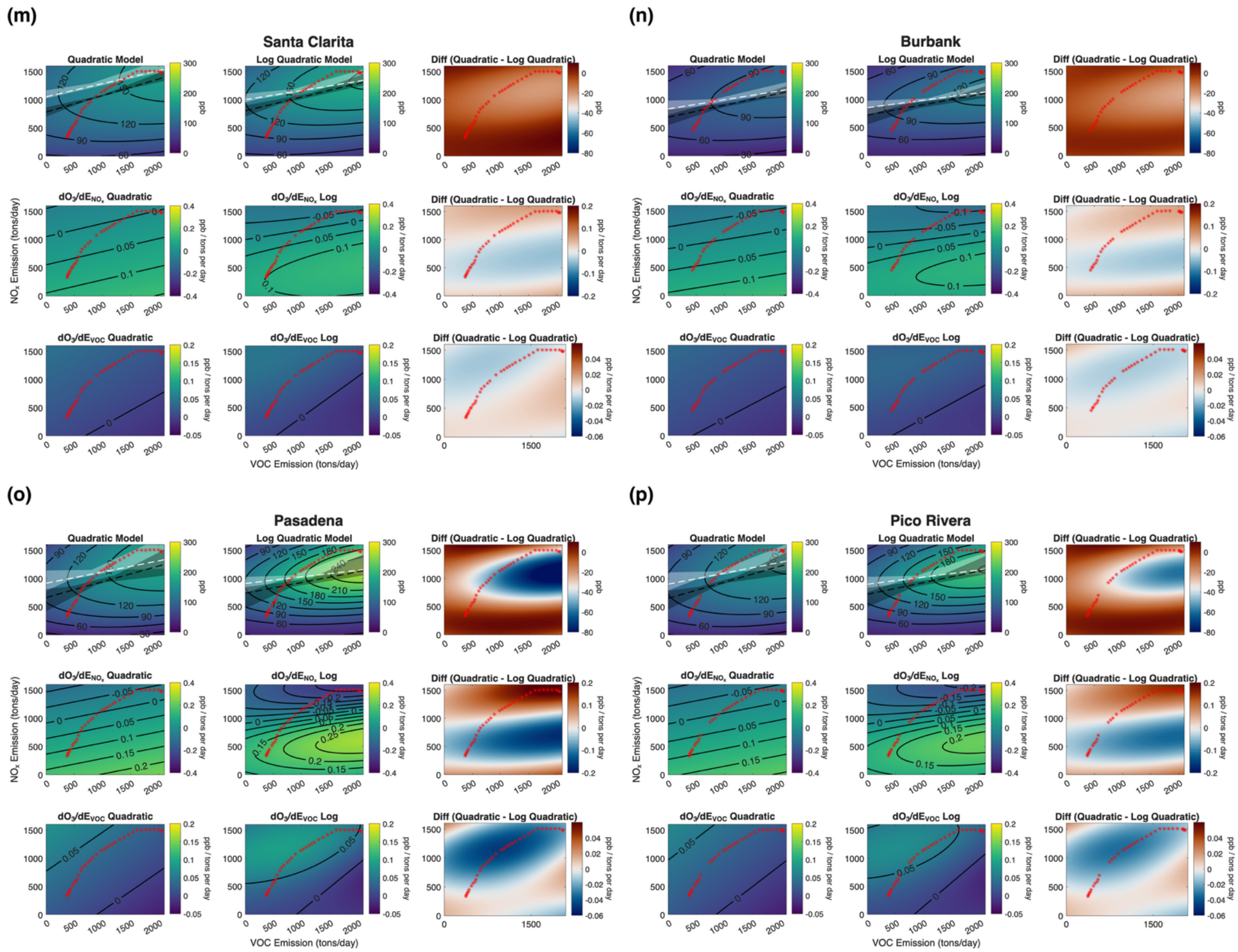
(m)
Santa Clarita
(n)
Burbank
(o)
Pasadena
(p)
Pico Rivera
Quadratic Model
Log Quadratic Model
Diff (Quadratic - Log Quadratic)
dO3/dENOx Quadratic
dO3/dENOx Log
dO3/dEVOC Quadratic
dO3/dEVOC Log
NOx Emission (tons/day)
VOC Emission (tons/day)
ppb
ppb / tons per day

(q)

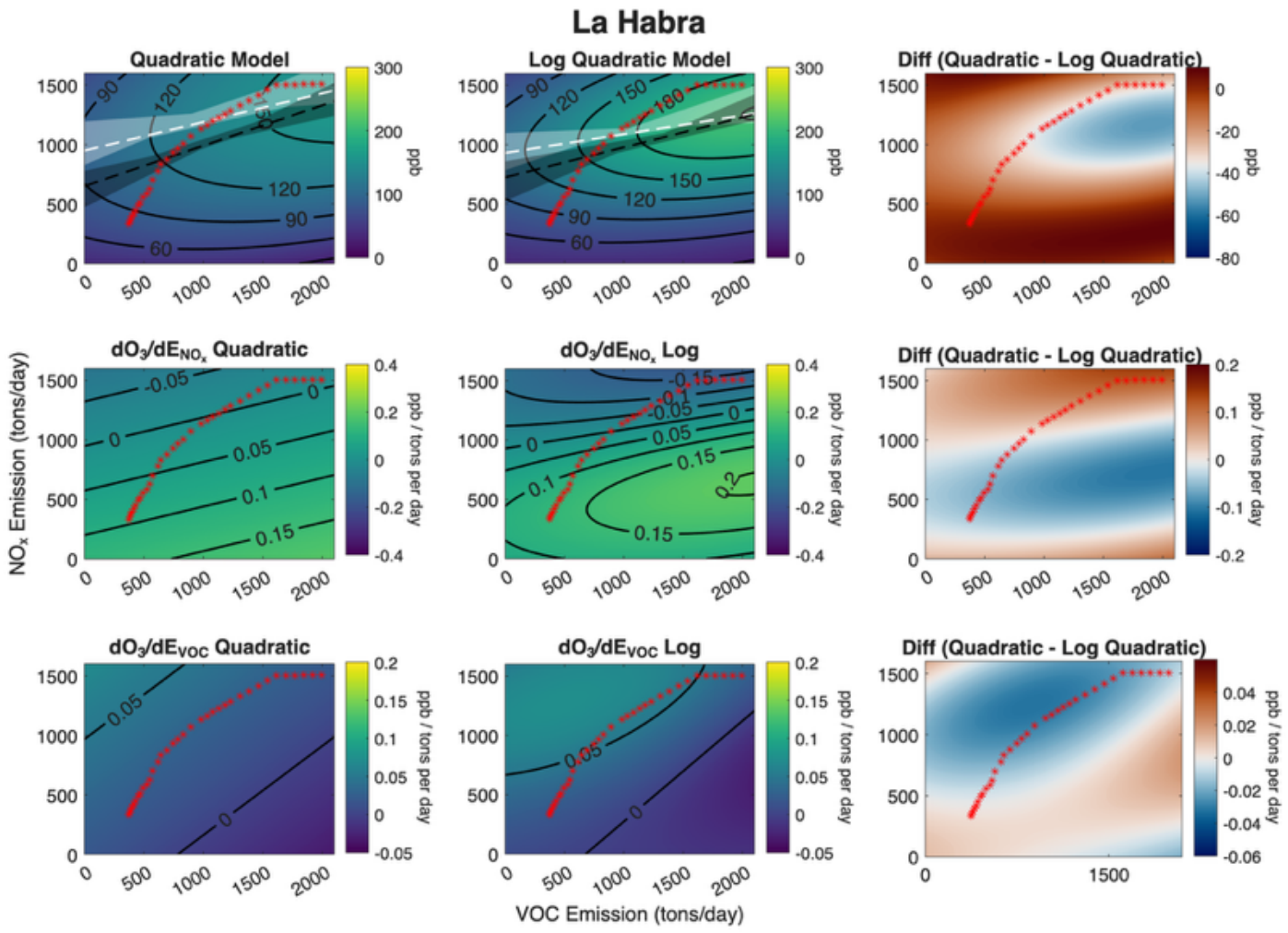
La Habra
Quadratic Model
Log Quadratic Model
Diff (Quadratic - Log Quadratic)
dO3/dENOx Quadratic
dO3/dENOx Log
dO3/dEVOC Quadratic
dO3/dEVOC Log
NOx Emission (tons/day)
VOC Emission (tons/day)
ppb
ppb / tons per day

(r)

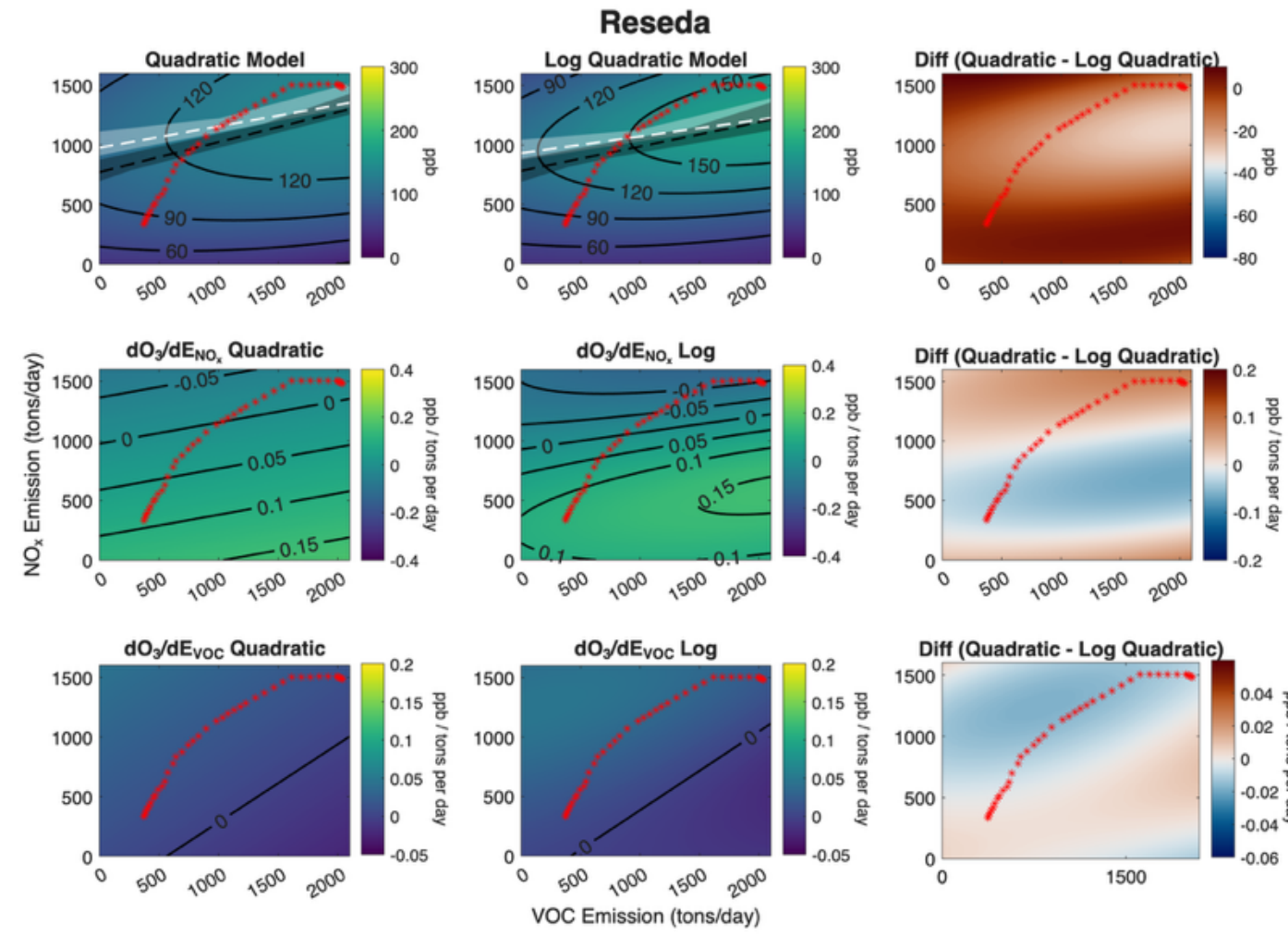
Reseda
Quadratic Model
Log Quadratic Model
Diff (Quadratic - Log Quadratic)
dO3/dENOx Quadratic
dO3/dENOx Log
dO3/dEVOC Quadratic
dO3/dEVOC Log
NOx Emission (tons/day)
VOC Emission (tons/day)
ppb
ppb / tons per day

(s)

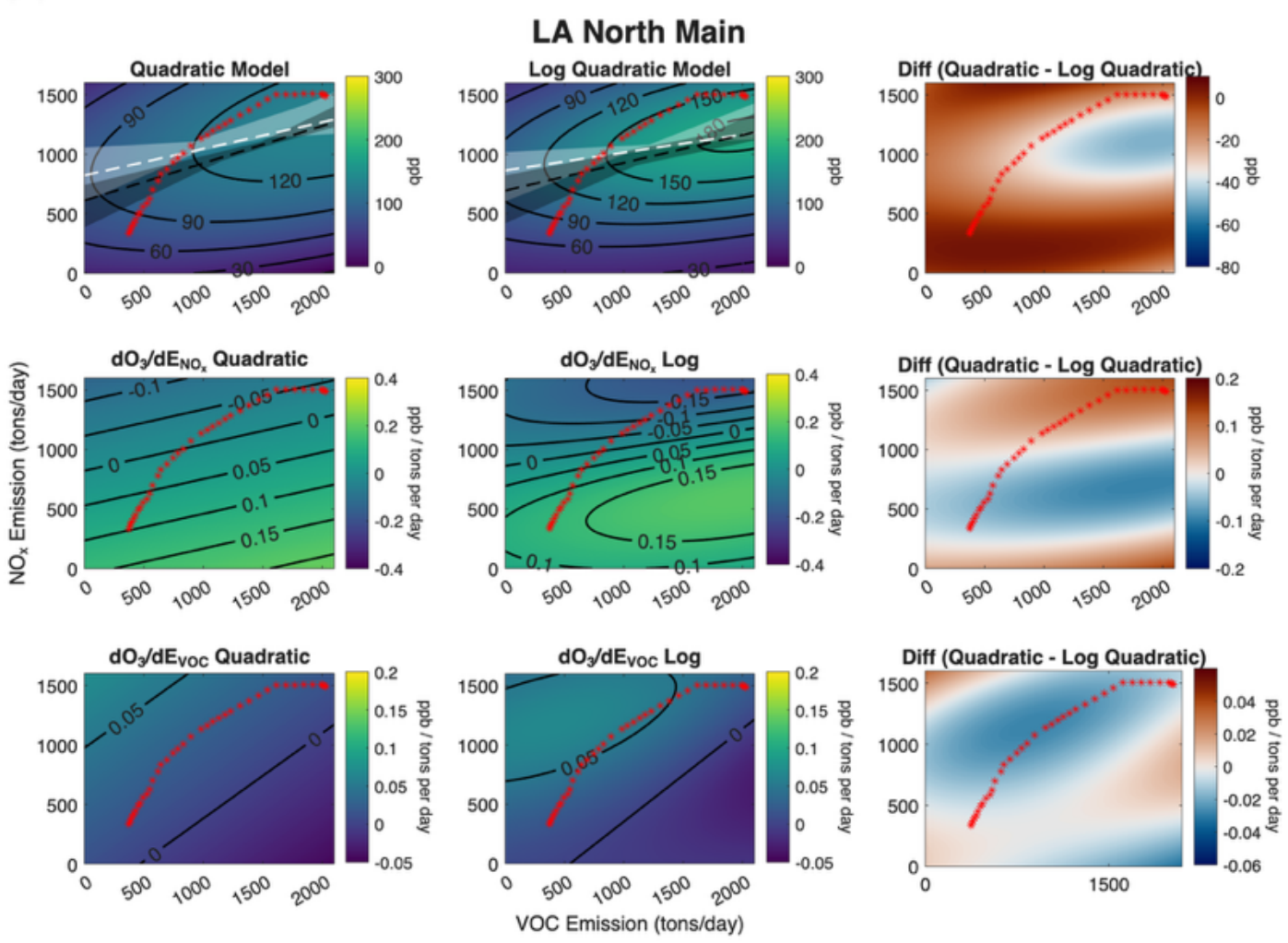
LA North Main
Quadratic Model
Log Quadratic Model
Diff (Quadratic - Log Quadratic)
dO3/dENOx Quadratic
dO3/dENOx Log
dO3/dEVOC Quadratic
dO3/dEVOC Log
NOx Emission (tons/day)
VOC Emission (tons/day)
ppb
ppb / tons per day

(t)

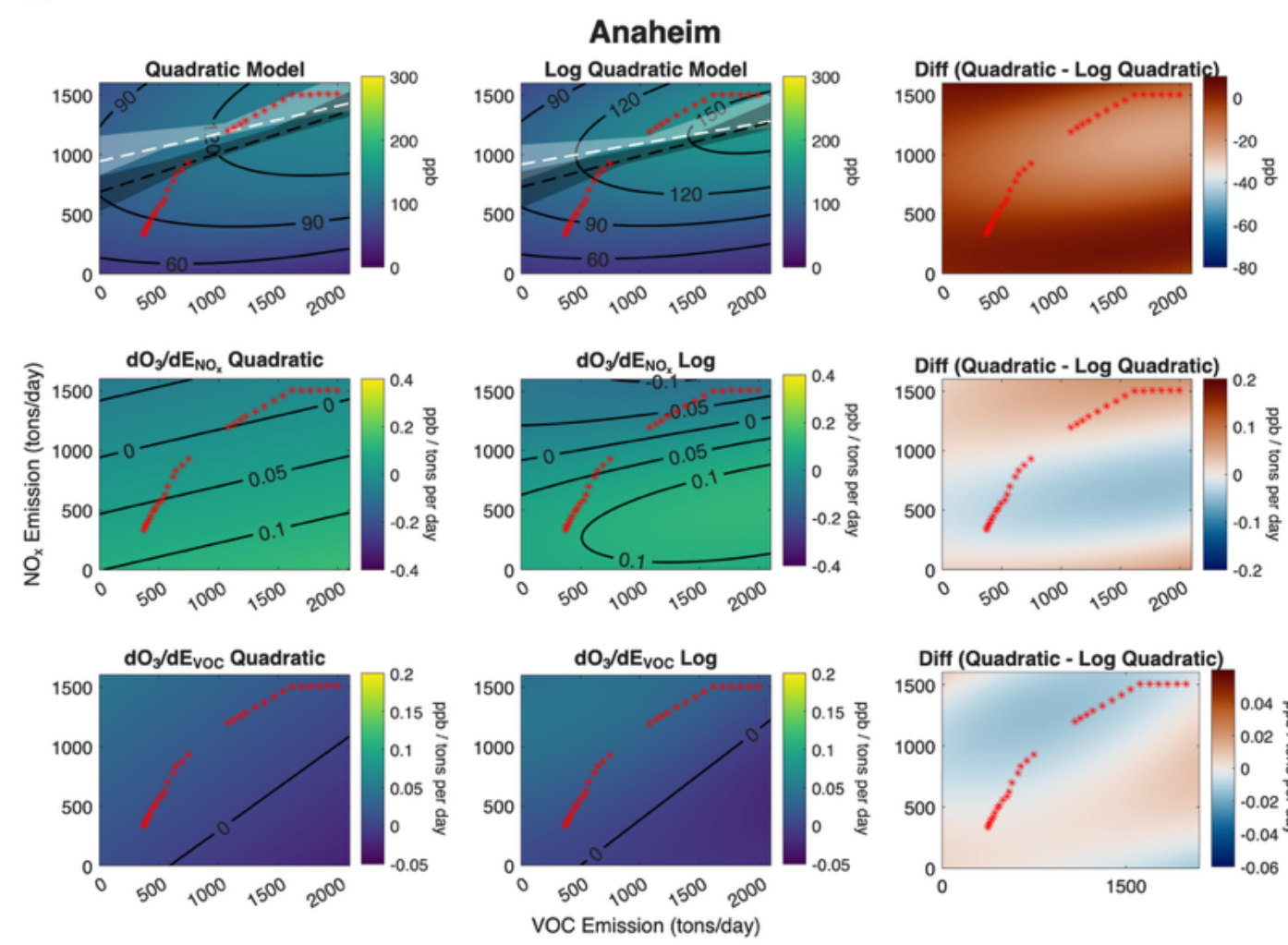
Anaheim
Quadratic Model
Log Quadratic Model
Diff (Quadratic - Log Quadratic)
dO3/dENOx Quadratic
dO3/dENOx Log
dO3/dEVOC Quadratic
dO3/dEVOC Log
NOx Emission (tons/day)
VOC Emission (tons/day)
ppb
ppb / tons per day

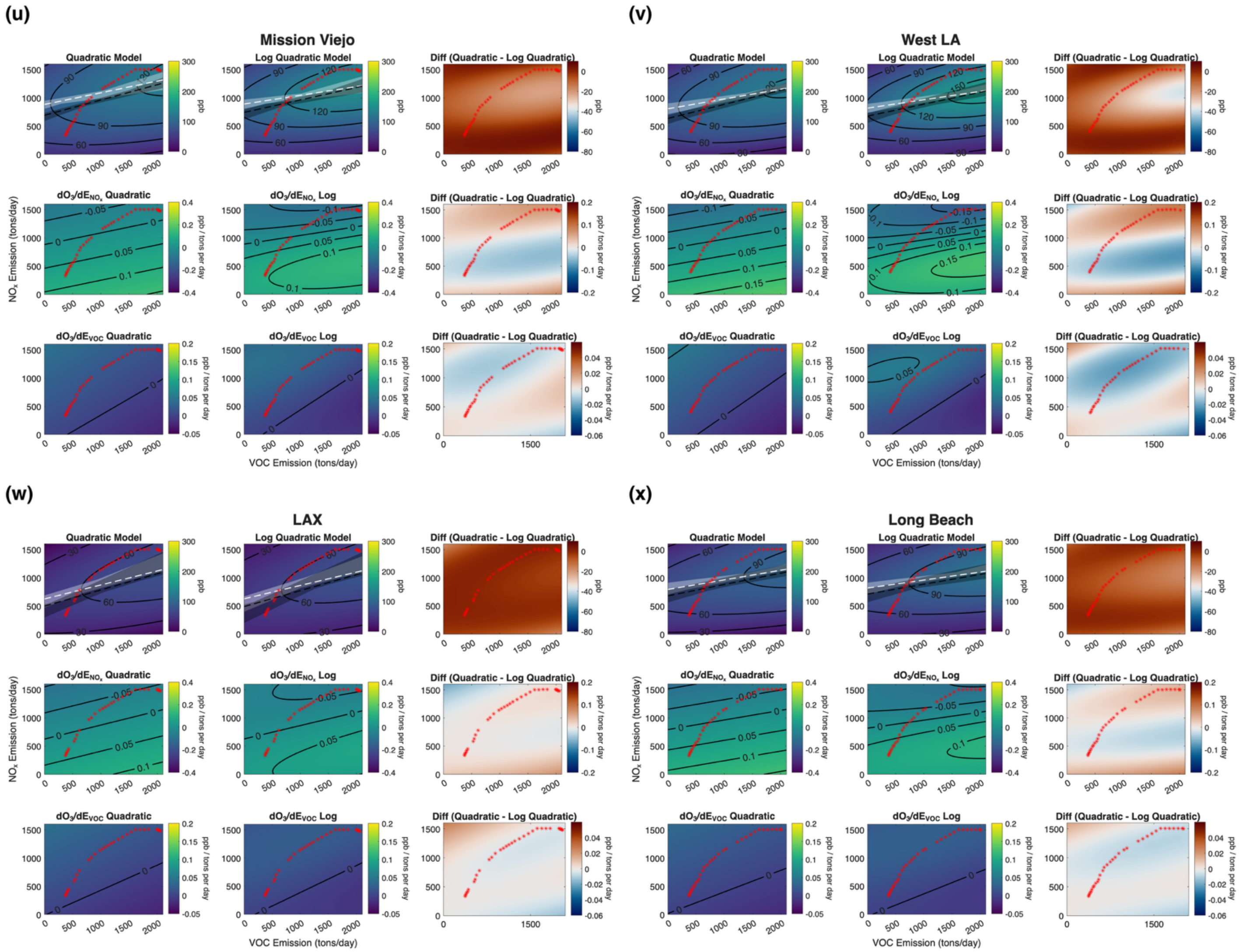

Figure S10. Comparisons between the CMAQ-HDDM-derived quadratic model and log-quadratic model ozone-emission concentrations and sensitivity isopleth for 24 monitoring sites in SoCAB (panels a to x). In each panel, the first column shows the quadratic model-based isopleths. The second column shows the log-quadratic model-based isopleths. The third column shows the difference between those two. The first row is the ozone concentration isopleths; the second row is the ozone-to-NO_x emissions sensitivity isopleths; and the third row is the ozone-to-VOC emissions sensitivity isopleths. The black dashed line indicates the zero-NO_x-sensitivity line, and the white dashed line indicates the equal-NO_x-VOC sensitivity line. The shaded area around each line spans the full range of the 15 leave-one-out fits, representing the uncertainty in the construction of the CMAQ-HDDM-derived ridge line. The red points show the historical emission trajectory from 1980 to 2019.

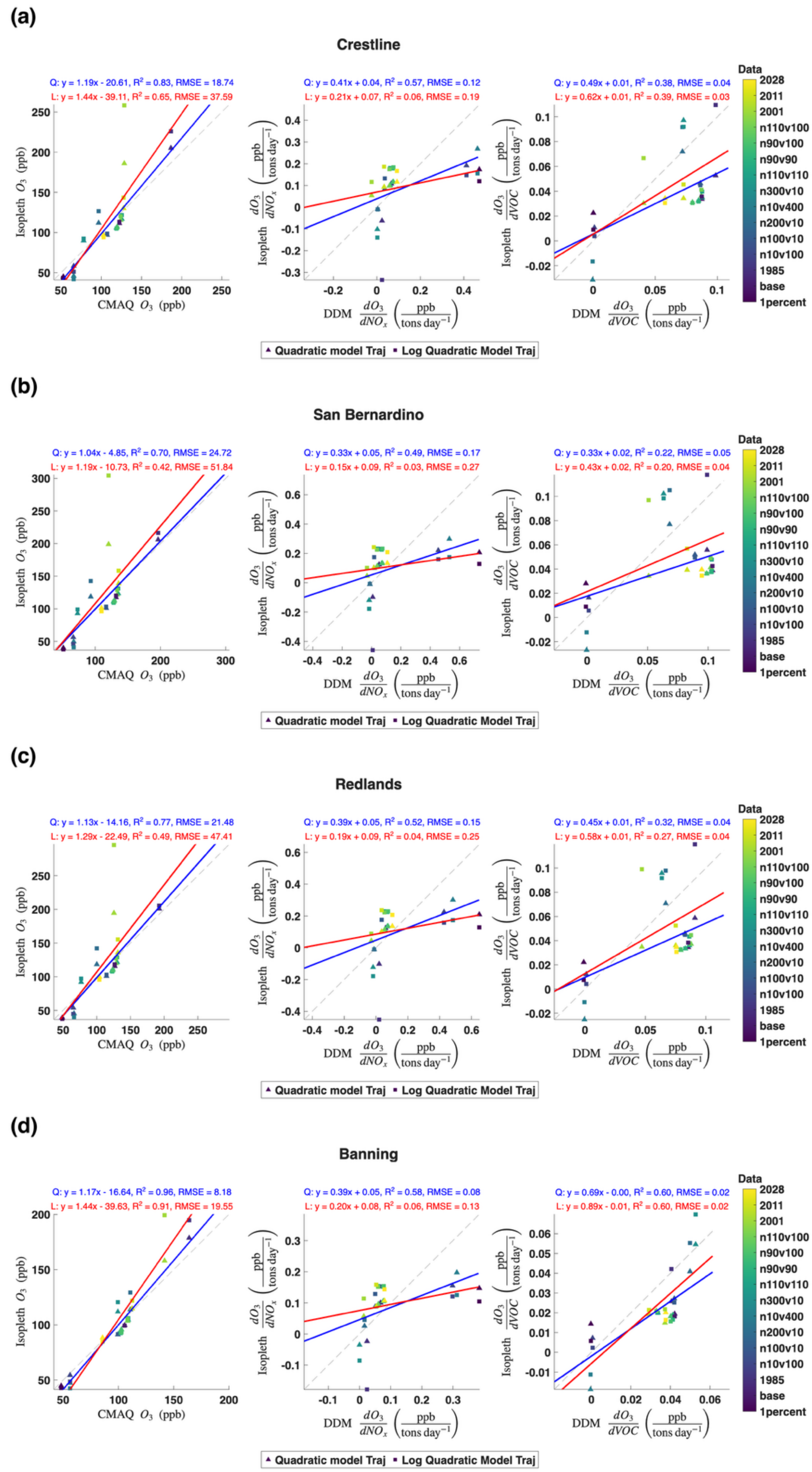
(a)
Crestline
Q: y = 1.19x - 20.61, R² = 0.83, RMSE = 18.74
L: y = 1.44x - 39.11, R² = 0.65, RMSE = 37.59
Q: y = 0.41x + 0.04, R² = 0.57, RMSE = 0.12
L: y = 0.21x + 0.07, R² = 0.06, RMSE = 0.19
Q: y = 0.49x + 0.01, R² = 0.38, RMSE = 0.04
L: y = 0.62x + 0.01, R² = 0.39, RMSE = 0.03
Quadratic model Traj
Log Quadratic Model Traj
(b)
San Bernardino
Q: y = 1.04x - 4.85, R² = 0.70, RMSE = 24.72
L: y = 1.19x - 10.73, R² = 0.42, RMSE = 51.84
Q: y = 0.33x + 0.05, R² = 0.49, RMSE = 0.17
L: y = 0.15x + 0.09, R² = 0.03, RMSE = 0.27
Q: y = 0.33x + 0.02, R² = 0.22, RMSE = 0.05
L: y = 0.43x + 0.02, R² = 0.20, RMSE = 0.04
Quadratic model Traj
Log Quadratic Model Traj
(c)
Redlands
Q: y = 1.13x - 14.16, R² = 0.77, RMSE = 21.48
L: y = 1.29x - 22.49, R² = 0.49, RMSE = 47.41
Q: y = 0.39x + 0.05, R² = 0.52, RMSE = 0.15
L: y = 0.19x + 0.09, R² = 0.04, RMSE = 0.25
Q: y = 0.45x + 0.01, R² = 0.32, RMSE = 0.04
L: y = 0.58x + 0.01, R² = 0.27, RMSE = 0.04
Quadratic model Traj
Log Quadratic Model Traj
(d)
Banning
Q: y = 1.17x - 16.64, R² = 0.96, RMSE = 8.18
L: y = 1.44x - 39.63, R² = 0.91, RMSE = 19.55
Q: y = 0.39x + 0.05, R² = 0.58, RMSE = 0.08
L: y = 0.20x + 0.08, R² = 0.06, RMSE = 0.13
Q: y = 0.69x - 0.00, R² = 0.60, RMSE = 0.02
L: y = 0.89x - 0.01, R² = 0.60, RMSE = 0.02
Quadratic model Traj
Log Quadratic Model Traj
Isopleth O3 (ppb)
CMAQ O3 (ppb)
Isopleth dO3/dNOx (ppb / tons day⁻¹)
DDM dO3/dNOx (ppb / tons day⁻¹)
Isopleth dO3/dVOC (ppb / tons day⁻¹)
DDM dO3/dVOC (ppb / tons day⁻¹)
Data
2028
2011
2001
n110v100
n90v100
n90v90
n110v110
n300v10
n10v400
n200v10
n100v10
n10v100
1985
base
1percent

(e)

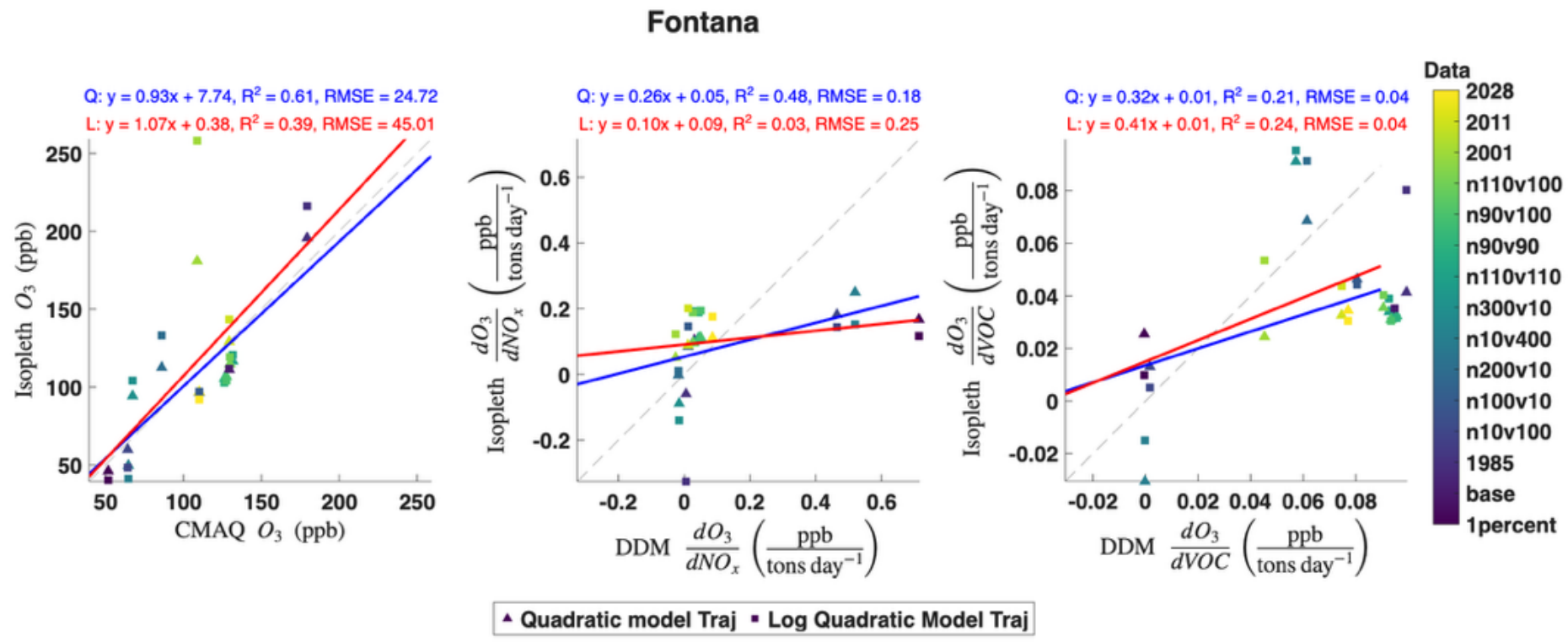
Fontana
Q: y = 0.93x + 7.74, R2 = 0.61, RMSE = 24.72
L: y = 1.07x + 0.38, R2 = 0.39, RMSE = 45.01
Q: y = 0.26x + 0.05, R2 = 0.48, RMSE = 0.18
L: y = 0.10x + 0.09, R2 = 0.03, RMSE = 0.25
Q: y = 0.32x + 0.01, R2 = 0.21, RMSE = 0.04
L: y = 0.41x + 0.01, R2 = 0.24, RMSE = 0.04
Isopleth O3 (ppb)
CMAQ O3 (ppb)
Isopleth dO3/dNOx (ppb / tons day−1)
DDM dO3/dNOx (ppb / tons day−1)
Isopleth dO3/dVOC (ppb / tons day−1)
DDM dO3/dVOC (ppb / tons day−1)
Data
2028
2011
2001
n110v100
n90v100
n90v90
n110v110
n300v10
n10v400
n200v10
n100v10
n10v100
1985
base
1percent
Quadratic model Traj
Log Quadratic Model Traj

(f)

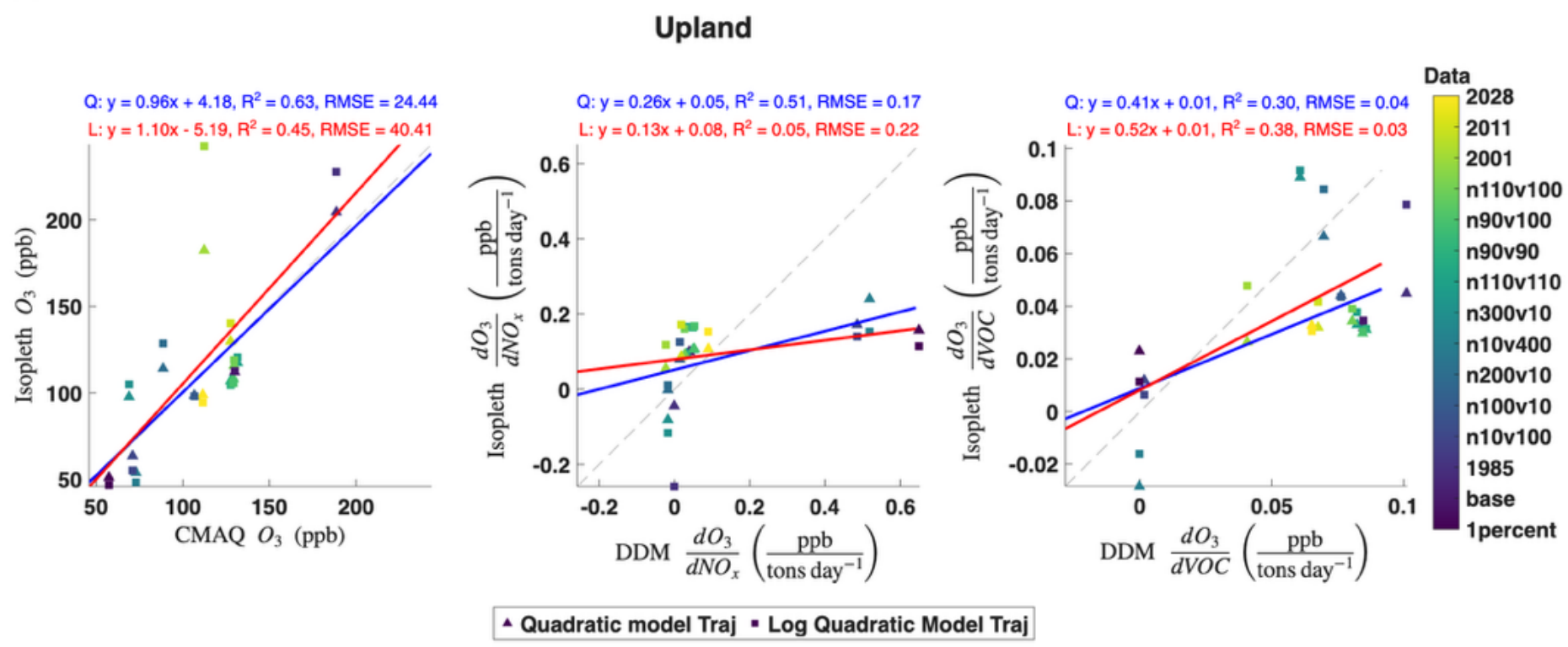
Upland
Q: y = 0.96x + 4.18, R2 = 0.63, RMSE = 24.44
L: y = 1.10x - 5.19, R2 = 0.45, RMSE = 40.41
Q: y = 0.26x + 0.05, R2 = 0.51, RMSE = 0.17
L: y = 0.13x + 0.08, R2 = 0.05, RMSE = 0.22
Q: y = 0.41x + 0.01, R2 = 0.30, RMSE = 0.04
L: y = 0.52x + 0.01, R2 = 0.38, RMSE = 0.03
Isopleth O3 (ppb)
CMAQ O3 (ppb)
Isopleth dO3/dNOx (ppb / tons day−1)
DDM dO3/dNOx (ppb / tons day−1)
Isopleth dO3/dVOC (ppb / tons day−1)
DDM dO3/dVOC (ppb / tons day−1)
Data
2028
2011
2001
n110v100
n90v100
n90v90
n110v110
n300v10
n10v400
n200v10
n100v10
n10v100
1985
base
1percent
Quadratic model Traj
Log Quadratic Model Traj

(g)

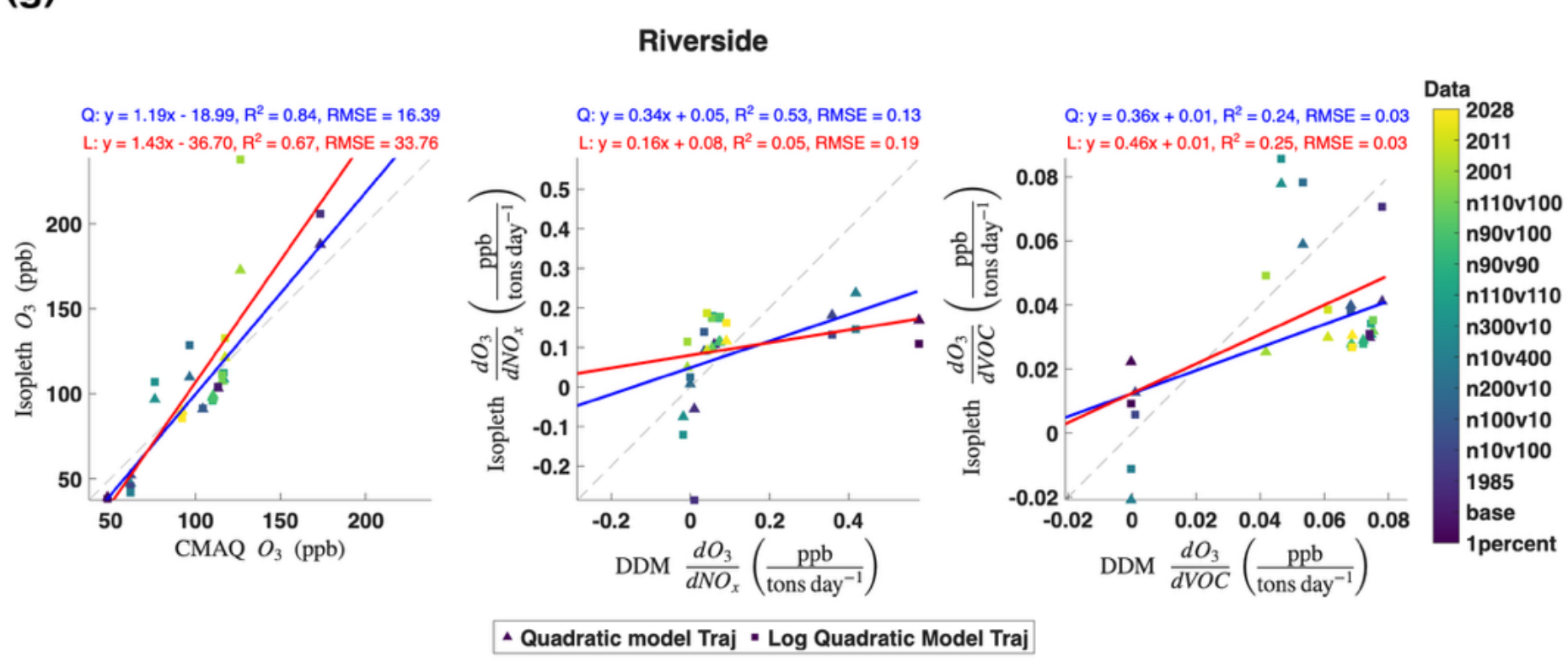
Riverside
Q: y = 1.19x - 18.99, R2 = 0.84, RMSE = 16.39
L: y = 1.43x - 36.70, R2 = 0.67, RMSE = 33.76
Q: y = 0.34x + 0.05, R2 = 0.53, RMSE = 0.13
L: y = 0.16x + 0.08, R2 = 0.05, RMSE = 0.19
Q: y = 0.36x + 0.01, R2 = 0.24, RMSE = 0.03
L: y = 0.46x + 0.01, R2 = 0.25, RMSE = 0.03
Isopleth O3 (ppb)
CMAQ O3 (ppb)
Isopleth dO3/dNOx (ppb / tons day−1)
DDM dO3/dNOx (ppb / tons day−1)
Isopleth dO3/dVOC (ppb / tons day−1)
DDM dO3/dVOC (ppb / tons day−1)
Data
2028
2011
2001
n110v100
n90v100
n90v90
n110v110
n300v10
n10v400
n200v10
n100v10
n10v100
1985
base
1percent
Quadratic model Traj
Log Quadratic Model Traj

(h)

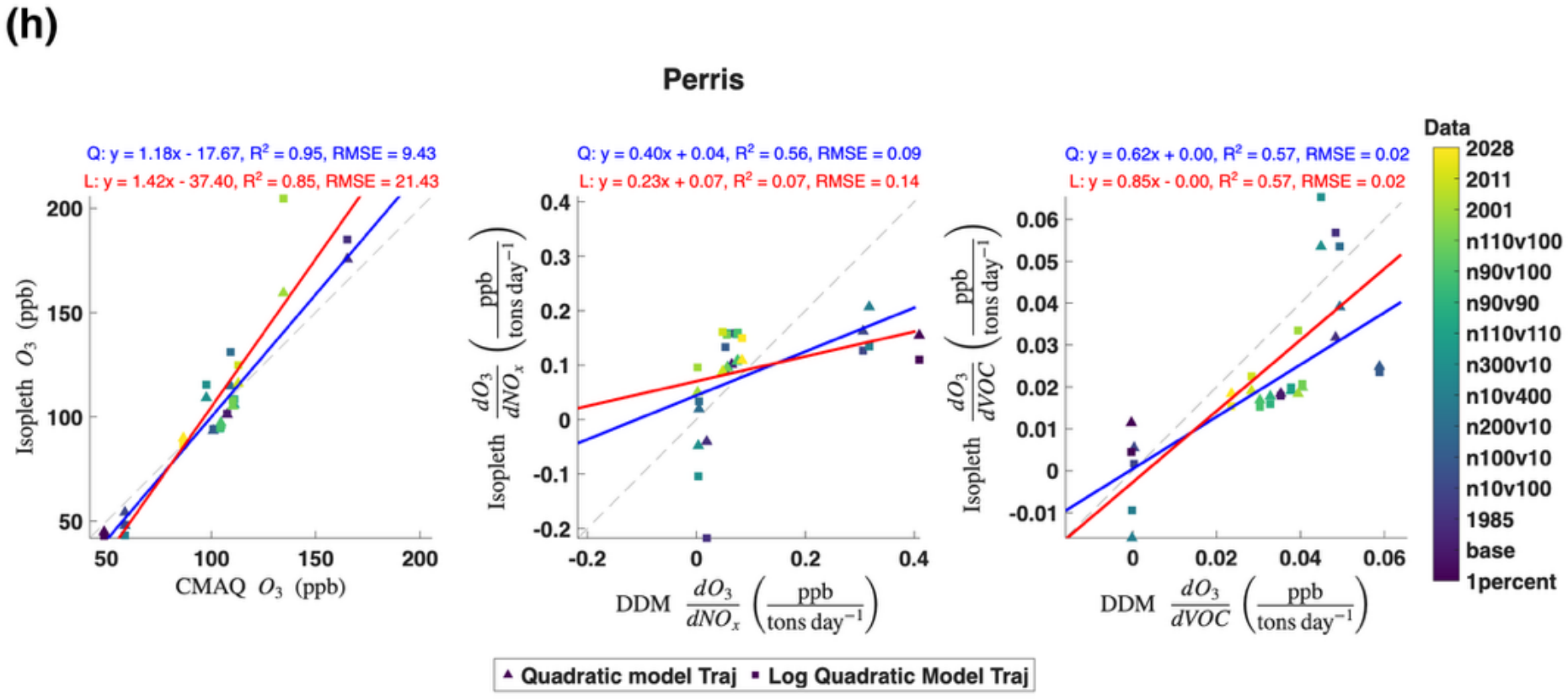
Perris
Q: y = 1.18x - 17.67, R2 = 0.95, RMSE = 9.43
L: y = 1.42x - 37.40, R2 = 0.85, RMSE = 21.43
Q: y = 0.40x + 0.04, R2 = 0.56, RMSE = 0.09
L: y = 0.23x + 0.07, R2 = 0.07, RMSE = 0.14
Q: y = 0.62x + 0.00, R2 = 0.57, RMSE = 0.02
L: y = 0.85x - 0.00, R2 = 0.57, RMSE = 0.02
Isopleth O3 (ppb)
CMAQ O3 (ppb)
Isopleth dO3/dNOx (ppb / tons day−1)
DDM dO3/dNOx (ppb / tons day−1)
Isopleth dO3/dVOC (ppb / tons day−1)
DDM dO3/dVOC (ppb / tons day−1)
Data
2028
2011
2001
n110v100
n90v100
n90v90
n110v110
n300v10
n10v400
n200v10
n100v10
n10v100
1985
base
1percent
Quadratic model Traj
Log Quadratic Model Traj

(i)

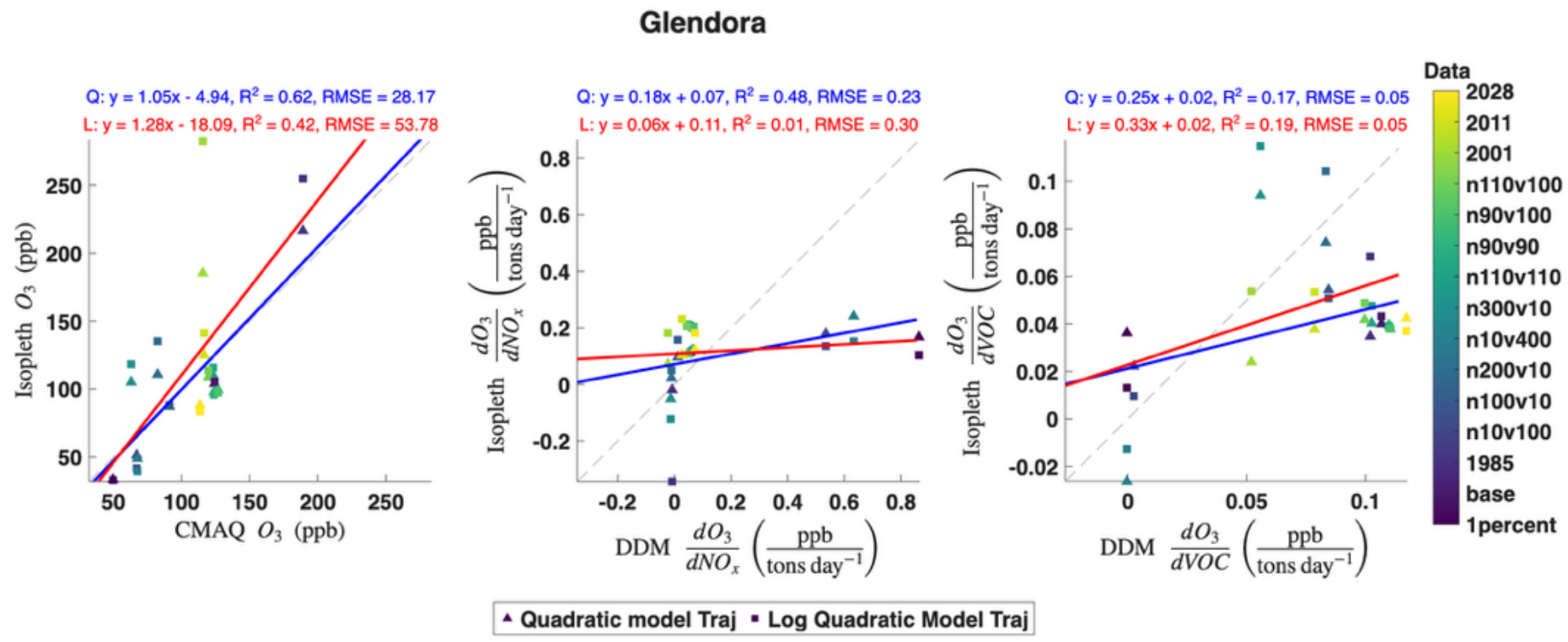
Glendora
Q: y = 1.05x - 4.94, R² = 0.62, RMSE = 28.17
L: y = 1.28x - 18.09, R² = 0.42, RMSE = 53.78
Q: y = 0.18x + 0.07, R² = 0.48, RMSE = 0.23
L: y = 0.06x + 0.11, R² = 0.01, RMSE = 0.30
Q: y = 0.25x + 0.02, R² = 0.17, RMSE = 0.05
L: y = 0.33x + 0.02, R² = 0.19, RMSE = 0.05
Isopleth O3 (ppb)
CMAQ O3 (ppb)
Isopleth dO3/dNOx (ppb/tons day⁻¹)
DDM dO3/dNOx (ppb/tons day⁻¹)
Isopleth dO3/dVOC (ppb/tons day⁻¹)
DDM dO3/dVOC (ppb/tons day⁻¹)
Data
2028
2011
2001
n110v100
n90v100
n90v90
n110v110
n300v10
n10v400
n200v10
n100v10
n10v100
1985
base
1percent
Quadratic model Traj
Log Quadratic Model Traj

(j)

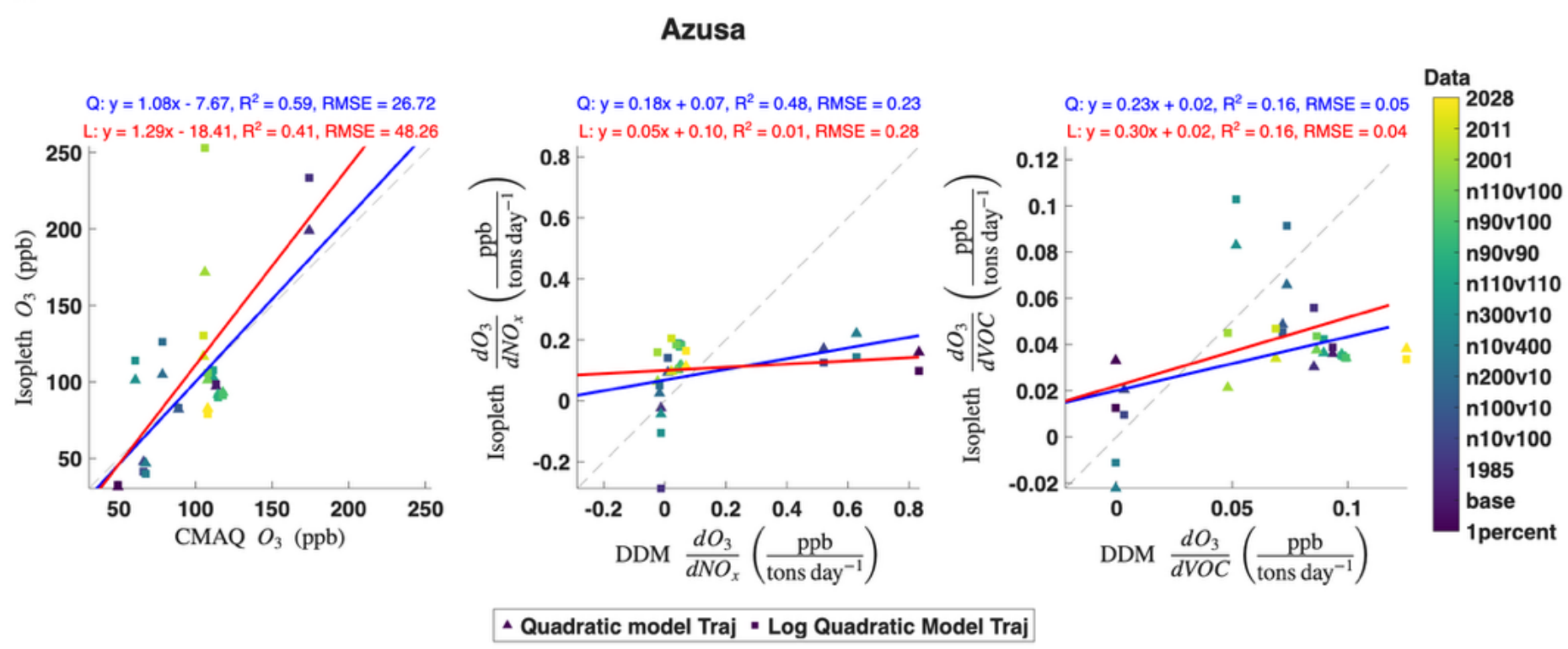
Azusa
Q: y = 1.08x - 7.67, R² = 0.59, RMSE = 26.72
L: y = 1.29x - 18.41, R² = 0.41, RMSE = 48.26
Q: y = 0.18x + 0.07, R² = 0.48, RMSE = 0.23
L: y = 0.05x + 0.10, R² = 0.01, RMSE = 0.28
Q: y = 0.23x + 0.02, R² = 0.16, RMSE = 0.05
L: y = 0.30x + 0.02, R² = 0.16, RMSE = 0.04
Isopleth O3 (ppb)
CMAQ O3 (ppb)
Isopleth dO3/dNOx (ppb/tons day⁻¹)
DDM dO3/dNOx (ppb/tons day⁻¹)
Isopleth dO3/dVOC (ppb/tons day⁻¹)
DDM dO3/dVOC (ppb/tons day⁻¹)
Data
2028
2011
2001
n110v100
n90v100
n90v90
n110v110
n300v10
n10v400
n200v10
n100v10
n10v100
1985
base
1percent
Quadratic model Traj
Log Quadratic Model Traj

(k)

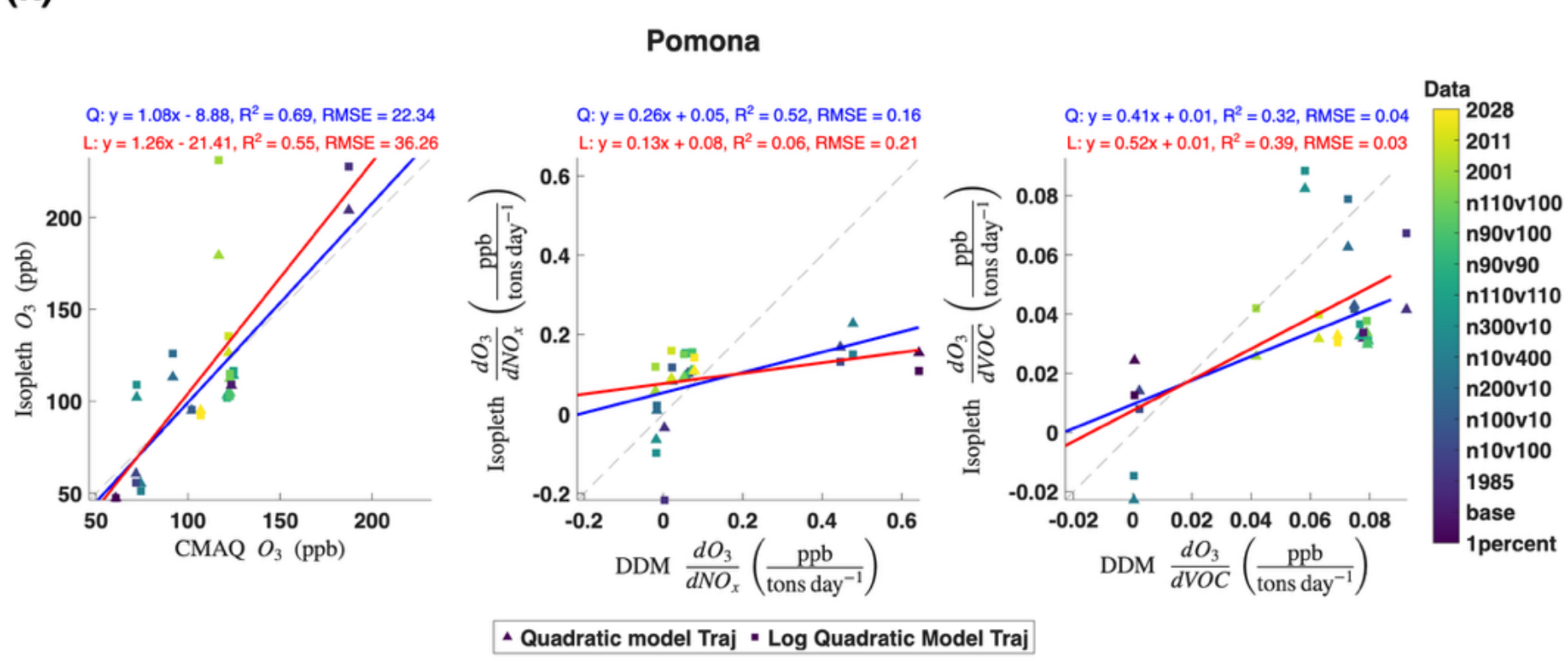
Pomona
Q: y = 1.08x - 8.88, R² = 0.69, RMSE = 22.34
L: y = 1.26x - 21.41, R² = 0.55, RMSE = 36.26
Q: y = 0.26x + 0.05, R² = 0.52, RMSE = 0.16
L: y = 0.13x + 0.08, R² = 0.06, RMSE = 0.21
Q: y = 0.41x + 0.01, R² = 0.32, RMSE = 0.04
L: y = 0.52x + 0.01, R² = 0.39, RMSE = 0.03
Isopleth O3 (ppb)
CMAQ O3 (ppb)
Isopleth dO3/dNOx (ppb/tons day⁻¹)
DDM dO3/dNOx (ppb/tons day⁻¹)
Isopleth dO3/dVOC (ppb/tons day⁻¹)
DDM dO3/dVOC (ppb/tons day⁻¹)
Data
2028
2011
2001
n110v100
n90v100
n90v90
n110v110
n300v10
n10v400
n200v10
n100v10
n10v100
1985
base
1percent
Quadratic model Traj
Log Quadratic Model Traj

(l)

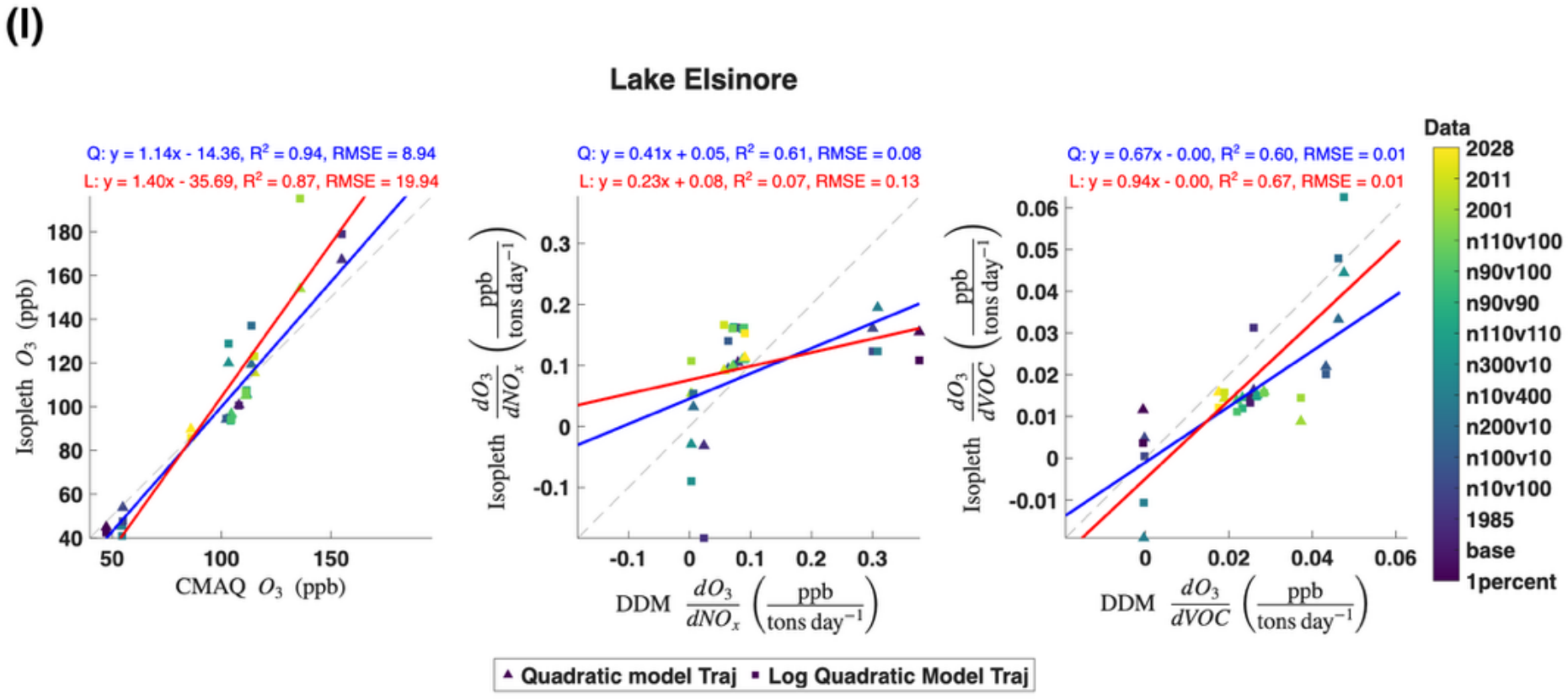
Lake Elsinore
Q: y = 1.14x - 14.36, R² = 0.94, RMSE = 8.94
L: y = 1.40x - 35.69, R² = 0.87, RMSE = 19.94
Q: y = 0.41x + 0.05, R² = 0.61, RMSE = 0.08
L: y = 0.23x + 0.08, R² = 0.07, RMSE = 0.13
Q: y = 0.67x - 0.00, R² = 0.60, RMSE = 0.01
L: y = 0.94x - 0.00, R² = 0.67, RMSE = 0.01
Isopleth O3 (ppb)
CMAQ O3 (ppb)
Isopleth dO3/dNOx (ppb/tons day⁻¹)
DDM dO3/dNOx (ppb/tons day⁻¹)
Isopleth dO3/dVOC (ppb/tons day⁻¹)
DDM dO3/dVOC (ppb/tons day⁻¹)
Data
2028
2011
2001
n110v100
n90v100
n90v90
n110v110
n300v10
n10v400
n200v10
n100v10
n10v100
1985
base
1percent
Quadratic model Traj
Log Quadratic Model Traj

(m)

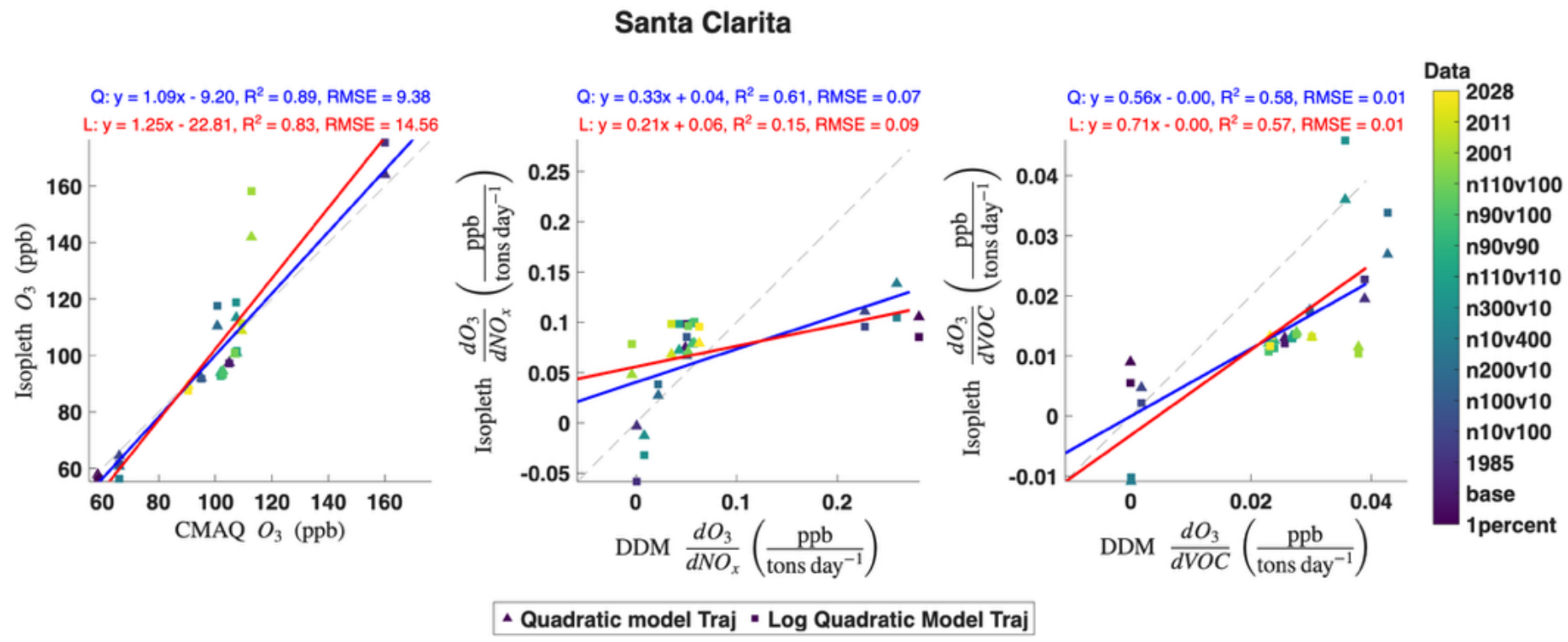
Santa Clarita
Q: y = 1.09x - 9.20, R² = 0.89, RMSE = 9.38
L: y = 1.25x - 22.81, R² = 0.83, RMSE = 14.56
Q: y = 0.33x + 0.04, R² = 0.61, RMSE = 0.07
L: y = 0.21x + 0.06, R² = 0.15, RMSE = 0.09
Q: y = 0.56x - 0.00, R² = 0.58, RMSE = 0.01
L: y = 0.71x - 0.00, R² = 0.57, RMSE = 0.01
Isopleth O3 (ppb)
CMAQ O3 (ppb)
Isopleth dO3/dNOx (ppb/tons day-1)
DDM dO3/dNOx (ppb/tons day-1)
Isopleth dO3/dVOC (ppb/tons day-1)
DDM dO3/dVOC (ppb/tons day-1)
Data
2028
2011
2001
n110v100
n90v100
n90v90
n110v110
n300v10
n10v400
n200v10
n100v10
n10v100
1985
base
1percent
Quadratic model Traj
Log Quadratic Model Traj

(n)

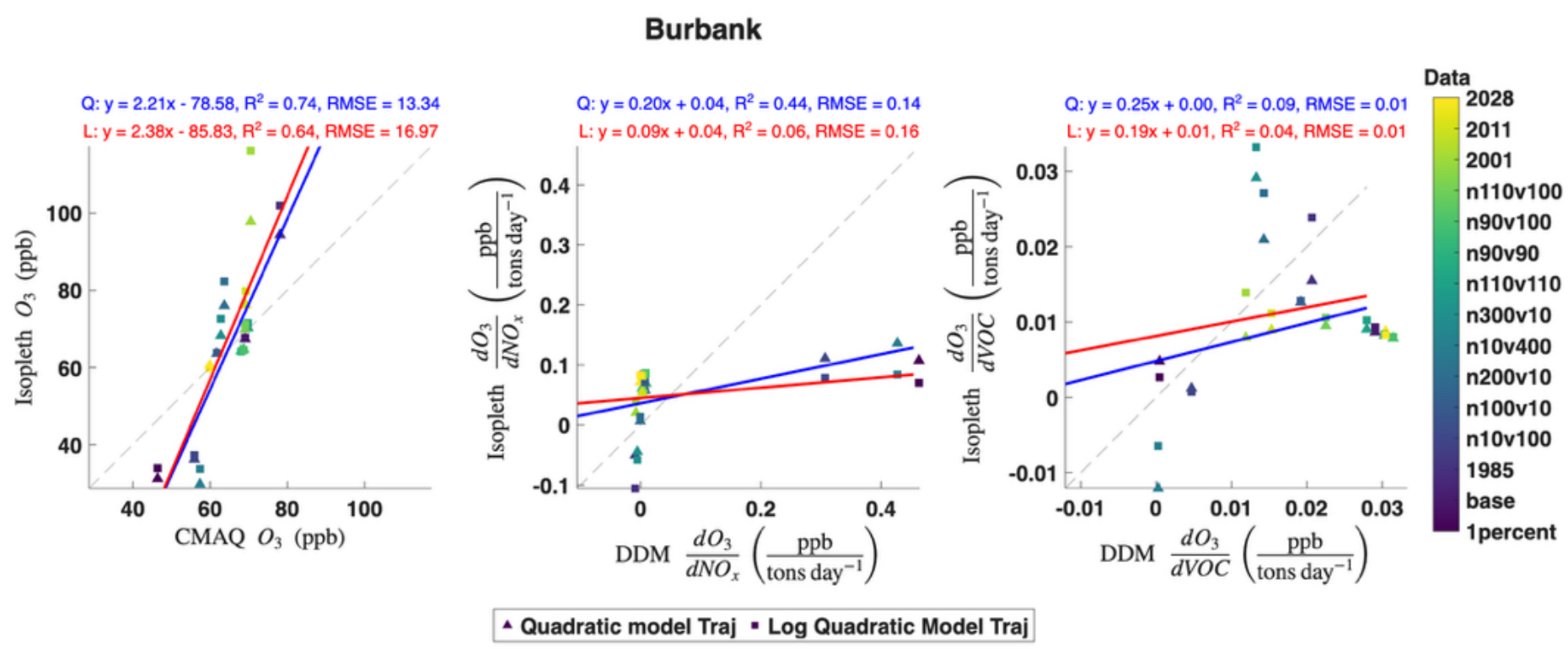
Burbank
Q: y = 2.21x - 78.58, R² = 0.74, RMSE = 13.34
L: y = 2.38x - 85.83, R² = 0.64, RMSE = 16.97
Q: y = 0.20x + 0.04, R² = 0.44, RMSE = 0.14
L: y = 0.09x + 0.04, R² = 0.06, RMSE = 0.16
Q: y = 0.25x + 0.00, R² = 0.09, RMSE = 0.01
L: y = 0.19x + 0.01, R² = 0.04, RMSE = 0.01
Isopleth O3 (ppb)
CMAQ O3 (ppb)
Isopleth dO3/dNOx (ppb/tons day-1)
DDM dO3/dNOx (ppb/tons day-1)
Isopleth dO3/dVOC (ppb/tons day-1)
DDM dO3/dVOC (ppb/tons day-1)
Data
2028
2011
2001
n110v100
n90v100
n90v90
n110v110
n300v10
n10v400
n200v10
n100v10
n10v100
1985
base
1percent
Quadratic model Traj
Log Quadratic Model Traj

(o)

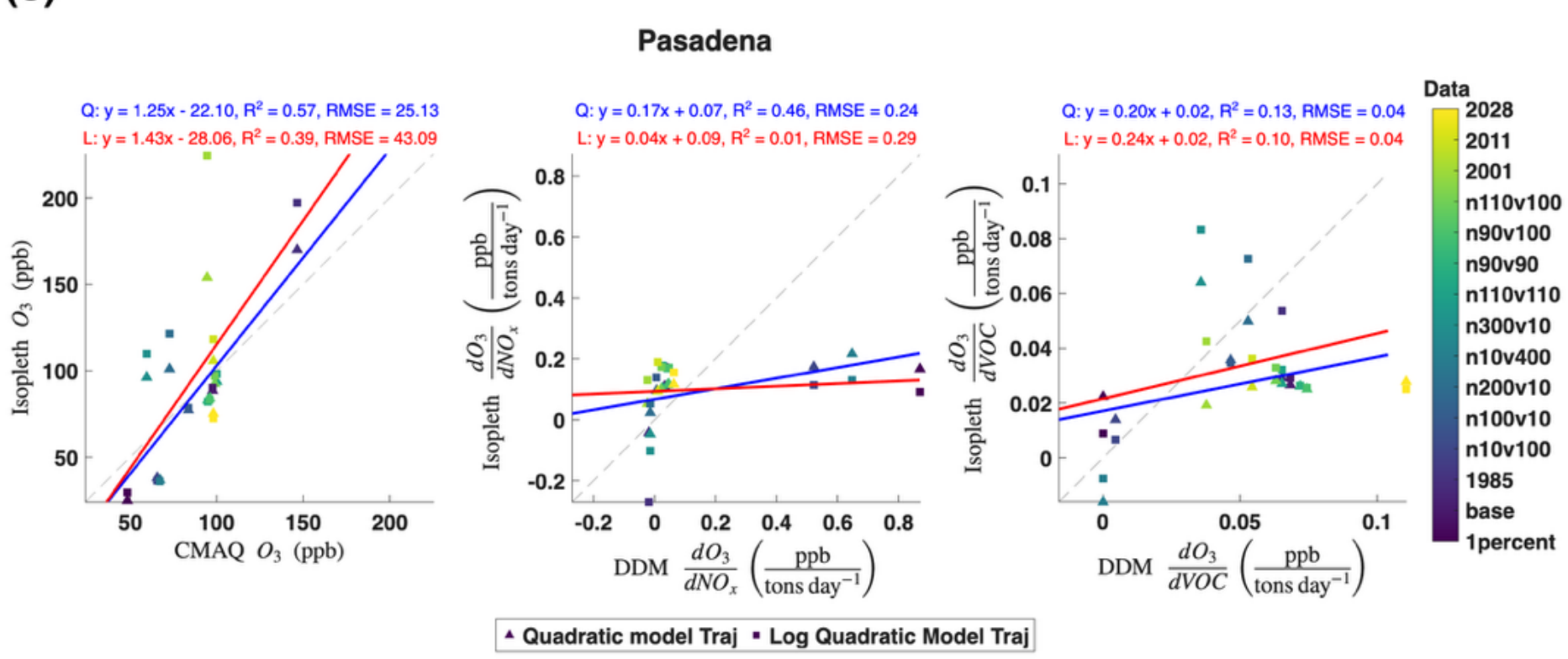
Pasadena
Q: y = 1.25x - 22.10, R² = 0.57, RMSE = 25.13
L: y = 1.43x - 28.06, R² = 0.39, RMSE = 43.09
Q: y = 0.17x + 0.07, R² = 0.46, RMSE = 0.24
L: y = 0.04x + 0.09, R² = 0.01, RMSE = 0.29
Q: y = 0.20x + 0.02, R² = 0.13, RMSE = 0.04
L: y = 0.24x + 0.02, R² = 0.10, RMSE = 0.04
Isopleth O3 (ppb)
CMAQ O3 (ppb)
Isopleth dO3/dNOx (ppb/tons day-1)
DDM dO3/dNOx (ppb/tons day-1)
Isopleth dO3/dVOC (ppb/tons day-1)
DDM dO3/dVOC (ppb/tons day-1)
Data
2028
2011
2001
n110v100
n90v100
n90v90
n110v110
n300v10
n10v400
n200v10
n100v10
n10v100
1985
base
1percent
Quadratic model Traj
Log Quadratic Model Traj

(p)

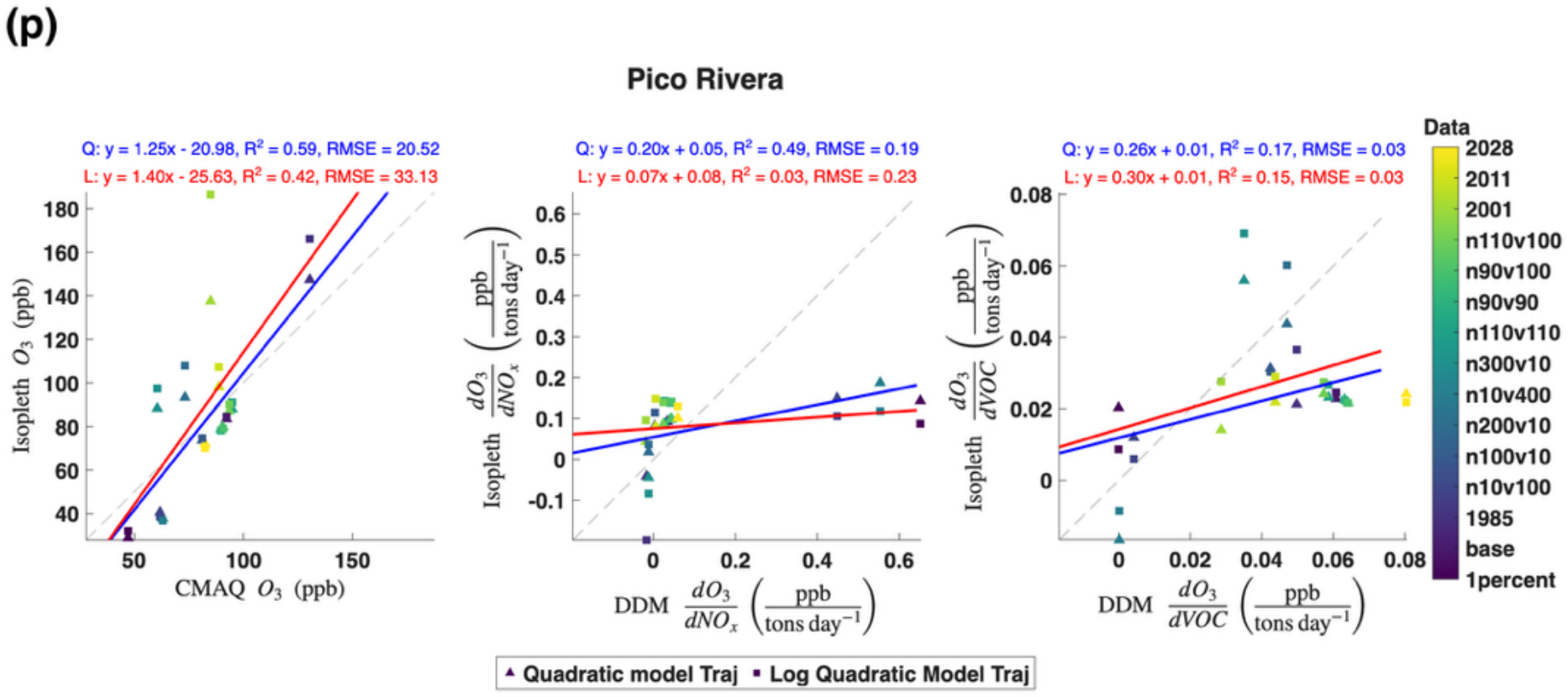
Pico Rivera
Q: y = 1.25x - 20.98, R² = 0.59, RMSE = 20.52
L: y = 1.40x - 25.63, R² = 0.42, RMSE = 33.13
Q: y = 0.20x + 0.05, R² = 0.49, RMSE = 0.19
L: y = 0.07x + 0.08, R² = 0.03, RMSE = 0.23
Q: y = 0.26x + 0.01, R² = 0.17, RMSE = 0.03
L: y = 0.30x + 0.01, R² = 0.15, RMSE = 0.03
Isopleth O3 (ppb)
CMAQ O3 (ppb)
Isopleth dO3/dNOx (ppb/tons day-1)
DDM dO3/dNOx (ppb/tons day-1)
Isopleth dO3/dVOC (ppb/tons day-1)
DDM dO3/dVOC (ppb/tons day-1)
Data
2028
2011
2001
n110v100
n90v100
n90v90
n110v110
n300v10
n10v400
n200v10
n100v10
n10v100
1985
base
1percent
Quadratic model Traj
Log Quadratic Model Traj

(q)

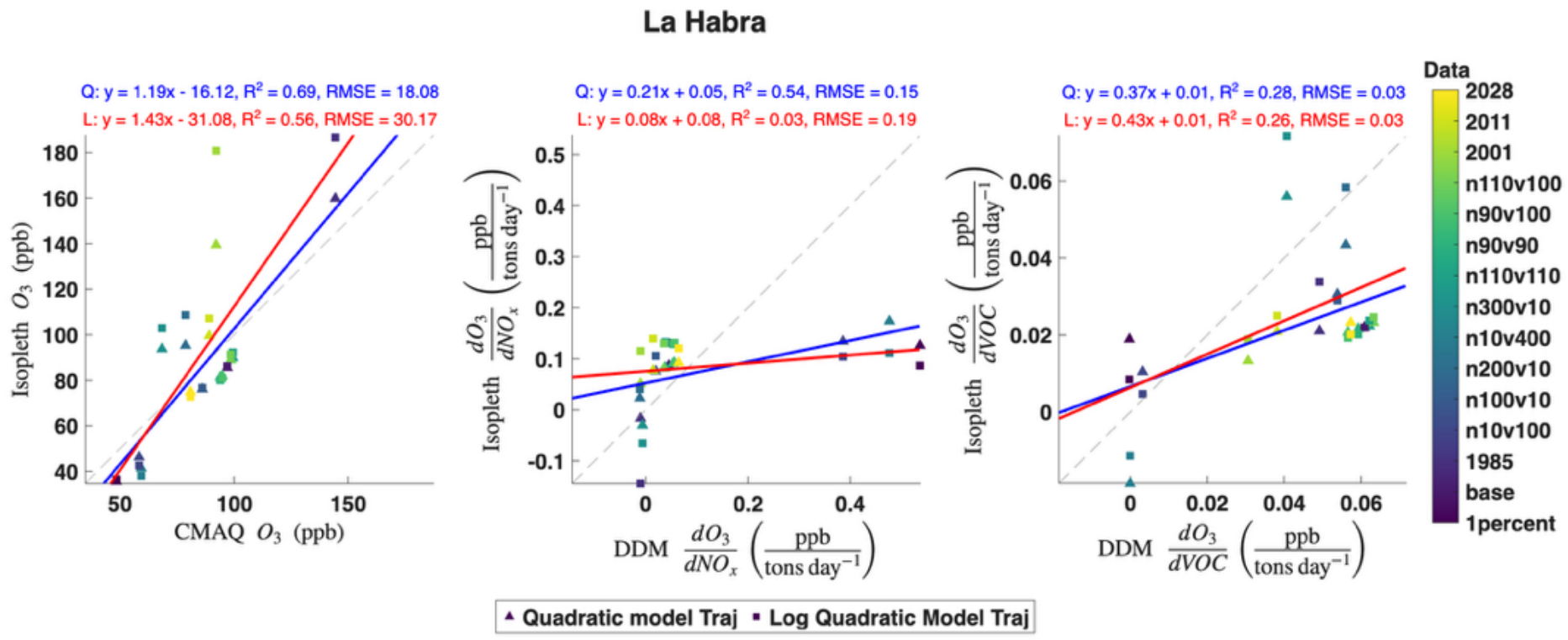
La Habra
Q: y = 1.19x - 16.12, R² = 0.69, RMSE = 18.08
L: y = 1.43x - 31.08, R² = 0.56, RMSE = 30.17
Isopleth O₃ (ppb)
CMAQ O₃ (ppb)
Q: y = 0.21x + 0.05, R² = 0.54, RMSE = 0.15
L: y = 0.08x + 0.08, R² = 0.03, RMSE = 0.19
Isopleth dO₃/dNOₓ (ppb/tons day⁻¹)
DDM dO₃/dNOₓ (ppb/tons day⁻¹)
Q: y = 0.37x + 0.01, R² = 0.28, RMSE = 0.03
L: y = 0.43x + 0.01, R² = 0.26, RMSE = 0.03
Isopleth dO₃/dVOC (ppb/tons day⁻¹)
DDM dO₃/dVOC (ppb/tons day⁻¹)
Data
2028
2011
2001
n110v100
n90v100
n90v90
n110v110
n300v10
n10v400
n200v10
n100v10
n10v100
1985
base
1percent
Quadratic model Traj
Log Quadratic Model Traj

(r)

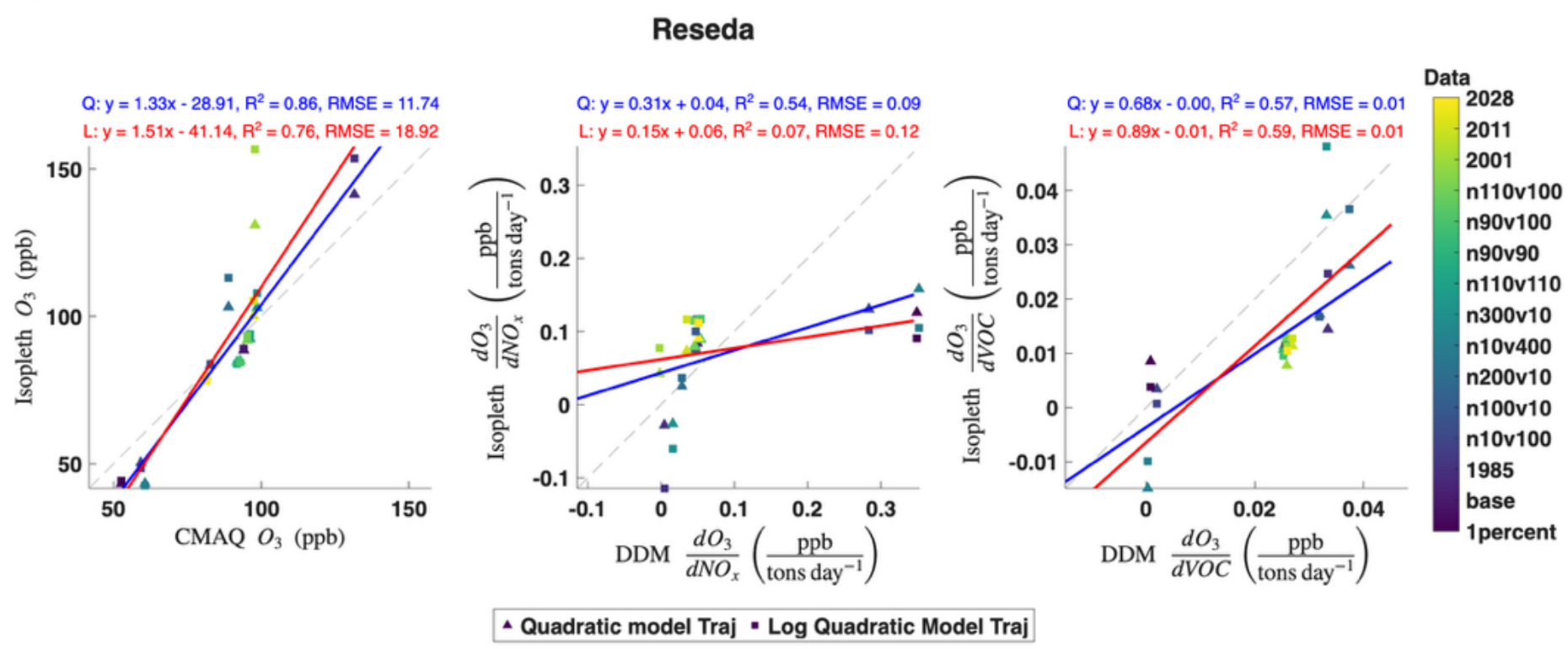
Reseda
Q: y = 1.33x - 28.91, R² = 0.86, RMSE = 11.74
L: y = 1.51x - 41.14, R² = 0.76, RMSE = 18.92
Isopleth O₃ (ppb)
CMAQ O₃ (ppb)
Q: y = 0.31x + 0.04, R² = 0.54, RMSE = 0.09
L: y = 0.15x + 0.06, R² = 0.07, RMSE = 0.12
Isopleth dO₃/dNOₓ (ppb/tons day⁻¹)
DDM dO₃/dNOₓ (ppb/tons day⁻¹)
Q: y = 0.68x - 0.00, R² = 0.57, RMSE = 0.01
L: y = 0.89x - 0.01, R² = 0.59, RMSE = 0.01
Isopleth dO₃/dVOC (ppb/tons day⁻¹)
DDM dO₃/dVOC (ppb/tons day⁻¹)
Data
2028
2011
2001
n110v100
n90v100
n90v90
n110v110
n300v10
n10v400
n200v10
n100v10
n10v100
1985
base
1percent
Quadratic model Traj
Log Quadratic Model Traj

(s)

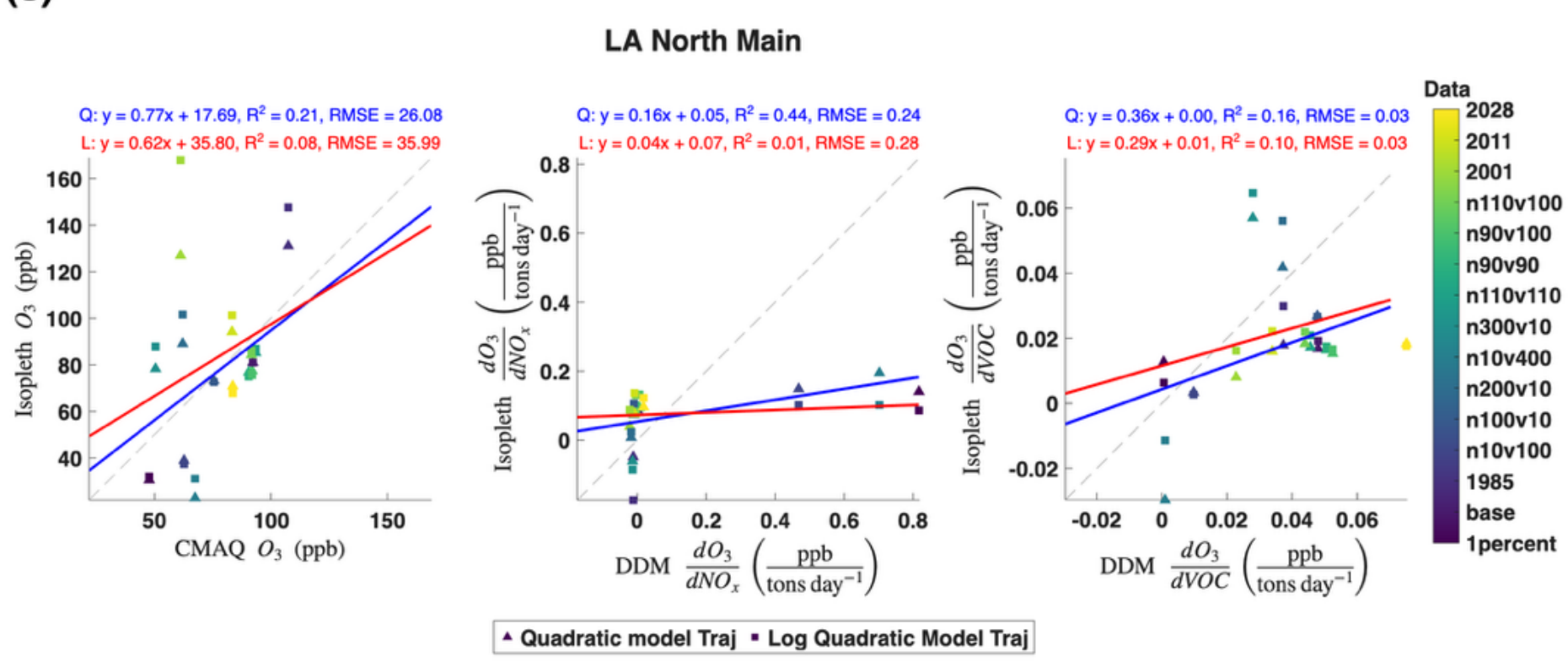
LA North Main
Q: y = 0.77x + 17.69, R² = 0.21, RMSE = 26.08
L: y = 0.62x + 35.80, R² = 0.08, RMSE = 35.99
Isopleth O₃ (ppb)
CMAQ O₃ (ppb)
Q: y = 0.16x + 0.05, R² = 0.44, RMSE = 0.24
L: y = 0.04x + 0.07, R² = 0.01, RMSE = 0.28
Isopleth dO₃/dNOₓ (ppb/tons day⁻¹)
DDM dO₃/dNOₓ (ppb/tons day⁻¹)
Q: y = 0.36x + 0.00, R² = 0.16, RMSE = 0.03
L: y = 0.29x + 0.01, R² = 0.10, RMSE = 0.03
Isopleth dO₃/dVOC (ppb/tons day⁻¹)
DDM dO₃/dVOC (ppb/tons day⁻¹)
Data
2028
2011
2001
n110v100
n90v100
n90v90
n110v110
n300v10
n10v400
n200v10
n100v10
n10v100
1985
base
1percent
Quadratic model Traj
Log Quadratic Model Traj

(t)

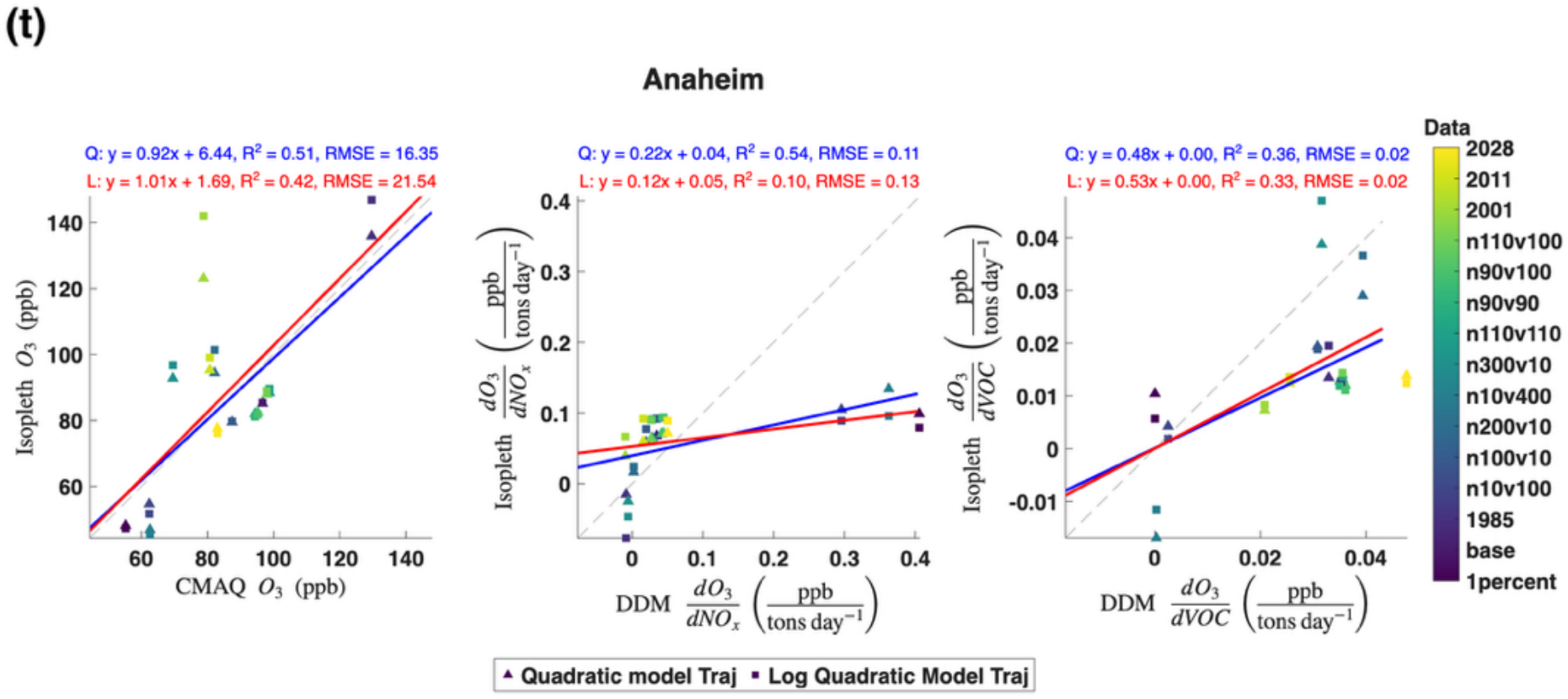
Anaheim
Q: y = 0.92x + 6.44, R² = 0.51, RMSE = 16.35
L: y = 1.01x + 1.69, R² = 0.42, RMSE = 21.54
Isopleth O₃ (ppb)
CMAQ O₃ (ppb)
Q: y = 0.22x + 0.04, R² = 0.54, RMSE = 0.11
L: y = 0.12x + 0.05, R² = 0.10, RMSE = 0.13
Isopleth dO₃/dNOₓ (ppb/tons day⁻¹)
DDM dO₃/dNOₓ (ppb/tons day⁻¹)
Q: y = 0.48x + 0.00, R² = 0.36, RMSE = 0.02
L: y = 0.53x + 0.00, R² = 0.33, RMSE = 0.02
Isopleth dO₃/dVOC (ppb/tons day⁻¹)
DDM dO₃/dVOC (ppb/tons day⁻¹)
Data
2028
2011
2001
n110v100
n90v100
n90v90
n110v110
n300v10
n10v400
n200v10
n100v10
n10v100
1985
base
1percent
Quadratic model Traj
Log Quadratic Model Traj

(u)

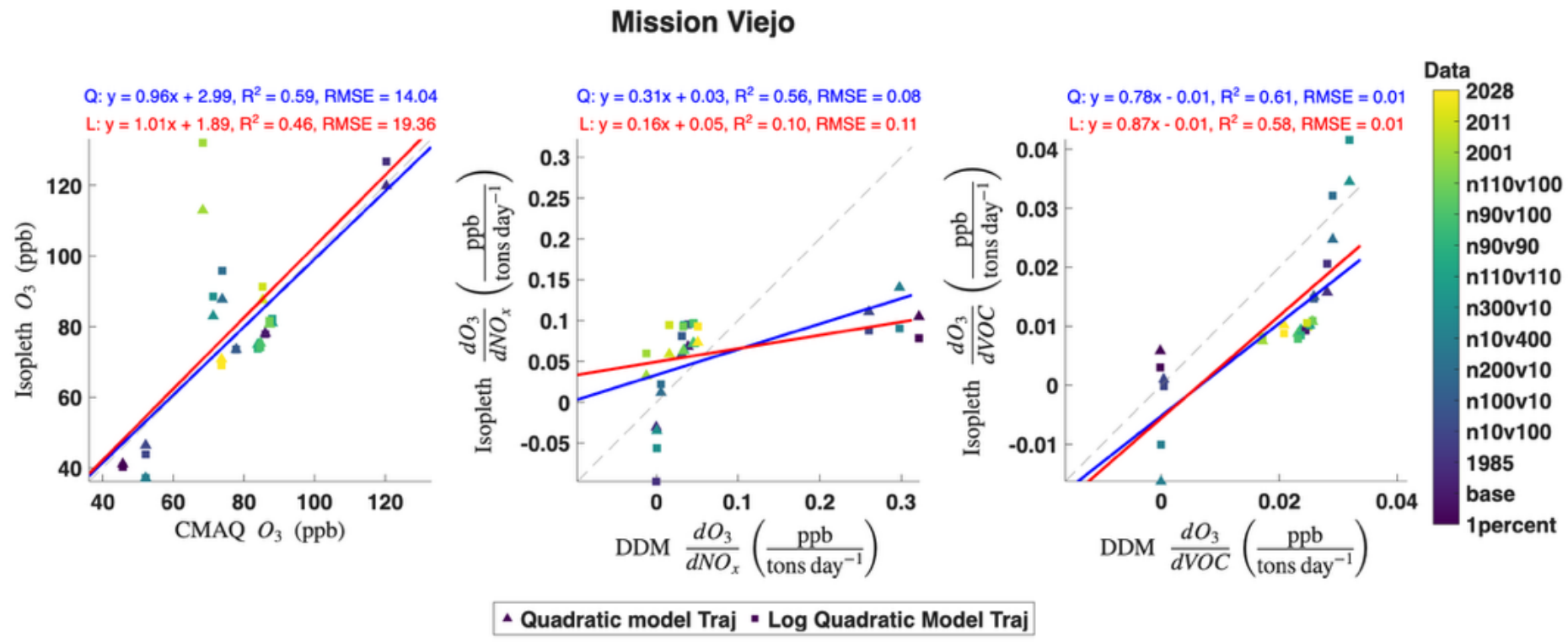
Mission Viejo
Q: y = 0.96x + 2.99, R2 = 0.59, RMSE = 14.04
L: y = 1.01x + 1.89, R2 = 0.46, RMSE = 19.36
Q: y = 0.31x + 0.03, R2 = 0.56, RMSE = 0.08
L: y = 0.16x + 0.05, R2 = 0.10, RMSE = 0.11
Q: y = 0.78x - 0.01, R2 = 0.61, RMSE = 0.01
L: y = 0.87x - 0.01, R2 = 0.58, RMSE = 0.01
Isopleth O3 (ppb)
CMAQ O3 (ppb)
DDM dO3/dNOx (ppb/tons day−1)
DDM dO3/dVOC (ppb/tons day−1)
Data
2028
2011
2001
n110v100
n90v100
n90v90
n110v110
n300v10
n10v400
n200v10
n100v10
n10v100
1985
base
1percent
Quadratic model Traj
Log Quadratic Model Traj

(v)

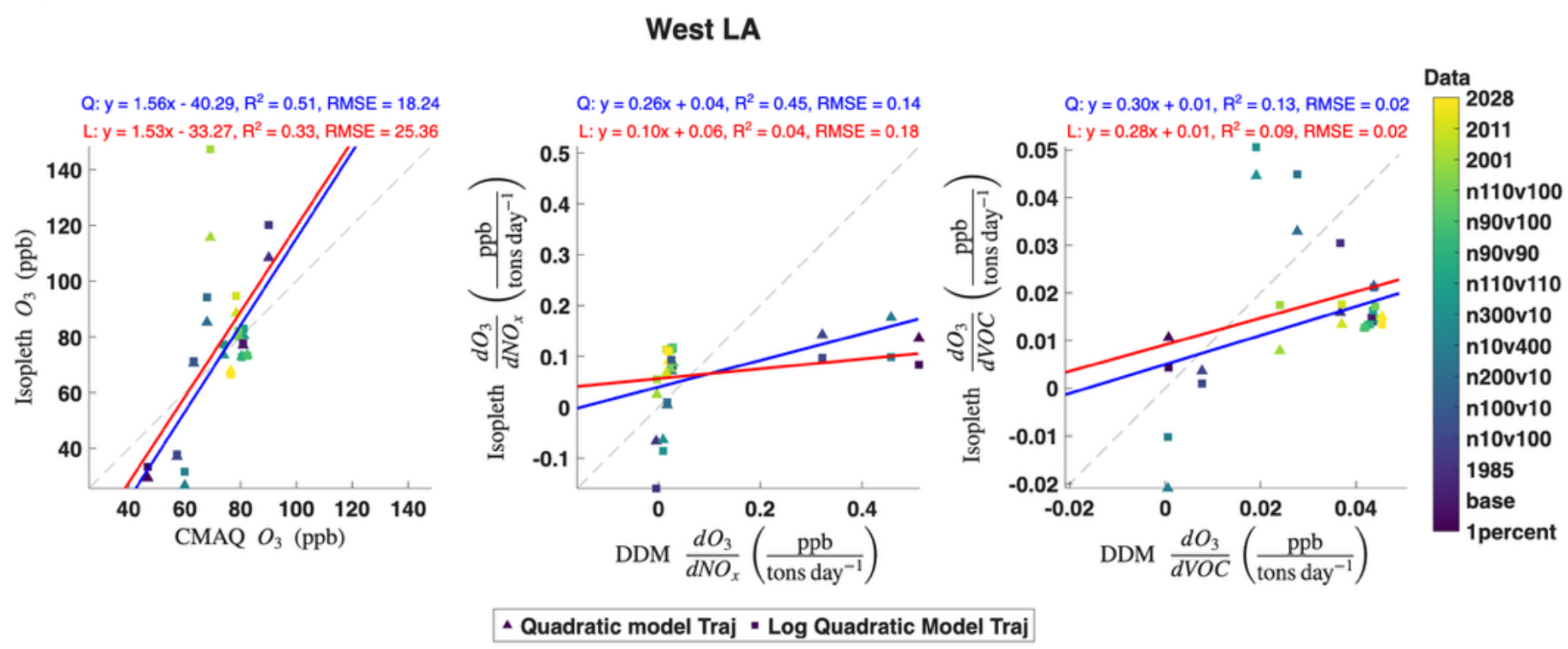
West LA
Q: y = 1.56x - 40.29, R2 = 0.51, RMSE = 18.24
L: y = 1.53x - 33.27, R2 = 0.33, RMSE = 25.36
Q: y = 0.26x + 0.04, R2 = 0.45, RMSE = 0.14
L: y = 0.10x + 0.06, R2 = 0.04, RMSE = 0.18
Q: y = 0.30x + 0.01, R2 = 0.13, RMSE = 0.02
L: y = 0.28x + 0.01, R2 = 0.09, RMSE = 0.02
Isopleth O3 (ppb)
CMAQ O3 (ppb)
DDM dO3/dNOx (ppb/tons day−1)
DDM dO3/dVOC (ppb/tons day−1)
Data
Quadratic model Traj
Log Quadratic Model Traj

(w)

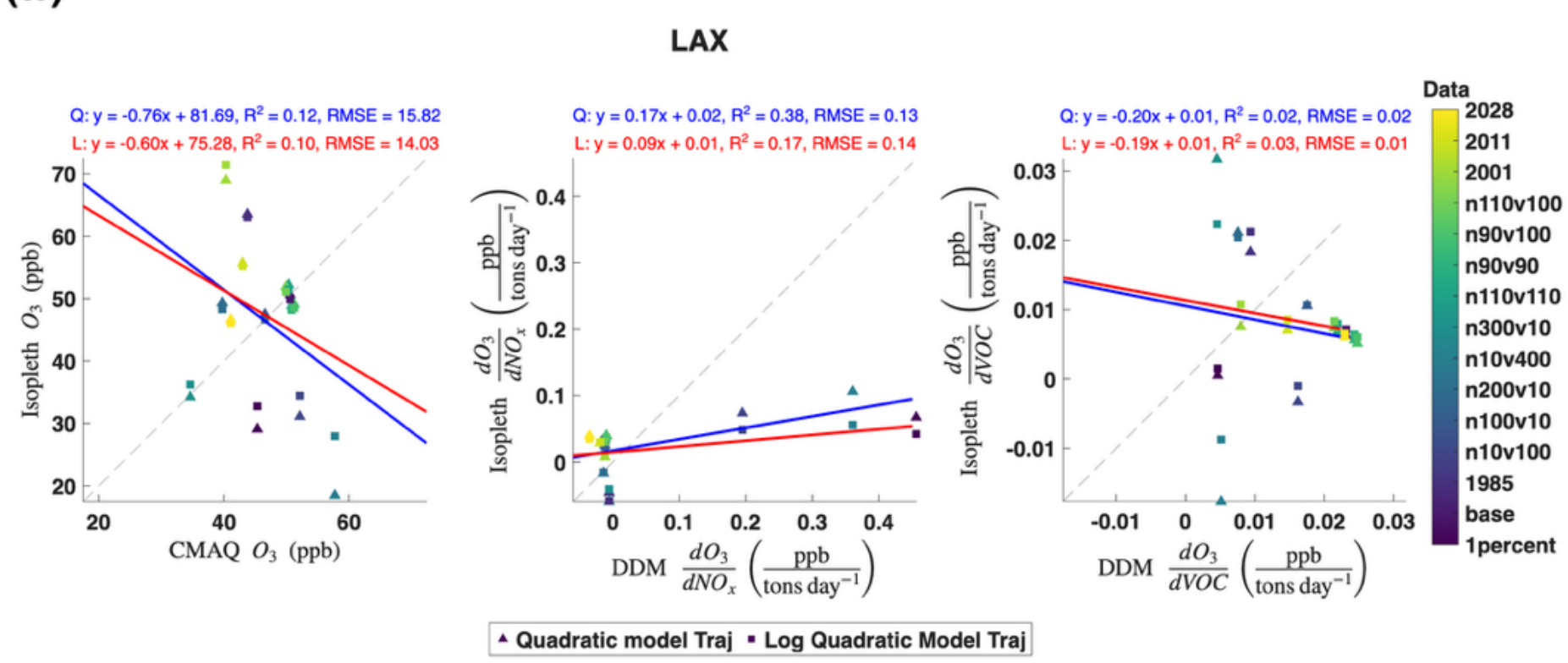
LAX
Q: y = -0.76x + 81.69, R2 = 0.12, RMSE = 15.82
L: y = -0.60x + 75.28, R2 = 0.10, RMSE = 14.03
Q: y = 0.17x + 0.02, R2 = 0.38, RMSE = 0.13
L: y = 0.09x + 0.01, R2 = 0.17, RMSE = 0.14
Q: y = -0.20x + 0.01, R2 = 0.02, RMSE = 0.02
L: y = -0.19x + 0.01, R2 = 0.03, RMSE = 0.01
Isopleth O3 (ppb)
CMAQ O3 (ppb)
DDM dO3/dNOx (ppb/tons day−1)
DDM dO3/dVOC (ppb/tons day−1)
Data
Quadratic model Traj
Log Quadratic Model Traj

(x)

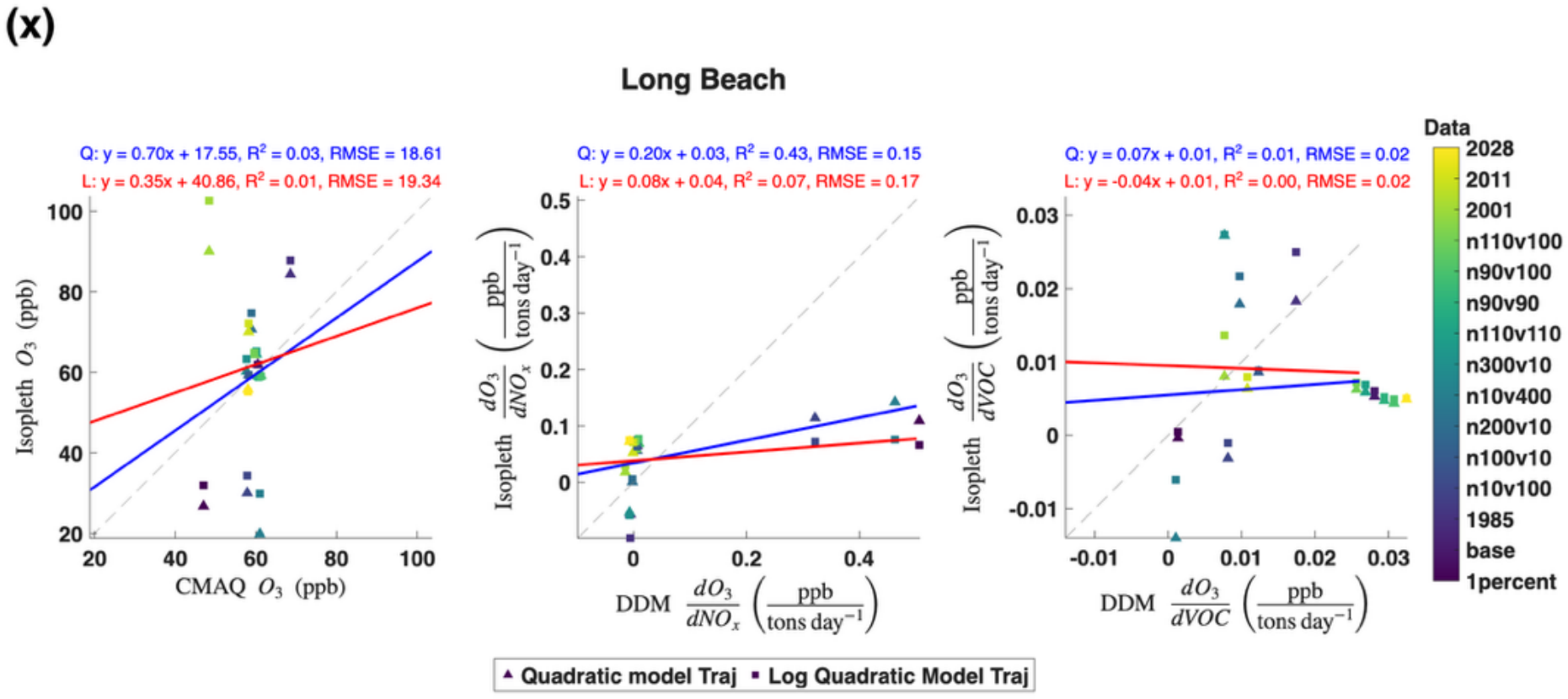
Long Beach
Q: y = 0.70x + 17.55, R2 = 0.03, RMSE = 18.61
L: y = 0.35x + 40.86, R2 = 0.01, RMSE = 19.34
Q: y = 0.20x + 0.03, R2 = 0.43, RMSE = 0.15
L: y = 0.08x + 0.04, R2 = 0.07, RMSE = 0.17
Q: y = 0.07x + 0.01, R2 = 0.01, RMSE = 0.02
L: y = -0.04x + 0.01, R2 = 0.00, RMSE = 0.02
Isopleth O3 (ppb)
CMAQ O3 (ppb)
DDM dO3/dNOx (ppb/tons day−1)
DDM dO3/dVOC (ppb/tons day−1)
Data
Quadratic model Traj
Log Quadratic Model Traj

Figure S11. Comparisons between CMAQ-HDDM estimates and values reconstructed using the quadratic and log-quadratic models, including ozone, ozone to NO_x sensitivity, and ozone to VOC sensitivity for 24 monitoring sites in SoCAB (panels a to x). Comparisons between CMAQ-HDDM and the quadratic model are shown as triangle markers, while those between CMAQ-HDDM and the log-quadratic model are shown as square markers. The red and blue lines represent the regression relationships between CMAQ-HDDM and the quadratic and log-quadratic models, respectively. Point colors indicate different emission levels used in the CMAQ-HDDM simulations.

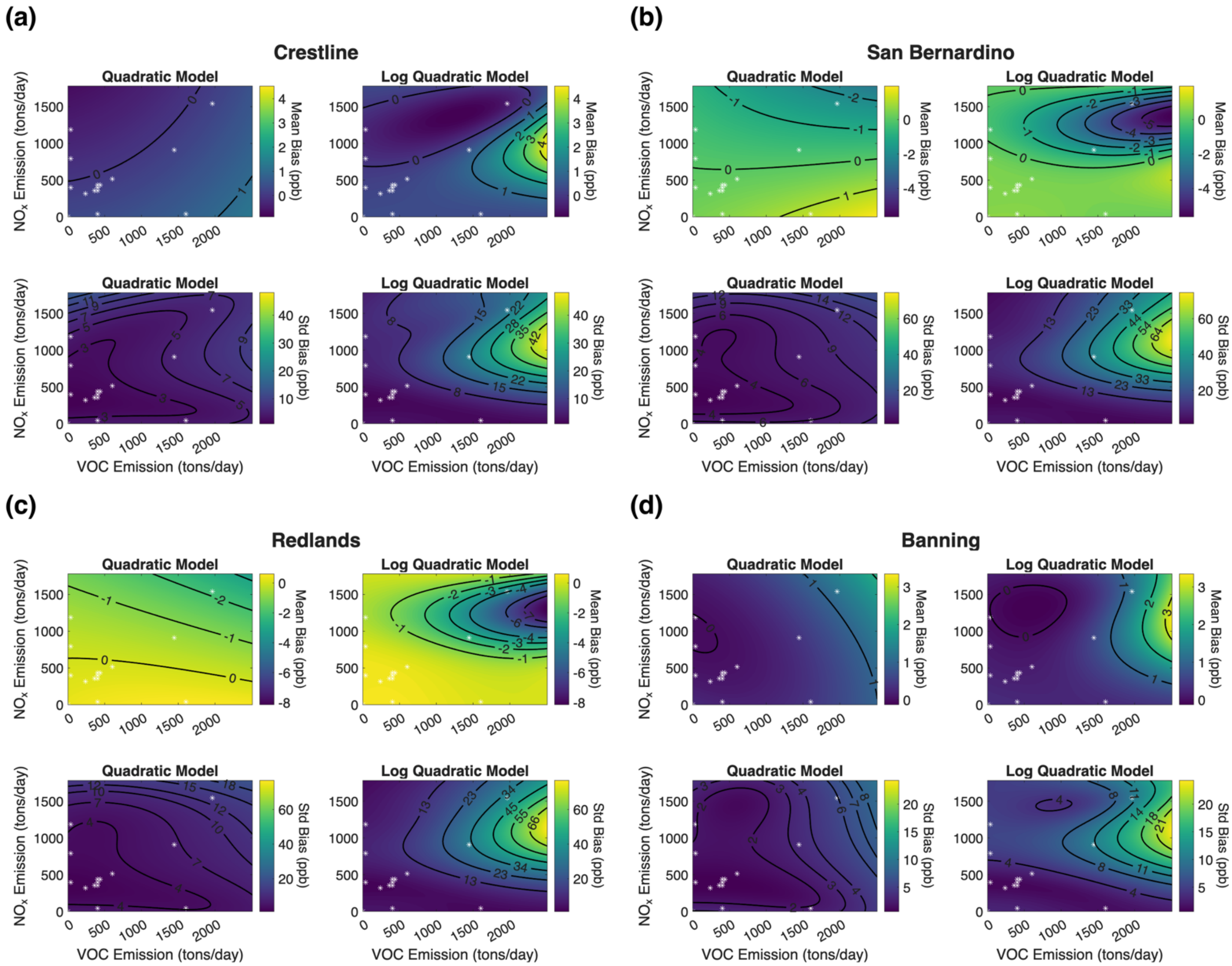
(a)
Crestline
(b)
San Bernardino
(c)
Redlands
(d)
Banning
Quadratic Model
Log Quadratic Model
NOx Emission (tons/day)
VOC Emission (tons/day)
Mean Bias (ppb)
Std Bias (ppb)

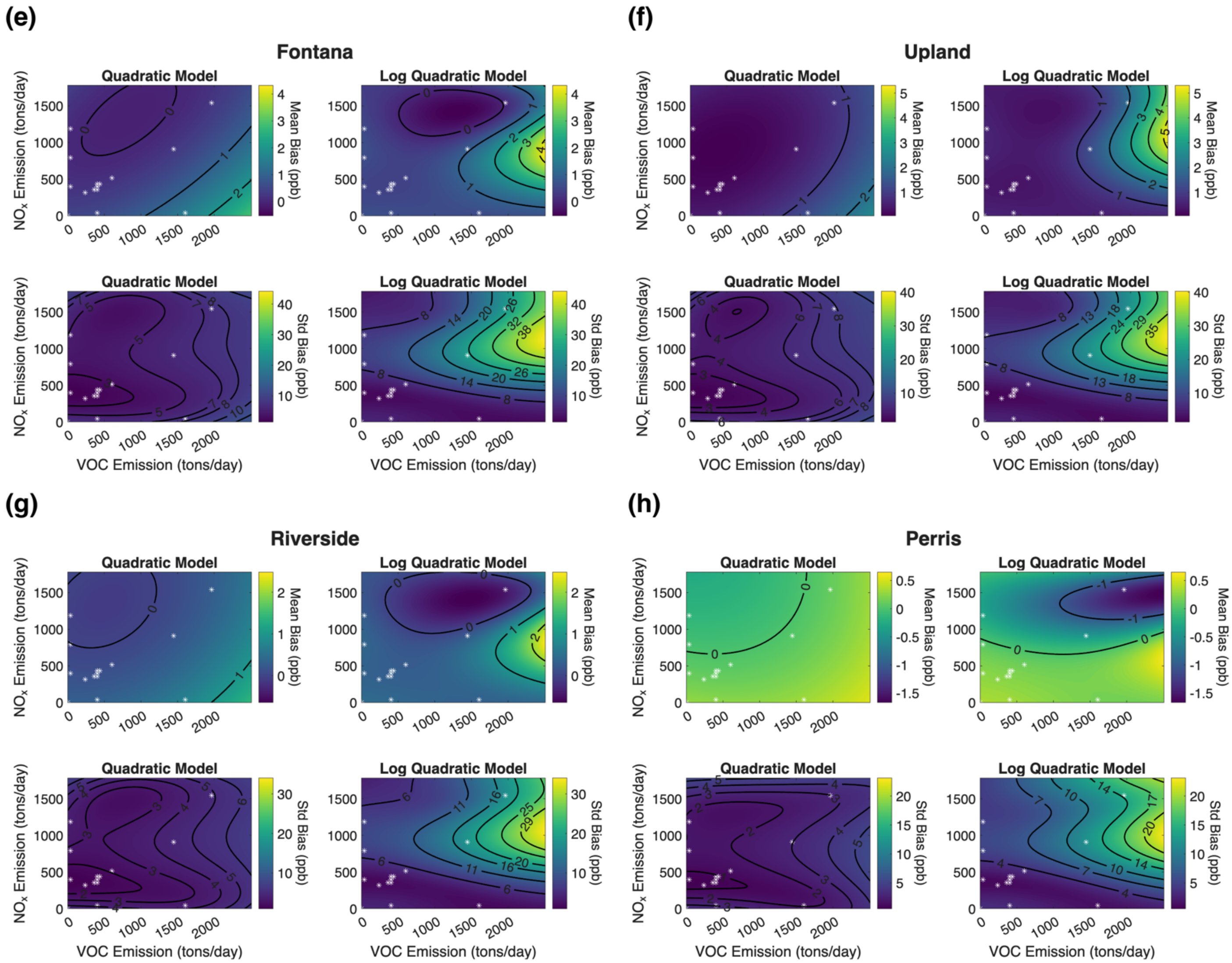
(e)
Fontana
(f)
Upland
(g)
Riverside
(h)
Perris
Quadratic Model
Log Quadratic Model
Mean Bias (ppb)
Std Bias (ppb)
NO_x Emission (tons/day)
VOC Emission (tons/day)

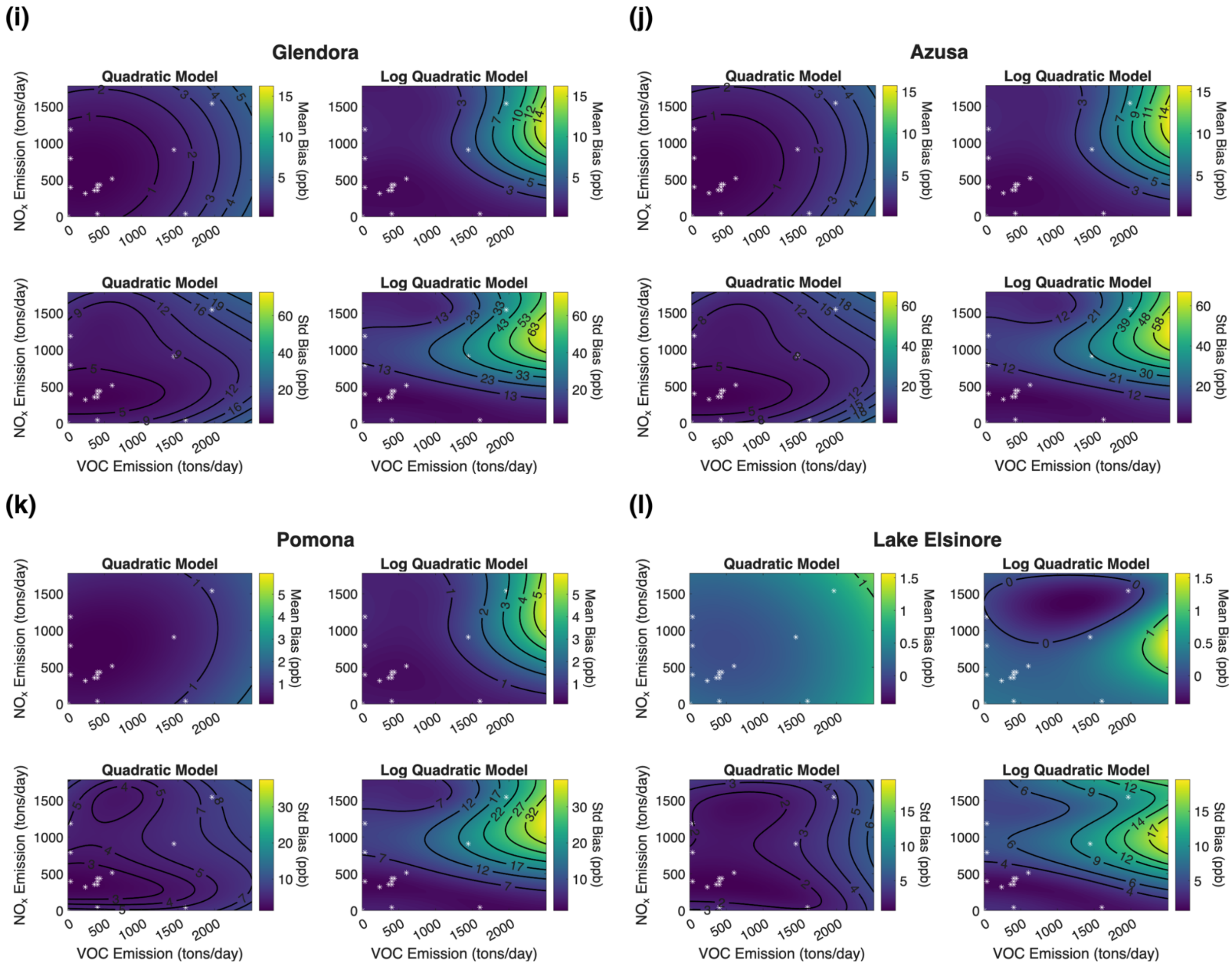
(i)
Glendora
(j)
Azusa
(k)
Pomona
(l)
Lake Elsinore
Quadratic Model
Log Quadratic Model
Mean Bias (ppb)
Std Bias (ppb)
NOx Emission (tons/day)
VOC Emission (tons/day)

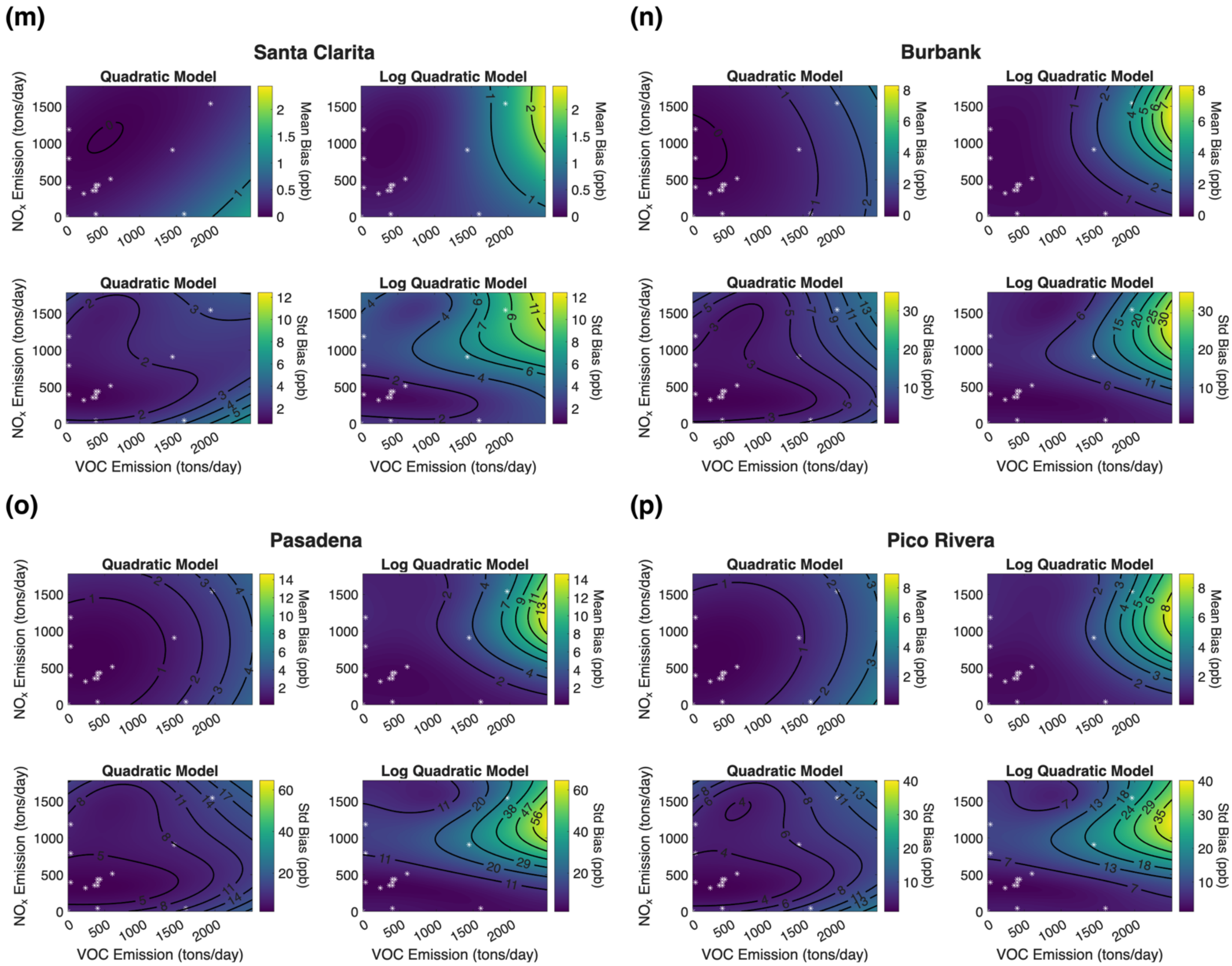
(m)
Santa Clarita
Quadratic Model
Log Quadratic Model
Mean Bias (ppb)
Std Bias (ppb)
NOx Emission (tons/day)
VOC Emission (tons/day)
(n)
Burbank
Quadratic Model
Log Quadratic Model
Mean Bias (ppb)
Std Bias (ppb)
NOx Emission (tons/day)
VOC Emission (tons/day)
(o)
Pasadena
Quadratic Model
Log Quadratic Model
Mean Bias (ppb)
Std Bias (ppb)
NOx Emission (tons/day)
VOC Emission (tons/day)
(p)
Pico Rivera
Quadratic Model
Log Quadratic Model
Mean Bias (ppb)
Std Bias (ppb)
NOx Emission (tons/day)
VOC Emission (tons/day)

(q)

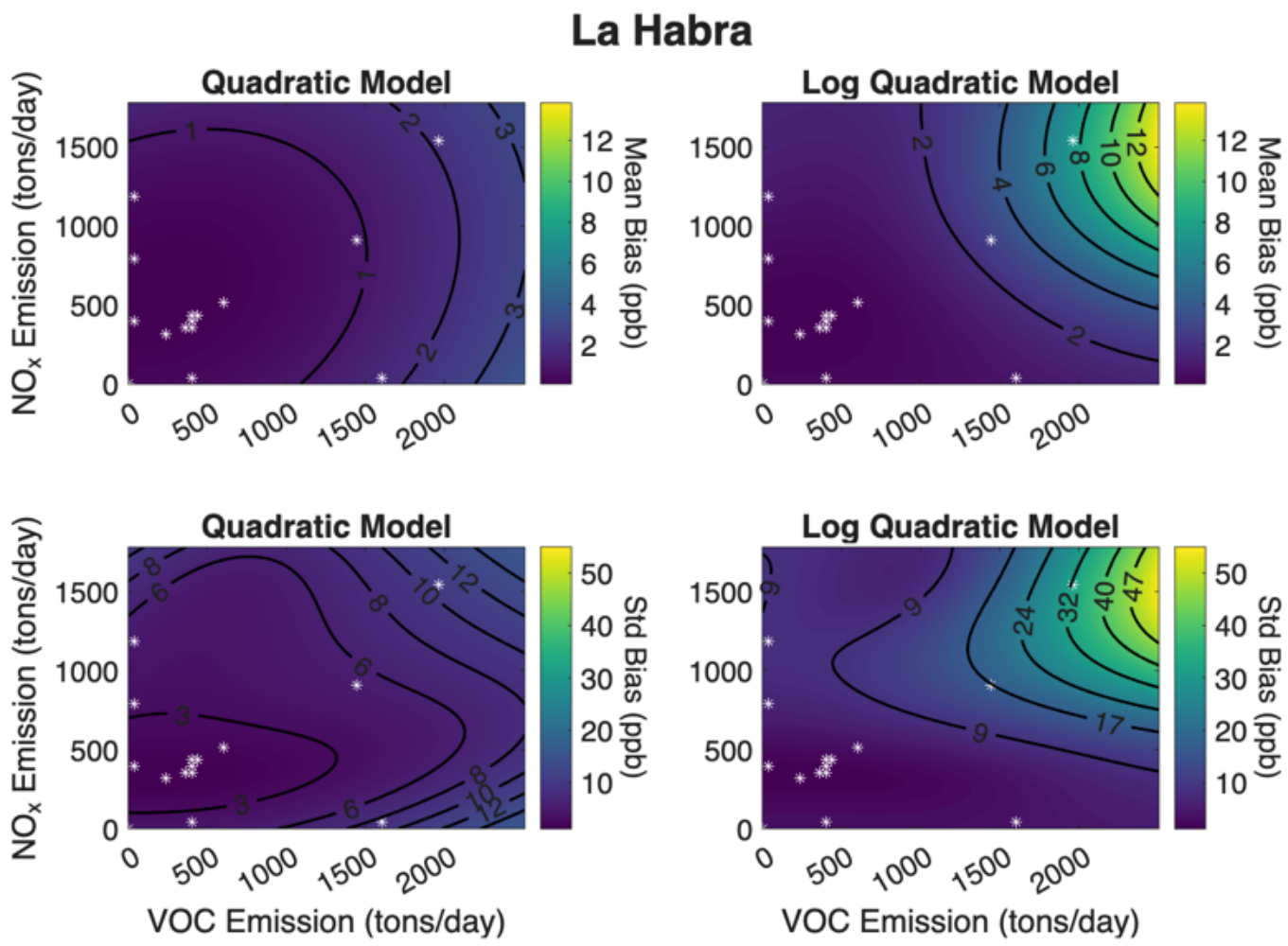
La Habra
Quadratic Model
Log Quadratic Model
Mean Bias (ppb)
Std Bias (ppb)
NO$_x$ Emission (tons/day)
VOC Emission (tons/day)

(r)

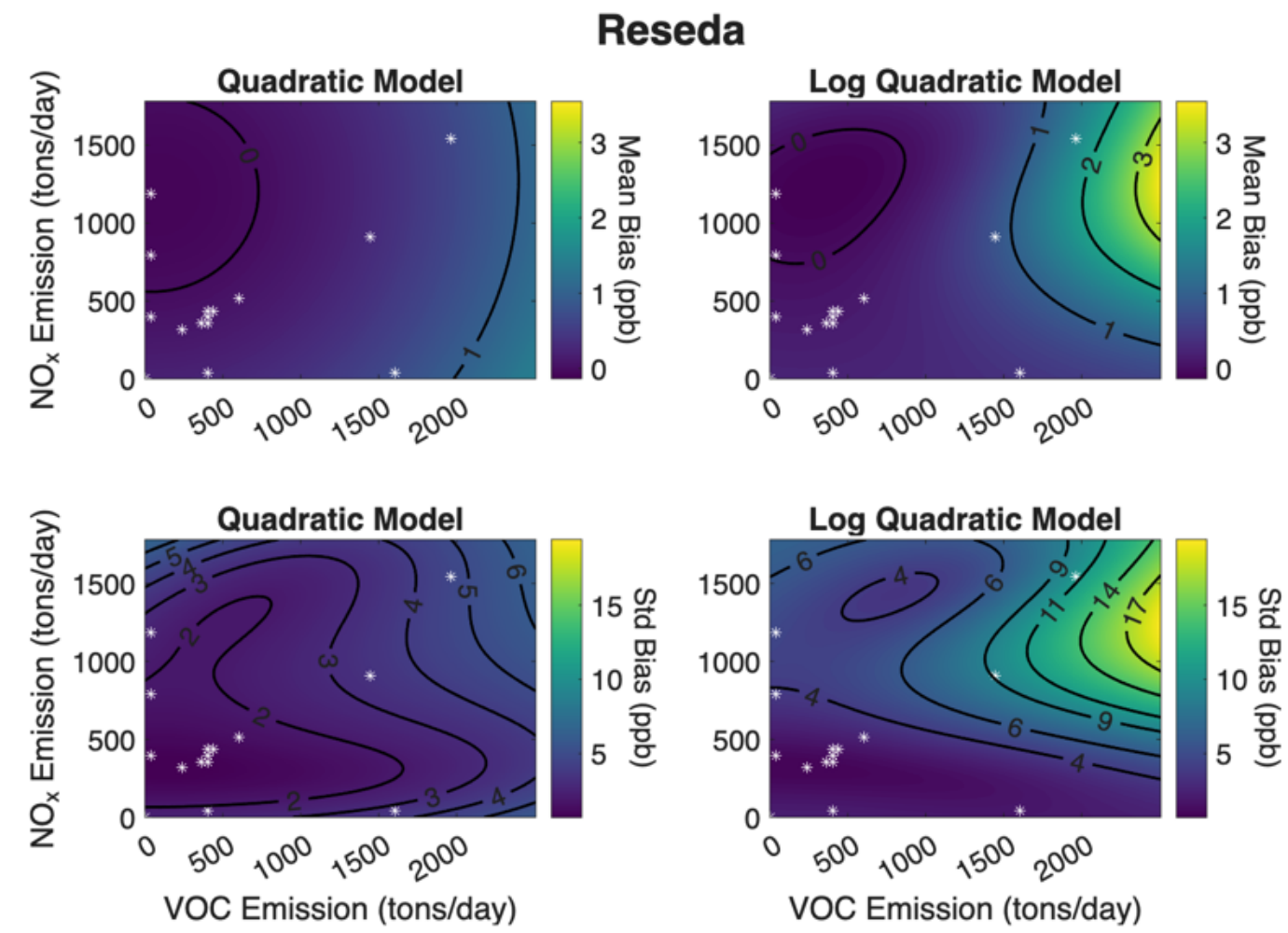
Reseda
Quadratic Model
Log Quadratic Model
Mean Bias (ppb)
Std Bias (ppb)
NO$_x$ Emission (tons/day)
VOC Emission (tons/day)

(s)

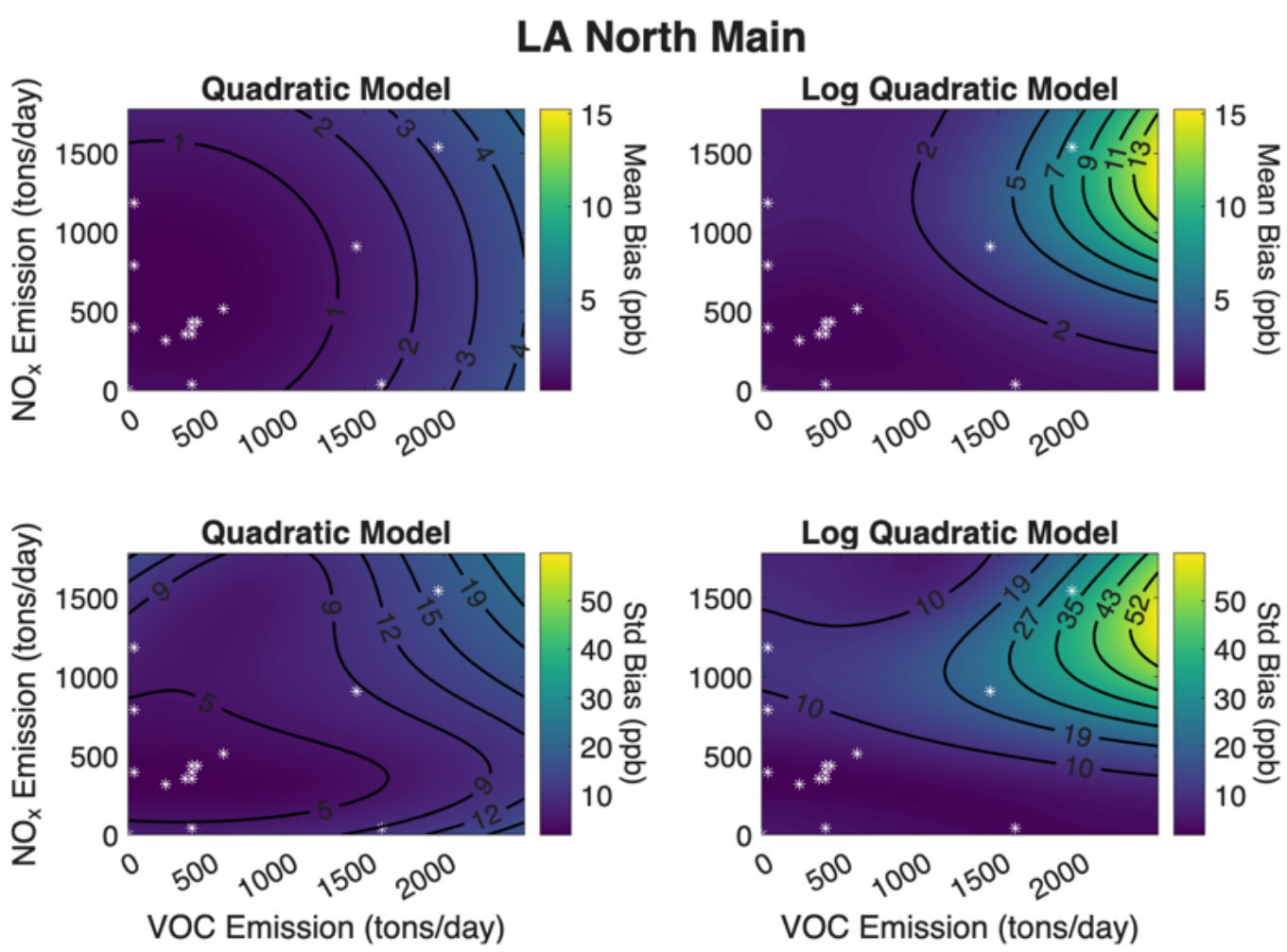
LA North Main
Quadratic Model
Log Quadratic Model
Mean Bias (ppb)
Std Bias (ppb)
NO$_x$ Emission (tons/day)
VOC Emission (tons/day)

(t)

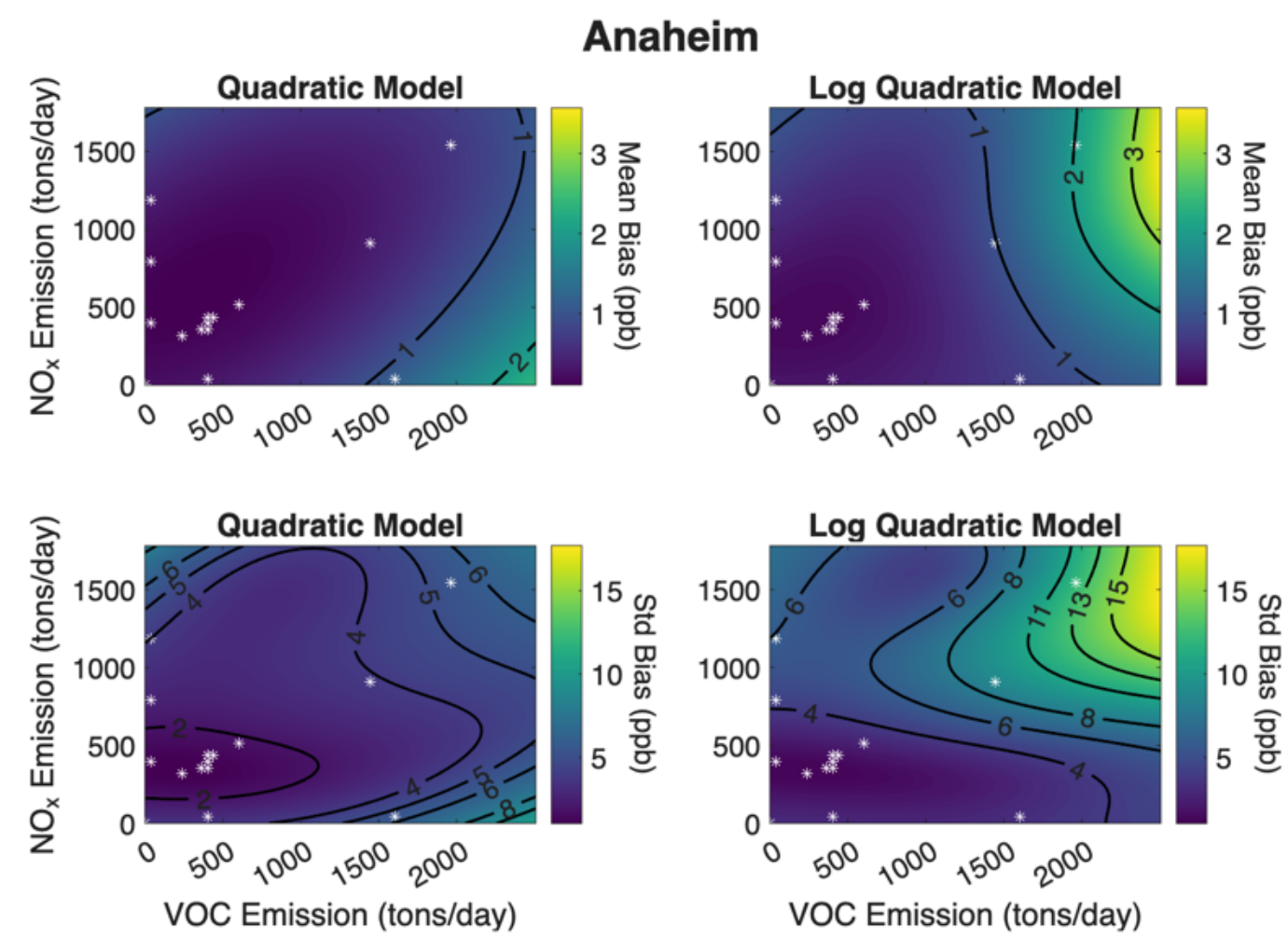
Anaheim
Quadratic Model
Log Quadratic Model
Mean Bias (ppb)
Std Bias (ppb)
NO$_x$ Emission (tons/day)
VOC Emission (tons/day)

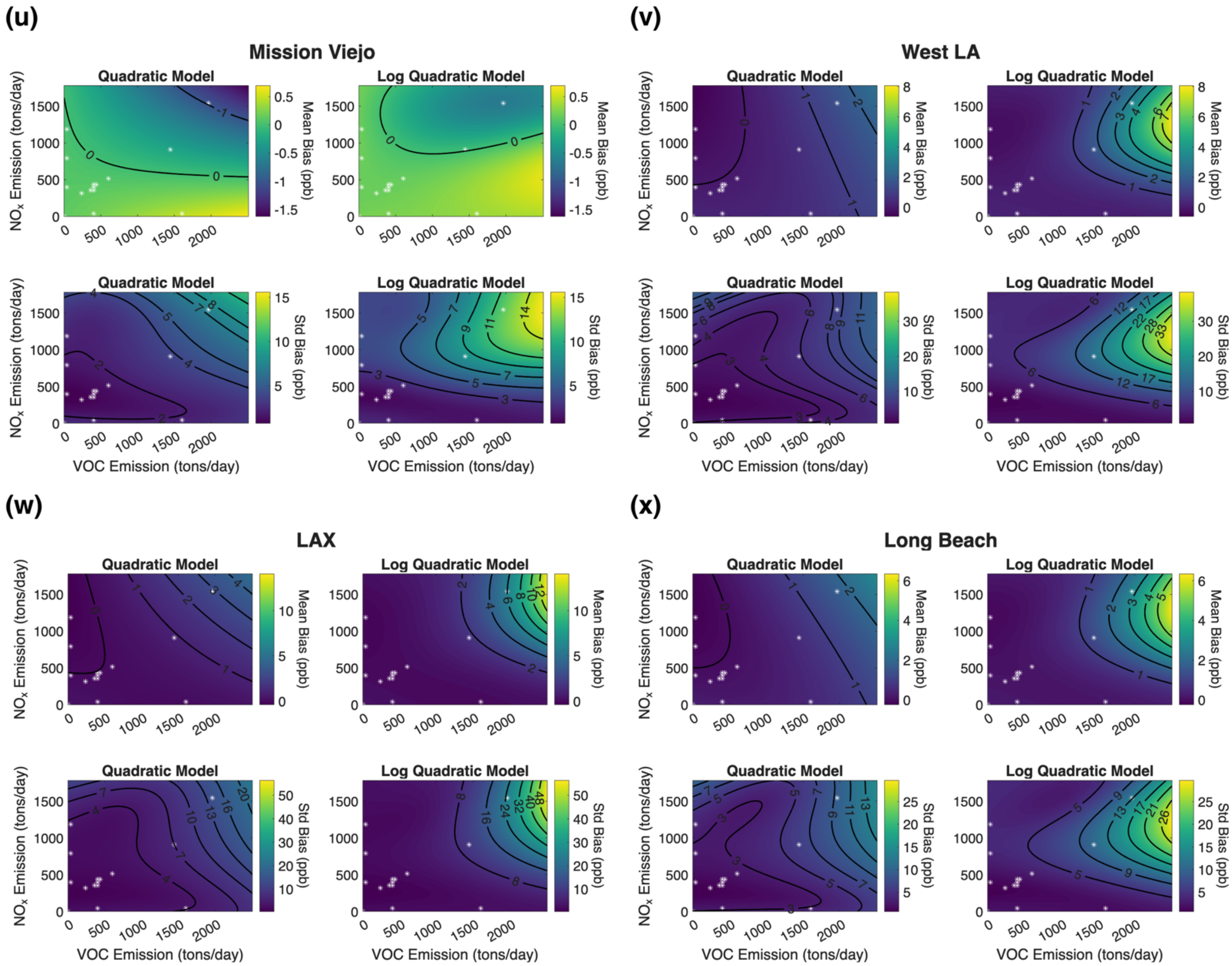

Figure S12. Mean bias (first row in each panel) and standard bias (second row in each panel) of CMAQ-HDDM-derived isopleths for 24 monitoring sites in SoCAB (panels a to x). In each panel, the first column and second column show the uncertainties of the isopleth developed by using the quadratic model and the log-quadratic model, respectively. The white crosses indicate the 15 emission levels used in the isopleth development.

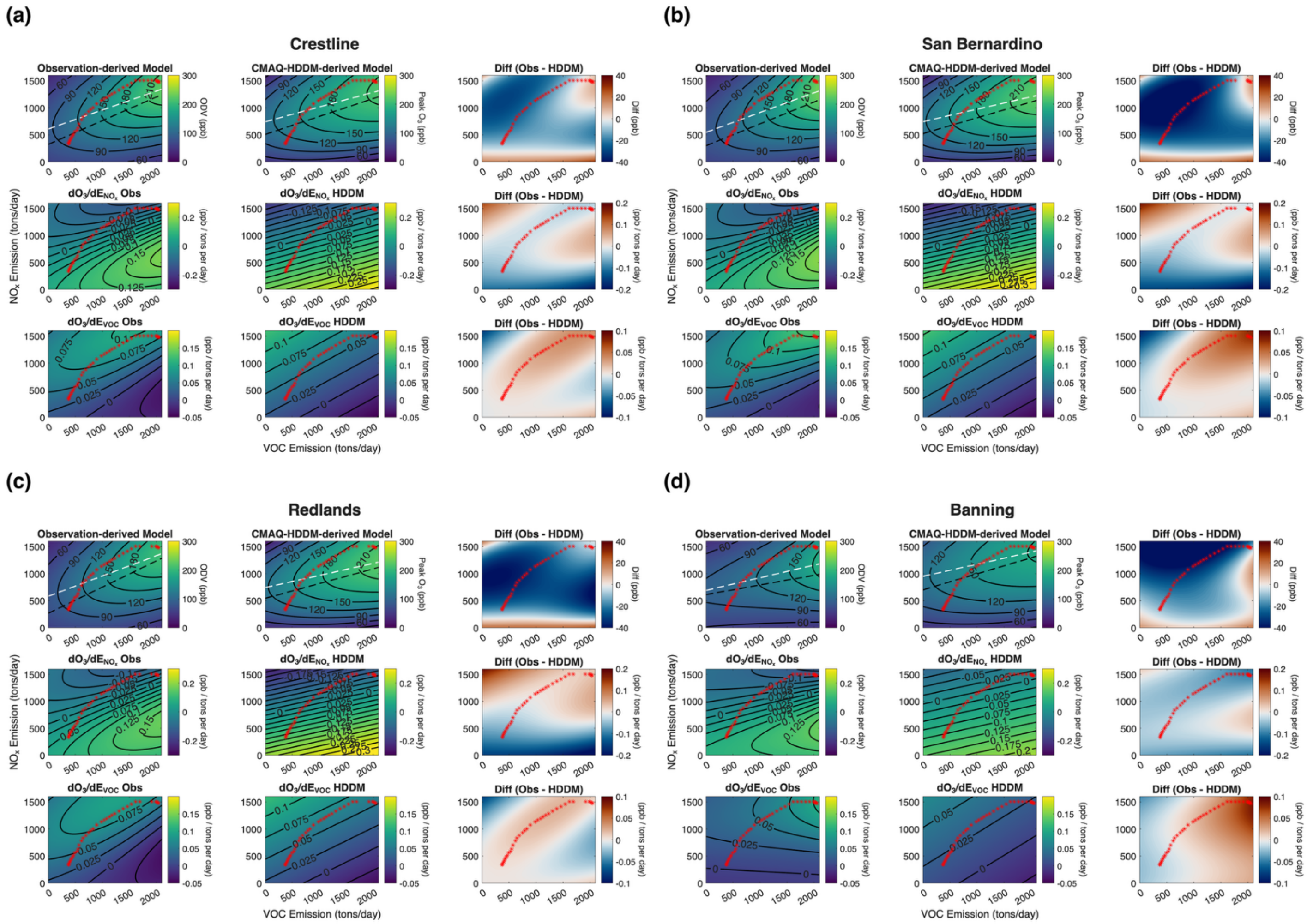

(a)
Crestline
(b)
San Bernardino
(c)
Redlands
(d)
Banning
Observation-derived Model
CMAQ-HDDM-derived Model
Diff (Obs - HDDM)
dO3/dENOx Obs
dO3/dENOx HDDM
dO3/dEVOC Obs
dO3/dEVOC HDDM
ODV (ppb)
Peak O3 (ppb)
Diff (ppb)
(ppb / tons per day)
NOx Emission (tons/day)
VOC Emission (tons/day)

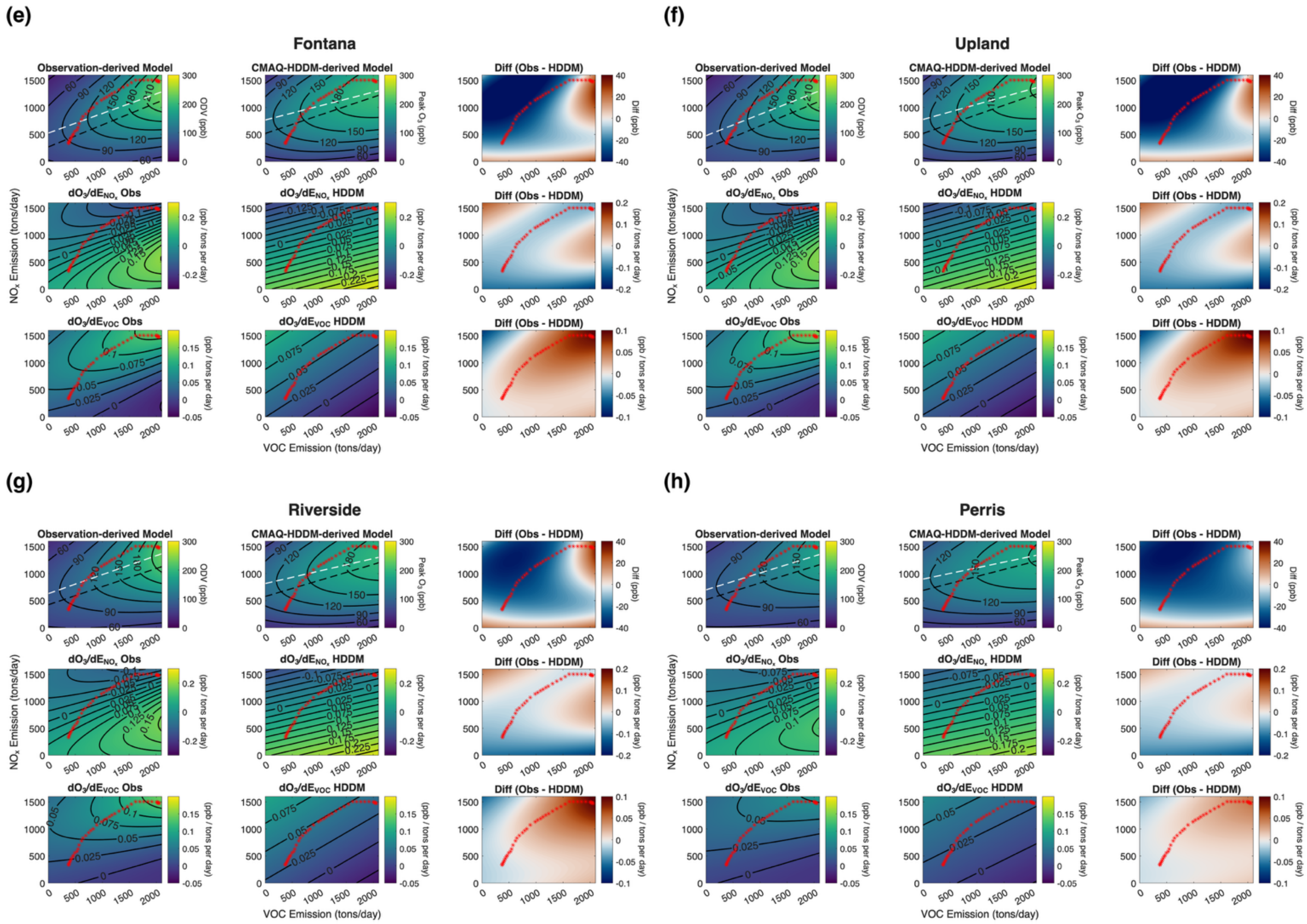
(e)
Fontana
(f)
Upland
(g)
Riverside
(h)
Perris
Observation-derived Model
CMAQ-HDDM-derived Model
Diff (Obs - HDDM)
dO3/dENOx Obs
dO3/dENOx HDDM
dO3/dEVOC Obs
dO3/dEVOC HDDM
NOx Emission (tons/day)
VOC Emission (tons/day)
ODV (ppb)
Peak O3 (ppb)
Diff (ppb)
(ppb / tons per day)

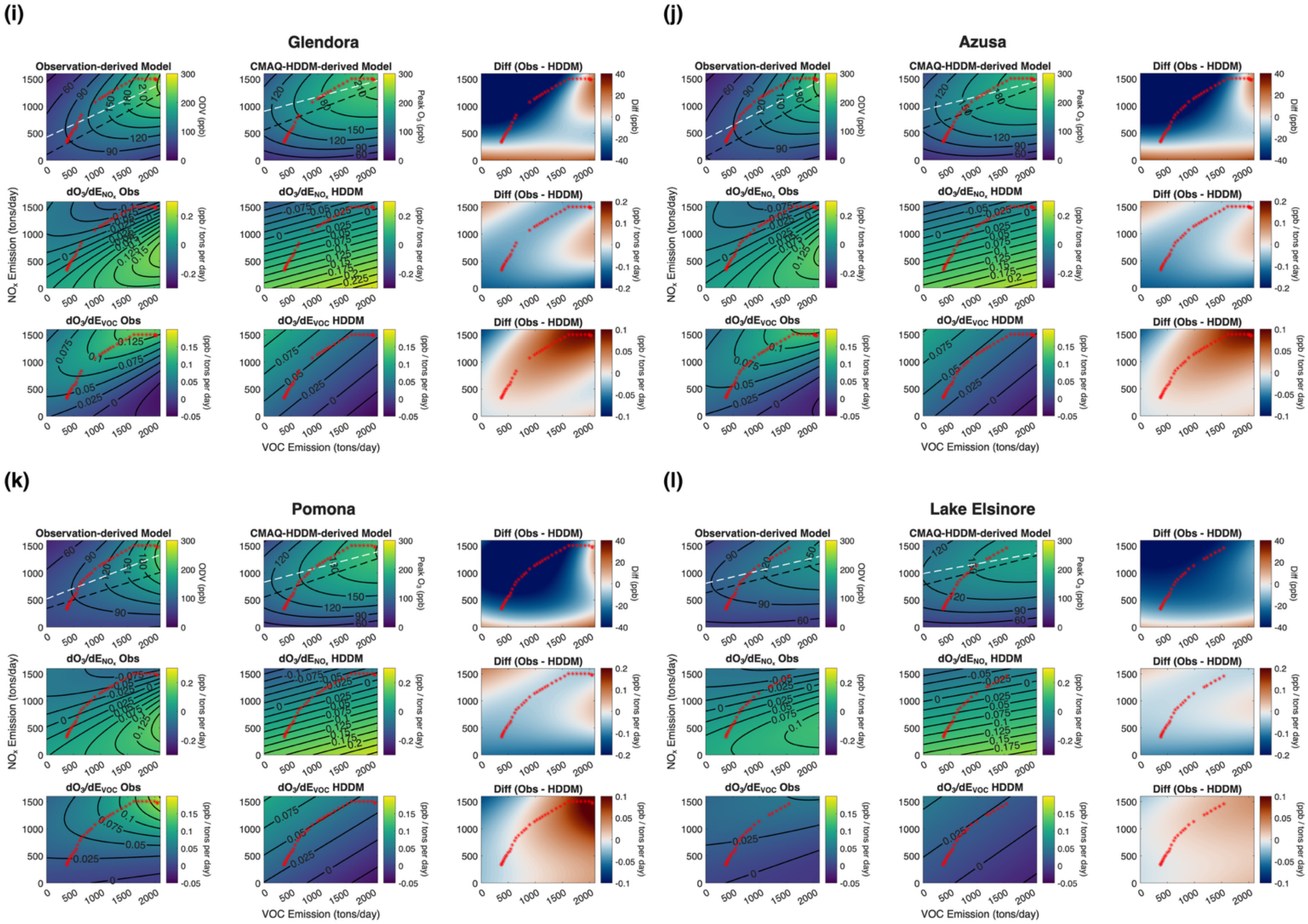
(i)
Glendora
(j)
Azusa
(k)
Pomona
(l)
Lake Elsinore
Observation-derived Model
CMAQ-HDDM-derived Model
Diff (Obs - HDDM)
dO3/dENOx Obs
dO3/dENOx HDDM
dO3/dEVOC Obs
dO3/dEVOC HDDM
ODV (ppb)
Peak O3 (ppb)
Diff (ppb)
(ppb / tons per day)
NOx Emission (tons/day)
VOC Emission (tons/day)

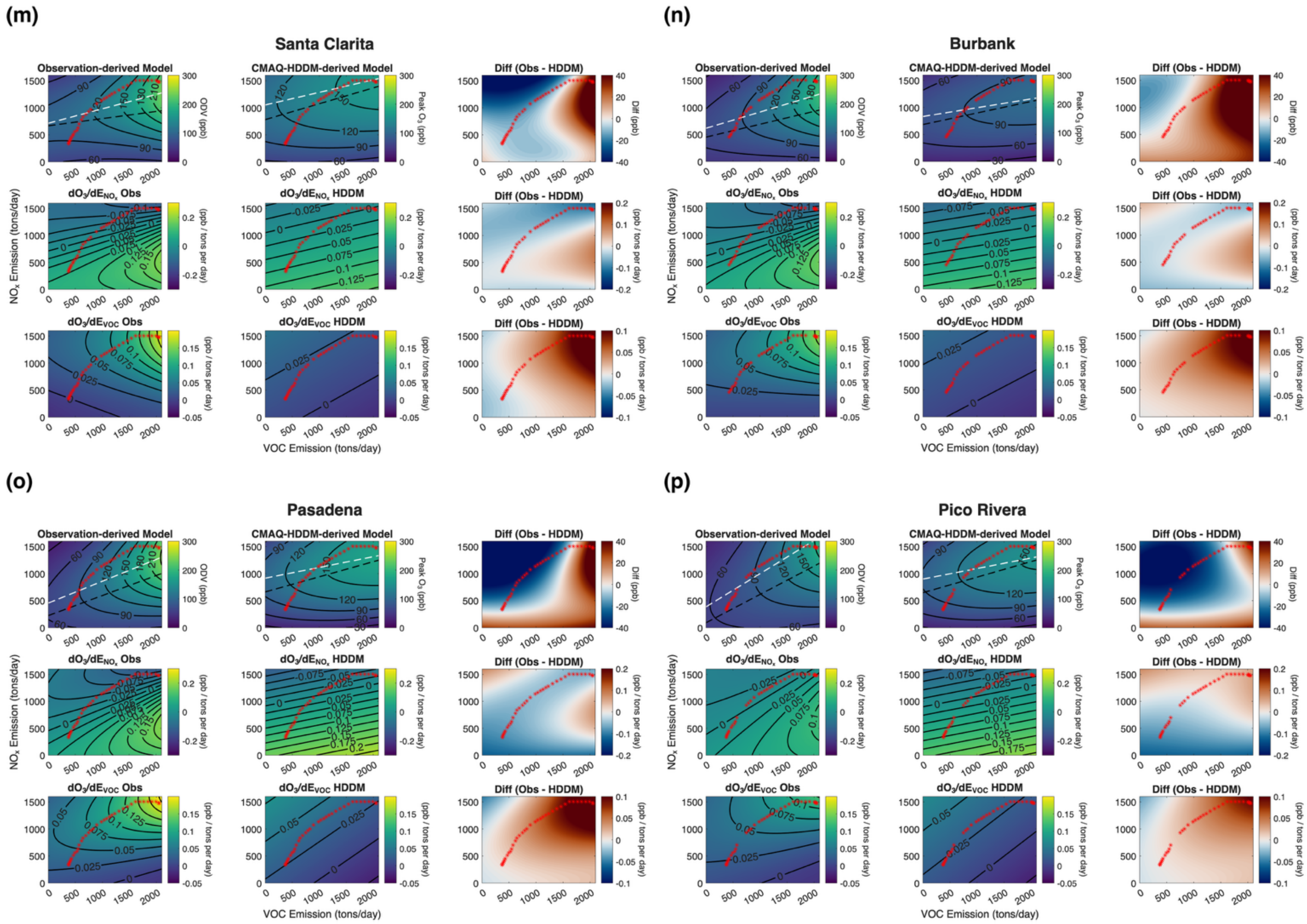
(m)
Santa Clarita
(n)
Burbank
(o)
Pasadena
(p)
Pico Rivera
Observation-derived Model
CMAQ-HDDM-derived Model
Diff (Obs - HDDM)
dO_3/dE_{NO_x} Obs
dO_3/dE_{NO_x} HDDM
dO_3/dE_{VOC} Obs
dO_3/dE_{VOC} HDDM
ODV (ppb)
Peak O_3 (ppb)
Diff (ppb)
(ppb / tons per day)
NO_x Emission (tons/day)
VOC Emission (tons/day)

(q)

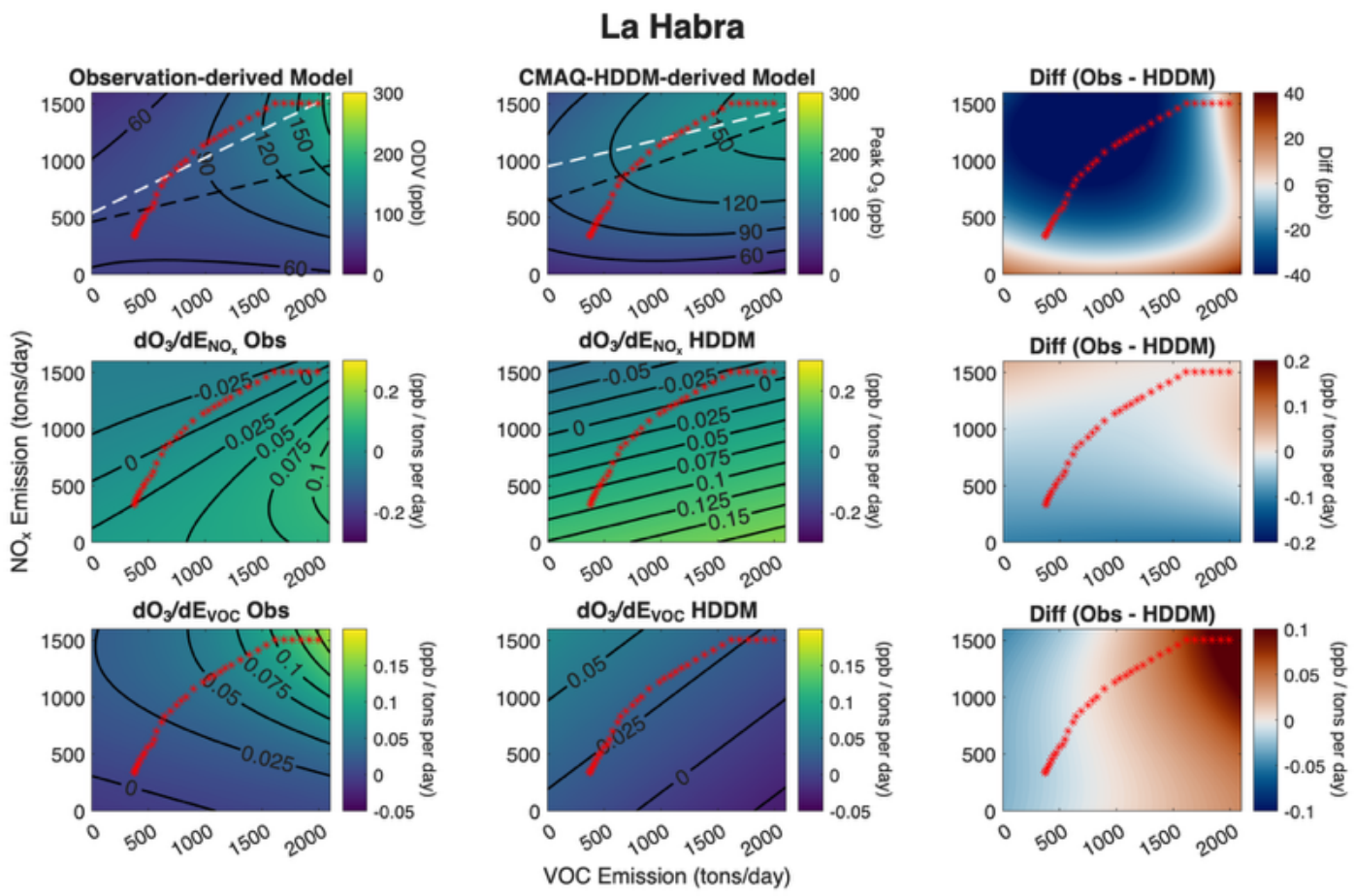
La Habra
Observation-derived Model
CMAQ-HDDM-derived Model
Diff (Obs - HDDM)
ODV (ppb)
Peak O3 (ppb)
Diff (ppb)
dO3/dENOx Obs
dO3/dENOx HDDM
dO3/dEVOC Obs
dO3/dEVOC HDDM
(ppb / tons per day)
NOx Emission (tons/day)
VOC Emission (tons/day)

(r)

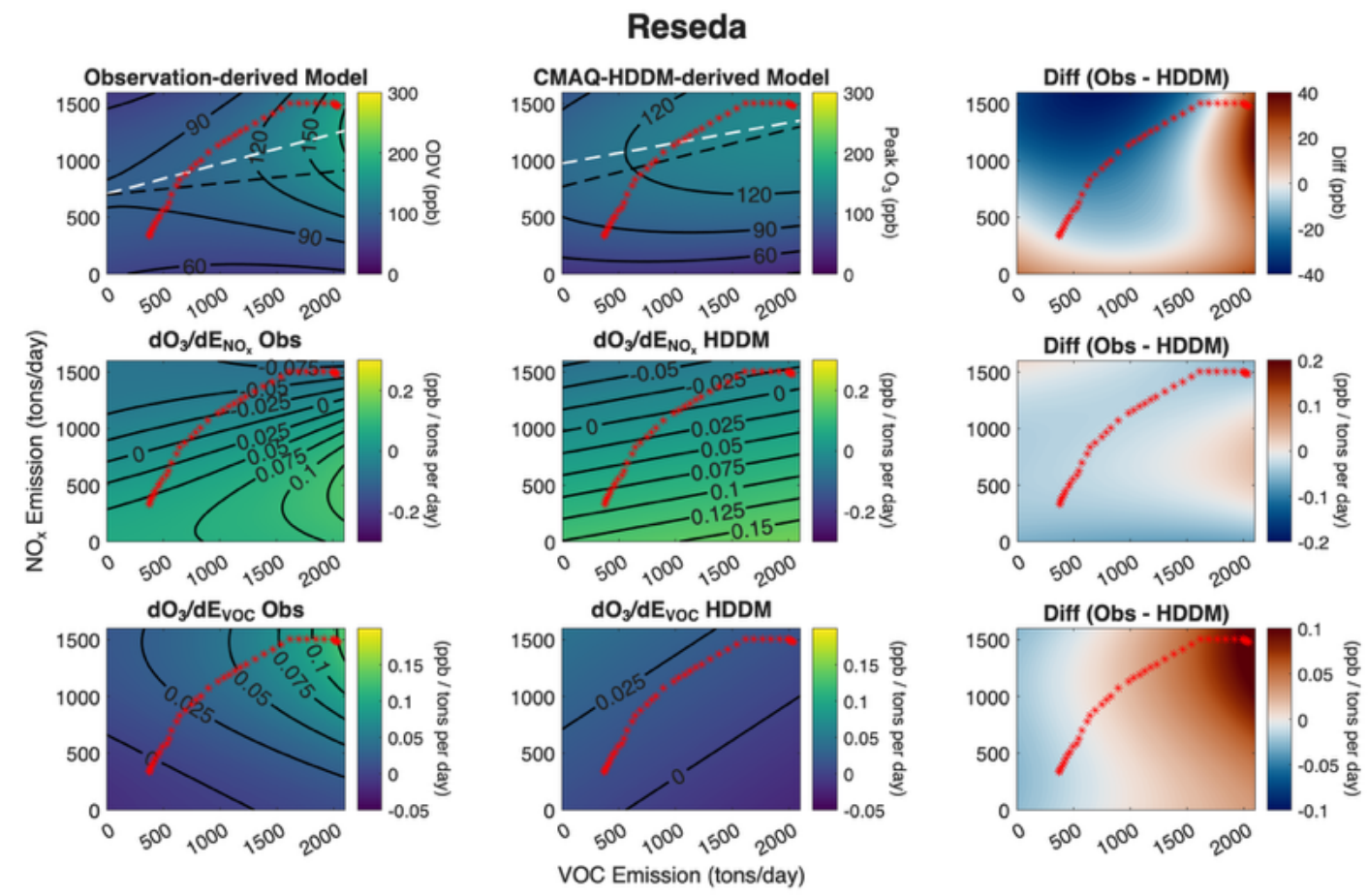
Reseda
Observation-derived Model
CMAQ-HDDM-derived Model
Diff (Obs - HDDM)
ODV (ppb)
Peak O3 (ppb)
Diff (ppb)
dO3/dENOx Obs
dO3/dENOx HDDM
dO3/dEVOC Obs
dO3/dEVOC HDDM
(ppb / tons per day)
NOx Emission (tons/day)
VOC Emission (tons/day)

(s)

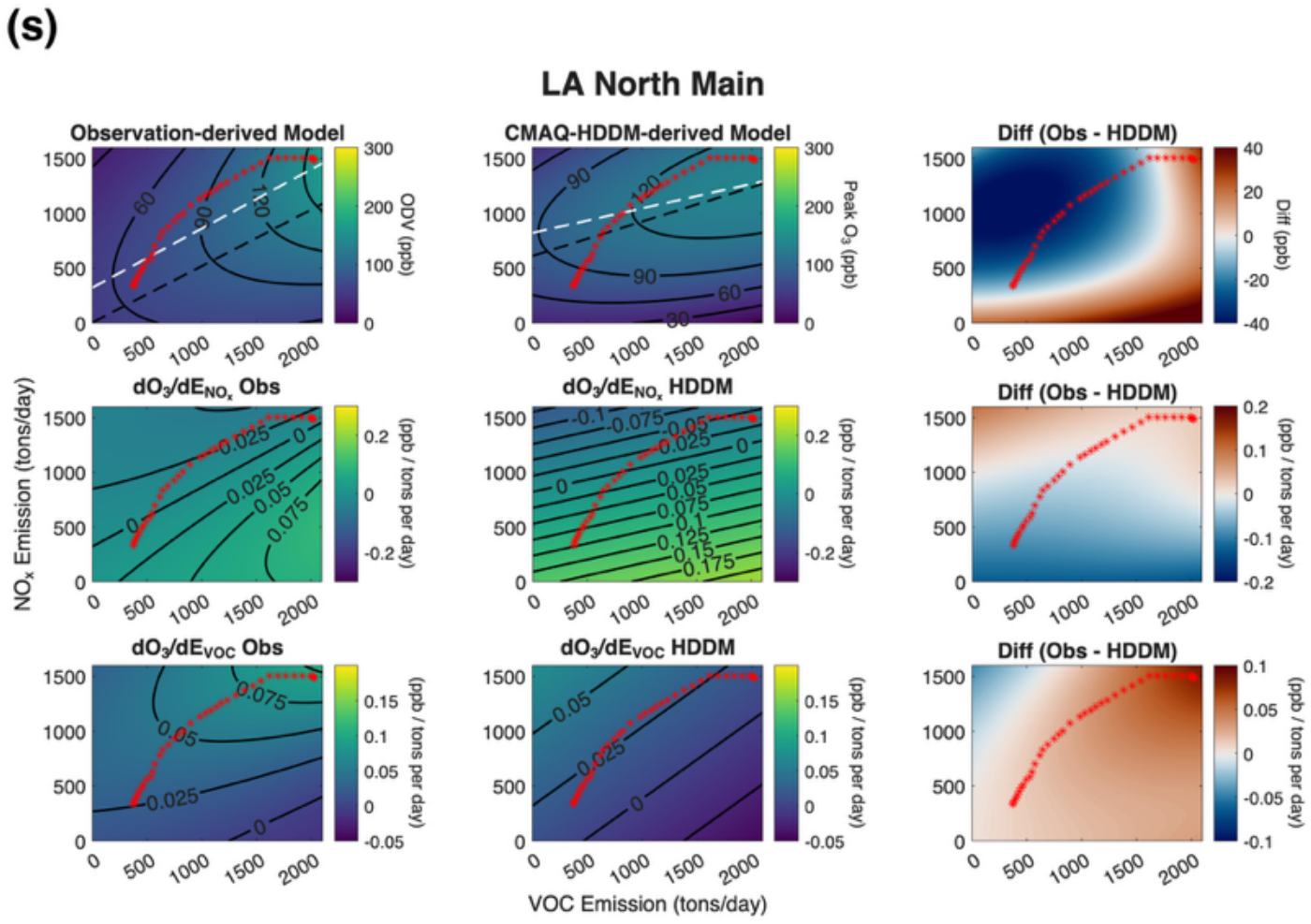
LA North Main
Observation-derived Model
CMAQ-HDDM-derived Model
Diff (Obs - HDDM)
ODV (ppb)
Peak O3 (ppb)
Diff (ppb)
dO3/dENOx Obs
dO3/dENOx HDDM
dO3/dEVOC Obs
dO3/dEVOC HDDM
(ppb / tons per day)
NOx Emission (tons/day)
VOC Emission (tons/day)

(t)

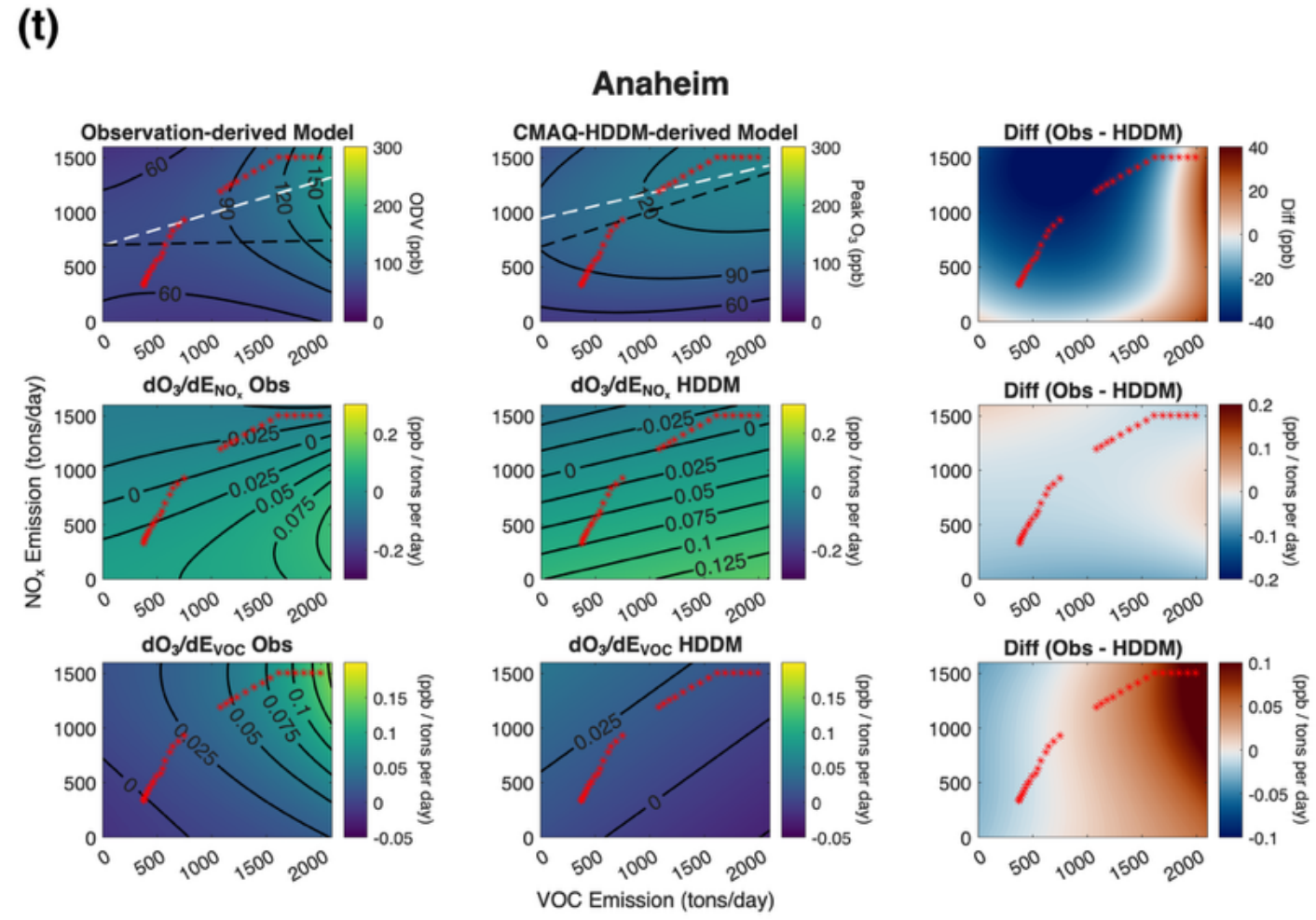
Anaheim
Observation-derived Model
CMAQ-HDDM-derived Model
Diff (Obs - HDDM)
ODV (ppb)
Peak O3 (ppb)
Diff (ppb)
dO3/dENOx Obs
dO3/dENOx HDDM
dO3/dEVOC Obs
dO3/dEVOC HDDM
(ppb / tons per day)
NOx Emission (tons/day)
VOC Emission (tons/day)

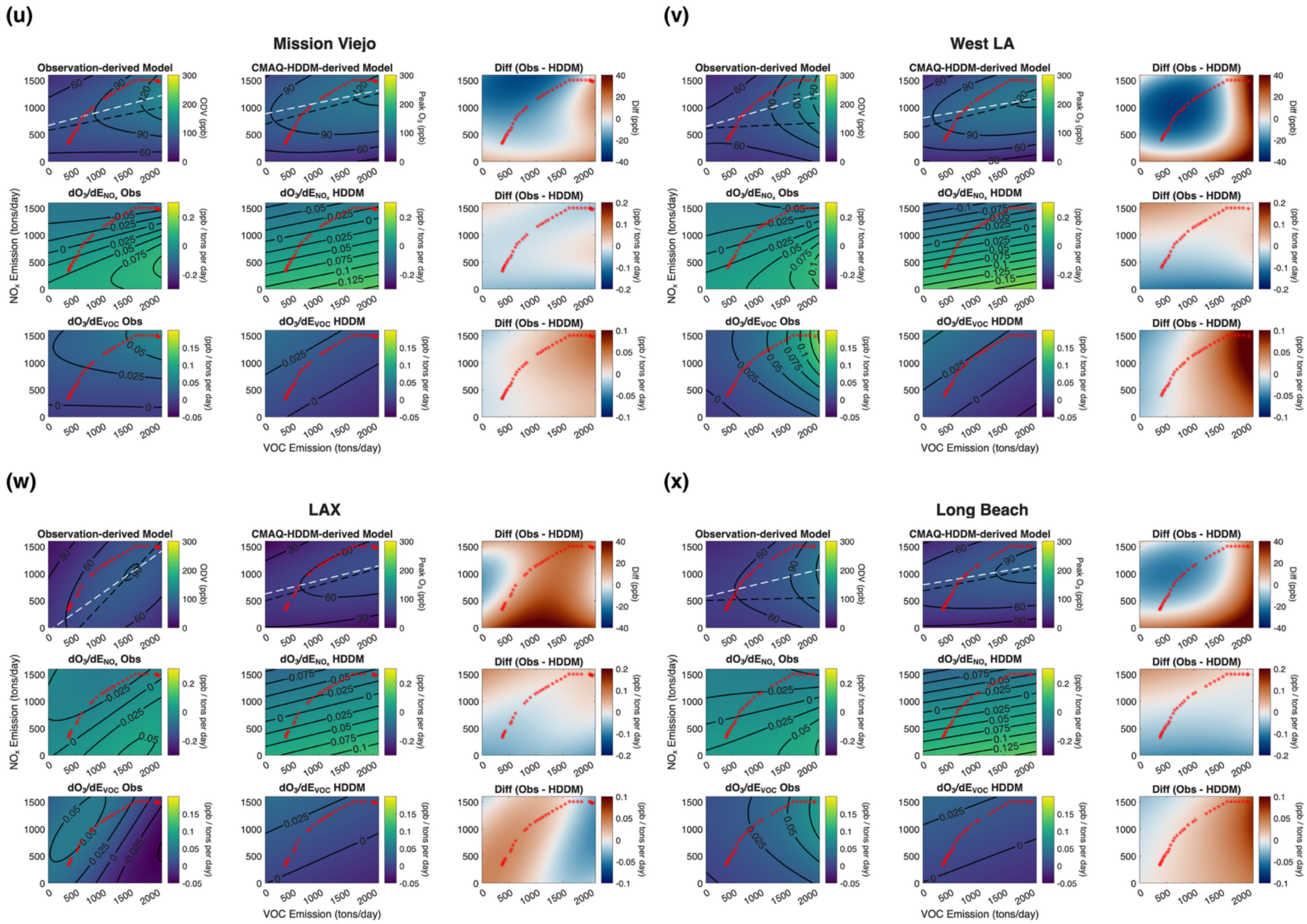

Figure S13. Comparisons between the CMAQ-HDDM-derived (quadratic model) and the observation-derived (log-quadratic model) ozone-emission concentrations and sensitivity isopleth for 24 monitoring sites at SoCAB (panels a to x). For each panel, the first column shows the observation-derived isopleths; the second column shows the CMAQ-HDDM-derived isopleths; the third column shows the difference between those two. For each panel, the first row is the ozone concentration isopleths and their differences; the second row is the ozone-to-NO_x emissions sensitivity isopleths and their differences; and the third row is the ozone-to-VOC emissions sensitivity isopleths and their differences. The black dashed line indicates the zero-NO_x-sensitivity line, and the white dashed line indicates the equal-NO_x-VOC sensitivity line. The red points show the historical emission trajectory from 1980 to 2019.

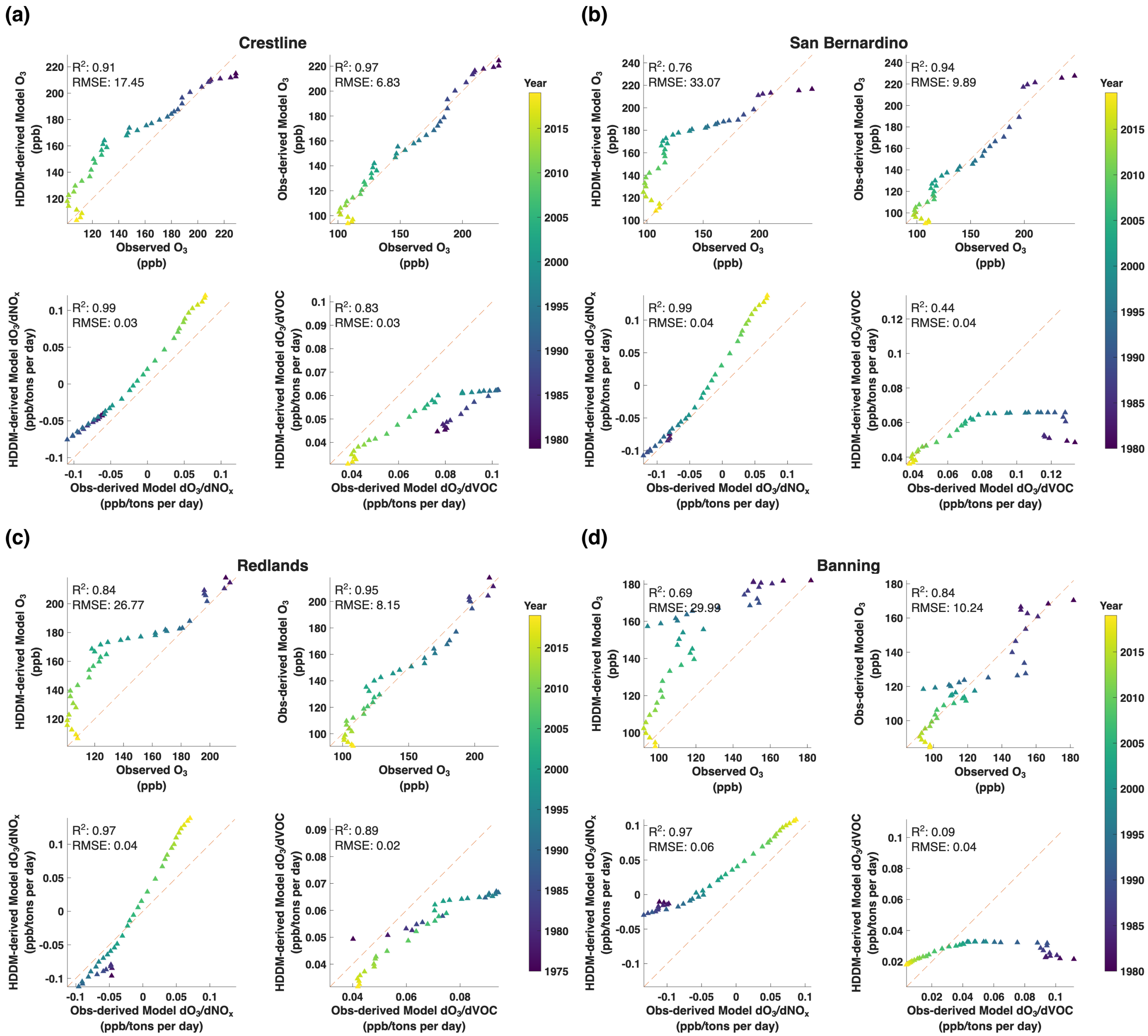

(a)
Crestline
R^2: 0.91
RMSE: 17.45
HDDM-derived Model O$_3$ (ppb)
Observed O$_3$ (ppb)
R^2: 0.97
RMSE: 6.83
Obs-derived Model O$_3$ (ppb)
R^2: 0.99
RMSE: 0.03
HDDM-derived Model dO$_3$/dNO$_x$ (ppb/tons per day)
Obs-derived Model dO$_3$/dNO$_x$ (ppb/tons per day)
R^2: 0.83
RMSE: 0.03
HDDM-derived Model dO$_3$/dVOC (ppb/tons per day)
Obs-derived Model dO$_3$/dVOC (ppb/tons per day)
Year
(b)
San Bernardino
R^2: 0.76
RMSE: 33.07
R^2: 0.94
RMSE: 9.89
R^2: 0.99
RMSE: 0.04
R^2: 0.44
RMSE: 0.04
(c)
Redlands
R^2: 0.84
RMSE: 26.77
R^2: 0.95
RMSE: 8.15
R^2: 0.97
RMSE: 0.04
R^2: 0.89
RMSE: 0.02
(d)
Banning
R^2: 0.69
RMSE: 29.99
R^2: 0.84
RMSE: 10.24
R^2: 0.97
RMSE: 0.06
R^2: 0.09
RMSE: 0.04

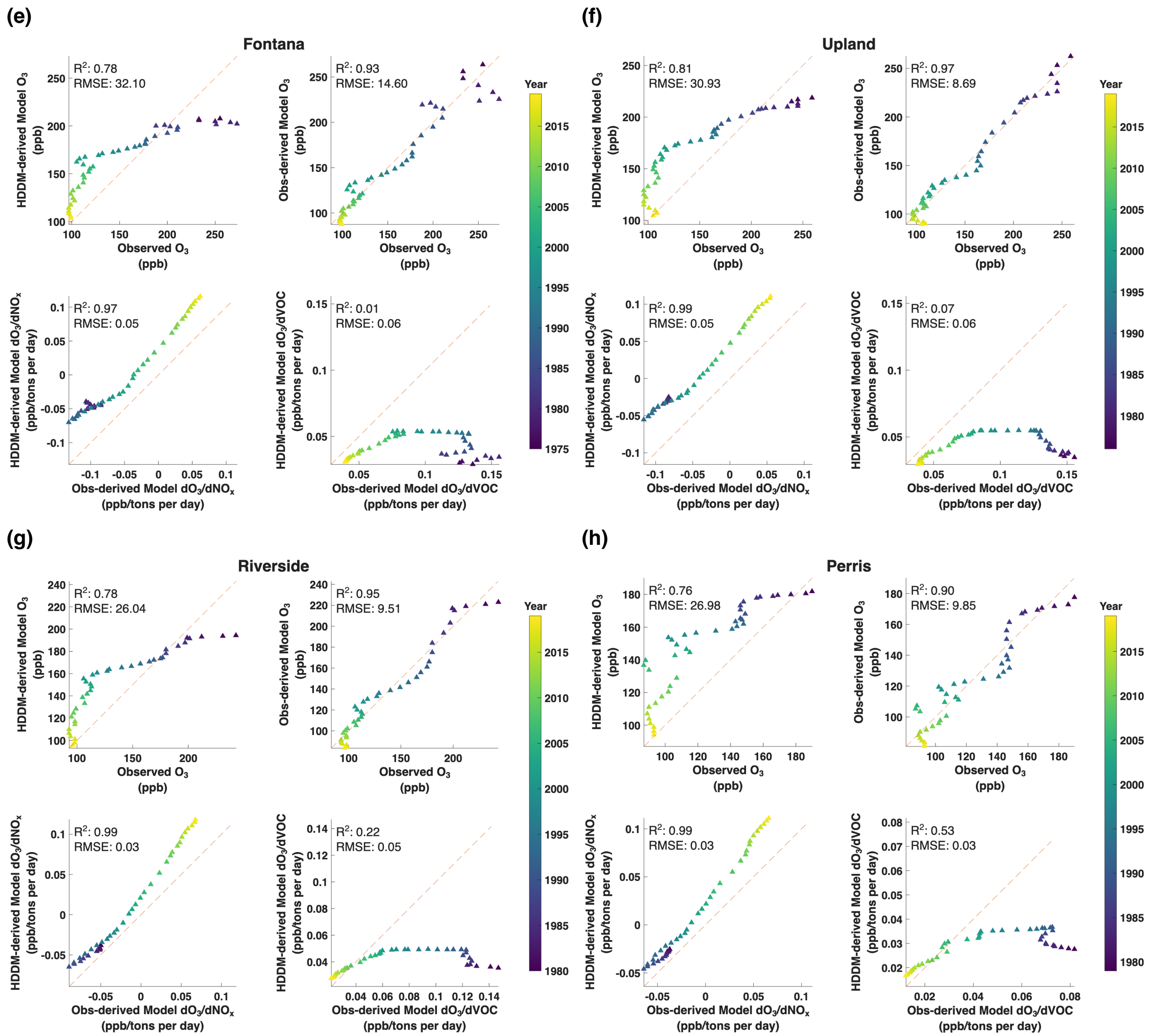
(e)
Fontana
R²: 0.78
RMSE: 32.10
HDDM-derived Model O3 (ppb)
Observed O3 (ppb)
R²: 0.93
RMSE: 14.60
Obs-derived Model O3 (ppb)
Observed O3 (ppb)
R²: 0.97
RMSE: 0.05
HDDM-derived Model dO3/dNOx (ppb/tons per day)
Obs-derived Model dO3/dNOx (ppb/tons per day)
R²: 0.01
RMSE: 0.06
HDDM-derived Model dO3/dVOC (ppb/tons per day)
Obs-derived Model dO3/dVOC (ppb/tons per day)
Year
(f)
Upland
R²: 0.81
RMSE: 30.93
R²: 0.97
RMSE: 8.69
R²: 0.99
RMSE: 0.05
R²: 0.07
RMSE: 0.06
(g)
Riverside
R²: 0.78
RMSE: 26.04
R²: 0.95
RMSE: 9.51
R²: 0.99
RMSE: 0.03
R²: 0.22
RMSE: 0.05
(h)
Perris
R²: 0.76
RMSE: 26.98
R²: 0.90
RMSE: 9.85
R²: 0.99
RMSE: 0.03
R²: 0.53
RMSE: 0.03

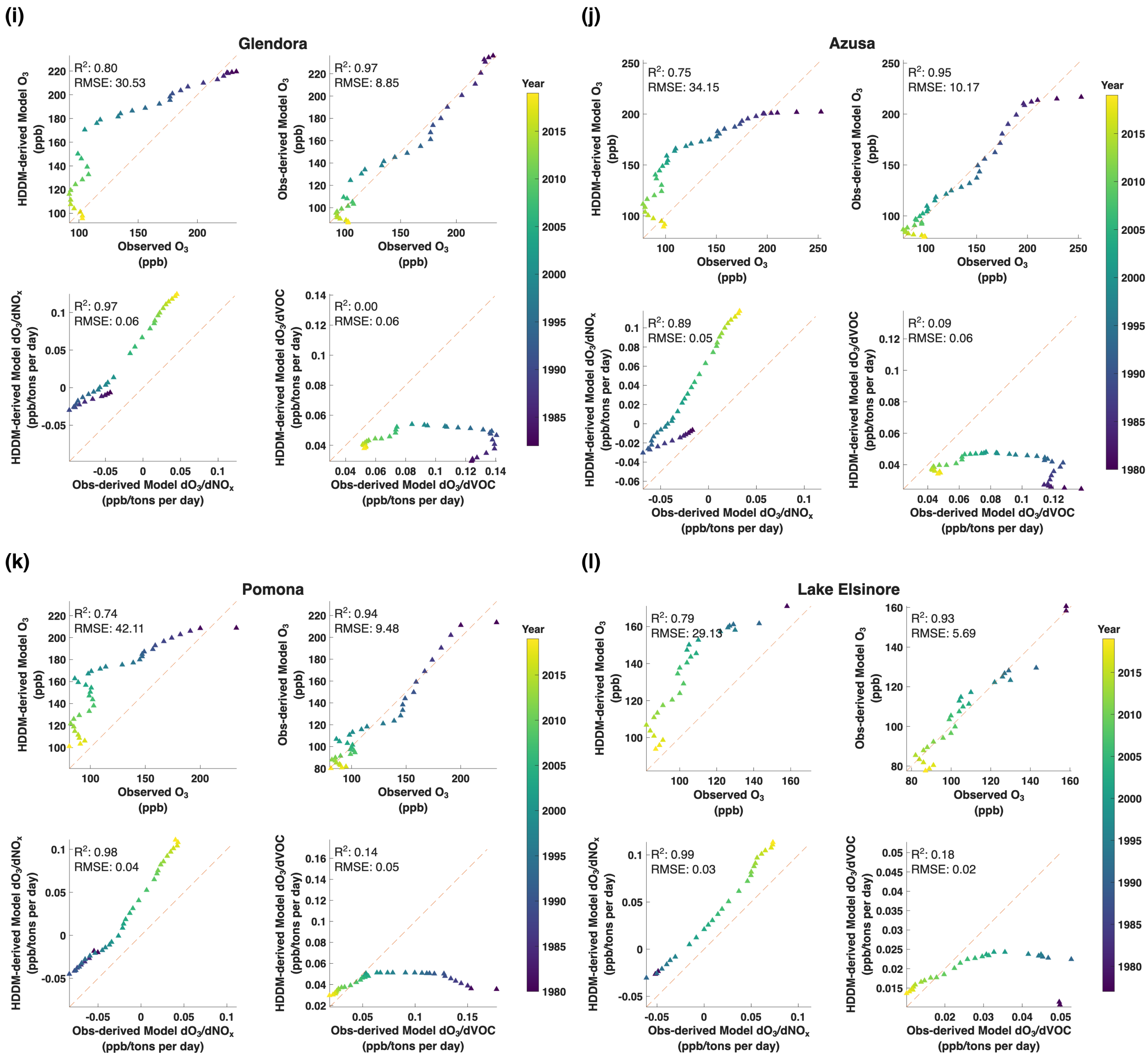

(i)
Glendora
R²: 0.80
RMSE: 30.53
HDDM-derived Model O3 (ppb)
Observed O3 (ppb)
R²: 0.97
RMSE: 8.85
Obs-derived Model O3 (ppb)
Observed O3 (ppb)
R²: 0.97
RMSE: 0.06
HDDM-derived Model dO3/dNOx (ppb/tons per day)
Obs-derived Model dO3/dNOx (ppb/tons per day)
R²: 0.00
RMSE: 0.06
HDDM-derived Model dO3/dVOC (ppb/tons per day)
Obs-derived Model dO3/dVOC (ppb/tons per day)
Year
(j)
Azusa
R²: 0.75
RMSE: 34.15
HDDM-derived Model O3 (ppb)
Observed O3 (ppb)
R²: 0.95
RMSE: 10.17
Obs-derived Model O3 (ppb)
Observed O3 (ppb)
R²: 0.89
RMSE: 0.05
HDDM-derived Model dO3/dNOx (ppb/tons per day)
Obs-derived Model dO3/dNOx (ppb/tons per day)
R²: 0.09
RMSE: 0.06
HDDM-derived Model dO3/dVOC (ppb/tons per day)
Obs-derived Model dO3/dVOC (ppb/tons per day)
Year
(k)
Pomona
R²: 0.74
RMSE: 42.11
HDDM-derived Model O3 (ppb)
Observed O3 (ppb)
R²: 0.94
RMSE: 9.48
Obs-derived Model O3 (ppb)
Observed O3 (ppb)
R²: 0.98
RMSE: 0.04
HDDM-derived Model dO3/dNOx (ppb/tons per day)
Obs-derived Model dO3/dNOx (ppb/tons per day)
R²: 0.14
RMSE: 0.05
HDDM-derived Model dO3/dVOC (ppb/tons per day)
Obs-derived Model dO3/dVOC (ppb/tons per day)
Year
(l)
Lake Elsinore
R²: 0.79
RMSE: 29.13
HDDM-derived Model O3 (ppb)
Observed O3 (ppb)
R²: 0.93
RMSE: 5.69
Obs-derived Model O3 (ppb)
Observed O3 (ppb)
R²: 0.99
RMSE: 0.03
HDDM-derived Model dO3/dNOx (ppb/tons per day)
Obs-derived Model dO3/dNOx (ppb/tons per day)
R²: 0.18
RMSE: 0.02
HDDM-derived Model dO3/dVOC (ppb/tons per day)
Obs-derived Model dO3/dVOC (ppb/tons per day)
Year

(m)

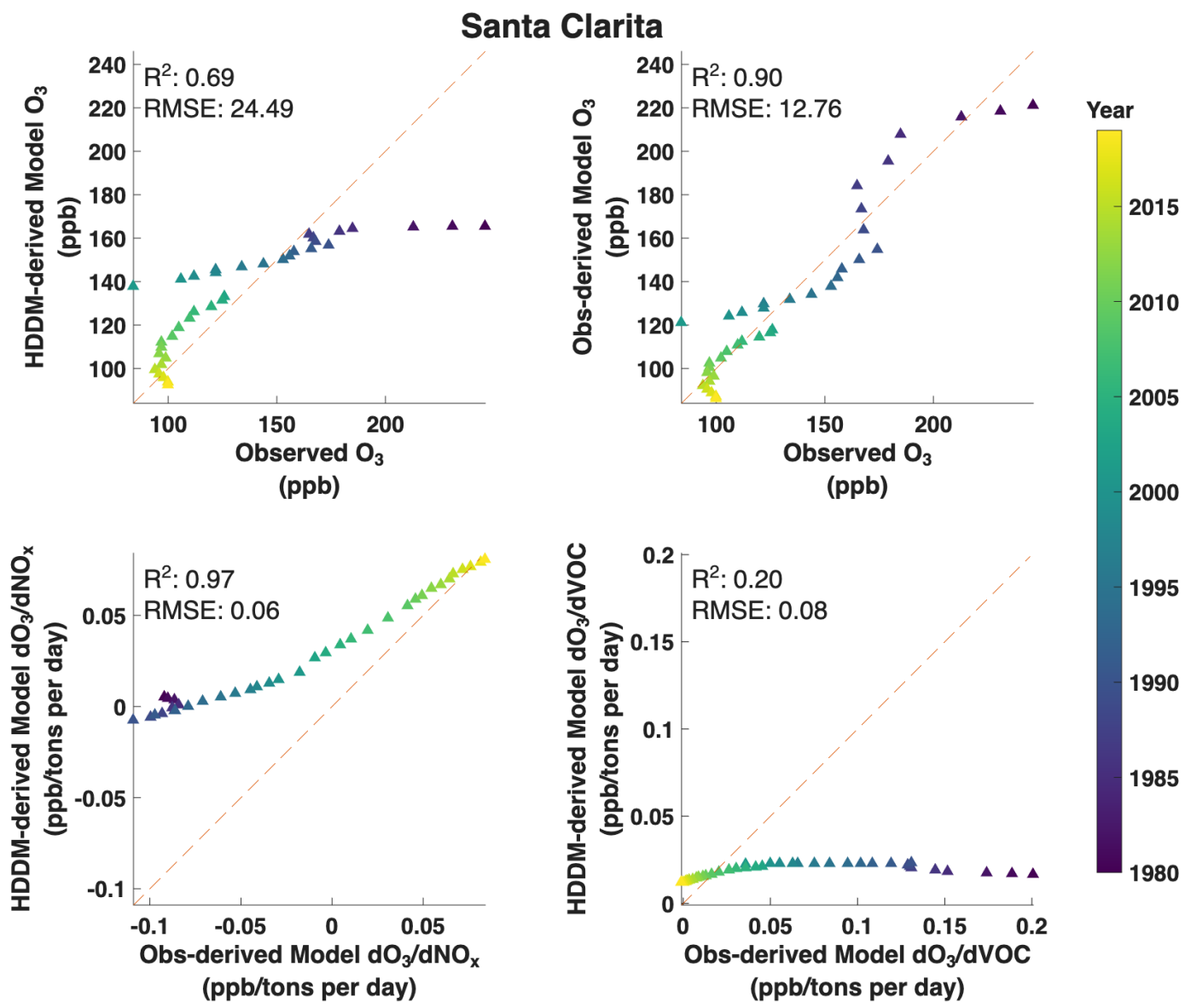

Santa Clarita
R^2: 0.69
RMSE: 24.49
HDDM-derived Model O$_3$ (ppb)
Observed O$_3$ (ppb)
R^2: 0.90
RMSE: 12.76
Obs-derived Model O$_3$ (ppb)
R^2: 0.97
RMSE: 0.06
HDDM-derived Model dO$_3$/dNO$_x$ (ppb/tons per day)
Obs-derived Model dO$_3$/dNO$_x$ (ppb/tons per day)
R^2: 0.20
RMSE: 0.08
HDDM-derived Model dO$_3$/dVOC (ppb/tons per day)
Obs-derived Model dO$_3$/dVOC (ppb/tons per day)
Year

(n)

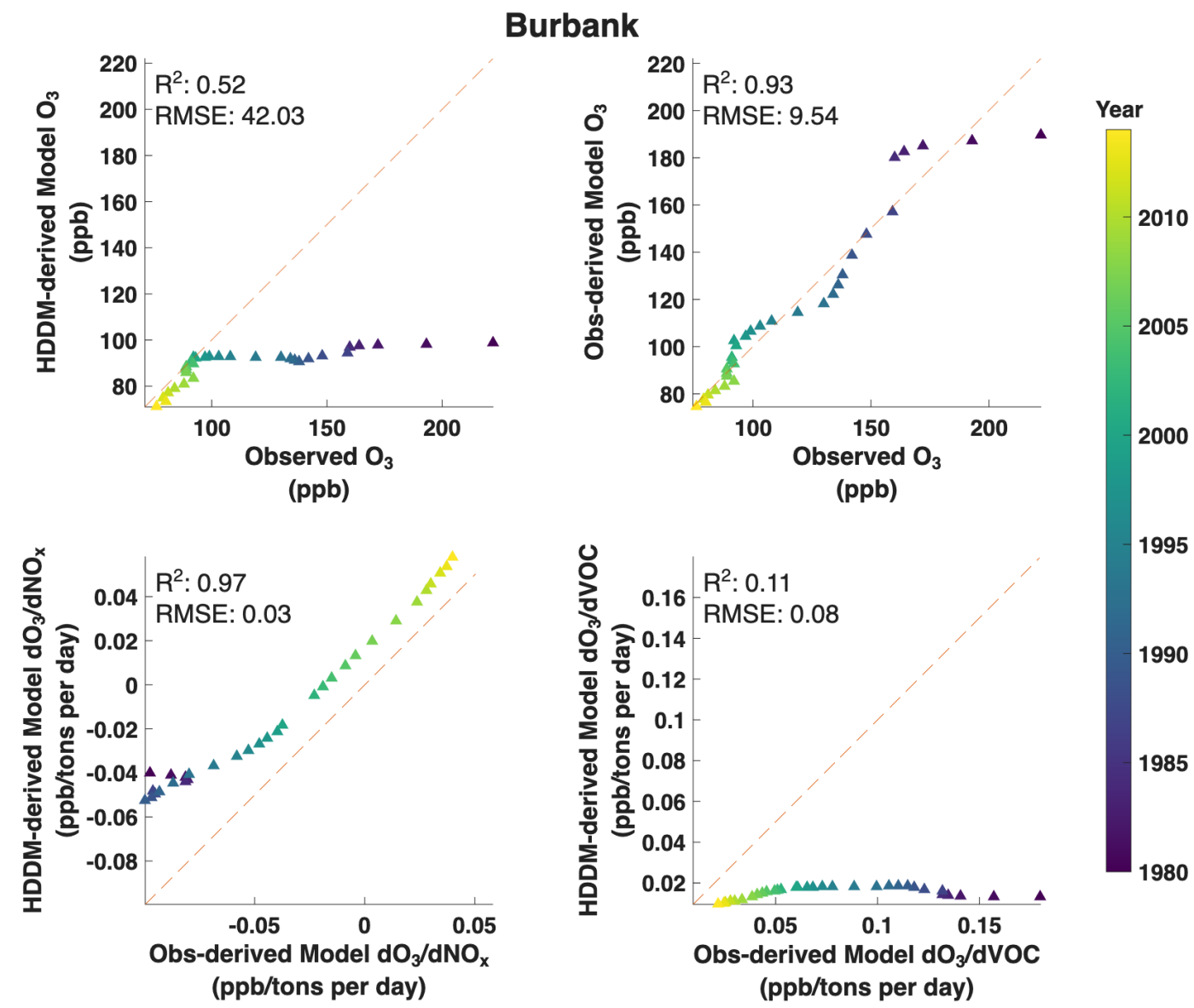

Burbank
R^2: 0.52
RMSE: 42.03
HDDM-derived Model O$_3$ (ppb)
Observed O$_3$ (ppb)
R^2: 0.93
RMSE: 9.54
Obs-derived Model O$_3$ (ppb)
R^2: 0.97
RMSE: 0.03
HDDM-derived Model dO$_3$/dNO$_x$ (ppb/tons per day)
Obs-derived Model dO$_3$/dNO$_x$ (ppb/tons per day)
R^2: 0.11
RMSE: 0.08
HDDM-derived Model dO$_3$/dVOC (ppb/tons per day)
Obs-derived Model dO$_3$/dVOC (ppb/tons per day)
Year

(o)

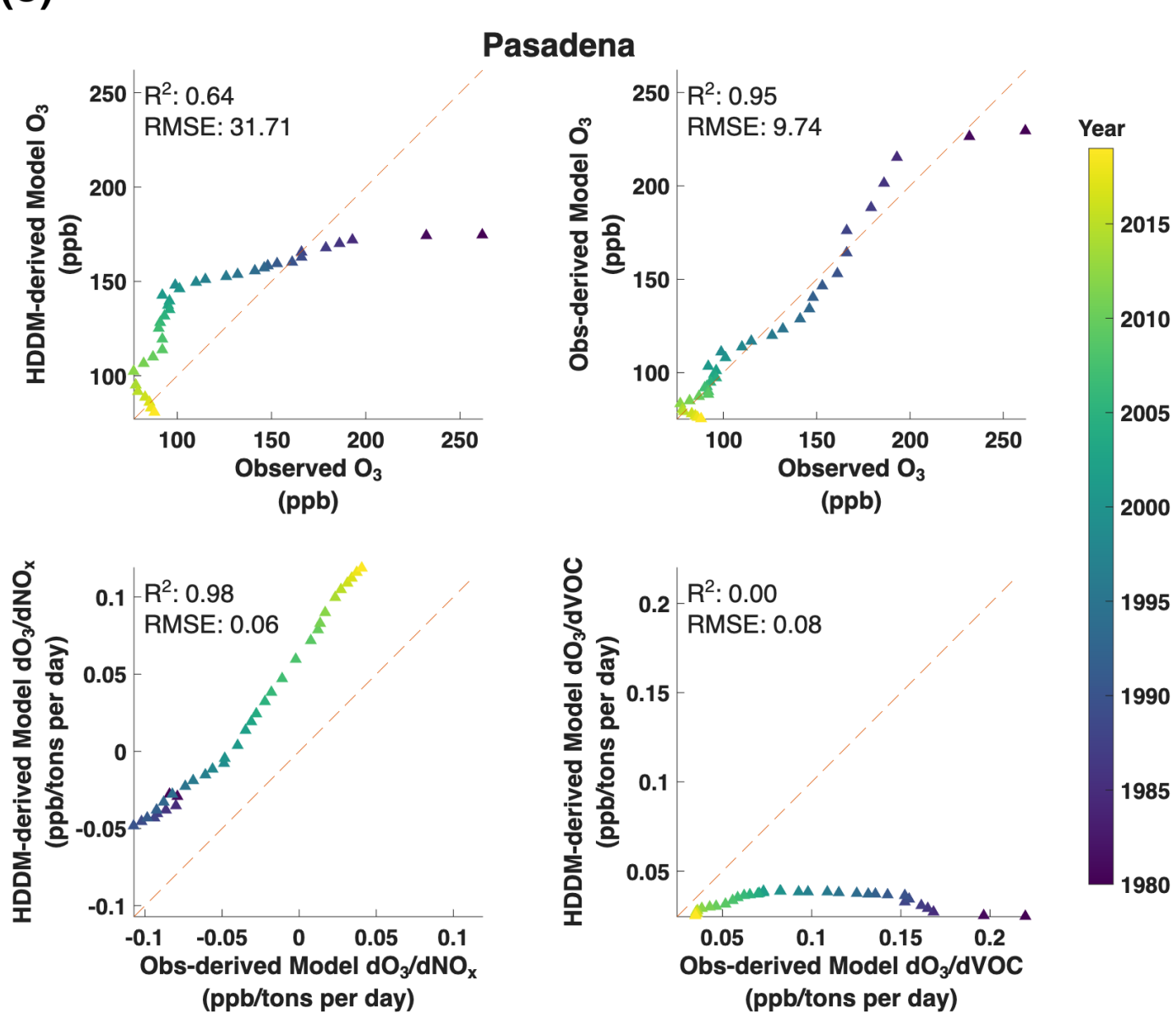

Pasadena
R^2: 0.64
RMSE: 31.71
HDDM-derived Model O$_3$ (ppb)
Observed O$_3$ (ppb)
R^2: 0.95
RMSE: 9.74
Obs-derived Model O$_3$ (ppb)
R^2: 0.98
RMSE: 0.06
HDDM-derived Model dO$_3$/dNO$_x$ (ppb/tons per day)
Obs-derived Model dO$_3$/dNO$_x$ (ppb/tons per day)
R^2: 0.00
RMSE: 0.08
HDDM-derived Model dO$_3$/dVOC (ppb/tons per day)
Obs-derived Model dO$_3$/dVOC (ppb/tons per day)
Year

(p)

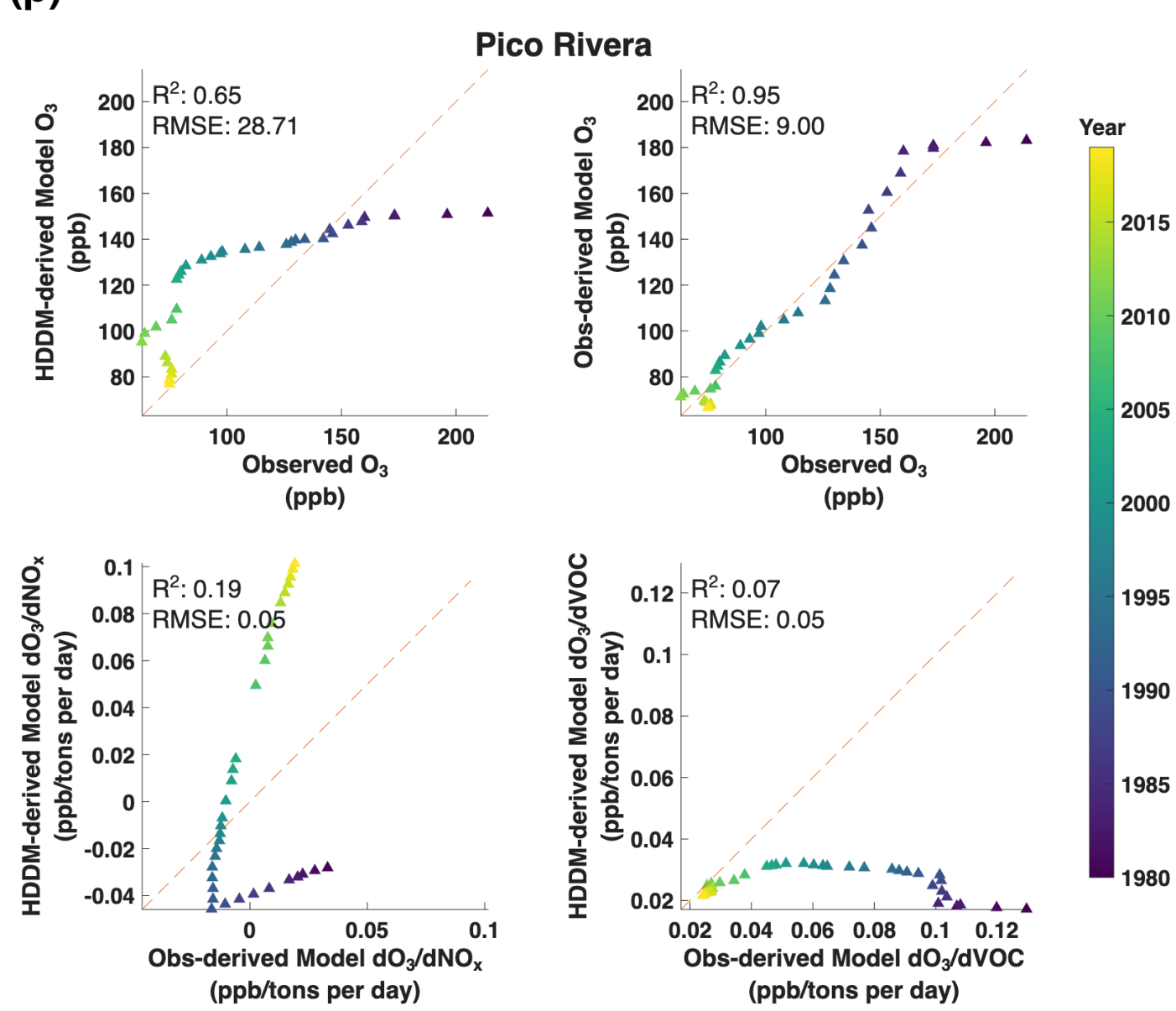

Pico Rivera
R^2: 0.65
RMSE: 28.71
HDDM-derived Model O$_3$ (ppb)
Observed O$_3$ (ppb)
R^2: 0.95
RMSE: 9.00
Obs-derived Model O$_3$ (ppb)
R^2: 0.19
RMSE: 0.05
HDDM-derived Model dO$_3$/dNO$_x$ (ppb/tons per day)
Obs-derived Model dO$_3$/dNO$_x$ (ppb/tons per day)
R^2: 0.07
RMSE: 0.05
HDDM-derived Model dO$_3$/dVOC (ppb/tons per day)
Obs-derived Model dO$_3$/dVOC (ppb/tons per day)
Year

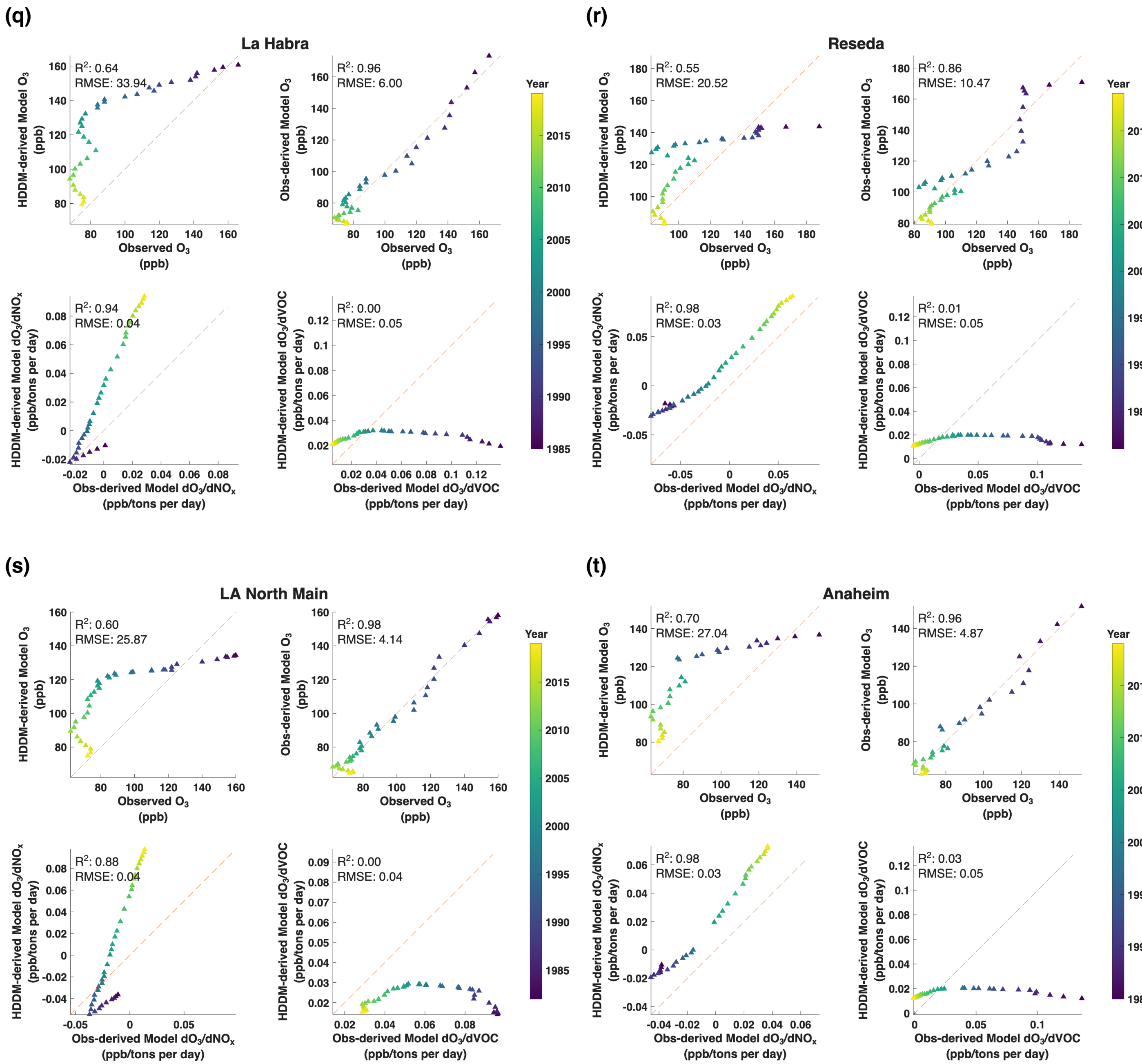
(q)
La Habra
R²: 0.64
RMSE: 33.94
R²: 0.96
RMSE: 6.00
R²: 0.94
RMSE: 0.04
R²: 0.00
RMSE: 0.05
(r)
Reseda
R²: 0.55
RMSE: 20.52
R²: 0.86
RMSE: 10.47
R²: 0.98
RMSE: 0.03
R²: 0.01
RMSE: 0.05
(s)
LA North Main
R²: 0.60
RMSE: 25.87
R²: 0.98
RMSE: 4.14
R²: 0.88
RMSE: 0.04
R²: 0.00
RMSE: 0.04
(t)
Anaheim
R²: 0.70
RMSE: 27.04
R²: 0.96
RMSE: 4.87
R²: 0.98
RMSE: 0.03
R²: 0.03
RMSE: 0.05
HDDM-derived Model O3 (ppb)
Obs-derived Model O3 (ppb)
Observed O3 (ppb)
HDDM-derived Model dO3/dNOx (ppb/tons per day)
HDDM-derived Model dO3/dVOC (ppb/tons per day)
Obs-derived Model dO3/dNOx (ppb/tons per day)
Obs-derived Model dO3/dVOC (ppb/tons per day)
Year

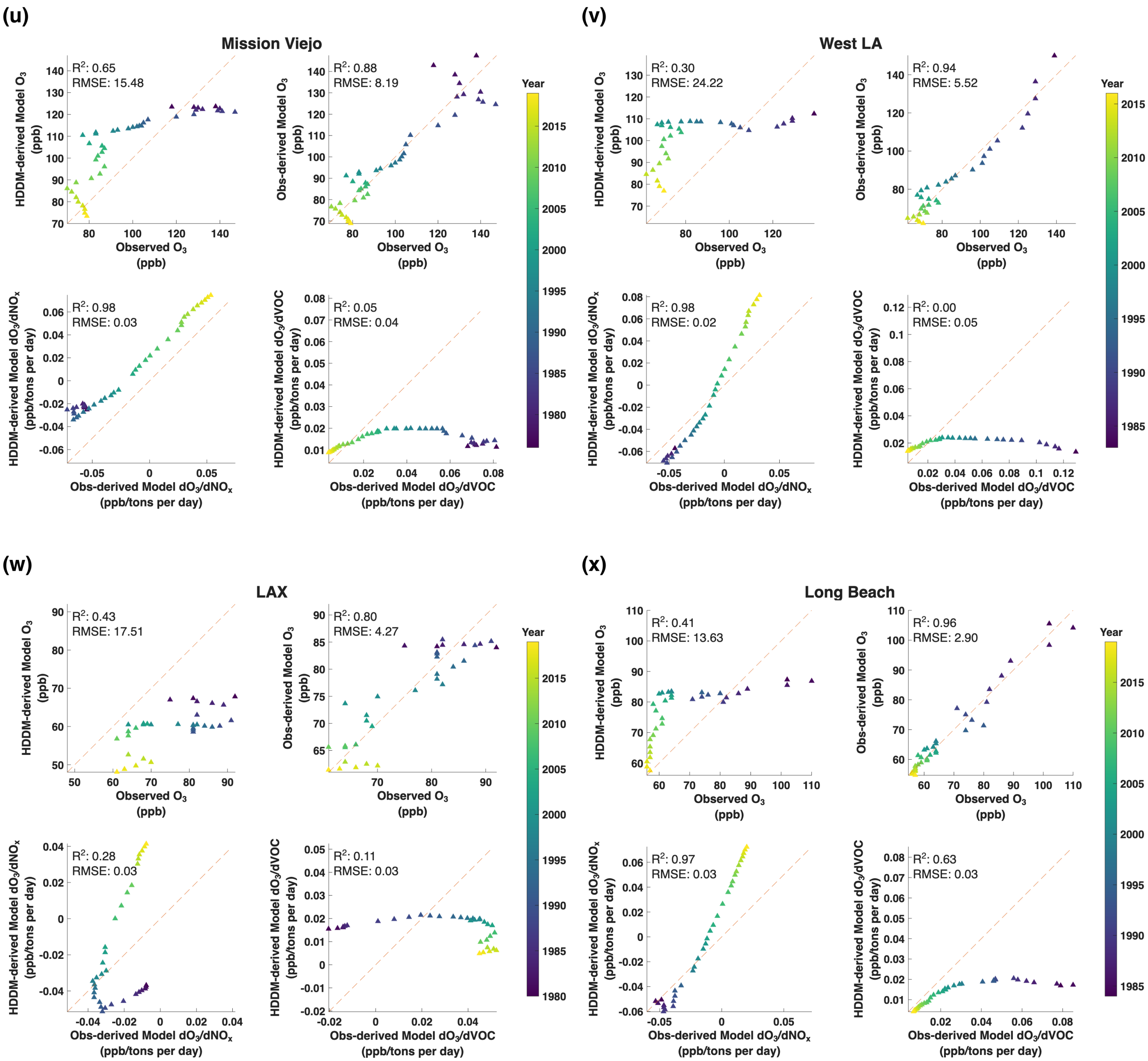

Figure S14. In each panel from a to x, the first row: comparison of observed ozone with CMAQ-HDDM- and observation-derived model estimates along emission levels from 1985 to 2019; the second row: comparisons between HDDM-derived and observation-derived sensitivity trends along emission levels from 1985 to 2019. The color indicates the year of the points. The dashed line indicated the 1:1 reference line.

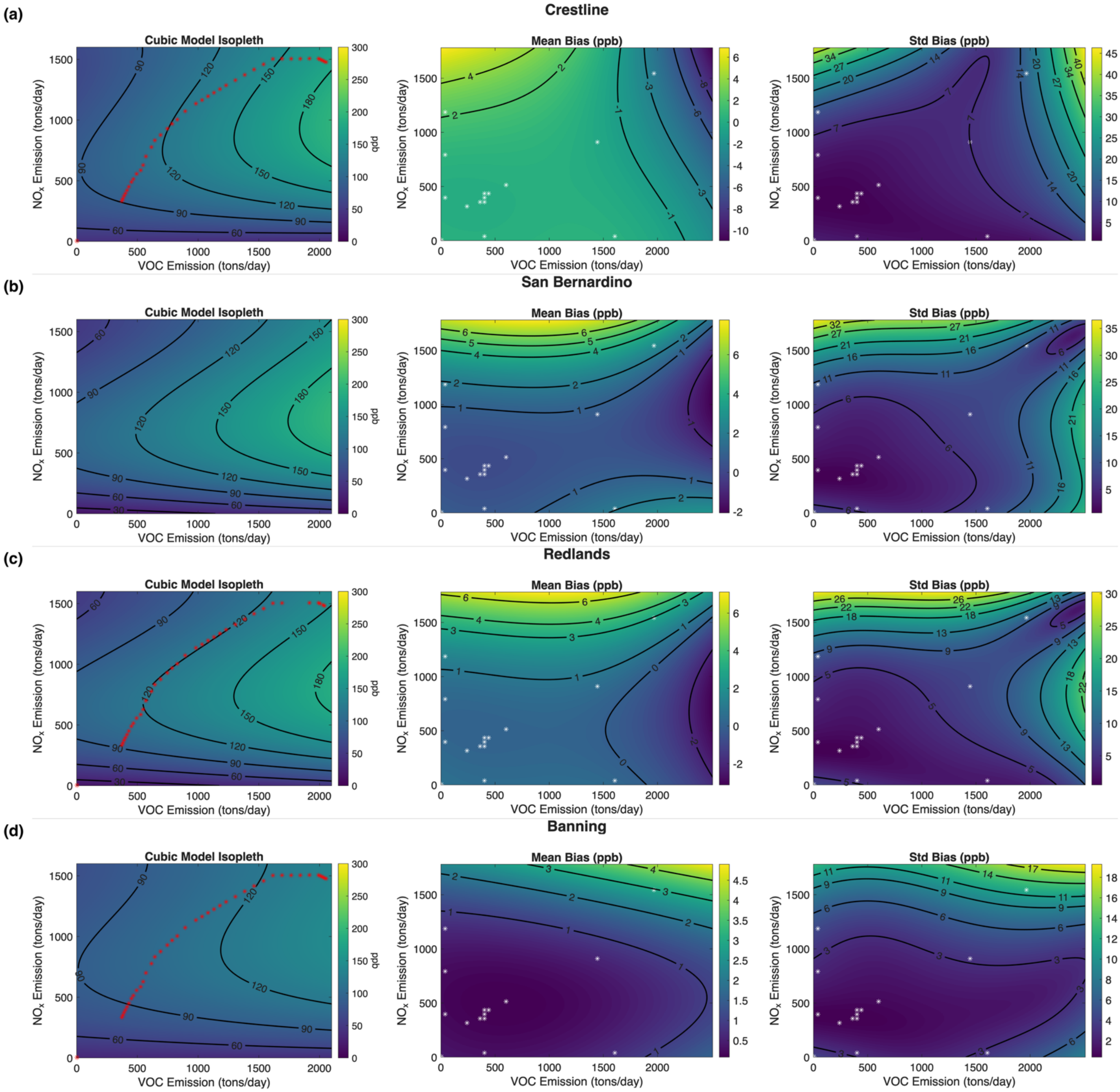
(a)
Crestline
(b)
San Bernardino
(c)
Redlands
(d)
Banning
Cubic Model Isopleth
Mean Bias (ppb)
Std Bias (ppb)
NOx Emission (tons/day)
VOC Emission (tons/day)
ppb

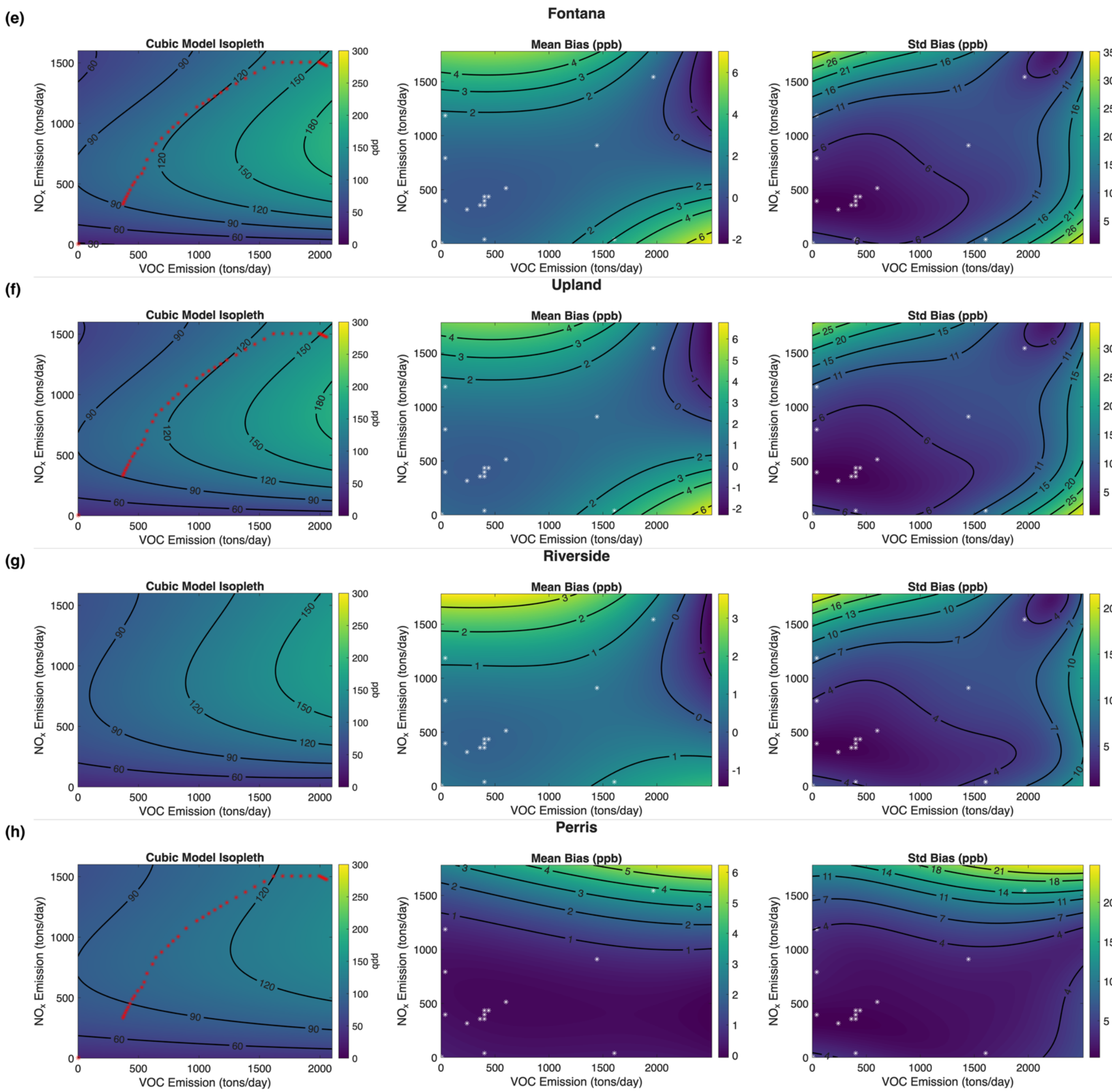
(e)
Fontana
(f)
Upland
(g)
Riverside
(h)
Perris
Cubic Model Isopleth
Mean Bias (ppb)
Std Bias (ppb)
NOx Emission (tons/day)
VOC Emission (tons/day)
ppb

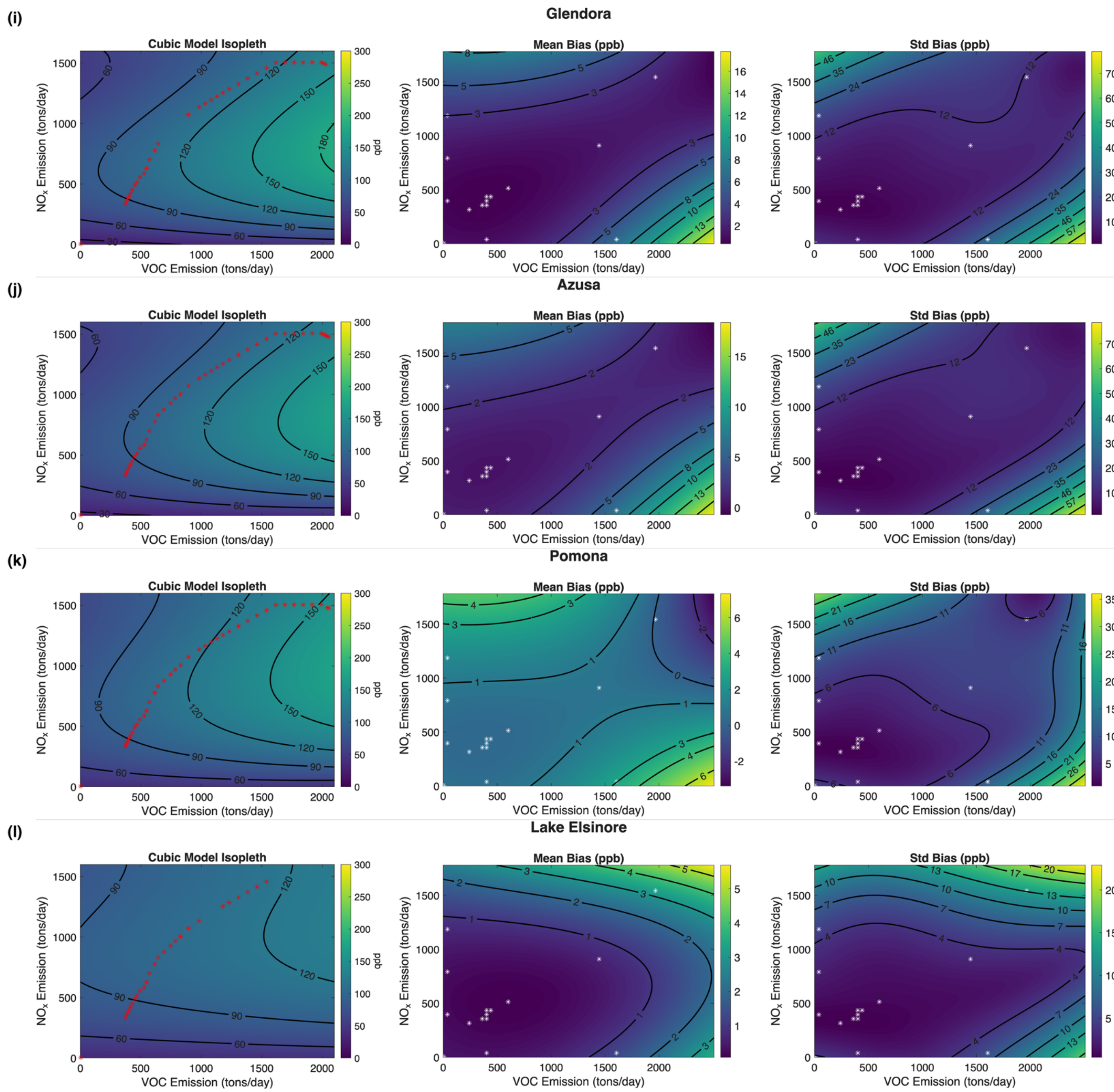
(i)
Glendora
(j)
Azusa
(k)
Pomona
(l)
Lake Elsinore
Cubic Model Isopleth
Mean Bias (ppb)
Std Bias (ppb)
NOx Emission (tons/day)
VOC Emission (tons/day)
ppb
0
500
1000
1500
2000

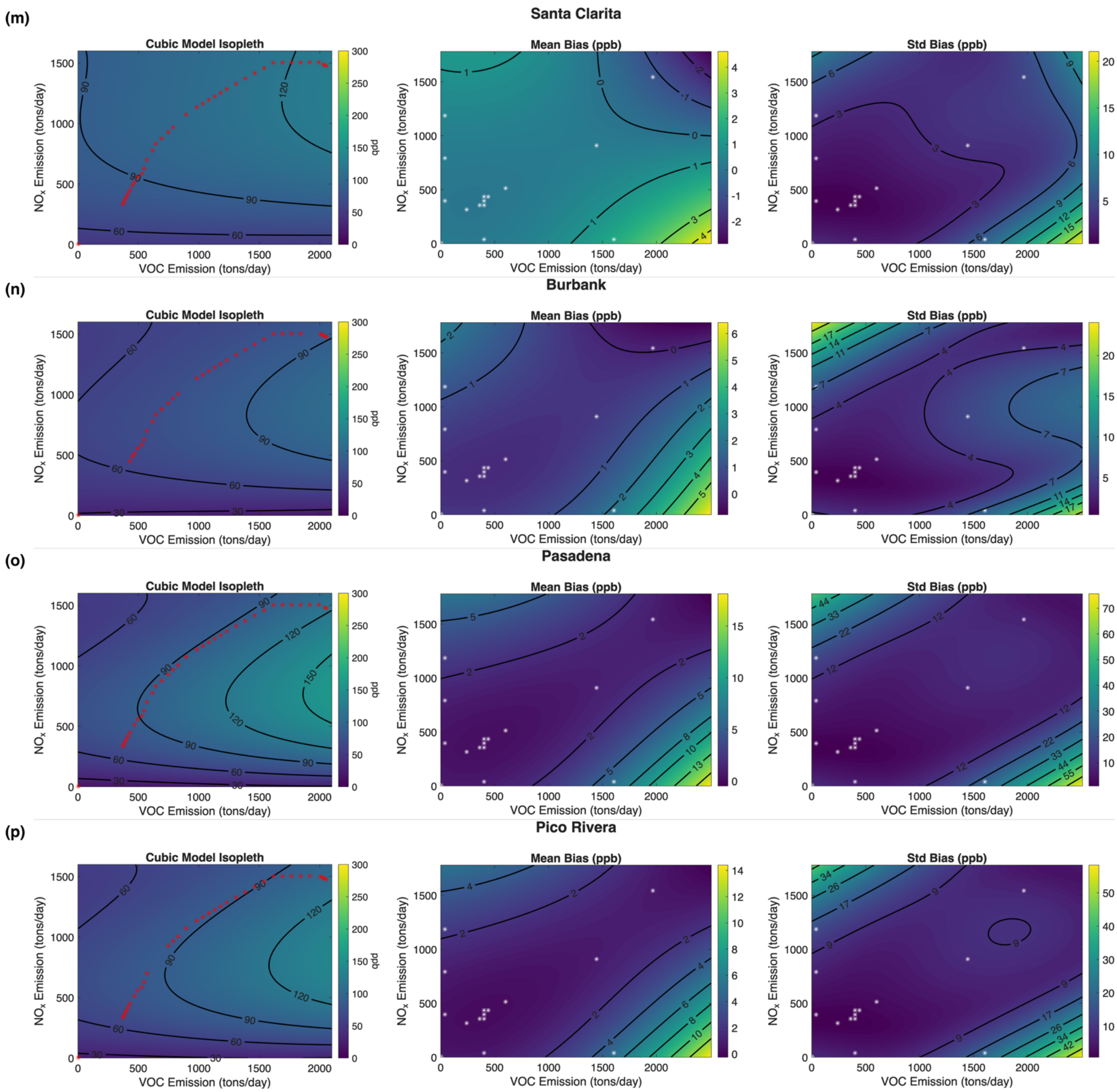
(m)
Santa Clarita
(n)
Burbank
(o)
Pasadena
(p)
Pico Rivera
Cubic Model Isopleth
Mean Bias (ppb)
Std Bias (ppb)
VOC Emission (tons/day)
NO_x Emission (tons/day)
ppb

(q) La Habra

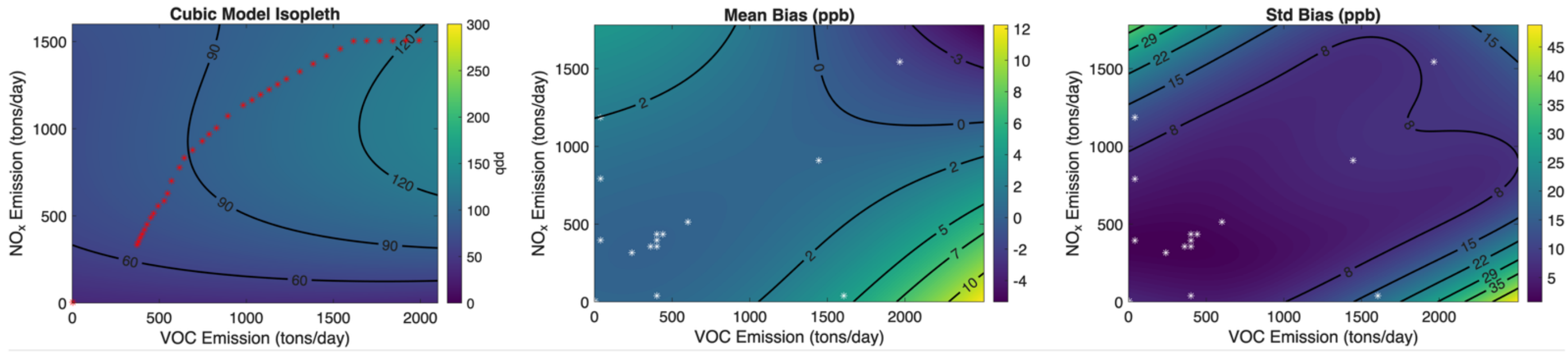

(r) Reseda

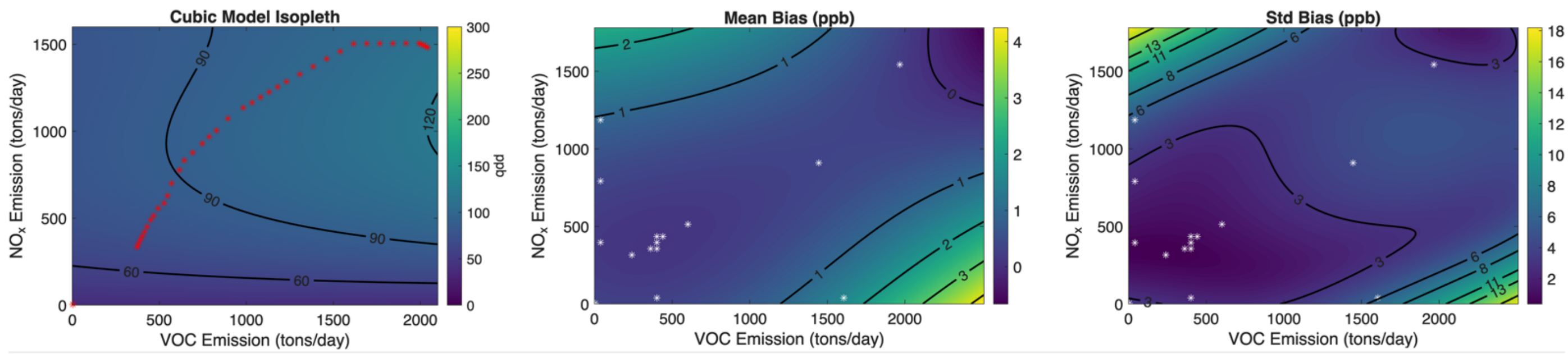

(s) LA North Main

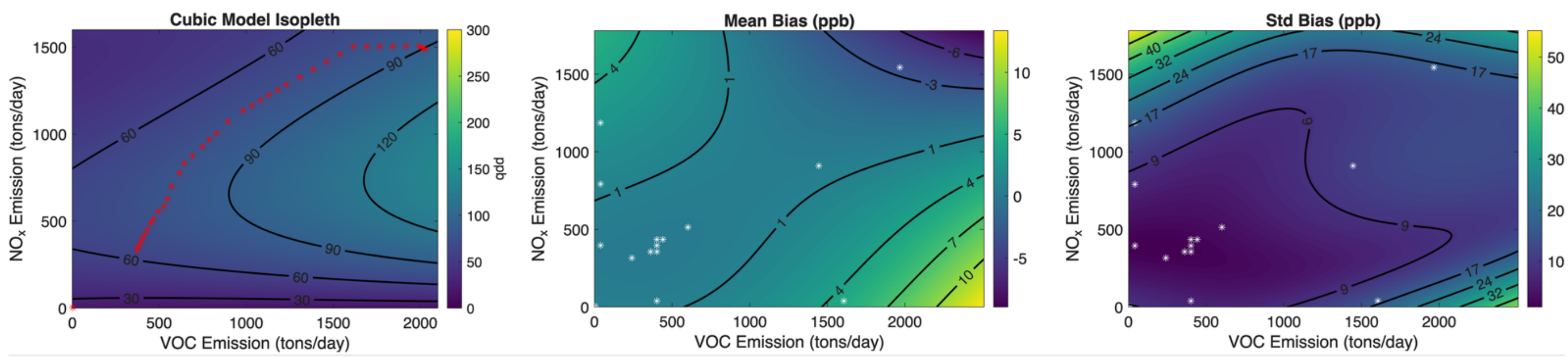

(t) Anaheim

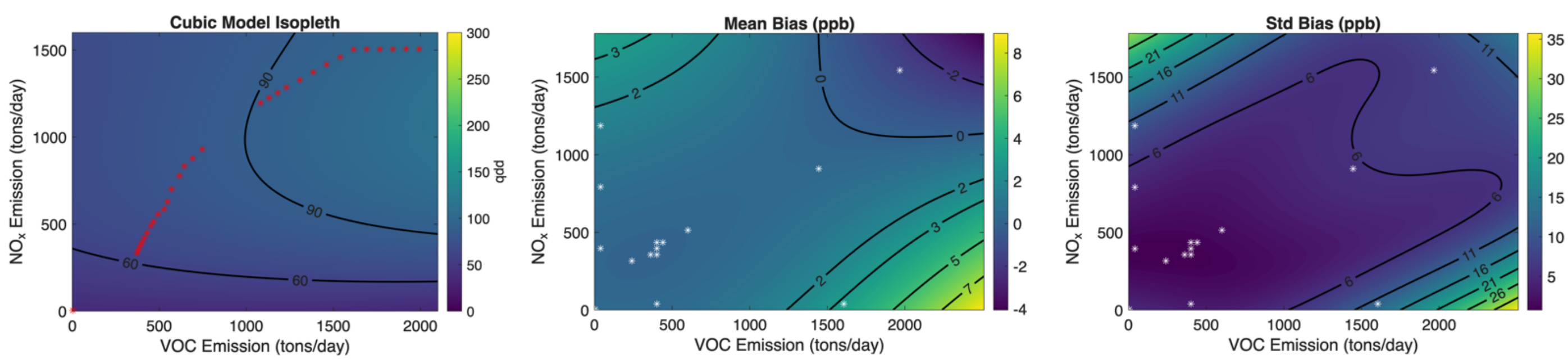

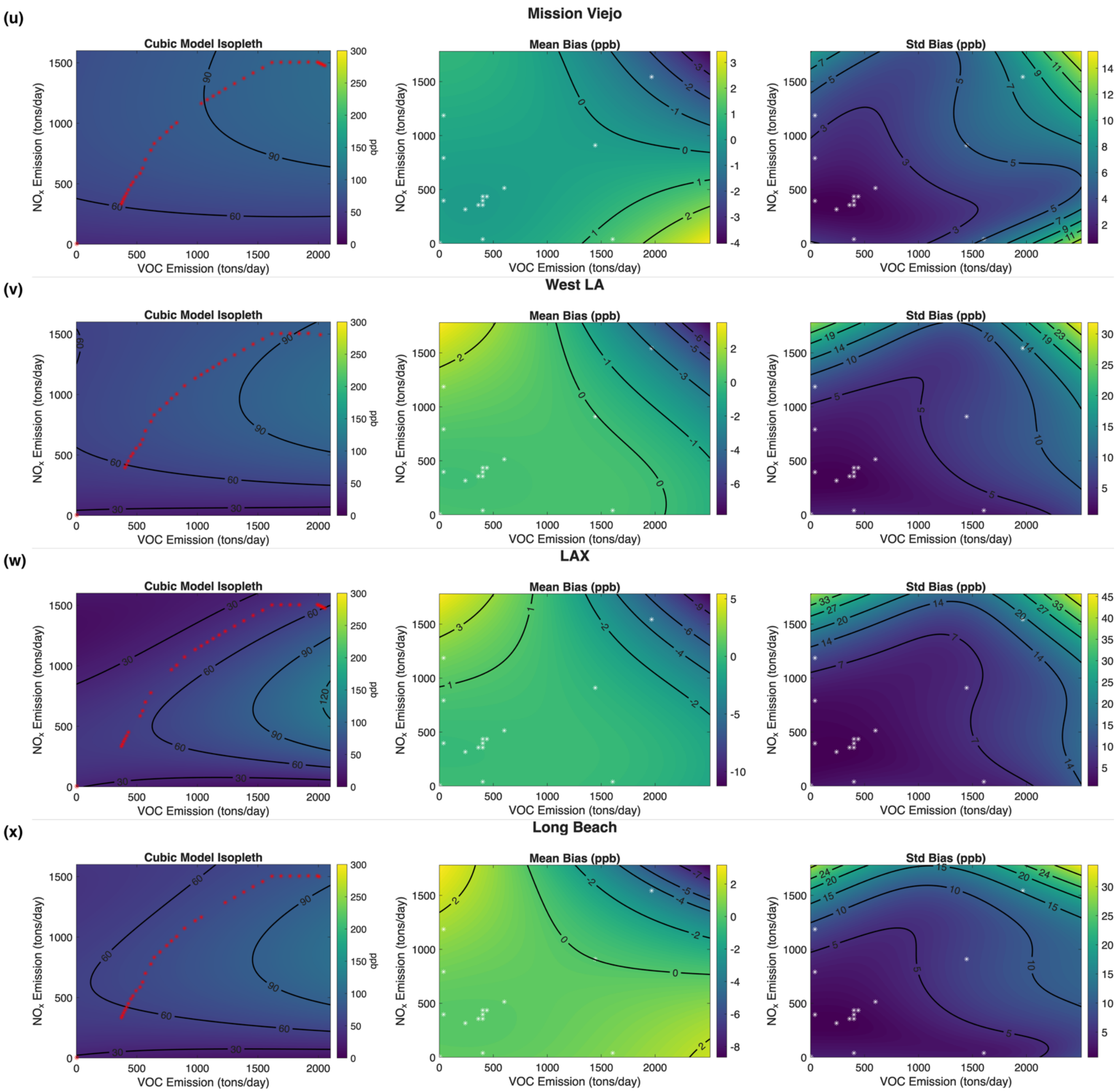

Figure S15. The first column in panels (a to x) shows the CMAQ-HDDM-derived ozone isopleth diagram using a cubic fit for the ozone-emission concentration relationship for 24 monitoring sites in the SoCAB. The red points show the historical emission trajectory from 1980 to 2019. The second and third columns show the mean bias and standard deviation of bias of CMAQ-HDDM-derived isopleths based the cubic model, respectively. The white crosses indicate the 15 emission levels used in the isopleth development.

REFERENCES

Emery, C., Z. Liu, A. G. Russell, M. T. Odman, G. Yarwood and N. Kumar (2017). "Recommendations on statistics and benchmarks to assess photochemical model performance." Journal of the Air & Waste Management Association **67**(5): 582-598.
